# Enantioselective direct Mannich-type reactions of 2-benzylpyridine N-oxides catalyzed by chiral bis(guanidino)iminophosphorane organosuperbase†
†Electronic supplementary information (ESI) available: Experimental procedures, screening of reaction conditions, and characterization data. CCDC 1811498. For ESI and crystallographic data in CIF or other electronic format see DOI: 10.1039/c8sc00808f


**DOI:** 10.1039/c8sc00808f

**Published:** 2018-04-20

**Authors:** Qiupeng Hu, Azusa Kondoh, Masahiro Terada

**Affiliations:** a Department of Chemistry , Graduate School of Science , Tohoku University , Aramaki, Aoba-ku , Sendai 980-8578 , Japan . Email: mterada@m.tohoku.ac.jp; b Research and Analytical Center for Giant Molecules , Graduate School of Science , Tohoku University , Aramaki, Aoba-ku , Sendai , 980-8578 , Japan

## Abstract

2-Benzylpyridine N-oxides possessing less acidic α-protons were utilized as pronucleophiles for the first time in enantioselective addition reactions under Brønsted base catalysis. A chiral bis(guanidino)iminophosphorane was able to overcome the inherent issue of low acidity of the pronucleophiles, establishing the diastereo- and enantioselective direct Mannich-type reaction with *N*-Boc imines. The control experiments indicated that the N-oxide moiety of the substrates played a critical role in achieving the high stereoselectivity.

## Abstract 2

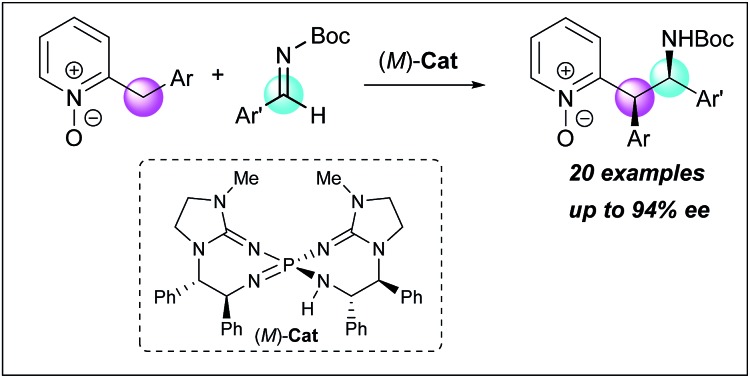
2-Benzylpyridine N-oxides possessing less acidic α-protons were utilized as pronucleophiles for the first time in enantioselective addition reactions under Brønsted base catalysis.

## Introduction

**Scheme 1:**
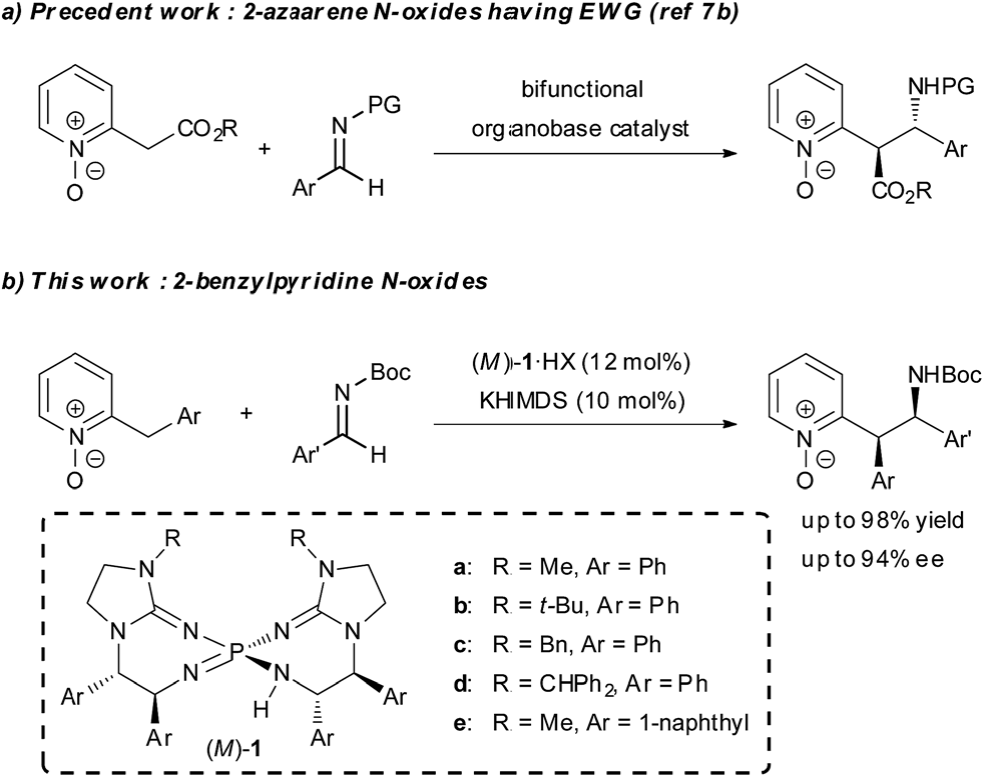
Catalytic enantioselective direct Mannich-type reactions of 2-alkylazaarene N-oxides.

Asymmetric organocatalysis has been recognized as one of the most important research areas in synthetic chemistry.^1^ In particular, asymmetric organobase catalysis has recently attracted great interest and made remarkable progress. A series of structurally diverse chiral organobase catalysts has been designed and successfully utilized in many catalytic enantioselective transformations.^2^ Compared to the vast majority of examples involving the enolate formation of carbonyl compounds in asymmetric organobase catalysis, the strategy using direct α-deprotonation of 2-alkylazaarenes, which is strongly associated with enolate formation,^3^ is rarely utilized in catalytic enantioselective reactions to synthesize 2-substituted azaarenes with a stereogenic center at the *α*-position. Such azaarenes are the common units existing in natural products and pharmaceuticals.^4^ The main obstacle to establishing such reactions is the low acidity of the α-protons in the 2-alkyl substituents, which impedes the deprotonation required for the initiation of the catalytic process. The principal strategy for overcoming the obstacle is the introduction of additional strong electron-withdrawing groups on either the azaarenes rings or the α-carbon atoms for acidity enhancement.^5^ On the other hand, the application of azaarene N-oxides, which serve as azaarene surrogates having higher electron-deficient properties, is an alternative strategy. The N-oxide moiety increases the acidity of the α-proton in the 2-alkyl substituent,^6^ and thus facilitates deprotonation. In addition, the N-oxide moiety is easily removable under mild conditions. Recently, the Palomo group reported related work on bifunctional organobase-catalyzed asymmetric addition reactions of 2-(cyanomethyl)azaarene N-oxides and N-oxides of 2-azaaryl acetates by using this strategy (Scheme 1a).^7^ However, the reported asymmetric reactions of 2-alkylazaarene N-oxides still required an additional strong electron-withdrawing group on the α-carbon, and the reactions of simple 2-alkylazaarene N-oxides were not explored due to their insufficient acidity for direct deprotonation by conventional chiral organobase catalysts. Meanwhile, recently, we developed a novel family of pseudo-*C*_2_-symmetric bis(guanidino)iminophosphoranes (*M*)-**1** (Scheme 1b), which possess much higher basicity than those of reported chiral organobases.^8^ As a part of our effort to expand the scope of pronucleophiles in asymmetric transformations under Brønsted base catalysis, herein, we describe the use of chiral organosuperbase bis(guanidino)iminophosphoranes (*M*)-**1** in the direct Mannich-type reaction of 2-benzylpyridine N-oxides with *N*-Boc imines in an enantioselective manner (Scheme 1b). Chiral bis(guanidino)iminophosphoranes (*M*)-**1** could successfully overcome the obstacle to the direct use of less acidic pronucleophiles, such as 2-benzylazaarene derivatives under catalytic conditions,^9^ with only the assistance of a removable N-oxide moiety and accelerated the reaction with the construction of two adjacent stereogenic centers in good diastereo- and enantioselectivities. This is a rare example of direct construction of a stereogenic center at the less acidic *α*-position of an azaarene derivative under Brønsted base catalysis.^10^–^12^

## Results and discussion

We began our study by choosing *N*-Boc imine **3a**, which was found to be a promising electrophile, and N-oxide **2a** (see Table S1 in the ESI† for details). The corresponding adduct was provided in 83% yield with *syn*/*anti* = 78 : 22 and 89% ee for the major isomer using (*M*)-**1a** generated *in situ* by treating the HBr salt of (*M*)-**1a** with NaHMDS in toluene (Table 1, entry 1). To optimize the reaction conditions, other bis(guanidino)iminophosphoranes (*M*)-**1b–d** with different substituents on the guanidine subunits were tested first. As shown in Table 1, (*M*)-**1a** with a methyl substituent on the guanidine subunits exhibited the highest catalytic efficiency from the viewpoint of both yield and stereoselectivity (entries 2–4). Replacing the phenyl groups on the 7,7-membered spirocyclic ring with 1-naphthyl groups, giving (*M*)-**1e**, did not improve the outcome (entry 5). Second, the effects of solvents and inorganic bases for the generation of the catalyst from precatalyst (*M*)-**1a**·HBr were evaluated. Toluene and benzene were clearly superior to the other common solvents (entries 1 and 9 *vs.* 6–8). The screening of inorganic bases showed that the counter cation had a marked influence on both diastereoselectivity and enantioselectivity. NaH gave similar results to NaHMDS (entries 9 and 10); however, in contrast to NaHMDS, LiHMDS exhibited a reversal in the diastereomeric ratio (entry 11). The best result was obtained with the combination of benzene as a solvent and KHMDS as an inorganic base, producing **4aa** with *syn*/*anti* = 83 : 17 and 91% ee for the major isomer (entry 12).^13^ No further improvement was achieved even by screening of reaction conditions with KHMDS as an inorganic base (entries 13 and 14), altering the carbamate moiety on the imines, adding additives, or decreasing the reaction temperature (see Tables S2 and S3 in the ESI† for details).

**Table 1:** Optimization of reaction conditions^*a*^

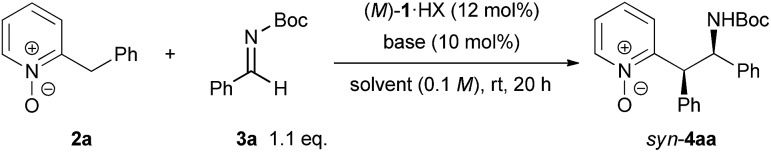						
Entry	**1**·HX	Base	Solvent	Yield^*b*^ (%)	*Syn*/*anti*^*c*^	ee^*c*^ (%)
1	**1a**·HBr	NaHMDS	Toluene	83	78 : 22	89/5
2	**1b**·HCl	NaHMDS	Toluene	82	66 : 34	75/21
3	**1c**·HCl	NaHMDS	Toluene	67	55 : 45	58/9
4	**1d**·HCl	NaHMDS	Toluene	28	40 : 60	26/36
5	**1e**·HBr	NaHMDS	Toluene	69	71 : 29	89/21
6	**1a**·HBr	NaHMDS	THF	74	39 : 61	47/2
7	**1a**·HBr	NaHMDS	EtOAc	87	45 : 55	67/24
8	**1a**·HBr	NaHMDS	Et_2_O	93	60 : 40	72/0
9	**1a**·HBr	NaHMDS	Benzene	85	81 : 19	91/4
10	**1a**·HBr	NaH	Benzene	77	78 : 22	88/0
11	**1a**·HBr	LiHMDS	Benzene	74	22 : 78	32/4
12	**1a**·HBr	KHMDS	Benzene	83	83 : 17	91/10
13	**1b**·HBr	KHMDS	Benzene	83	71 : 29	76/21
14	**1a**·HBr	KHMDS	Toluene	80	78 : 22	87/7

^*a*^Reaction conditions: **2a** (0.10 mmol), **3a** (0.11 mmol), (*M*)-**1**·HX (0.012 mmol), base (0.010 mmol), solvent (1.0 mL), rt, 20 h.

^*b*^The combined yield of the diastereomeric mixtures is indicated.

^*c*^The *syn*/*anti* ratios and ee values were determined by chiral HPLC analysis.

**Scheme 2:**
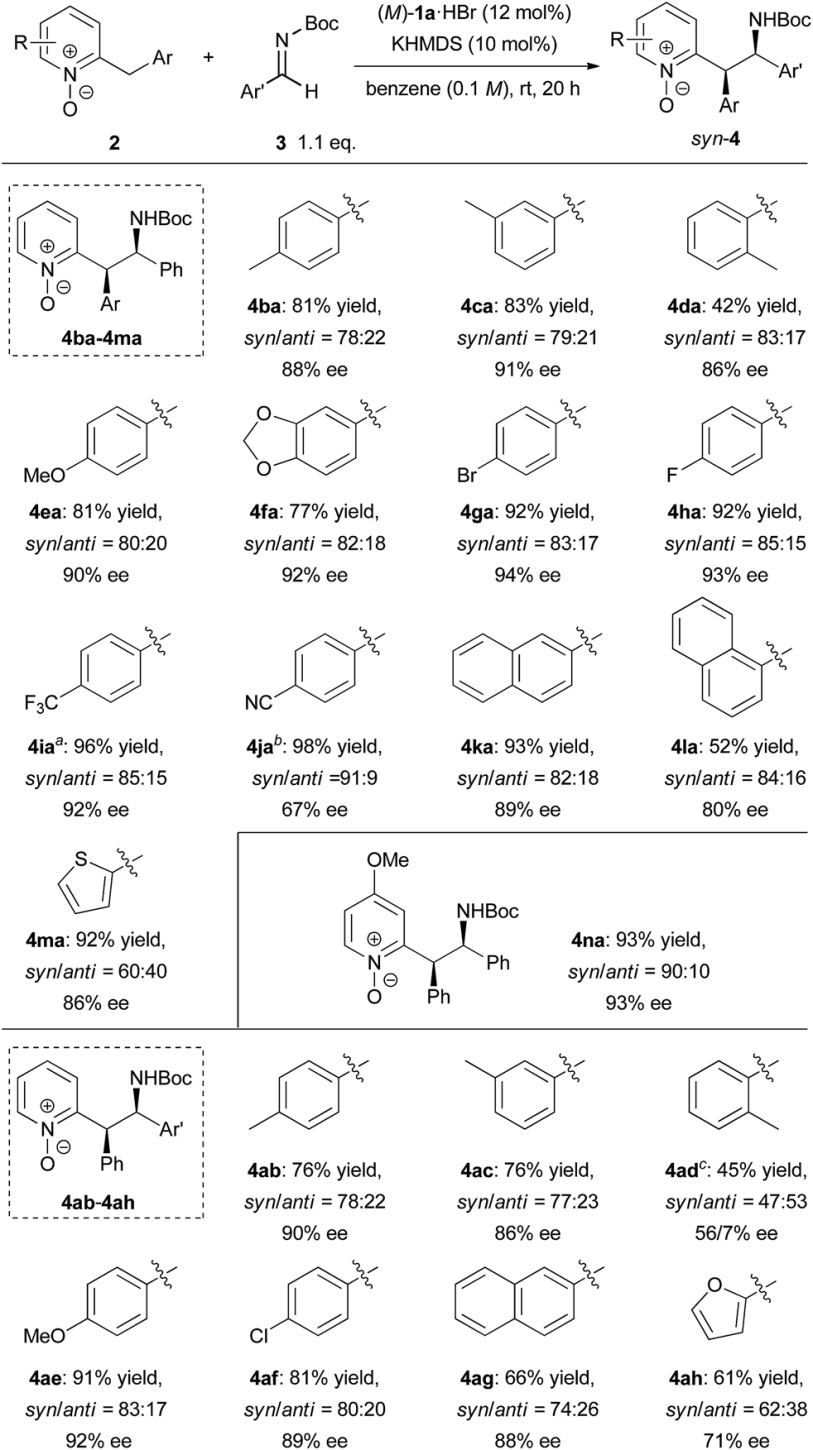
Substrate scope. Unless stated otherwise, the reaction conditions were as follows: **2** (0.10 mmol), **3** (0.11 mmol), (*M*)-**1a**·HBr (0.012 mmol), KHMDS (0.010 mmol), benzene (1.0 mL), rt, 20 h. The combined yield of the diastereomeric mixtures is indicated. The dr and ee values were determined by chiral stationary phase HPLC analysis. The ee values of the major isomers are indicated. ^a^The reaction was conducted in toluene at 0 °C and the concentration was 0.025 M.^b^The reaction was conducted in toluene at –30 °C for 4 h. ^c^The absolute configuration was not confirmed. The minor and major isomers were obtained with 56% ee and 7% ee, respectively.

With the optimized conditions in hand, the generality of the reaction was demonstrated by using a variety of 2-benzylpyridine N-oxides **2** and imines (Scheme 2). In studying the scope of 2-benzylpyridine N-oxides **2** as the pronucleophile, both high yields and enantioselectivities were achieved with **2b** and **2c** possessing a methyl substituent at the *para* or *meta* position on the phenyl ring. In contrast, **2d** with a methyl substituent at the *ortho* position gave a lower yield of 42% with 84% ee. The reactions of **2e** and **2f** having electron-donating 4-methoxy and 3,4-methylenedioxy substituents on the phenyl ring, respectively, also proceeded smoothly to afford desired **4ea** and **4fa** both with 90% ee. Substrates **2g**, **2h**, and **2i** containing electron-withdrawing groups, such as fluoro, bromo, and trifluoromethyl groups, on the phenyl ring showed higher reactivity, and the corresponding products were obtained in high yields with excellent enantioselectivities. In the case of **2j** having a cyano group, however, racemic **4ja** was obtained with a low *syn*/*anti* ratio under the optimized reaction conditions.^14^ This result strongly suggests that the reverse reaction was also accelerated at room temperature. In order to suppress the reverse reaction, the temperature was reduced to –30 °C. As a result, the undesired reverse pathway was partially blocked and the enantio-enriched product **4ja** was obtained with *syn*/*anti* = 91 : 9 and moderate ee of 67%. The reactions of both 2-naphthyl- and 1-naphthyl-substituted **2k** and **2l** proceeded with good enantioselectivities, albeit in a moderate yield with **2l**. Substrate **2m** having a 2-thienyl group was also applicable to the reaction, and the corresponding product was obtained with moderate diastereoselectivity and good enantioselectivity. In the case of **2n** possessing a methoxy group at the 4-position of the pyridine ring, both high reactivity (93% yield) and stereoselectivity (*syn*/*anti* = 89 : 11, 92% ee) were achieved. The scope of imines as electrophiles was then investigated. Most of the reactions of aromatic imines, including both electron-rich and electron-deficient ones, proceeded smoothly to provide the corresponding products in high yields with good diastereo- and enantioselectivities. The only exception was the reaction of *o*-tolyl imine **3d**. In this case, low diastereoselectivity was observed and the minor isomer of **4ad** gave a higher ee of 56%. Heteroaromatic imine **3h** was also applicable to provide **4ah** in 61% yield with moderate *syn*/*anti* = 62 : 38 and 71% ee.

**Scheme 3:**
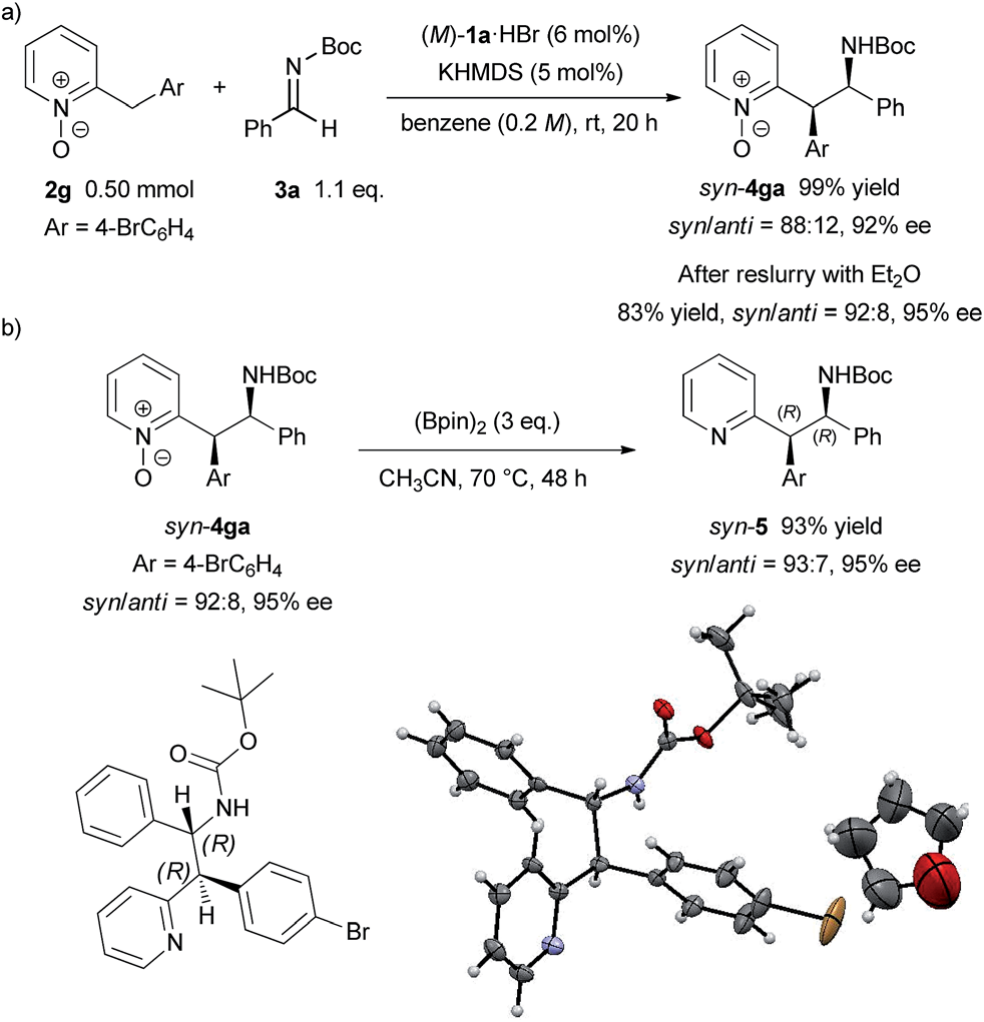
Larger scale reaction with lower catalyst loading and removal of N-oxide moiety. Single-crystal X-ray analysis of **5**. Thermal ellipsoids are shown at 50% probability.

To demonstrate the utility of the present method, a larger scale experiment with a lower catalyst loading and removal of the N-oxide moiety were examined (Scheme 3). The larger scale reaction proceeded very well in the presence of 5 mol% catalyst to afford **4ga** in quantitative yield with only a slight decrease of diastereomeric ratio and enantiomeric excess (Scheme 3a). The adduct **4ga** was then subjected to reslurry in Et_2_O and both the diastereomeric ratio and enantiomeric excess were improved. Treatment of product **4ga** with bis(pinacolato)diboron easily removed the N-oxide moiety in quantitative yield without any loss in diastereomeric ratio and enantiomeric excess (Scheme 3b).^15^ The absolute configuration of **5** was unambiguously confirmed as (1*R*, 2*R*) by X-ray single-crystal diffraction analysis.^16^

**Scheme 4:**
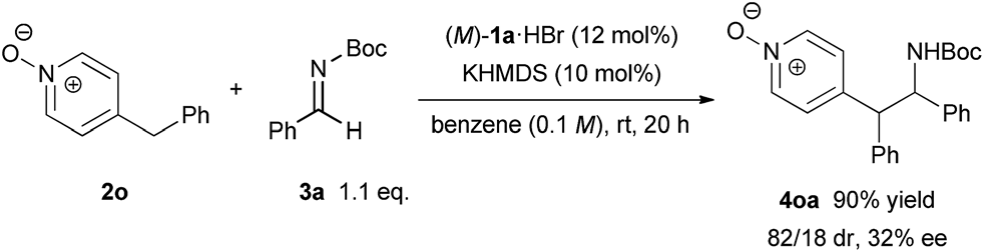
Control experiment.

In order to illustrate the primary role of the N-oxide moiety in our reaction system,^17^ 4-benzylpyridine N-oxide **2n**^6^ was tested in our catalytic system (Scheme 4). As a result, the reaction proceeded smoothly with good diastereoselectivity but a low ee value of 32% was observed. This result reveals that the N-oxide moiety proximate to the nucleophilic site is essential to control the stereochemical outcome in a highly enantioselective manner. The N-oxide moiety presumably acted as an additional coordination site^7^,^18^ for the bis(guanidino)iminophosphorane catalyst, where the orientation of the nucleophilic pyridine N-oxides would be well-organized under the chiral environment created by the catalyst.

## Conclusions

In conclusion, 2-benzylpyridine N-oxides were successfully utilized as less acidic pronucleophiles in enantioselective direct Mannich-type reactions by using a bis(guanidino)iminophosphorane as a chiral organosuperbase catalyst. The addition reactions to *N*-Boc imines proceeded efficiently with good diastereo- and enantioselectivities, which is a rare example of the direct construction of a stereogenic center at the less acidic *α*-position of an azaarene derivative under Brønsted base catalysis. The control experiments disclosed the critical role of the N-oxide moiety for achieving high stereoselectivity. More detailed studies to reveal the mechanism of the stereocontrol are ongoing.

## Conflicts of interest

There are no conflicts to declare.

## Supplementary Material

Supplementary information

Crystal structure data
