# Supplementary material for: Enantioselective direct Mannich-type reactions of 2-benzylpyridine N-oxides catalyzed by chiral bis(guanidino)iminophosphorane organosuperbase
Source: Chem Sci. 2018 Apr 20;9(18):4348–51. doi: 10.1039/c8sc00808f (PMC5944382; doi:10.1039/c8sc00808f)
Supplement: Supplementary file 1 [file SC-009-C8SC00808F-s001.pdf]

Electronic Supplementary Material (ESI) for Chemical Science.
This journal is © The Royal Society of Chemistry 2018

Electronic Supplementary Information

Enantioselective Direct Mannich-Type Reactions of 2-Benzylpyridine *N*-Oxides Catalyzed by Chiral Bis(guanidino)iminophosphorane Organosuperbase

*Qiupeng Hu, Azusa Kondoh, and Masahiro Terada**

Table of Contents

1. General information S2
2. The details for optimization of reaction conditions S3
3. Synthesis of 2-benzylpyridine and their derivatives **S2-S14** S5
    3.1 Typical procedure for the substituted 2-benzylpyridine (**S2-S6**, **S8-S13**) S5
    3.2 Synthesis of 2-(4-bromobenzyl)pyridine **S7** S5
    3.3 Synthesis of 2-benzyl-4-methoxypyridine **S14** S6
4. Synthesis of 2-benzylpyridine *N*-oxide and their derivatives **2a-n** S9
    4.1 Typical procedure for the substituted 2-benzylpyridine *N*-oxides S9
5. Catalytic enantioselective direct Mannich-type reactions S13
    5.1 Typical procedure S13
6. Catalytic reaction in larger scale and reduction of pyridine *N*-oxide S24
7. HPLC Charts S26
8. NMR Spectra S49
9. Referances S96

1. General information

Unless otherwise noted, the reactions were carried out with dried glassware under argon or nitrogen atmosphere. Analytical thin layer chromatography (TLC) was performed on Merck precoated TLC plates (silica gel 60 GF254, 0.25 mm). Flash column chromatography was performed on silica gel 60N (spherical, neutral, 40-50 m; Kanto Chemical Co., Inc.). ^{1}H NMR spectra were recorded on a JEOL JNM-ECA600 (600 MHz) spectrometer. Chemical shifts are reported in ppm from the solvent resonance or tetramethylsilane (TMS) as the internal standard. Data are reported as follows: chemical shift, integration, multiplicity (s = singlet, d = doublet, t = triplet, q = quartet, m = multiplet, br = broad) and coupling constants (Hz). ^{13}C NMR spectra were recorded on a JEOL JNM-ECA600 (150 MHz) spectrometer with complete proton decoupling. Chemical shifts are reported in ppm from the solvent resonance as the internal standard. Optically rotations were measured on a Jasco P-1020 digital polarimeter with a sodium lamp and reported as follows; $[\alpha]^{T}_{D}$ (c = g/100 mL, solvent). HPLC was performed on JASCO HPLC systems. High resolution mass spectra analysis was performed on a Bruker Daltonics solariX 9.4T FT-ICR-MS spectrometer at the Research and Analytical Center for Giant Molecules, Graduate School of Science, Tohoku University.

2. The details for optimization of reaction conditions

Table S1. Details for screening of PG of imines.[a]

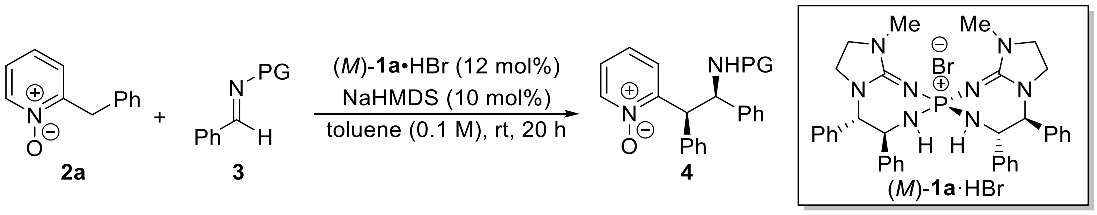

Entry	Solv.	PG	Yield [%][b]	*syn*/*anti*[c]	ee [%][c]
1	toluene	Boc	83	78:22	89/5
2	toluene	Alloc	89	56:44	67/17
3	toluene	Bz	46	60:40	23/38
4	toluene	Ts	NR	-	-
5	toluene	$Ph_2P(O)$	73	85:15	2/0

a Reaction conditions: **2a** (0.10 mmol), **3** (0.11 mmol), (*M*)-**1a**·HBr (0.012 mmol), $NaN(SiMe_3)_2$ (0.010 mmol), toluene (1.0 mL), rt, 20 h. *b* The combined yield of the diastereomeric mixtures was indicated. *c* The dr and ee values were determined by chiral HPLC analysis. Boc = *tert*-butyloxycarbonyl; Alloc = allyloxycarbonyl; Bz = benzoyl; Ts = tosyl.

Table S2. Details for screening of alkoxycarbonyl groups of imines.[a]

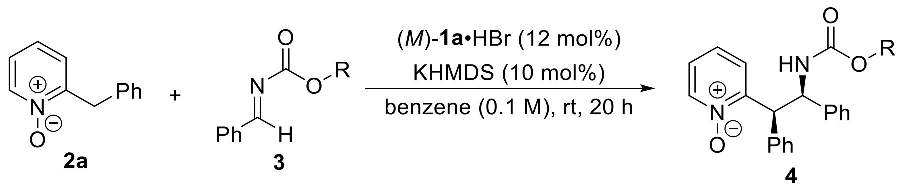

Entry	R	Yield [%][b]	*syn*/*anti*[c]	ee [%][c]
1	Me	71	59:41	87/13
2	Et	79	62:38	73/16
3	*t*Bu	83	83:17	91/10
4	$C(Et)_3$	80	83:17	89/8
5	$C(Et)(Me)_2$	57	69:31	88/27
6	Ad	63	81:19	88/11

a Reaction conditions: **2a** (0.1 mmol), **3** (0.11 mmol), (*M*)-**1a**·HBr (0.012 mmol), $KN(SiMe_3)_2$ (0.010 mmol), benzene (1.0 mL), rt, 20 h. *b* The combined yield of the diastereomeric mixtures was indicated. *c* The dr and ee values were determined by chiral HPLC analysis. Ad = adamantyl.

Table S3. Details for screening of other effects.[a]

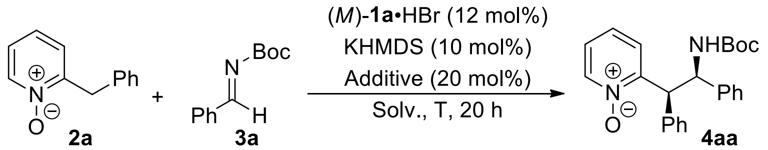

Entry	Additive	Solv.	Conc. (x M)	T [ºC]	Yield [%][b]	*syn*/*anti*[c]	ee [%][c]
1	18-crown-6	benzene	0.1	rt	84	69:31	67/0
2	KBr	benzene	0.1	rt	87	81:19	90/11
3	4Å MS	benzene	0.1	rt	83	82:18	89/12
4	-	benzene	0.25	rt	91	78:22	87/11
5	-	benzene/tol (2:3)	0.1	0	44	76:24	83/2
6	-	benzene/tol (2:3)	0.2	0	90	82:18	86/14
7	-	benzene/tol (2:3)	0.2	-10	trace		

[a] Reaction conditions: **2a** (0.1 mmol), **3a** (0.11 mmol), (*M*)-**1a**·HBr (0.012 mmol), $KN(SiMe_3)_2$ (0.010 mmol), solvent, 20 h. [b] The combined yield of the diastereomeric mixtures was indicated. [c] The dr and ee values were determined by chiral HPLC analysis.

3. Synthesis of 2-benzylpyridine and their derivatives S2-S14

3.1 Typical procedure for the substituted 2-benzylpyridine (S2-S6, S8-S13)

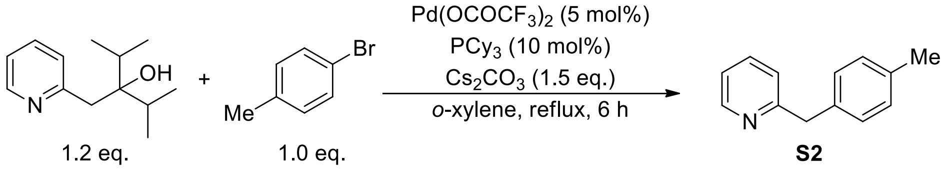

The synthesis of substituted 2-benzylpyridines (except **S7** and **S14**) was according to the method described in the literature.[1]

To a 20 mL two-necked reaction flask equipped with a condenser were added 2,4-dimethyl-3-((6-methylpyridin-2-yl)methyl)pentan-3-ol (0.62 g, 3.0 mmol), 4-bromotoluene (0.43 g, 2.5 mmol), $Pd(OCOCF_3)_2$ (42.1 mg, 0.12 mmol), tricyclohexylphosphine (70.0 mg, 0.24 mmol), Cs_2CO_3 (1.22 g, 3.8 mmol). The system was evacuated and filled with argon gas, and then *o*-xylene (6.0 mL) was added. The resulting mixture was refluxed for 6 h. After cooled to room temperature, H_2O (5.0 mL) was added, and the resulting solution was extracted with ethyl acetate (20 mL × 3). The combined organic layer was dried over sodium sulfate, and concentrated in vacuo. The residue was purified by flash chromatography (hexane/ethyl acetate = 4:1 as the eluent) to provide the desired product **S2** in 77% yield.

3.2 Synthesis of 2-(4-bromobenzyl)pyridine S7

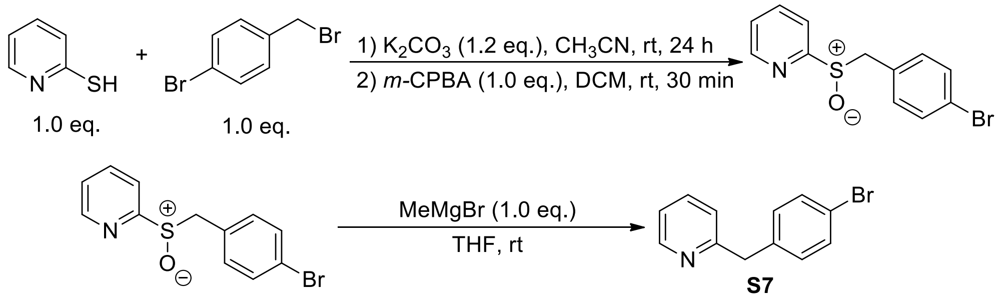

The synthesis of 2-(4-bromobenzyl)pyridine **S7** was according to the method described in the literature.[2]

To a solution of 2-mercaptopyridine (0.39 g, 3.5 mmol) in acetonitrile (35 mL) were added potassium carbonate (0.58 g, 4.2 mmol) and 4-bromobenzyl bromide (0.88 g, 3.5 mmol). After stirring for 24 h, 40 mL H_2O was added, and the mixture was extracted with CH_2Cl_2 (20 mL × 3). The combined organic layer was dried over magnesium sulfate and concentrated to give the crude sulfide. The crude sulfide was dissolved in 35 mL CH_2Cl_2. To the solution was added *m*-CPBA (77 wt%, 0.79 g, 3.5 mmol). After stirred for 30 min, 10 mL aq. NaOH (1 M) was added, the phases

were separated, and the aqueous phase was extracted with CH_2Cl_2 (10 mL × 2). The combined organic phase was dried over magnesium sulfate and concentrated in vacuo. The residue was purified by flash chromatography (hexane/ethyl acetate = 2:1 as the eluent) to afford the sulfoxide. To a solution of the sulfoxide (3.5 mmol) in 15 mL THF was added methylmagnesium bromide solution (3.0 M in THF, 3.9 mmol) dropwise at room temperature. Then the reaction mixture was stirred for 1 h. The reaction was quenched by adding 10 mL saturated aq. NH_4Cl slowly, and the mixture was extracted with ethyl acetate (20 mL × 3). The combined organic layer was dried over magnesium sulfate and concentrated in vacuo. The residue was purified by flash chromatography (hexane/ethyl acetate = 4:1 as the eluent) to provide **S7**.

3.3 Synthesis of 2-benzyl-4-methoxypyridine S14

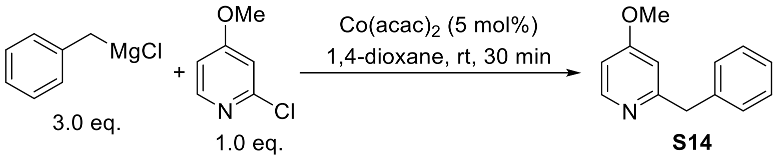

2-Benzyl-4-methoxypyridine **S14** was synthesized according to the method described in the literature.[3]

To a 100 mL two-necked flask were added anhydrous cobalt(II) acetylacetonate (85.7 mg, 0.33 mmol) and 25 mL anhydrous 1,4-dioxane under argon atmosphere. After cooled to 0 °C, benzylmagnesium chloride (1.0 M ether solution, 10 mL, 10.0 mmol) was added dropwise, then the solution was warmed to room temperature and stirred for 5 min. 2-Chloro-4-methoxypyridine (0.38 mL, 3.3 mmol) was added dropwise to the solution. After stirred for 30 min, the reaction mixture was poured into 30 mL H_2O. The mixture was extracted with ethyl acetate (20 mL × 3). The combined organic layer was dried over sodium sulfate, and concentrated in vacuo. The residue was purified by flash chromatography (hexane/ethyl acetate = 4:1 as the eluent) to provide desired product **S14**.

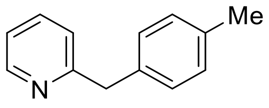

2-(4-Methylbenzyl)pyridine (S2)[2] 77% yield; ^{1}H NMR (600 MHz, $CDCl_3$) δ 8.57-8.52 (m, 1H), 7.57 (td, *J* = 7.7, 1.8 Hz, 1H), 7.17 (d, *J* = 8.0 Hz, 2H), 7.12 (d, *J* = 7.6 Hz, 2H), 7.10 (t, *J* = 3.7 Hz, 2H), 4.13 (s, 2H), 2.33 (s, 3H).

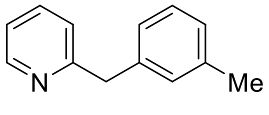

2-(3-Methylbenzyl)pyridine (S3)[2] 66% yield; ^{1}H NMR (600 MHz, $CDCl_3$) δ 8.56 (d, *J* = 4.9 Hz, 1H), 7.55 (td, *J* = 7.7, 1.8 Hz, 1H), 7.22-7.16 (m, 4H), 7.11 (dd, *J* = 7.2, 5.1 Hz, 1H), 6.96 (d, *J* = 7.8 Hz, 1H), 4.19 (s, 2H), 2.26 (s, 3H).

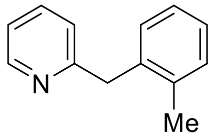

2-(2-Methylbenzyl)pyridine (S4)[1] 96% yield; ^{1}H NMR (600 MHz, $CDCl_3$) δ 8.56 (ddd, *J* = 4.8, 1.6, 0.8 Hz, 1H), 7.55 (td, *J* = 7.7, 1.8 Hz, 1H), 7.21-7.17 (m, 4H), 7.11 (ddd, *J* = 7.5, 4.7, 0.7 Hz, 1H), 6.96 (d, *J* = 7.9 Hz, 1H), 4.19 (s, 2H), 2.26 (s, 3H).

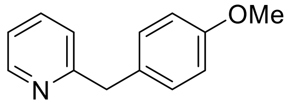

2-(4-Methoxybenzyl)pyridine (S5)[1] 78% yield; ^{1}H NMR (600 MHz, $CDCl_3$) δ 8.53 (d, *J* = 4.5 Hz, 1H), 7.55 (td, *J* = 7.6, 1.8 Hz, 1H), 7.23-7.14 (m, 2H), 7.13-7.01 (m, 2H), 6.91-6.78 (m, 2H), 4.09 (s, 2H), 3.77 (s, 3H).

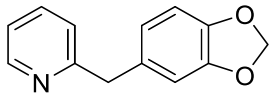

2-(Benzo[d][1,3]dioxol-5-ylmethyl)pyridine (S6)[4] 75% yield; ^{1}H NMR (600 MHz, $CDCl_3$) δ 8.52 (dd, *J* = 5.4, 1.8 Hz, 1H), 7.55 (td, *J* = 7.7, 1.9 Hz, 1H), 7.10-7.06 (m, 2H), 6.74-6.69 (m, 3H), 5.88 (s, 2H), 4.04 (s, 2H).

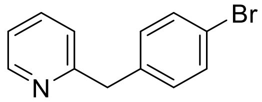

2-(4-Bromobenzyl)pyridine (S7)[2] 57% yield; ^{1}H NMR (600 MHz, $CDCl_3$) δ 8.55 (d, *J* = 4.8 Hz, 1H), 7.59 (td, *J* = 7.7, 1.8 Hz, 1H), 7.42 (d, *J* = 8.4 Hz, 2H), 7.14 (d, *J* = 8.3 Hz, 2H), 7.13 (dd, *J* = 6.3, 3.9 Hz, 1H), 7.10 (d, *J* = 7.8 Hz, 1H), 4.11 (s, 2H).

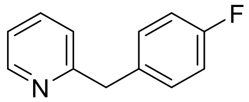

2-(4-Fluorobenzyl)pyridine (S8)[2] 67% yield; ^{1}H NMR (600 MHz, $CDCl_3$) δ 8.53 (ddd, *J* = 4.8, 1.7, 0.9 Hz, 1H), 7.55 (td, *J* = 7.7, 1.9 Hz, 1H), 7.22-7.17 (m, 2H), 7.10-7.06 (m, 2H), 6.99-6.93 (m, 2H), 4.10 (s, 2H).

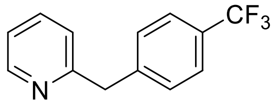

2-(4-(Trifluoromethyl)benzyl)pyridine (S9)[1] 74%yield; ^{1}H NMR (600 MHz, $CDCl_3$) δ 8.55 (ddd, *J* = 2.7, 2.0, 1.1 Hz, 1H), 7.59 (td, *J* = 7.7, 1.8 Hz, 1H), 7.54 (d, *J* = 8.1 Hz, 2H), 7.37 (d, *J* = 8.0 Hz, 2H), 7.13 (ddd, *J* = 7.7, 4.9, 0.9 Hz, 1H), 7.11 (d, *J* = 7.8 Hz, 1H), 4.19 (s, 2H).

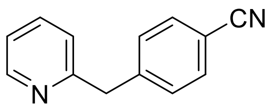

2-(4-Cyanobenzyl)pyridine (S10)[1] 89% yield; ^{1}H NMR (600 MHz, $CDCl_3$) δ 8.53 (d, *J* = 4.0 Hz, 1H), 7.60 (td, *J* = 7.6, 1.7 Hz, 1H), 7.56 (d, *J* = 8.0 Hz, 2H), 7.35 (d, *J* = 7.9 Hz, 2H), 7.20-7.04 (m, 2H), 4.17 (s, 2H).

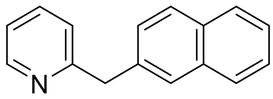

2-(Naphthalen-2-ylmethyl)pyridine (S11)[4] 91% yield; ^{1}H NMR (600 MHz, $CDCl_3$) δ 8.59-8.54 (m, 1H), 7.82-7.74 (m, 3H), 7.72 (s, 1H), 7.56 (td, *J* = 7.7, 1.8 Hz, 1H), 7.47-7.40 (m, 2H), 7.38 (dd, *J* = 8.4, 1.7 Hz, 1H), 7.13 (d, *J* = 7.9 Hz, 1H), 7.12-7.09 (m, 1H), 4.32 (s, 2H).

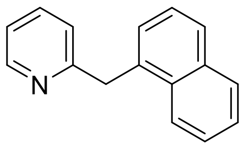

2-(Naphthalen-1-ylmethyl)pyridine (S12)[4] 81% yield; ^{1}H NMR (600 MHz, $CDCl_3$) δ 8.60 (dd, *J* = 4.9, 0.8 Hz, 1H), 8.06-8.00 (m, 1H), 7.90-7.84 (m, 1H), 7.80 (d, *J* = 8.2 Hz, 1H), 7.51-7.43 (m, 4H), 7.42 (d, *J* = 6.7 Hz, 1H), 7.10 (dd, *J* = 7.2, 5.1 Hz, 1H), 6.97 (d, *J* = 7.9 Hz, 1H), 4.65 (s, 2H).

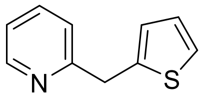

2-(Thiophen-2-ylmethyl)pyridine (S13) [5] 41% yield; ^{1}H NMR (600 MHz, CDCl3) δ 8.56 (d, *J* = 4.8 Hz, 1H), 7.61 (td, *J* = 7.7, 1.8 Hz, 1H), 7.19 (d, *J* = 7.8 Hz, 1H), 7.17 (dd, *J* = 5.1, 1.1 Hz, 1H), 7.14 (dd, *J* = 7.2, 4.8 Hz, 1H), 6.94 (dd, *J* = 5.1, 3.5 Hz, 1H), 6.90-6.88 (m, 1H), 4.34 (s, 2H).

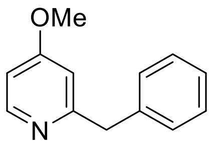

2-Benzyl-4-methoxypyridine (S14)[6] 86% yield; ^{1}H NMR (600 MHz, $CDCl_3$) δ 8.35 (t, *J* = 6.4 Hz, 1H), 7.30-7.24 (m, 4H), 7.21-7.18 (m, 1H), 6.63 (dd, *J* = 5.8, 2.5 Hz, 1H), 6.60 (d, *J* = 2.4 Hz, 1H), 4.09 (s, 2H), 3.74 (s, 3H).

4. Synthesis of 2-benzylpyridine *N*-oxide and their derivatives 2a-n

4.1 Typical procedure for the substituted 2-benzylpyridine *N*-oxides

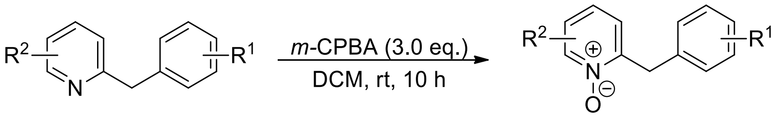

To a 50 mL flask were added 2-benzylpyridine (2.0 mmol, 1.0 eq.) and 20 mL CH_2Cl_2. The solution was cooled to 0 ºC, and *meta*-chloroperoxybenzoic acid (6.0 mmol, 3.0 eq) was added portionwise. After that, the mixture was warmed to room temperature and stirred for 6 h. The reaction mixture was quenched with potassium carbonate (1.24 g, 4.5 eq.) and stirred for additional 10 min. The mixture was filtrated through a celite pad. The filtrate was concentrated under reduced pressure. The residue was purified by flash chromatography (hexane/ethyl acetate/methanol = 3:6:1 as the eluent) to afford the desired product.

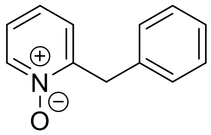

2-Benzylpyridine *N*-oxide (2a)[7] 60% yield; ^{1}H NMR (600 MHz, $CDCl_3$) δ 8.38-8.23 (m, 1H), 7.36 (t, *J* = 7.6 Hz, 2H), 7.29 (t, *J* = 8.6 Hz, 3H), 7.21-7.10 (m, 2H), 7.00-6.90 (m, 1H), 4.27 (s, 2H); ^{13}C NMR (150 MHz, $CDCl_3$) δ 152.1, 139.5, 136.4, 129.8, 128.9, 127.1, 125.9, 125.5, 123.6, 36.6.

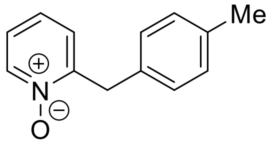

2-(4-Methylbenzyl)pyridine *N*-oxide (2b)[7] 81% yield; ^{1}H NMR (600 MHz, $CDCl_3$) δ 8.33-8.24 (m, 1H), 7.17 (s, 4H), 7.16-7.12 (m, 2H), 6.96-6.92 (m, 1H), 4.23 (s, 2H), 2.36 (s, 3H); ^{13}C NMR (150 MHz, $CDCl_3$) δ 152.2, 139.3, 136.6, 133.1, 129.6, 129.5, 125.7, 125.4, 123.4, 36.1, 21.1.

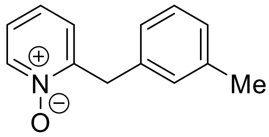

2-(3-Methylbenzyl)pyridine *N*-oxide (2c)[8] 75% yield; ^{1}H NMR (600 MHz, $CDCl_3$) δ 8.29 (dd, *J* = 5.3, 2.4 Hz, 1H), 7.25 (t, *J* = 7.5 Hz, 1H), 7.19-7.13 (m, 2H), 7.13-7.06 (m, 3H), 6.98-6.92 (m, 1H), 4.23 (s, 2H), 2.35 (s, 3H), ^{13}C NMR (150 MHz, $CDCl_3$) δ 152.1, 139.3, 138.5, 136.1, 130.4, 128.7, 127.8, 126.7, 125.8, 125.4, 123.4, 36.4, 21.4.

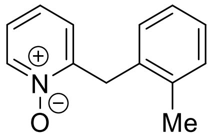

2-(2-Methylbenzyl)pyridine *N*-oxide (2d)[7] 84% yield; ^{1}H NMR (600 MHz, $CDCl_3$) δ 8.33 (dd, *J* = 6.5, 0.9 Hz, 1H), 7.26-7.19 (m, 3H), 7.19-7.14 (m, 2H), 7.11 (td, *J* = 7.8, 1.1 Hz, 1H), 6.71 (dd, *J* = 7.8, 1.4 Hz, 1H), 4.25 (s, 2H), 2.20 (s, 3H); ^{13}C NMR (150 MHz, $CDCl_3$) δ 151.2, 139.2, 137.1, 134.3, 130.51, 130.49, 127.4, 126.4, 125.5, 125.0, 123.3, 34.2, 19.2.

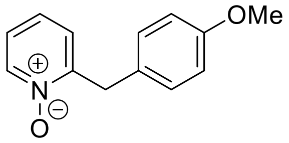

2-(4-Methoxybenzyl)pyridine *N*-oxide (2e)[7] 72% yield; ^{1}H NMR (600 MHz, $CDCl_3$) δ 8.31-8.27 (m, 1H), 7.21 (d, *J* = 8.5 Hz, 2H), 7.17-7.10 (m, 2H), 6.98-6.93 (m, 1H), 6.92-6.87 (m, 2H), 4.21 (s, 2H), 3.82 (s, 3H); ^{13}C NMR (150 MHz, $CDCl_3$) δ 158.7, 152.4, 139.4, 130.8, 128.1, 125.7, 125.4, 123.4, 114.3, 55.3, 35.7.

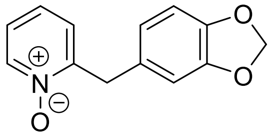

2-(Benzo[*d*][1,3]dioxol-5-ylmethyl)pyridine *N*-oxide (2f) 73% yield; ^{1}H NMR (600 MHz, $CDCl_3$) δ 8.30-8.27 (m, 1H), 7.17-7.13 (m, 2H), 7.01-6.97 (m, 1H), 6.80 (d, *J* = 7.8 Hz, 1H), 6.78-6.73 (m, 2H), 5.96 (s, 2H), 4.18 (s, 2H); ^{13}C NMR (150 MHz, $CDCl_3$) δ 152.0, 148.0, 146.6, 139.4, 129.8, 125.7, 125.4, 123.5, 122.8, 110.0, 108.6, 101.0, 36.2; IR (ATR): 3398, 2888, 1504, 1489, 1442, 1250, 1037 cm^{-1}; HRMS (ESI) m/z calcd for $C_{13}H_{11}NNaO_3$ $[M+Na]^+$ 252.0631, found: 252.0631.

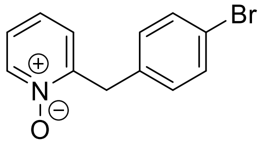

2-(4-Bromobenzyl)pyridine *N*-oxide (2g) 81% yield; ^{1}H NMR (600 MHz, $CDCl_3$) δ 8.31-8.25 (m, 1H), 7.51-7.44 (m, 2H), 7.20-7.13 (m, 4H), 7.01-6.96 (m, 1H), 4.22 (s, 2H); ^{13}C NMR (150 MHz, $CDCl_3$) δ 151.2, 139.5, 135.3, 131.9, 131., 125.8, 125.4, 123.8, 121.0, 36.0; IR (ATR): 3410, 3082, 1487, 1435, 1407, 1246, 1070, 1012, 913, 862 cm^{-1}; HRMS (ESI) m/z calcd for $C_{12}H_{10}BrNNaO$ $[M+Na]^+$ 285.9838, found: 285.9838.

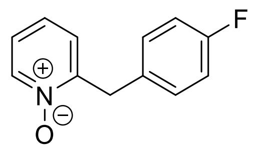

2-(4-Fluorobenzyl)pyridine *N*-oxide (2h)[7] 65% yield; ^{1}H NMR (600 MHz, $CDCl_3$) δ 8.31-8.27 (m, 1H), 7.27 (m, 3H), 7.19-7.14 (m, 2H), 7.08-7.01 (m, 2H), 7.00-6.95 (m, 1H), 4.24 (s, 2H); ^{13}C NMR (150 MHz, CDCl3) δ 162.0 (d, $J_{C\text{-}F}$ = 245.5 Hz)., 151.7, 139.5, 132.0 (d, $J_{C\text{-}F}$ = 2.6 Hz), 131.1 (d, $J_{C\text{-}F}$ = 8.2 Hz), 125.7, 125.4, 123.7, 115.7 (d, $J_{C\text{-}F}$ = 21.6 Hz), 35.8.

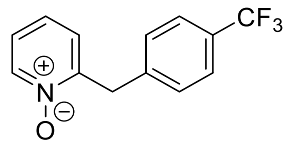

2-(4-(Trifluoromethyl)benzyl)pyridine *N*-oxide (2i)[7] 67% yield; ^{1}H NMR (600 MHz, $CDCl_3$) δ 8.32-8.27 (m, 1H), 7.60 (d, J = 8.1 Hz, 2H), 7.42 (d, J = 8.0 Hz, 2H), 7.22-7.16 (m, 2H), 7.06-7.01 (m, 1H), 4.32 (s, 2H); ^{13}C NMR (150 MHz, $CDCl_3$) δ 150.7, 140.5, 139.6, 129.8, 129.4 (q, $J_{C\text{-}F}$ = 32.2 Hz), 125.9, 125.7 (q, $J_{C\text{-}F}$ = 3.7 Hz), 125.5, 124.1 (q, $J_{C\text{-}F}$ = 270.5 Hz), 124.1, 36.4.

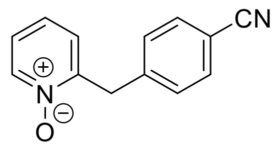

2-(4-Cyanobenzyl)pyridine *N*-oxide (2j)[7] 78% yield; ^{1}H NMR (600 MHz, $CDCl_3$) δ 8.27 (dd, J = 4.9, 2.8 Hz, 1H), 7.62 (d, J = 8.2 Hz, 2H), 7.42 (d, J = 8.1 Hz, 2H), 7.23-7.18 (m, 2H), 7.11-7.07 (m, 1H), 4.31 (s, 2H); ^{13}C NMR (150 MHz, $CDCl_3$) δ 149.9, 142.0, 139.7, 132.4, 130.1, 126.0, 125.5, 124.3, 118.7, 110.9, 36.7.

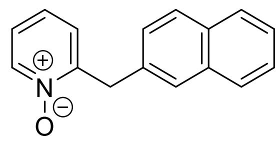

2-(Naphthalen-2-ylmethyl)pyridine *N*-oxide (2k)[9] 82% yield; ^{1}H NMR (600 MHz, $CDCl_3$) δ 8.32 (dd, J = 6.4, 1.0 Hz, 1H), 7.87-7.83 (m, 2H), 7.81 (dd, J = 6.3, 2.9 Hz, 1H), 7.76 (s, 1H), 7.52-7.45 (m, 2H), 7.39 (dd, J = 8.4, 1.7 Hz, 1H), 7.18-7.15 (m, 1H), 7.12 (td, J = 7.6, 1.3 Hz, 1H), 6.96 (dd, J = 7.7, 1.8 Hz, 1H), 4.44 (s, 2H); ^{13}C NMR (150 MHz, $CDCl_3$) δ 151.9, 139.4, 133.8, 133.6, 132.5, 128.6, 128.4, 127.8, 127.7, 127.6, 126.2, 125.9, 125.8, 125.5, 123.6, 36.6.

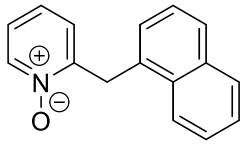

2-(Naphthalen-1-ylmethyl)pyridine *N*-oxide (2l)[7] 79% yield; ^{1}H NMR (600 MHz, $CDCl_3$) δ 8.38 (dd, *J* = 6.4, 0.7 Hz, 1H), 7.91 (d, *J* = 8.1 Hz, 1H), 7.86 (d, *J* = 8.2 Hz, 1H), 7.77 (d, *J* = 8.5 Hz, 1H), 7.52-7.43 (m, 4H), 7.14 (td, *J* = 7.5, 2.0 Hz, 1H), 7.00 (td, *J* = 8.1, 1.2 Hz, 1H), 6.63 (dd, *J* = 8.0, 1.3 Hz, 1H), 4.72 (s, 2H); ^{13}C NMR (150 MHz, $CDCl_3$) δ 151.5, 139.2, 134.0, 132.4, 132.1, 128.8, 128.5, 128.2, 126.5, 126.0, 125.7, 125.7, 125.5, 124.0, 124.0, 33.8.

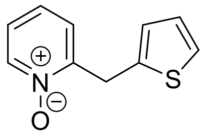

2-(Thiophen-2-ylmethyl)pyridine *N*-oxide (2m) 68% yield; ^{1}H NMR (600 MHz, $CDCl_3$) δ 8.32-8.25 (m, 1H), 7.24 (dd, *J* = 4.9, 1.4 Hz, 1H), 7.19-7.15 (m, 2H), 7.13-7.09 (m, 1H), 7.02-6.97 (m, 2H), 4.47 (s, 2H); ^{13}C NMR (150 MHz, $CDCl_3$) δ 150.9, 139.2, 137.6, 127.3, 127.1, 125.5, 125.4, 125.1, 123.8, 30.6; IR (ATR): 3388, 3076, 1489, 1436, 1240, 848, 768, 701 cm^{-1}; HRMS (ESI) m/z calcd for $C_{10}H_9NNaOS$ $[M+Na]^+$ 214.0297, found: 214.0297.

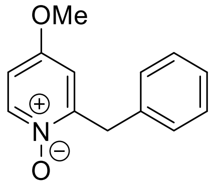

2-Benzyl-4-methoxypyridine *N*-oxide (2n) 79% yield; ^{1}H NMR (600 MHz, $CDCl_3$) δ 8.21 (d, *J* = 7.2 Hz, 1H), 7.40-7.34 (m, 2H), 7.32-7.28 (m, 3H), 6.70 (dd, *J* = 7.2, 3.5 Hz, 1H), 6.43 (d, *J* = 3.4 Hz, 1H), 4.27 (s, 2H), 3.73 (s, 3H); ^{13}C NMR (150 MHz, $CDCl_3$) δ 157.4, 152.8, 140.0, 136.2, 129.7, 128.9, 127.1, 111.3, 109.4, 55.8, 36.8; IR (ATR): 3379, 3062, 1632, 1560, 1485, 1467, 1433, 1302, 1214, 1160, 1032 cm^{-1}; HRMS (ESI) m/z calcd for $C_{13}H_{13}NNaO_2$ $[M+Na]^+$ 238.0839, found: 238.0838.

5. Catalytic enantioselective direct Mannich-type reactions

5.1 Typical procedure

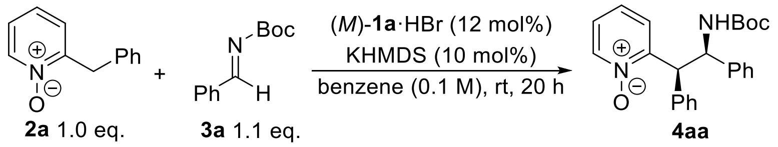

To a dried test tube was added (*M*)-**1a**·HBr (9.5 mg, 0.012 mmol), and then anhydrous benzene (1.0 mL) was added under argon atmosphere. A solution of $KN(SiMe_3)_2$ in toluene (0.5 M, 20 µL, 0.010 mmol) was added to the suspension at room temperature. After stirring for ca. 1 min, 2-benzylpyridine *N*-oxide **2a** was added in one portion, and the mixture was stirred for 1 min. Then *N*-Boc imine **3a** was added to the solution. After that, the reaction mixture was stirred at room temperature for 20 h. The reaction was quenched with 1.0 mL saturated NH_4Cl aq. and extracted with CH_2Cl_2 (5.0 mL × 3). The combined organic phase was dried over sodium sulfate, and concentrated in vacuo. The residue was purified by flash chromatography (hexane/ethyl acetate/methanol = 3:9:1 as the eluent) to afford **4aa** in 83% yield with *syn*/*anti* = 83/17 and 91% ee. The diastereomers of **4aa** were reslurried in a mixed solvent of ethyl acetate 2.0 mL and hexane 1.0 mL for 2 h. After filtration, the major *syn*-isomer of **4aa** was obtained as a white solid in 54% yield with 93% ee.

All the characterization data of the mixture of diastereomers were described in this section. In the case of **4aa**, the NMR spectra of two diastereomers were given respectively.

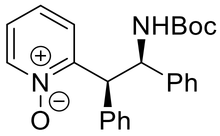

2-((1*R*,2*R*)-2-((*tert*-Butoxycarbonyl)amino)-1,2-diphenylethyl)pyridine *N*-oxide (4aa) 83% yield, 83/17 dr, 91% ee; **Major isomer**: ^{1}H NMR (600 MHz, $CDCl_3$) δ 8.08 (d, *J* = 6.3 Hz, 1H), 7.45-7.36 (m, 3H), 7.34-7.27 (m, 4H), 7.25-7.21 (m, 3H), 7.19-7.15 (m, 1H), 7.09 (t, *J* = 7.6 Hz, 1H), 6.99 (t, *J* = 6.4 Hz, 1H), 5.61 (brs, 2H), 5.08 (brs, 1H), 1.28 (s, 9H); ^{13}C NMR (151 MHz, $CDCl_3$) δ 154.9, 152.1, 140.7, 139.5, 137.5, 129.0, 128.5, 127.6, 127.4, 126.9, 125.2, 125.1, 123.5, 79.6, 56.1, 48.1, 28.2; **Minor isomer**: ^{1}H NMR (600 MHz, $CDCl_3$) δ 8.29 (d, *J* = 6.4 Hz, 1H), 7.48 (d, *J* = 7.9 Hz, 1H), 7.34 (d, *J* = 7.6 Hz, 2H), 7.28 (d, *J* = 7.6 Hz, 2H), 7.23-7.10 (m, 9H), 6.81 (d, *J* = 9.1 Hz, 1H), 5.63 (d, *J* = 11.5 Hz, 1H), 5.45 (t, *J* = 10.5 Hz, 1H), 1.26 (s, 9H); ^{13}C NMR (151 MHz, $CDCl_3$) δ 155.5, 152.9, 141.3, 139.5, 137.6, 129.4, 128.6, 128.3, 127.4, 127.3, 127.3, 126.1, 1266.0, 123.6, 79.1, 58.2, 50.0, 28.3; IR (ATR): 3227, 2978, 1698, 1491, 1237, 1169, 749 cm^{-1}; HRMS (ESI) m/z calcd for $C_{24}H_{26}N_2NaO_3$ $[M+Na]^+$ 413.1836, found: 413.1836; HPLC conditions: DAICEL Chiralpak IA-3 column, hexane/*i*-PrOH = 70/30, 254 nm, 1.0 mL/min, 40 °C, t_{major} = 5.81 min, t_{minor} = 7.88 min; $[\alpha]^{27}_D$ = 52.7 (c = 0.67, CH_3OH).

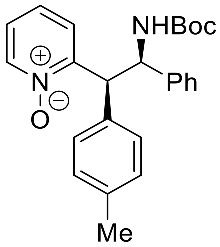

2-((1*R*,2*R*)-2-((*tert*-Butoxycarbonyl)amino)-2-phenyl-1-(*p*-tolyl)ethyl)pyridine *N*-oxide (4ba) 81% yield, 77/23 dr, 88% ee; **Major isomer**: ^{1}H NMR (600 MHz, $CDCl_3$) δ 8.06 (dd, *J* = 6.5, 0.8 Hz, 1H), 7.41-6.93 (m, 12H), 5.57 (brs, 2H), 5.11 (brs, 1H), 2.28 (s, 3H), 1.27 (s, 9H); ^{13}C NMR (150 MHz, $CDCl_3$) (representative peaks) δ 154.9, 152.2, 79.6, 56.2, 47.9, 28.1, 21.4; **Minor isomer**: ^{1}H NMR (600 MHz, $CDCl_3$) (representative peaks) δ 8.27 (d, *J* = 6.4 Hz, 1H), 6.63 (dd, *J* = 9.2, 4.6 Hz, 1H), 5.60 (d, *J* = 11.4 Hz, 1H), 5.45 (dd, *J* = 11.1, 9.9 Hz, 1H), 2.21 (s, 3H), 1.23 (s, 9H); ^{13}C NMR (150 MHz, $CDCl_3$) (representative peaks) δ 155.5, 153.1, 79.0, 58.2, 49.6, 28.2, 21.3; IR (ATR): 3230, 2976, 1698, 1365, 1294, 1229, 1169, 858 cm^{-1}; HRMS (ESI) m/z calcd for $C_{25}H_{28}N_2NaO_3$ $[M+Na]^+$ 427.1992, found: 427.1992; HPLC conditions: DAICEL Chiralpak IE-3 column, hexane/*i*-PrOH = 70/30, 254 nm, 1.0 mL/min, 40 °C, t_{major} = 24.1 min, t_{minor} = 25.8 min; $[\alpha]^{27}_{D}$ = 46.4 (c = 0.49, CH_3OH).

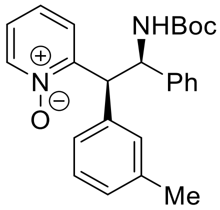

2-((1*R*,2*R*)-2-((*tert*-Butoxycarbonyl)amino)-2-phenyl-1-(*m*-tolyl)ethyl)pyridine *N*-oxide (4ca) 83% yield, 79/21 dr, 92% ee; **Major isomer**: ^{1}H NMR (600 MHz, $CDCl_3$) δ 8.06 (dd, *J* = 6.4, 0.7 Hz, 1H), 7.41-6.93 (m, 12H), 5.56 (brs, 2H), 5.08 (brs, 1H), 2.28 (s, 3H), 1.27 (s, 9H); ^{13}C NMR (150 MHz, $CDCl_3$) (representative peaks) δ 154.9, 152.2, 79.6, 56.1, 47.9, 28.2, 21.4; **Minor isomer**: ^{1}H NMR (600 MHz, $CDCl_3$) (representative peaks) δ 8.28 (d, *J* = 6.4 Hz, 1H), 6.60 (d, *J* = 9.2 Hz, 1H), 5.60 (t, *J* = 11.4 Hz, 1H), 5.45 (dd, *J* = 11.1, 9.8 Hz, 1H), 2.21 (s, 3H), 1.23 (s, 9H); ^{13}C NMR (150 MHz, $CDCl_3$) (representative peaks) δ 155.5, 153.1, 79.0, 58.2, 49.6, 28.2, 21.3; IR (ATR): 3337, 2976, 1700, 1364, 1250, 1017, 753, 700 cm^{-1}; HRMS (ESI) m/z calcd for $C_{25}H_{28}N_2NaO_3$ $[M+Na]^+$ 427.1992, found: 427.1992; HPLC conditions: DAICEL Chiralpak AD-3 column, hexane/*i*-PrOH = 70/30, 254 nm, 1.0 mL/min, 40 °C, t_{major} = 6.08 min, t_{minor} = 8.10 min; $[\alpha]^{25}_{D}$ = 40.0 (c = 0.25, CH_3OH).

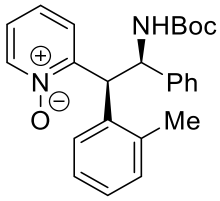

2-((1*R*,2*R*)-2-((*tert*-Butoxycarbonyl)amino)-2-phenyl-1-(*o*-tolyl)ethyl)pyridine *N*-oxide (4da) 42% yield, 83/17 dr, 84% ee; **Major isomer**: ^{1}H NMR (600 MHz, $CDCl_3$) δ 8.04 (d, *J* = 6.3 Hz, 1H), 7.43 (d, *J* = 7.9 Hz, 1H), 7.36-6.95 (m, 11H), 5.57 (brs, 1H), 5.51 (brs, 1H), 5.19 (brs, 1H), 2.20 (s, 3H), 1.28 (s, 9H); ^{13}C NMR (150 MHz, $CDCl_3$) (representative peaks) δ 154.8, 152.6, 79.7, 56.6, 44.6, 28.2, 19.8; **Minor isomer**: ^{1}H NMR (600 MHz, $CDCl_3$) (representative peaks) δ 8.30 (d, *J* = 6.4 Hz, 1H), 7.55 (d, *J* = 7.5 Hz, 1H), 6.61 (dd, *J* = 18.5, 8.5 Hz, 1H), 5.88 (dd, *J* = 11.6, 2.8 Hz, 1H), 5.48 (t, *J* = 10.4 Hz, 1H), 2.03 (s, 3H), 1.23 (s, 9H); ^{13}C NMR (150 MHz, $CDCl_3$) (representative peaks) δ 155.5, 153.1, 79.1, 58.5, 44.6, 28.3, 19.6; IR (ATR): 3210, 2975, 2928, 1703, 1491, 1433, 1365, 1236, 1170, 1011, 791, 752, 701 cm^{-1}; HRMS (ESI) m/z calcd for $C_{25}H_{28}N_2NaO_3$ $[M+Na]^+$ 427.1992, found: 427.1992; HPLC conditions: DAICEL Chiralpak IA-3 column, hexane/*i*-PrOH = 70/30, 254 nm, 1.0 mL/min, 40 °C, t_{major} = 5.18 min, t_{minor} = 7.00 min; $[\alpha]^{26}_D$ = 50.9 (c = 0.47, CH_3OH).

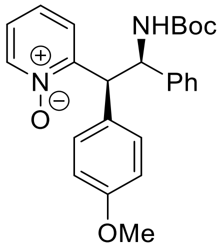

2-((1*R*,2*R*)-2-((*tert*-Butoxycarbonyl)amino)-1-(4-methoxyphenyl)-2-phenylethyl)pyridine *N*-oxide (4ea) 81% yield, 80/20 dr, 90% ee; **Major isomer**: ^{1}H NMR (600 MHz, $CDCl_3$) δ 8.07 (d, *J* = 6.2 Hz, 1H), 7.50-6.90 (m, 10H), 6.81 (d, *J* = 7.3 Hz, 2H), 5.53 (brs, 2H), 5.08 (brs, 1H), 3.74 (s, 3H), 1.28 (s, 9H); ^{13}C NMR (150 MHz, $CDCl_3$) (representative peaks) δ 158.8, 155.0, 79.6, 56.1, 55.2, 47.4, 28.2; **Minor isomer**: ^{1}H NMR (600 MHz, $CDCl_3$) (representative peaks) δ 8.27 (t, *J* = 5.5 Hz, 1H), 5.55 (d, *J* = 11.4 Hz, 1H), 5.40 (t, *J* = 10.5 Hz, 1H), 3.70 (s, 3H), 1.23 (s, 9H); ^{13}C NMR (150 MHz, $CDCl_3$) (representative peaks) δ 158.6, 155.5, 79.1, 58.5, 55.1, 49.0, 28.2; IR (ATR): 3230, 2976, 1698, 1609, 1510, 1432, 1365, 1251, 1171, 1035, 1015 cm^{-1}; HRMS (ESI) m/z calcd for $C_{25}H_{28}N_2NaO_4$ $[M+Na]^+$ 443.1941, found: 443.1941; HPLC conditions: DAICEL Chiralpak AD-3 column, hexane/*i*-PrOH = 70/30, 254 nm, 1.0 mL/min, 40 °C, t_{major} = 6.76 min, t_{minor} = 10.48 min; $[\alpha]^{26}_D$ = 39.9 (c = 0.50, CH_3OH).

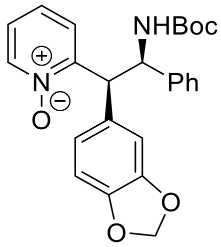

2-((1*R*,2*R*)-1-(Benzo[*d*][1,3]dioxol-5-yl)-2-((*tert*-butoxycarbonyl)amino)-2-phenylethyl)pyridine *N*-oxide (4fa) 77% yield, 76/24 dr, 90% ee; **Major isomer**: ^{1}H NMR (600 MHz, $CDCl_3$) δ 8.07 (d, *J* = 6.5 Hz, 1H), 7.86-6.40 (m, 11H), 5.88 (d, *J* = 1.7 Hz, 2H), 5.48 (brs, 2H), 5.13 (brs, 1H), 1.29 (s, 9H); ^{13}C NMR (150 MHz, $CDCl_3$) (representative peaks) δ 154.9, 152.1, 79.7, 56.1, 47.8, 28.2; **Minor isomer**: ^{1}H NMR (600 MHz, $CDCl_3$) (representative peaks) δ 8.26 (d, *J* = 6.4 Hz, 1H), 5.84 (d, *J* = 4.8 Hz, 2H), 5.53 (d, *J* = 12.0 Hz, 1H), 5.35 (t, *J* = 10.2 Hz, 1H), 1.23 (s, 9H); ^{13}C NMR (150 MHz, $CDCl_3$) (representative peaks) δ 154.4, 153.0, 79.1, 58.2, 49.5, 28.2; IR (ATR): 3224, 2976, 1694, 1488, 1431, 1365, 1292, 1246, 1168, 1039, 931 cm^{-1}; HRMS (ESI) m/z calcd for $C_{25}H_{26}N_2NaO_5$ $[M+Na]^+$ 457.1734, found: 457.1734; HPLC conditions: DAICEL Chiralpak AD-3 column, hexane/*i*-PrOH = 70/30, 254 nm, 1.0 mL/min, 40 °C, t_{major} = 10.55 min, t_{minor} = 18.00 min; $[\alpha]^{25}_{D}$ = 34.6 (c = 0.56, CH_3OH).

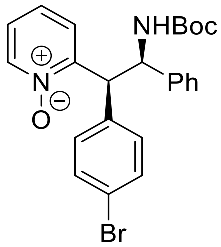

2-((1*R*,2*R*)-1-(4-Bromophenyl)-2-((*tert*-butoxycarbonyl)amino)-2-phenylethyl)pyridine *N*-oxide (4ga) 92% yield, 84/16 dr, 93% ee; **Major isomer**: ^{1}H NMR (600 MHz, $CDCl_3$) δ 8.07 (dd, *J* = 6.5, 0.9 Hz, 1H), 7.45-7.06 (m, 11H), 7.00 (t, *J* = 6.2 Hz, 1H), 5.58 (brs, 1H), 5.52 (brs, 1H), 4.99 (d, *J* = 9.2 Hz, 1H), 1.28 (s, 9H); ^{13}C NMR (150 MHz, $CDCl_3$) (representative peaks) δ 154.8, 151.5, 79.9, 55.7, 47.8, 28.2; **Minor isomer**: ^{1}H NMR (600 MHz, $CDCl_3$) (representative peaks) δ 8.26 (d, *J* = 6.4 Hz, 1H), 6.42 (dd, *J* = 33.7, 9.3 Hz, 1H), 5.56 (d, *J* = 11.4 Hz, 1H), 5.40 (t, *J* = 10.8 Hz, 1H), 1.24 (s, 9H); ^{13}C NMR (150 MHz, $CDCl_3$) (representative peaks) δ 155.4, 152.2, 79.3, 57.9, 49.4, 28.2; IR (ATR): 3237, 2980, 1697, 1542, 1490, 1276, 1259, 1169, 1012, 888 cm^{-1}; HRMS (ESI) m/z calcd for $C_{24}H_{25}BrN_2NaO_3$ $[M+Na]^+$ 491.0941, found: 491.0941; HPLC conditions: DAICEL Chiralpak AD-3 column, hexane/*i*-PrOH = 70/30, 254 nm, 1.0 mL/min, 40 °C, t_{major} = 6.58 min, t_{minor} = 9.39 min; $[\alpha]^{26}_{D}$ = 42.9 (c = 0.57, CH_3OH).

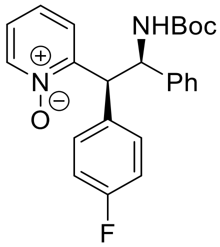

2-((1*R*,2*R*)-2-((*tert*-Butoxycarbonyl)amino)-1-(4-fluorophenyl)-2-phenylethyl)pyridine *N*-oxide (4ha) 92% yield, 85/15 dr, 93% ee; **Major isomer**: ^{1}H NMR (600 MHz, $CDCl_3$) δ 8.07 (dd, *J* = 6.4, 0.9 Hz, 1H), 7.44-6.93 (m, 12H), 5.56 (brs, 2H), 5.07 (d, *J* = 8.8 Hz, 1H), 1.27 (s, 9H); ^{13}C NMR (150 MHz, $CDCl_3$) (representative peaks) δ 163.0, 161.3, 130.8 (d, J_{C-F} = 7.7 Hz), 115.40 (d, J_{C-F} = 21.5 Hz), 79.9, 56.1, 47.6, 28.3; **Minor isomer**: ^{1}H NMR (600 MHz, $CDCl_3$) (representative peaks) δ 8.25 (dd, *J* = 6.4, 1.6 Hz, 1H), 5.40 (t, *J* = 10.2 Hz, 1H), 1.24 (s, 9H); ^{13}C NMR (150 MHz, $CDCl_3$) (representative peaks) δ 162.7, 161.1, 131.0 (d, J_{C-F} = 7.9 Hz), 79.3, 58.2, 49.4, 28.4; IR (ATR): 3262, 2991, 1698, 1685, 1541, 1520, 1472, 1276, 1260, 1168 cm^{-1}; HRMS (ESI) m/z calcd for $C_{24}H_{25}FN_2NaO_3$ $[M+Na]^+$ 431.1741, found: 431.1741; HPLC conditions: DAICEL Chiralpak AD-3 column, hexane/*i*-PrOH = 70/30, 254 nm, 1.0 mL/min, 40 °C, t_{major} = 5.49 min, t_{minor} = 6.91 min; $[\alpha]^{27}_D$ = 72.0 (c = 0.17, CH_3OH).

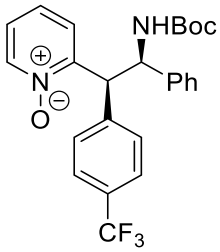

2-((1*R*,2*R*)-2-((*tert*-Butoxycarbonyl)amino)-2-phenyl-1-(4-(trifluoromethyl)phenyl)ethyl)pyridine *N*-oxide (4ia) 96% yield, 84/16 dr, 93% ee; **Major isomer**: ^{1}H NMR (600 MHz, $CDCl_3$) δ 8.08 (dd, *J* = 6.4, 0.6 Hz, 1H), 7.62-7.08 (m, 11H), 7.01 (t, *J* = 7.0 Hz, 1H), 5.64 (brs, 2H), 5.01 (d, *J* = 9.3 Hz, 1H), 1.24 (s, 9H); ^{13}C NMR (150 MHz, $CDCl_3$) (representative peaks) δ 154.9, 151.3, 124.2 (q, J_{C-F} = 271.8 Hz), 80.0, 55.8, 48.3, 28.2; **Minor isomer**: ^{1}H NMR (600 MHz, $CDCl_3$) (representative peaks) δ 8.26 (d, *J* = 6.5 Hz, 1H), 6.44 (dd, *J* = 59.9, 11.3 Hz, 1H), 5.47 (*t*, *J* = 10.6 Hz, 1H), 1.25 (s, 9H); ^{13}C NMR (150 MHz, $CDCl_3$) (representative peaks) δ 155.5, 152.0, 79.5, 57.8, 50.0, 28.3; IR (ATR): 3229, 2978, 1697, 1540, 1508, 1431, 1366, 1326, 1240, 1165, 1123, 1069, 1018 cm^{-1}; HRMS (ESI) m/z calcd for $C_{25}H_{25}F_3N_2NaO_3$ $[M+Na]^+$ 481.1710, found: 481.1709; HPLC conditions: DAICEL Chiralpak AD-3 column, hexane/*i*-PrOH = 80/20, 254 nm, 0.8 mL/min, 40 °C, t_{major} = 8.88 min, t_{minor} = 12.52 min; $[\alpha]^{27}_D$ = 58.4 (c = 0.53, CH_3OH).

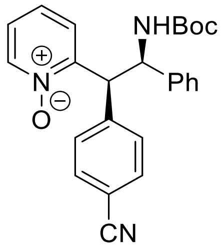

2-((1*R*,2*R*)-2-((*tert*-Butoxycarbonyl)amino)-1-(4-cyanophenyl)-2-phenylethyl)pyridine *N*-oxide (4ja) 98% yield, 91/9 dr, 67% ee; **Major isomer**: ^{1}H NMR (600 MHz, $CDCl_3$) δ 8.08 (d, *J* = 6.5 Hz, 1H), 7.64-6.99 (m, 12H), 5.63 (brs, 2H), 4.93 (d, *J* = 9.5 Hz, 1H), 1.26 (s, 9H); ^{13}C NMR (150 MHz, $CDCl_3$) (representative peaks) δ 154.8, 150.8, 80.2, 55.6, 48.6, 28.2; **Minor isomer**: ^{1}H NMR (600 MHz, $CDCl_3$) (representative peaks) δ 8.25 (d, *J* = 6.1 Hz, 1H), 6.23 (dd, *J* = 31.2, 6.3 Hz, 1H), 5.57 (d, *J* = 12.0 Hz, 1H), 4.97 (d, *J* = 9.4 Hz, 1H), 1.26 (s, 3H); ^{13}C NMR (150 MHz, $CDCl_3$) (representative peaks) δ 28.3; IR (ATR): 3263, 2977, 1698, 1522, 1507, 1456, 1433, 1366, 1251, 1168, 1015 cm^{-1}; HRMS (ESI) m/z calcd for $C_{25}H_{25}N_3NaO_3$ $[M+Na]^+$ 438.1788, found: 438.1788; HPLC conditions: DAICEL Chiralpak OD-3 column, hexane/*i*-PrOH = 80/20, 254 nm, 1.0 mL/min, 40 °C, t_{major} = 5.55 min, t_{minor} = 6.79 min; $[\alpha]^{27}_{D}$ = 20.7 (c = 0.30, CH_3OH).

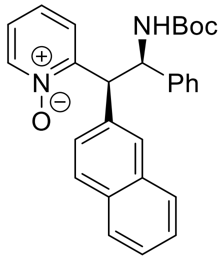

2-((1*R*,2*R*)-2-((*tert*-Butoxycarbonyl)amino)-1-(naphthalen-2-yl)-2-phenylethyl)pyridine *N*-oxide (4ka) 93% yield, 82/18 dr, 89% ee; **Major isomer**: ^{1}H NMR (600 MHz, $CDCl_3$) δ 8.07 (d, *J* = 6.4 Hz, 1H), 7.95-7.05 (m, 14H), 6.96 (t, *J* = 6.2 Hz, 1H), 5.81 (brs, 1H), 5.71 (brs, 1H), 5.21 (d, *J* = 8.7 Hz, 1H), 1.20 (s, 9H); ^{13}C NMR (150 MHz, $CDCl_3$) (representative peaks) δ 155.1, 152.2, 79.8, 56.1, 48.2, 28.2; **Minor isomer**: ^{1}H NMR (600 MHz, $CDCl_3$) (representative peaks) δ 8.35-8.20 (m, 1H), 6.71 (dd, *J* = 28.3, 9.4 Hz, 1H), 5.85 (d, *J* = 11.4 Hz, 2H), 5.62 (dd, *J* = 11.1, 9.9 Hz, 1H), 1.25 (s, 9H); ^{13}C NMR (150 MHz, $CDCl_3$) (representative peaks) δ 155.7, 153.1, 79.2, 58.3, 49.8, 28.4; IR (ATR): 3254, 2976, 1698, 1541, 1508, 1489, 1432, 1365, 1252, 1233, 1169, 1015 cm^{-1}; HRMS (ESI) m/z calcd for $C_{28}H_{28}N_2NaO_3$ $[M+Na]^+$ 463.1992, found: 463.1992; HPLC conditions: DAICEL Chiralpak AD-3 column, hexane/*i*-PrOH = 70/30, 254 nm, 1.0 mL/min, 40 °C, t_{major} = 9.06 min, t_{minor} = 14.25 min; $[\alpha]^{27}_{D}$ = 22.1 (c = 0.48, CH_3OH).

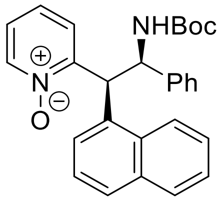

2-((1*R*,2*R*)-2-((*tert*-Butoxycarbonyl)amino)-1-(naphthalen-1-yl)-2-phenylethyl)pyridine *N*-oxide (4la) 52% yield, 84/16 dr, 80% ee; **Major isomer**: ^{1}H NMR (600 MHz, $CDCl_3$) δ 8.37 (d, *J* = 5.6 Hz, 1H), 7.94 (d, *J* = 6.4 Hz, 1H), 7.79 (dd, *J* = 17.2, 8.0 Hz, 3H), 7.57-7.36 (m, 3H), 7.30 (d, *J* = 6.6 Hz, 2H), 7.26 (dd, *J* = 8.1, 1.8 Hz, 1H), 7.20-7.15 (m, 3H), 6.97 (t, *J* = 7.7 Hz, 1H), 6.88 (t, *J* = 6.1 Hz, 1H), 6.42 (d, *J* = 9.2 Hz, 1H), 5.62 (brs, 1H), 5.29 (brs, 1H), 1.25 (s, 9H); ^{13}C NMR (150 MHz, $CDCl_3$) (representative peaks) δ 154.9, 152.1, 79.6, 57.4, 43.2, 28.2; **Minor isomer**: ^{1}H NMR (600 MHz, $CDCl_3$) (representative peaks) δ 7.98 (d, *J* = 8.4 Hz, 1H), 7.73 (d, *J* = 7.8 Hz, 1H), 7.68 (d, *J* = 8.2 Hz, 1H), 6.71 (d, *J* = 11.6 Hz, 1H), 6.60 (dd, *J* = 9.2, 6.3 Hz, 1H), 5.73 (dd, *J* = 11.1, 9.8 Hz, 1H), 1.22 (s, 9H); ^{13}C NMR (150 MHz, $CDCl_3$) (representative peaks) δ 155.5, 153.5, 79.1, 58.2, 42.5, 28.2; IR (ATR): 3320, 2976, 1698, 1508, 1456, 1365, 1260, 1166, 1016 cm^{-1}; HRMS (ESI) m/z calcd for $C_{28}H_{28}N_2NaO_3$ $[M+Na]^+$ 463.1992, found: 463.1992; HPLC conditions: DAICEL Chiralpak AD-3 column, hexane/*i*-PrOH = 70/30, 254 nm, 1.0 mL/min, 40 °C, t_{major} = 7.29 min, t_{minor} = 10.62 min; $[\alpha]^{27}_D$ = -88.4 (c = 0.36, CH_3OH).

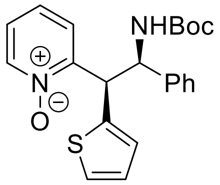

2-((1*R*,2*R*)-2-((*tert*-Butoxycarbonyl)amino)-2-phenyl-1-(thiophen-2-yl)ethyl)pyridine *N*-oxide (4ma) 92% yield, 60/40 dr, 86% ee; **Major isomer**: ^{1}H NMR (600 MHz, $CDCl_3$) δ 8.18 (d, *J* = 6.0 Hz, 1H), 7.44 (d, *J* = 7.5 Hz, 1H), 7.40-6.98 (m, 9H), 6.95 (dd, *J* = 5.1, 3.6 Hz, 1H), 5.90 (brs, 1H), 5.61 (t, *J* = 8.0 Hz, 1H), 5.54 (brs, 1H), 1.33 (s, 9H); ^{13}C NMR (150 MHz, $CDCl_3$) (representative peaks) δ 154.9, 151.3, 79.6, 56.8, 44.5, 28.2; **Minor isomer**: ^{1}H NMR (600 MHz, $CDCl_3$) (representative peaks) δ 8.32 (d, *J* = 6.3 Hz, 1H), 7.51 (d, *J* = 7.9 Hz, 1H), 6.87 (dd, *J* = 4.7, 3.9 Hz, 1H), 6.64 (dd, *J* = 7.9, 6.6 Hz, 1H), 5.97 (d, *J* = 11.1 Hz, 1H), 5.36 (t, *J* = 10.3 Hz, 1H), 1.23 (s, 9H); ^{13}C NMR (150 MHz, $CDCl_3$) (representative peaks) δ 155.3, 152.9, 79.1, 59.9, 44.8, 28.2; IR (ATR): 3236, 2974, 1703, 1541, 1491, 1431, 1364, 1289, 1248, 1169, 794, 700 cm^{-1}; HRMS (ESI) m/z calcd for $C_{22}H_{24}N_2NaO_3S$ $[M+Na]^+$ 419.1400, found: 419.1400; HPLC conditions: DAICEL Chiralpak IA-3 column, hexane/*i*-PrOH = 70/30, 254 nm, 0.5 mL/min, 30 °C, t_{major} = 18.38 min, t_{minor} = 25.98 min; $[\alpha]^{27}_D$ = 14.2 (c = 0.73, CH_3OH).

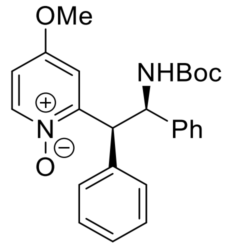

2-((1*R*,2*R*)-2-((*tert*-Butoxycarbonyl)amino)-1,2-diphenylethyl)-4-methoxypyridine *N*-oxide (4na) 93% yield, 89/11 dr, 92% ee; **Major isomer**: ^{1}H NMR (600 MHz, $CDCl_3$) δ 7.99 (d, *J* = 7.2 Hz, 1H), 7.46-7.07 (m, 10H), 6.94 (d, *J* = 3.4 Hz, 1H), 6.54 (dd, *J* = 7.2, 3.3 Hz, 1H), 5.57 (brs, 2H), 5.15 (d, *J* = 8.3 Hz, 1H), 3.73 (s, 3H), 1.27 (s, 9H); ^{13}C NMR (150 MHz, $CDCl_3$) (representative peaks) δ 157.3, 155.1, 79.8, 56.2, 56.0, 48.4, 28.3; **Minor isomer**: ^{1}H NMR (600 MHz, $CDCl_3$) (representative peaks) δ 8.16 (t, *J* = 7.3 Hz, 1H), 6.98 (dd, *J* = 13.1, 3.2 Hz, 1H), 6.72-6.62 (m, 1H), 5.63 (d, *J* = 11.4 Hz, 1H), 5.43 (dd, *J* = 11.2, 9.6 Hz, 1H), 3.79 (d, *J* = 4.8 Hz, 3H), 1.25 (s, 9H); ^{13}C NMR (150 MHz, $CDCl_3$) (representative peaks) δ 158.2, 155.8, 79.2, 58.4, 56.1, 50.4, 28.4; IR (ATR): 3436, 2976, 1705, 1492, 1458, 1430, 1307, 1226, 1170, 1039 cm^{-1}; HRMS (ESI) m/z calcd for $C_{25}H_{28}N_2NaO_4$ $[M+Na]^+$ 443.1941, found: 443.1941; HPLC conditions: DAICEL Chiralpak AD-3 column, hexane/*i*-PrOH = 70/30, 254 nm, 1.0 mL/min, 40 °C, t_{major} = 5.64 min, t_{minor} = 7.73 min; $[\alpha]^{27}_{D}$ = 97.1 (c = 0.43, CH_3OH).

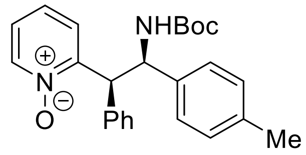

2-((1*R*,2*R*)-2-((*tert*-Butoxycarbonyl)amino)-1-phenyl-2-(*p*-tolyl)ethyl)pyridine *N*-oxide (4ab) 76% yield, 78/22 dr, 90% ee; **Major isomer**: ^{1}H NMR (600 MHz, $CDCl_3$) δ 8.07 (dd, *J* = 6.5, 0.9 Hz, 1H), 7.55-6.91 (m, 12H), 5.56 (brs, 2H), 5.00 (brs, 1H), 2.24 (s, 3H), 1.26 (s, 9H); ^{13}C NMR (150 MHz, $CDCl_3$) (representative peaks) δ 155.0, 152.3, 79.7, 55.9, 48.1, 28.3, 21.1; **Minor isomer**: ^{1}H NMR (600 MHz, $CDCl_3$) (representative peaks) δ 8.28 (d, *J* = 6.4 Hz, 1H), 6.54-6.31 (m, 1H), 5.64 (d, *J* = 11.4 Hz, 2H), 5.43 (dd, *J* = 11.0, 10.0 Hz, 1H), 2.22 (s, 3H), 1.23 (s, 9H); ^{13}C NMR (150 MHz, $CDCl_3$) (representative peaks) δ 155.6, 153.2, 79.2, 58.0, 49.7, 28.4; IR (ATR): 3223, 2977, 1695, 1515, 1490, 1431, 1365, 1236, 1167, 1014, 750 cm^{-1};HRMS (ESI) m/z calcd for $C_{25}H_{28}N_2NaO_3$ $[M+Na]^+$ 427.1992, found: 427.1992; HPLC conditions: DAICEL Chiralpak AD-3 column, hexane/*i*-PrOH = 70/30, 254 nm, 1.0 mL/min, 40 °C, t_{major} = 6.26 min, t_{minor} = 8.82 min; $[\alpha]^{27}_{D}$ = 69.8 (c = 0.51, CH_3OH).

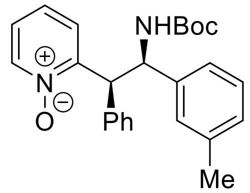

2-((1*R*,2*R*)-2-((*tert*-Butoxycarbonyl)amino)-1-phenyl-2-(*m*-tolyl)ethyl)pyridine *N*-oxide (4ac) 76% yield, 77/23 dr, 86% ee; **Major isomer**: ^{1}H NMR (600 MHz, $CDCl_3$) δ 8.08 (d, *J* = 6.4 Hz, 1H), 7.57-6.72 (m, 12H), 5.53 (brs, 2H), 5.03 (brs, 1H), 2.24 (s, 3H), 1.26 (s, 9H); ^{13}C NMR (150 MHz, $CDCl_3$) (representative peaks) δ 155.0, 152.3, 79.7, 56.1, 48.5, 28.3, 21.5; **Minor isomer**: ^{1}H NMR (600 MHz, $CDCl_3$) (representative peaks) δ 8.33-8.23 (m, 1H), 6.66-6.35 (m, 1H), 5.63 (d, *J* = 11.4 Hz, 1H), 5.43 (t, *J* = 10.5 Hz, 1H), 1.23 (s, 9H); ^{13}C NMR (150 MHz, $CDCl_3$) (representative peaks) δ 155.6, 153.1, 79.2, 58.3, 49.9, 28.4; IR (ATR): 3220, 2977, 1693, 1492, 1429, 1365, 1233, 1168, 1041, 1014 cm^{-1}; HRMS (ESI) m/z calcd for $C_{25}H_{28}N_2NaO_3$ $[M+Na]^+$ 427.1992, found: 427.1992; HPLC conditions: DAICEL Chiralpak AD-3 column, hexane/*i*-PrOH = 70/30, 254 nm, 1.0 mL/min, 40 °C, t_{major} = 5.13 min, t_{minor} = 6.78 min; $[\alpha]^{27}_{D}$ = 58.8 (c = 0.56, CH_3OH).

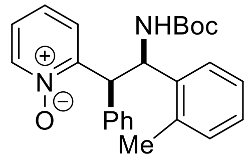

2-(2-((*tert*-Butoxycarbonyl)amino)-1-phenyl-2-(*o*-tolyl)ethyl)pyridine *N*-oxide (4ad) 45% yield, 47/53 dr, 56/7% ee; **Major isomer**: ^{1}H NMR (600 MHz, $CDCl_3$) δ 8.25 (d, *J* = 6.2 Hz, 1H), 7.71 (d, *J* = 5.8 Hz, 1H), 7.47-6.87 (m, 11H), 6.50 (s, 1H), 5.84-5.68 (m, 2H), 2.23 (s, 3H), 1.24 (s, 9H); ^{13}C NMR (150 MHz, $CDCl_3$) (representative peaks) δ 155.6, 153.1, 79.2, 58.3, 49.9, 28.4; **Minor isomer**: ^{1}H NMR (600 MHz, $CDCl_3$) (representative peaks) δ 8.09 (d, *J* = 6.4 Hz, 1H), 7.63 (s, 1H), 5.56 (brs, 1H), 4.86 (brs, 1H), 2.47 (s, 3H), 1.25 (s, 9H); ^{13}C NMR (150 MHz, $CDCl_3$) (representative peaks) δ 154.6, 152.3, 79.5, 52.1, 47.6, 28.1; IR (ATR): 3230, 2976, 1699, 1541, 1522, 1508, 1432, 1365, 1171, 1040, 1012 cm^{-1}; HRMS (ESI) m/z calcd for $C_{25}H_{28}N_2NaO_3$ $[M+Na]^+$ 427.1992, found: 427.1992; HPLC conditions: DAICEL Chiralpak AD-3 column, hexane/*i*-PrOH = 70/30, 254 nm, 1.0 mL/min, 40 °C, t_{major} = 5.83 min, t_{minor} = 11.57 min; $[\alpha]^{27}_{D}$ = 56.4 (c = 0.33, CH_3OH).

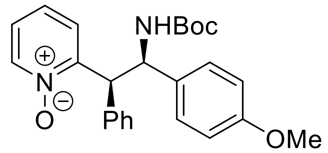

2-((1*R*,2*R*)-2-((*tert*-Butoxycarbonyl)amino)-2-(4-methoxyphenyl)-1-phenylethyl)pyridine *N*-oxide (4ae) 91% yield, 83/17 dr, 92% ee; **Major isomer**: ^{1}H NMR (600 MHz, $CDCl_3$) δ 8.07 (dd, *J* = 6.4, 0.9 Hz, 1H), 7.61-6.90 (m, 10H), 6.80-6.68 (m, 2H), 5.58 (brs, 1H), 5.53 (brs, 1H), 5.07 (d, *J* = 7.5 Hz, 1H), 3.71 (s, 3H), 1.25 (s, 9H); ^{13}C NMR (150 MHz, $CDCl_3$) (representative

peaks) δ 158.8, 154.9, 79.5, 55.5, 55.1, 48.0, 28.2; **Minor isomer**: ^{1}H NMR (600 MHz, $CDCl_3$) (representative peaks) δ 8.26 (t, *J* = 5.6 Hz, 1H), 6.55 (dd, *J* = 28.2, 9.5 Hz, 1H), 5.61 (d, *J* = 11.5 Hz, 1H), 5.41 (t, *J* = 10.8 Hz, 1H), 3.70 (s, 3H), 1.23 (s, 9H); ^{13}C NMR (150 MHz, $CDCl_3$) (representative peaks) δ 158.6, 155.5, 79.0, 57.6, 55.1, 49.8, 28.2; IR (ATR): 3271, 2976, 1686, 1512, 1241, 1166, 1035, 1013, 829, 746 cm^{-1}; HRMS (ESI) m/z calcd for $C_{25}H_{28}N_2NaO_4$ $[M+Na]^+$ 443.1941, found: 443.1941; HPLC conditions: DAICEL Chiralpak AD-3 column, hexane/*i*-PrOH = 70/30, 254 nm, 1.0 mL/min, 40 °C, t_{major} = 7.50 min, t_{minor} = 13.00 min; $[\alpha]^{27}_{D}$ = 78.4 (c = 0.39, CH_3OH).

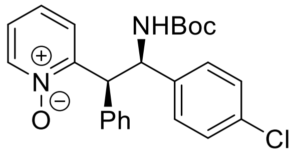

2-((1*R*,2*R*)-2-((*tert*-Butoxycarbonyl)amino)-2-(4-chlorophenyl)-1-phenylethyl)pyridine 1-oxide (4af) 81% yield, 80/20 dr, 89% ee; **Major isomer**: ^{1}H NMR (600 MHz, $CDCl_3$) δ 8.08 (dd, *J* = 6.4, 0.9 Hz, 1H), 7.56-6.95 (m, 12H), 5.67-5.50 (m, 2H), 5.13 (brs, 1H), 1.27 (s, 9H); ^{13}C NMR (150 MHz, $CDCl_3$) (representative peaks) δ 154.9, 151.8, 79.9, 55.6, 47.9, 28.2; **Minor isomer**: ^{1}H NMR (600 MHz, $CDCl_3$) (representative peaks) δ 8.28 (t, *J* = 6.1 Hz, 1H), 6.81 (dd, *J* = 28.0, 9.1 Hz, 1H), 5.41 (t, *J* = 10.2 Hz, 1H), 1.24 (d, *J* = 3.6 Hz, 9H); ^{13}C NMR (150 MHz, $CDCl_3$) (representative peaks) δ 155.5, 152.6, 79.3, 58.0, 49.7, 28.2; IR (ATR): 3263, 2978, 1698, 1522, 1508, 1431, 1365, 1168, 1090, 1014 cm^{-1}; HRMS (ESI) m/z calcd for $C_{24}H_{25}ClN_2NaO_3$ $[M+Na]^+$ 447.1446, found: 447.1446; HPLC conditions: DAICEL Chiralpak AD-3 column, hexane/*i*-PrOH = 70/30, 254 nm, 1.0 mL/min, 40 °C, t_{major} = 6.59 min, t_{minor} = 11.52 min; $[\alpha]^{27}_{D}$ = 41.7 (c = 0.55, CH_3OH).

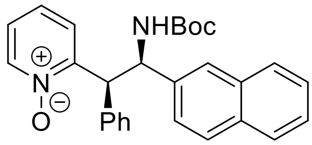

2-((1*R*,2*R*)-2-((*tert*-Butoxycarbonyl)amino)-2-(naphthalen-2-yl)-1-phenylethyl)pyridine *N*-oxide (4ag) 66% yield, 74/26 dr, 88% ee; **Major isomer**: ^{1}H NMR (600 MHz, $CDCl_3$) δ 8.06 (d, *J* = 6.3 Hz, 1H), 7.83-6.82 (m, 15H), 5.75 (brs, 2H), 5.27 (d, *J* = 6.9 Hz, 1H), 1.26 (s, 9H); ^{13}C NMR (150 MHz, $CDCl_3$) (representative peaks) δ 155.1, 152.2, 79.8, 56.4, 48.2, 28.3; **Minor isomer**: ^{1}H NMR (600 MHz, $CDCl_3$) (representative peaks) δ 8.29 (d, *J* = 6.4 Hz, 1H), 5.80 (dd, *J* = 11.6, 2.9 Hz, 1H), 5.64 (t, *J* = 10.4 Hz, 1H), 1.24 (d, *J* = 4.0 Hz, 9H); ^{13}C NMR (150 MHz, $CDCl_3$) (representative peaks) δ 155.7, 153.1, 79.3, 58.6, 49.8, 28.4; HRMS (ESI) m/z calcd for $C_{28}H_{28}N_2NaO_3$ $[M+Na]^+$ 463.1992, found: 463.1992; IR (ATR): 3203, 2972, 1699, 1490, 1432, 1365, 1236, 1169, 1014, 822, 749 cm^{-1}; HPLC conditions: DAICEL Chiralpak AD-3 column, hexane/*i*-PrOH = 70/30, 254 nm, 1.0 mL/min, 40 °C, t_{major} = 7.25 min, t_{minor} = 10.42 min; $[\alpha]^{27}_{D}$ = 93.6 (c = 0.44, CH_3OH).

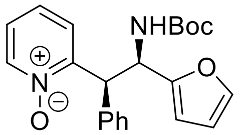

2-((1*R*,2*R*)-2-((*tert*-Butoxycarbonyl)amino)-2-(furan-2-yl)-1-phenylethyl)pyridine *N*-oxide (4ah) 61% yield, 67/33 dr, 71% ee; **Major isomer**: ^{1}H NMR (600 MHz, $CDCl_3$) δ 8.13 (d, *J* = 6.4 Hz, 1H), 7.54-6.97 (m, 9H), 6.14 (s, 2H), 5.68 (d, *J* = 8.2 Hz, 1H), 5.52 (brs, 1H), 5.41 (brs, 1H), 1.30 (s, 9H); ^{13}C NMR (150 MHz, $CDCl_3$) (representative peaks) δ 155.1, 153.5, 152.1, 79.9, 50.7, 47.8, 28.3; **Minor isomer**: ^{1}H NMR (600 MHz, $CDCl_3$) (representative peaks) δ 8.24 (d, *J* = 6.4 Hz, 1H), 6.12-6.10 (m, 1H), 5.72 (d, *J* = 12.0 Hz, 1H), 5.59 (t, *J* = 10.8 Hz, 1H), 1.26 (s, 9H); ^{13}C NMR (150 MHz, $CDCl_3$) (representative peaks) δ 155.5, 153.0, 152.4, 79.6, 51.7, 47.6; IR (ATR): 3216, 2976, 1698, 1490, 1431, 1240, 1168, 1011, 749 cm^{-1}; HRMS (ESI) m/z calcd for $C_{22}H_{24}N_2NaO_4$ $[M+Na]^+$ 431.1628, found: 431.1628; HPLC conditions: DAICEL Chiralpak OD-3 column, hexane/*i*-PrOH = 90/10, 254 nm, 1.0 mL/min, 30 °C, t_{major} = 11.59 min, t_{minor} = 14.65 min; $[\alpha]^{27}_D$ = 53.7 (c = 0.60, CH_3OH).

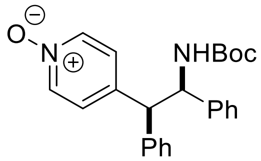

4-(2-((*tert*-Butoxycarbonyl)amino)-1,2-diphenylethyl)pyridine *N*-oxide (4oa) 90% yield, 82/18 dr, 32% ee; **Major isomer**: ^{1}H NMR (600 MHz, $CDCl_3$) δ 7.91 (d, *J* = 6.9 Hz, 2H), 7.79-7.04 (m, 10H), 6.90 (d, *J* = 6.3 Hz, 2H) 5.35 (brs, 1H), 5.10 (brs, 1H), 4.43-4.08 (m, 1H), 1.27 (s, 9H); ^{13}C NMR (150 MHz, $CDCl_3$) (representative peaks) δ154.8, 80.0, 56.9, 56.6, 28.2; **Minor isomer**: ^{1}H NMR (600 MHz, $CDCl_3$) (representative peaks) δ 8.08 (d, *J* = 7.0 Hz, 2H), 6.99 (d, *J* = 7.3 Hz, 2H), 1.31 (s, 9H); ^{13}C NMR (150 MHz, $CDCl_3$) (representative peaks) δ 154.9, 28.2; IR (ATR): 3217, 2977, 1704, 1540, 1487, 1365, 1242, 1174, 1013, 750, 700 cm^{-1}; HRMS (ESI) m/z calcd for $C_{24}H_{26}N_2NaO_3$ $[M+Na]^+$ 413.1836, found: 413.1835; HPLC conditions: DAICEL Chiralpak IA-3 column, hexane/*i*-PrOH = 70/30, 254 nm, 1.0 mL/min, 40 °C, t_{major} = 6.29 min, t_{minor} = 7.48 min; $[\alpha]^{26}_D$ = 28.5 (c = 0.11, CH_3OH).

6. Catalytic reaction in larger scale and reduction of pyridine *N*-oxide

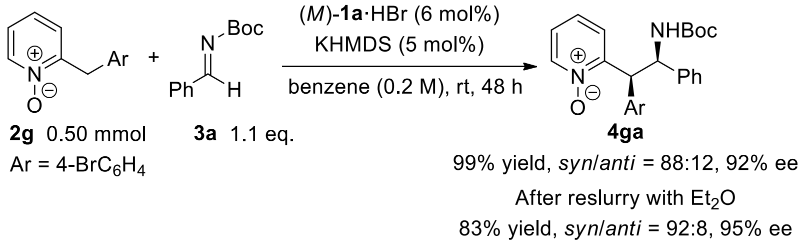

To a dried test tube was added (*M*)-**1a**·HBr (24.0 mg, 0.030 mmol), then 2.5 mL anhydrous benzene was added under argon atmosphere. A solution of $KN(SiMe_3)_2$ in toluene (0.5 M, 50 µL, 0.025 mmol) was added to the suspension at room temperature. After stirring for ca. 3 min, 2-(4-bromobenzyl)pyridine *N*-oxide **2g** (0.13 g, 0.50 mmol) was added in one portion and the mixture was stirred for 3 min. Then *N*-Boc imine **3a** (0.12 g, 0.60 mmol) was added to the solution. After that, the reaction mixture was stirred at room temperature for 48 h. The reaction was quenched with 3.0 mL saturated NH_4Cl aq. and extracted with CH_2Cl_2 (20 mL × 3). The combined organic phase was dried over sodium sulfate, and concentrated in vacuo. The residue was purified by silica gel column chromatography (hexane/ethyl acetate/Methanol = 3:9:1 as the eluent) to afford the corresponding product as a white solid **4ga** (0.23 g, 99% yield, 82/18 dr, 92% ee). The solid was reslurried in 20 mL ether for 2 h. After filtration, the product was obtained in 83% yield with the improvement of dr and ee values.

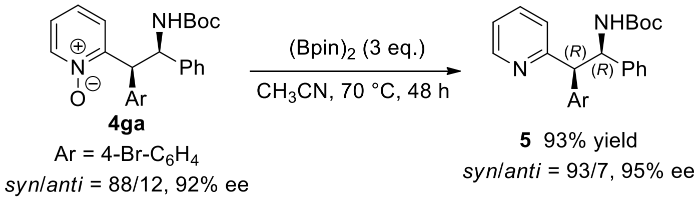

To a 30 mL two-neck flask were added the adduct **4ga** (94.0 mg, 0.20 mmol), bis(pinacolato)diboron (0.15 g, 0.60 mmol) and 4.0 mL acetonitrile. The solution was stirred at 70 ºC for 48 h. After that, the mixture was cooled to room temperature, 1.6 mL 1,2-diaminoethane was added, and the mixture was stirred at rt for 1 h. The product was extracted with CH_2Cl_2 (10 mL × 3). The combined organic phase was dried over sodium sulfate, and concentrated in vacuo. The residue was purified by silica gel column chromatography (hexane/ethyl acetate = 4:1 as the eluent) to afford **5** as a white solid.

***tert*-Butyl ((1*R*,2*R*)-2-(4-bromophenyl)-1-phenyl-2-(pyridin-2-yl)ethyl)carbamate (5)** 93% yield, 93/7 dr, 95% ee; ^{1}H NMR (600 MHz, $CDCl_3$) δ 8.48 (d, *J* = 4.1 Hz, 1H), 7.45-7.41 (m, 2H), 7.40-7.36 (m, 3H), 7.23-7.05 (m, 5H), 7.02-6.92 (m, 2H), 5.66 (brs, 1H), 5.01 (brs, 1H), 4.34 (brs, 1H), 1.27 (s, 9H); ^{13}C NMR (150 MHz, $CDCl_3$) δ159.9, 154.9, 149.2, 141.4, 139.1, 136.3, 131.4, 130.7, 128.1, 127.1, 127.0, 123.3, 121.6, 121.0, 83.5, 59.6, 28.1, 25.0; IR (ATR): 2917, 2849, 1687,

1510, 1170, 772 cm^{-1}; HRMS (ESI) m/z calcd for $C_{24}H_{25}BrN_2NaO_2$ $[M+Na]^+$ 475.0992, found: 475.0992; HPLC conditions: DAICEL Chiralpak AD-3 column, hexane/*i*-PrOH = 70/30, 254 nm, 1.0 mL/min, 40 °C, t_{major} = 7.46 min, t_{minor} = 7.06 min; $[\alpha]^{26}_{D}$ = -2.3 (c = 0.32, $CHCl_3$); CCDC No. 1811498.

ORTEP Diagram of 5 (recrystalized from THF)

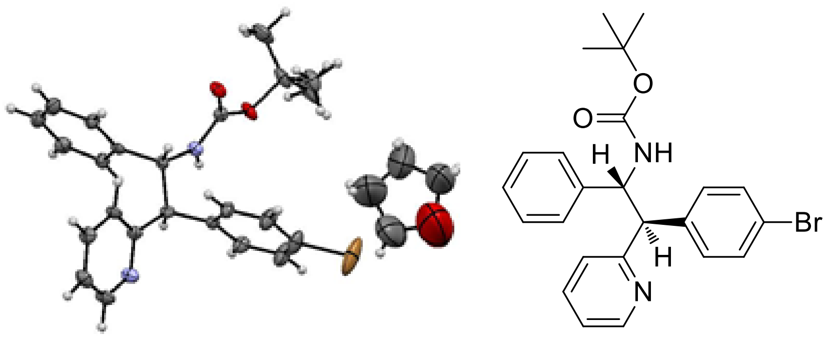

7. HPLC Charts

Compound **4aa**

HPLC conditions: DAICEL Chiralpak IA-3 column, hexane/*i*-PrOH = 70/30, 254 nm, 1.0 mL/min, 40 ℃, t_{major} = 5.81 min, t_{minor} = 7.88 min.

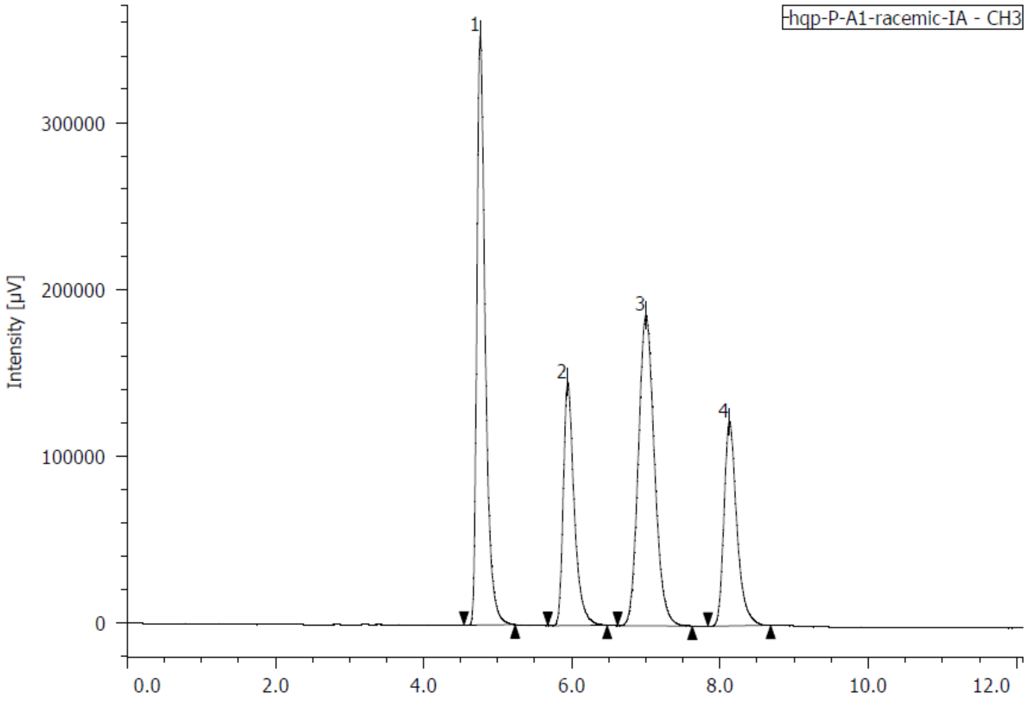

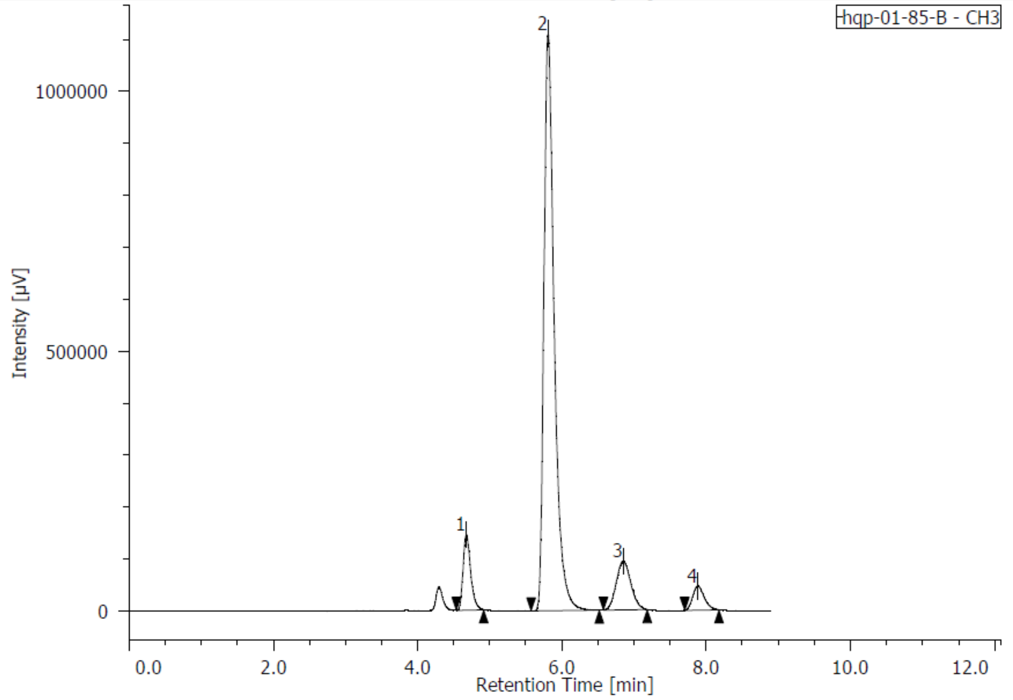

**Peak**	**1**	**2**	**3**	**4**
Retention Time (min)	4.68	5.81	6.85	7.88
Area (%)	7.7	79.0	9.6	3.7

Compound **4ba**

HPLC conditions: DAICEL Chiralpak IE-3 column, hexane/*i*-PrOH = 70/30, 254 nm, 1.0 mL/min, 40 °C, t_{major} = 24.1 min, t_{minor} = 25.8 min

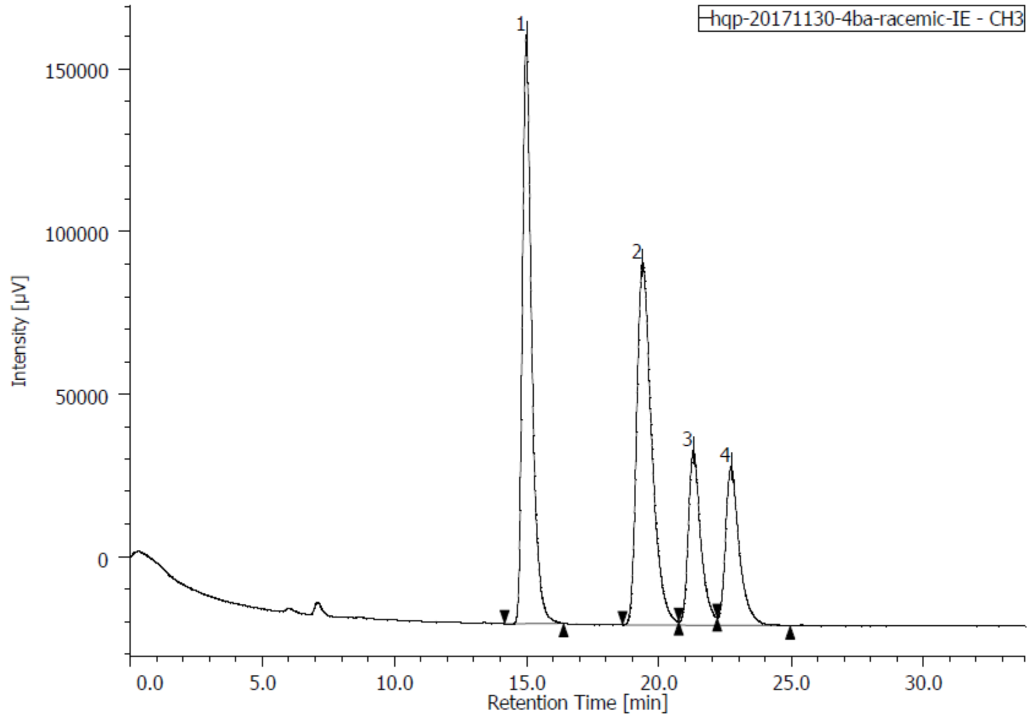

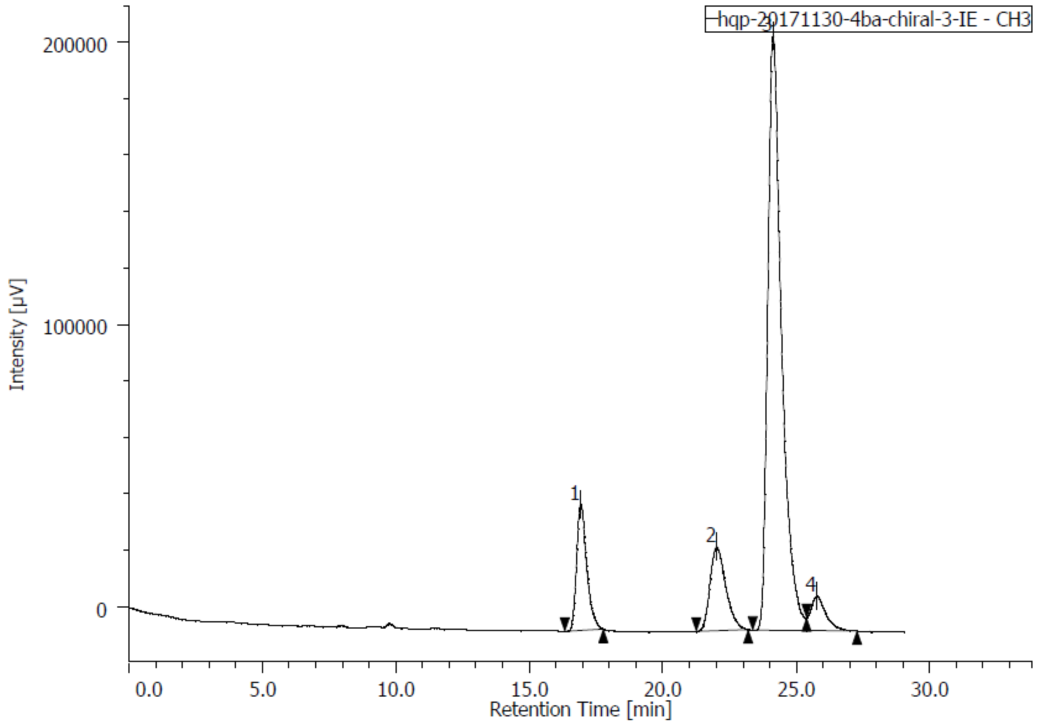

**Peak**	**1**	**2**	**3**	**4**
Retention Time (min)	16.9	22.0	24.1	25.8
Area (%)	11.2	11.6	72.5	4.7

Compound **4ca**

HPLC conditions: DAICEL Chiralpak AD-3 column, hexane/*i*-PrOH = 70/30, 254 nm, 1.0 mL/min, 40 °C, t_{major} = 6.08 min, t_{minor} = 8.10 min

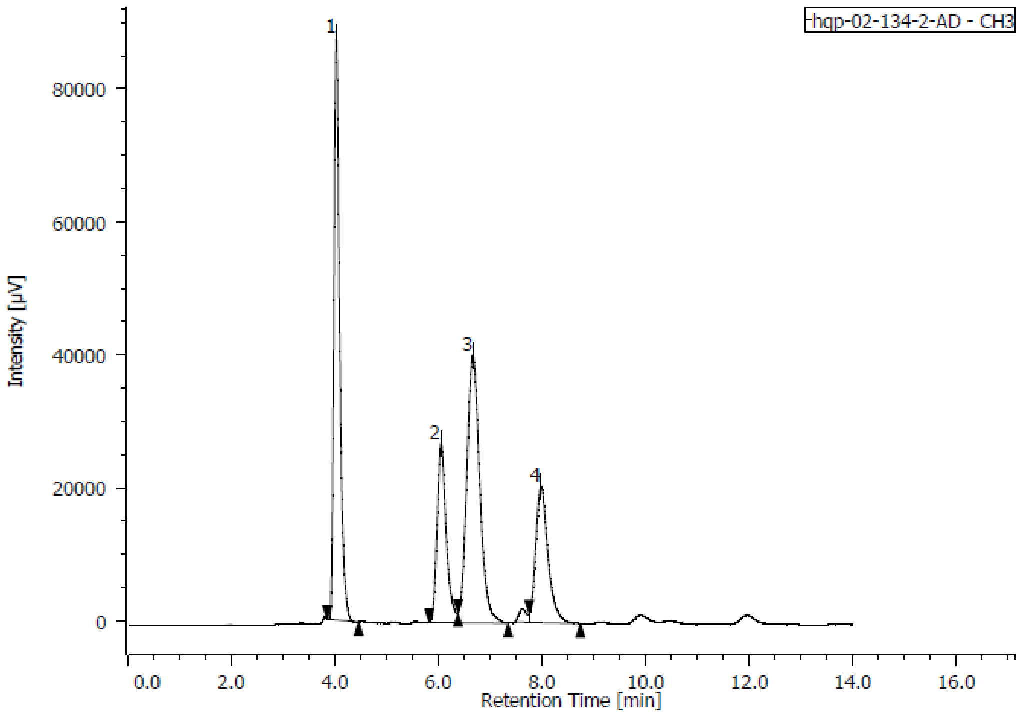

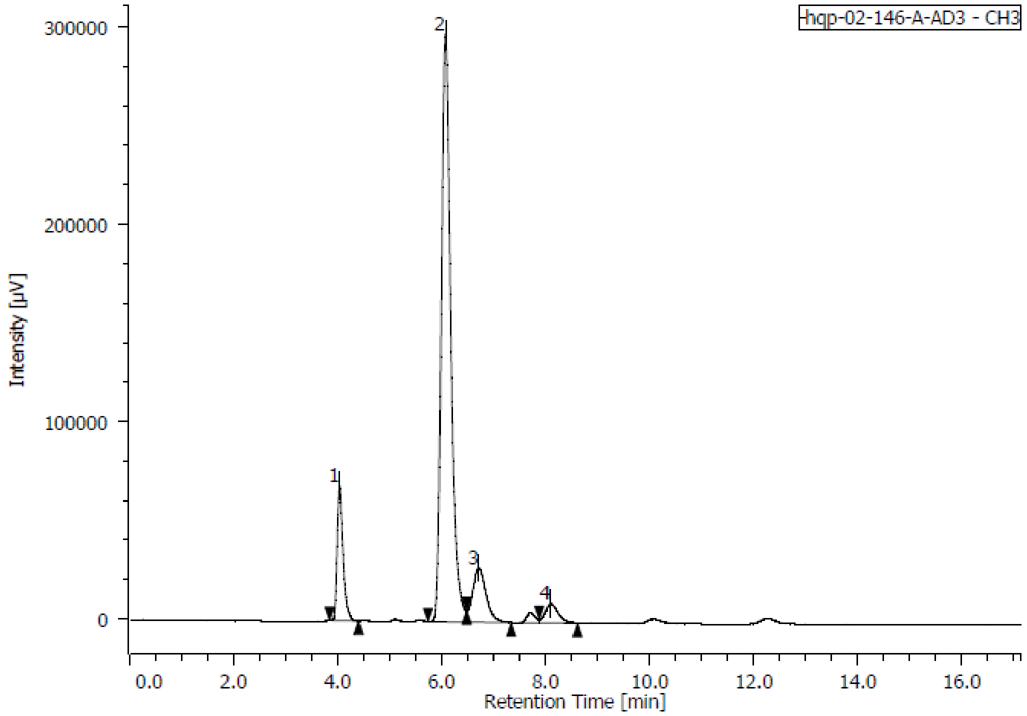

**Peak**	**1**	**2**	**3**	**4**
Retention Time (min)	4.04	6.07	6.71	8.10
Area (%)	11.1	75.5	10.1	3.3

Compound **4da**

HPLC conditions: DAICEL Chiralpak IA-3 column, hexane/*i*-PrOH = 70/30, 254 nm, 1.0 mL/min, 40 °C, t_{major} = 5.18 min, t_{minor} = 7.01 min;

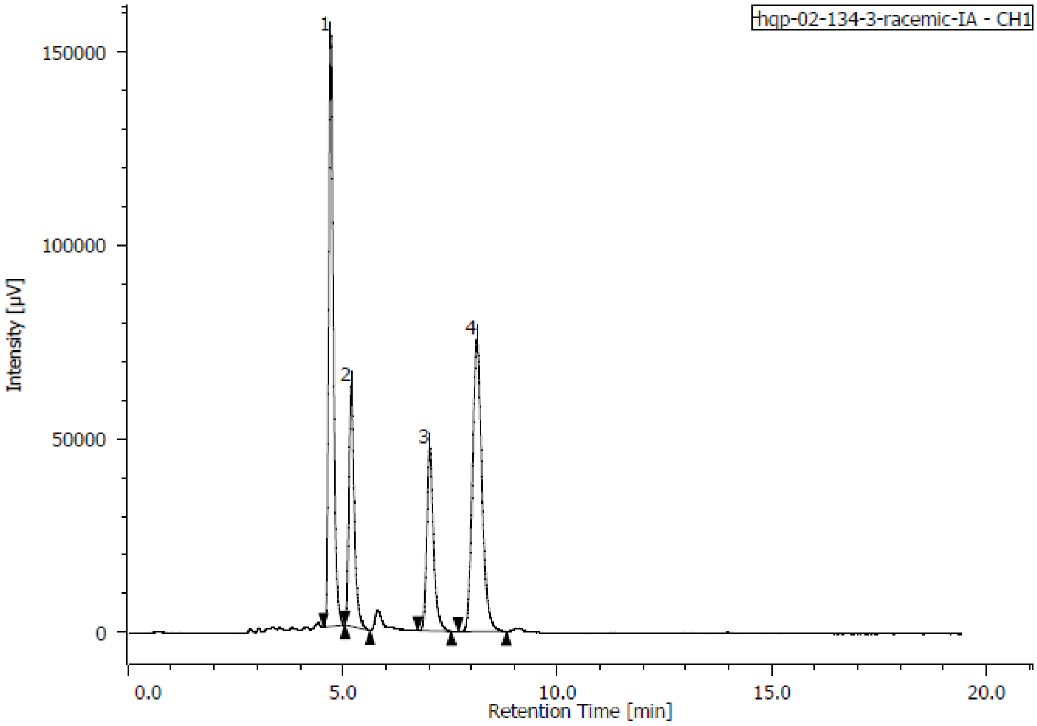

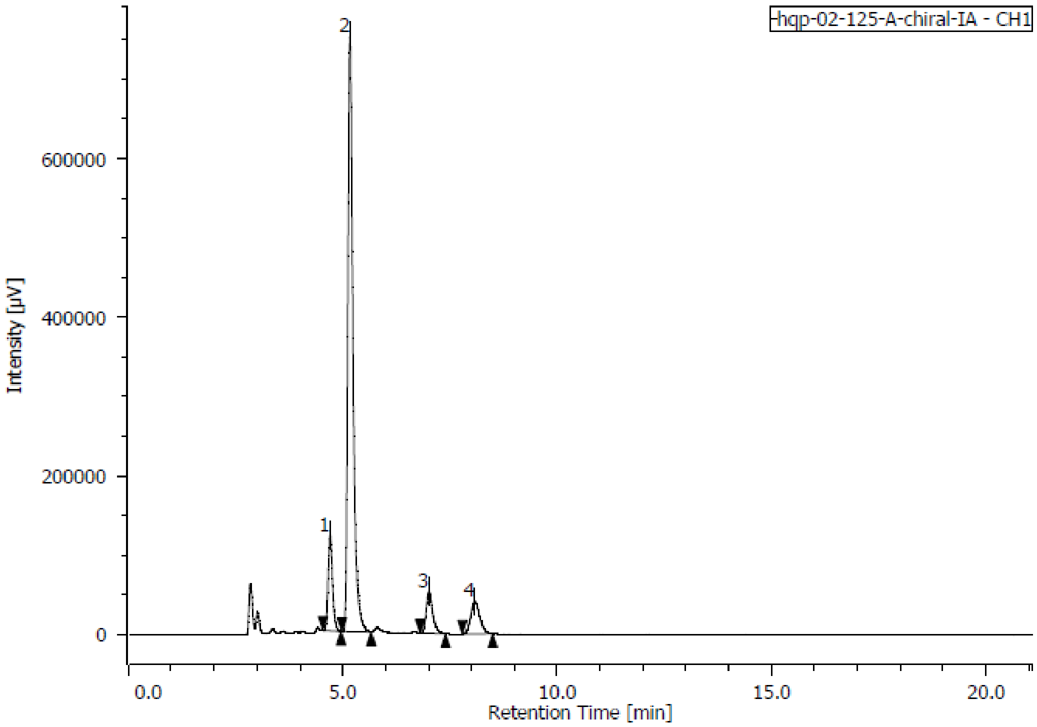

**Peak**	**1**	**2**	**3**	**4**
Retention Time (min)	4.71	5.18	7.01	8.08
Area (%)	10.0	76.1	6.7	7.2

Compound **4ea**

HPLC conditions: DAICEL Chiralpak AD-3 column, hexane/*i*-PrOH = 70/30, 254 nm, 1.0 mL/min, 40 °C, t_{major} = 6.76 min, t_{minor} = 10.48 min;

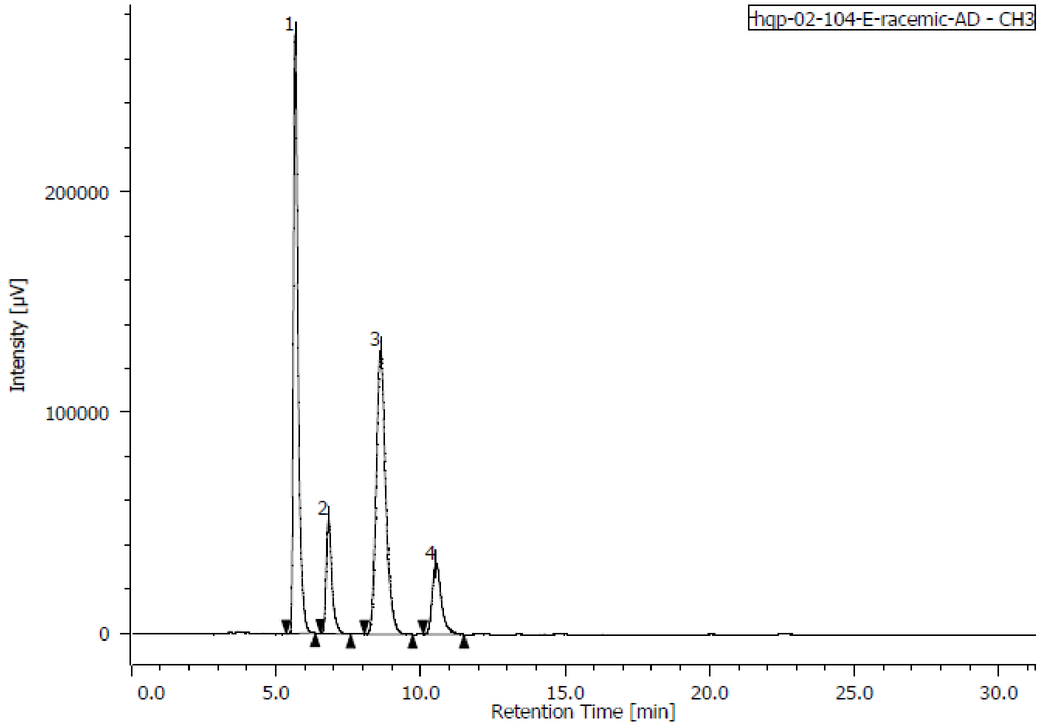

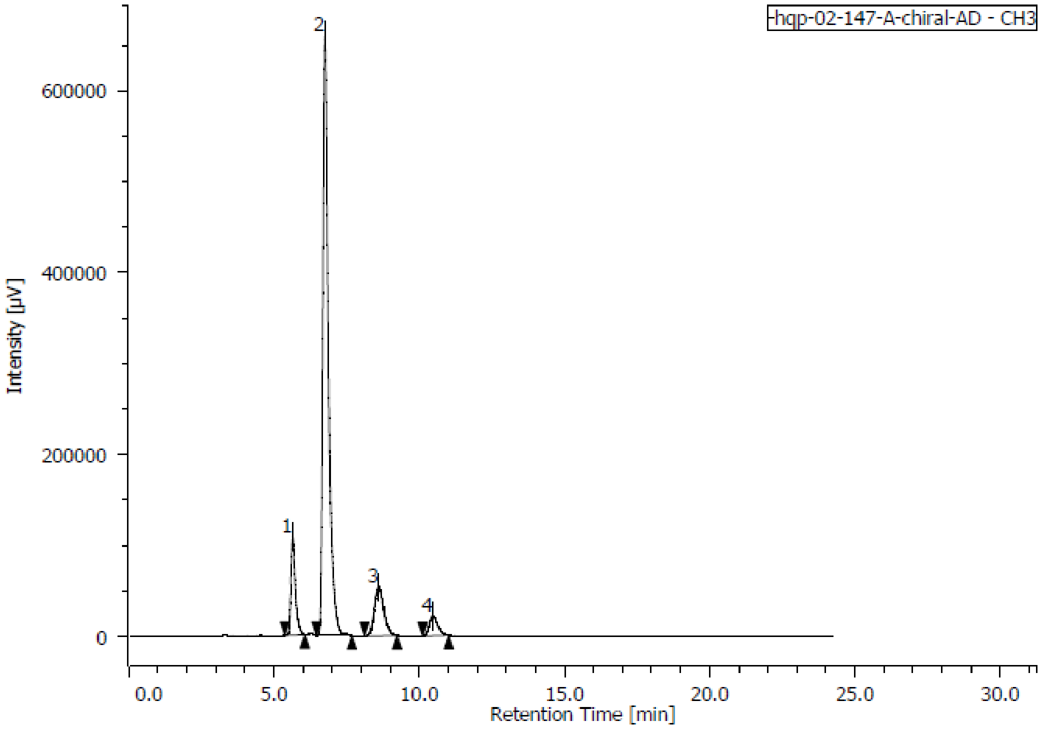

**Peak**	**1**	**2**	**3**	**4**
Retention Time (min)	5.64	6.76	8.61	10.48
Area (%)	9.8	76.1	10.3	3.8

Compound **4fa**

HPLC conditions: DAICEL Chiralpak AD-3 column, hexane/*i*-PrOH = 70/30, 254 nm, 1.0 mL/min, 40 °C, t_{major} = 10.55 min, t_{minor} = 18.00 min;

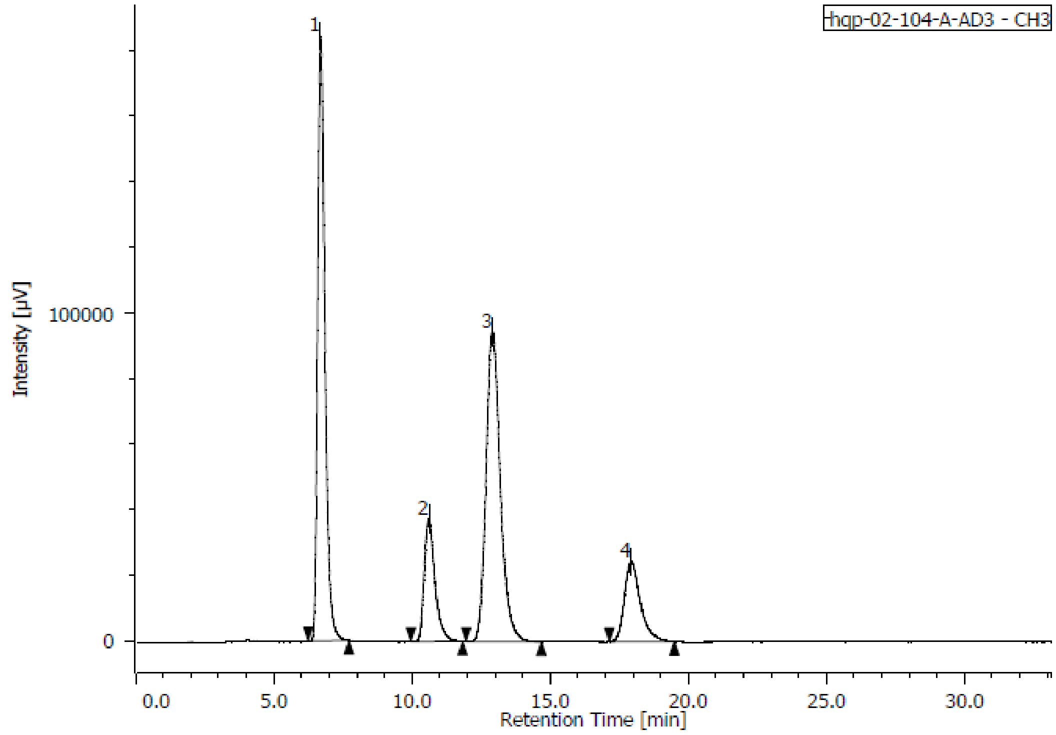

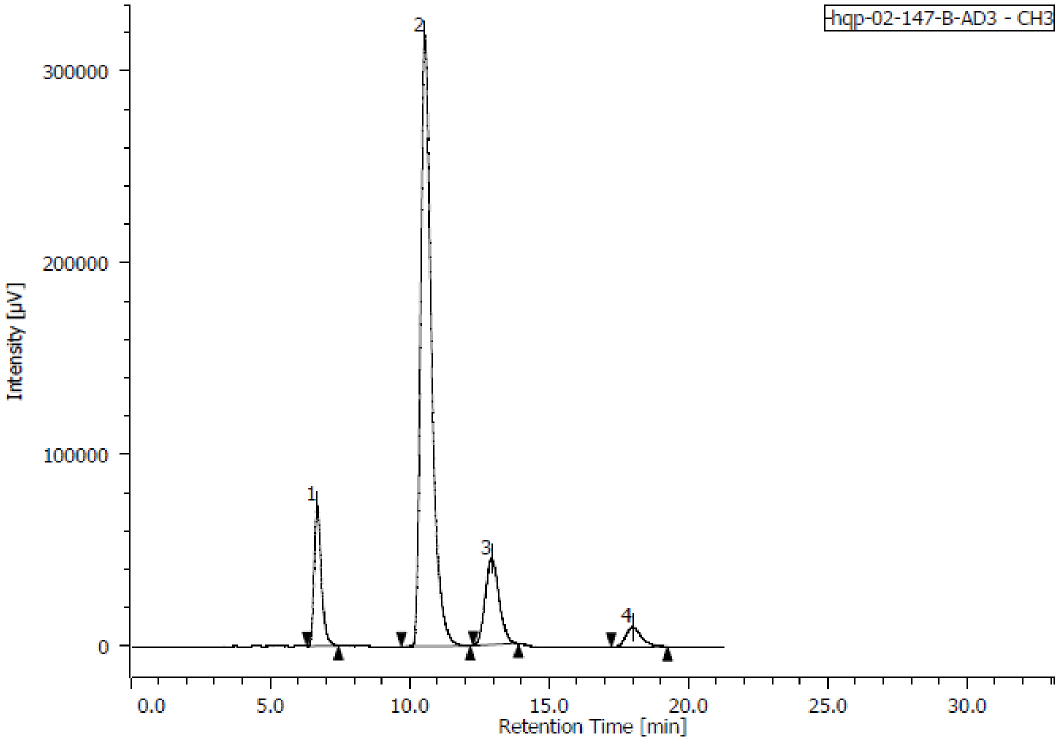

**Peak**	**1**	**2**	**3**	**4**
Retention Time (min)	6.68	10.55	12.94	18.00
Area (%)	11.1	72.1	13.2	3.6

Compound **4ga**

HPLC conditions: DAICEL Chiralpak AD-3 column, hexane/*i*-PrOH = 70/30, 254 nm, 1.0 mL/min, 40 °C, t_{major} = 6.58 min, t_{minor} = 9.39 min;

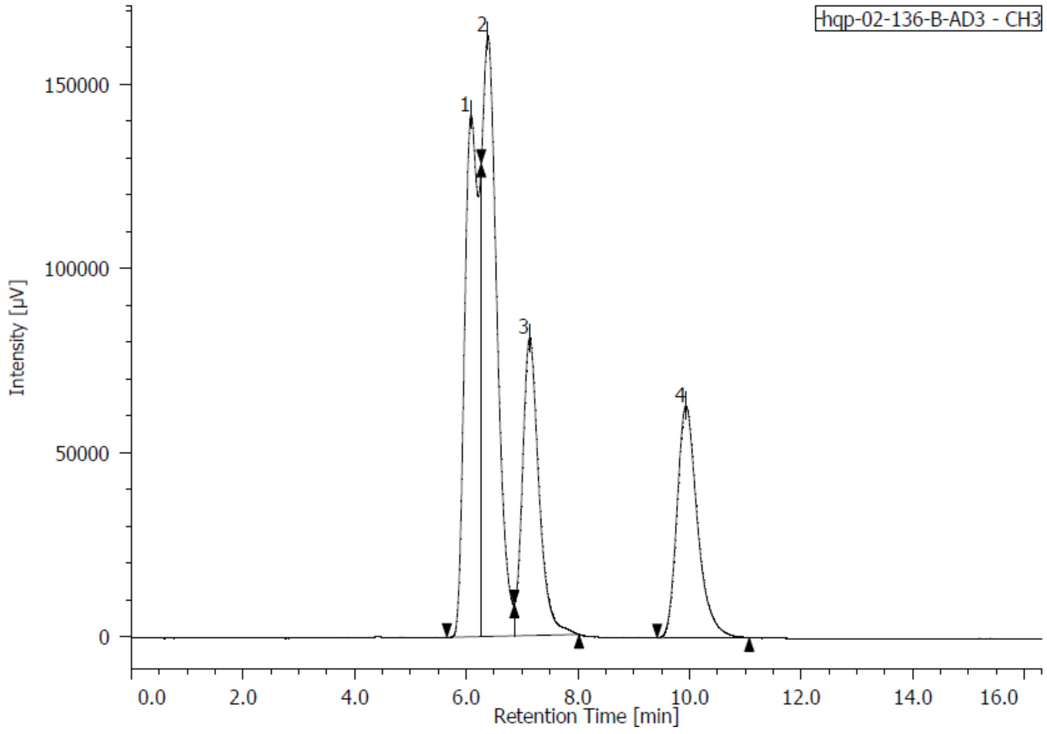

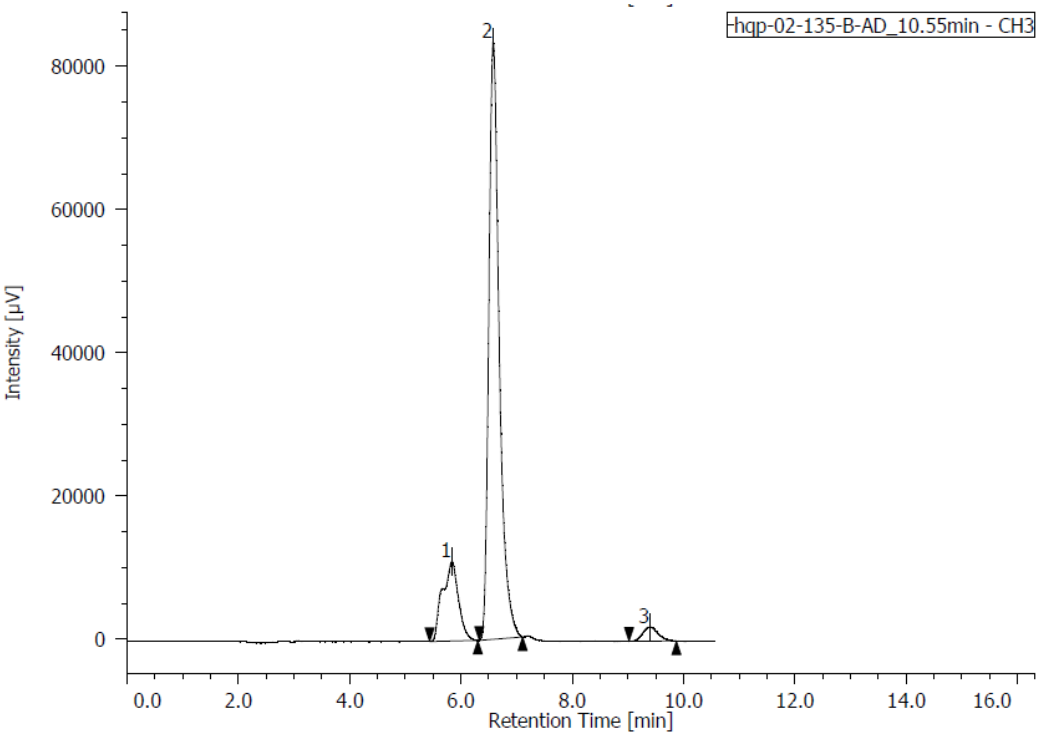

**Peak**	**1+2**	**3**	**4**
Retention Time (min)	5.83	6.58	9.39
Area (%)	16.5	80.7	2.8

Compound **4ha**

HPLC conditions: DAICEL Chiralpak AD-3 column, hexane/*i*-PrOH = 70/30, 254 nm, 1.0 mL/min, 40 ℃, t_{major} = 5.49 min, t_{minor} = 6.91 min;

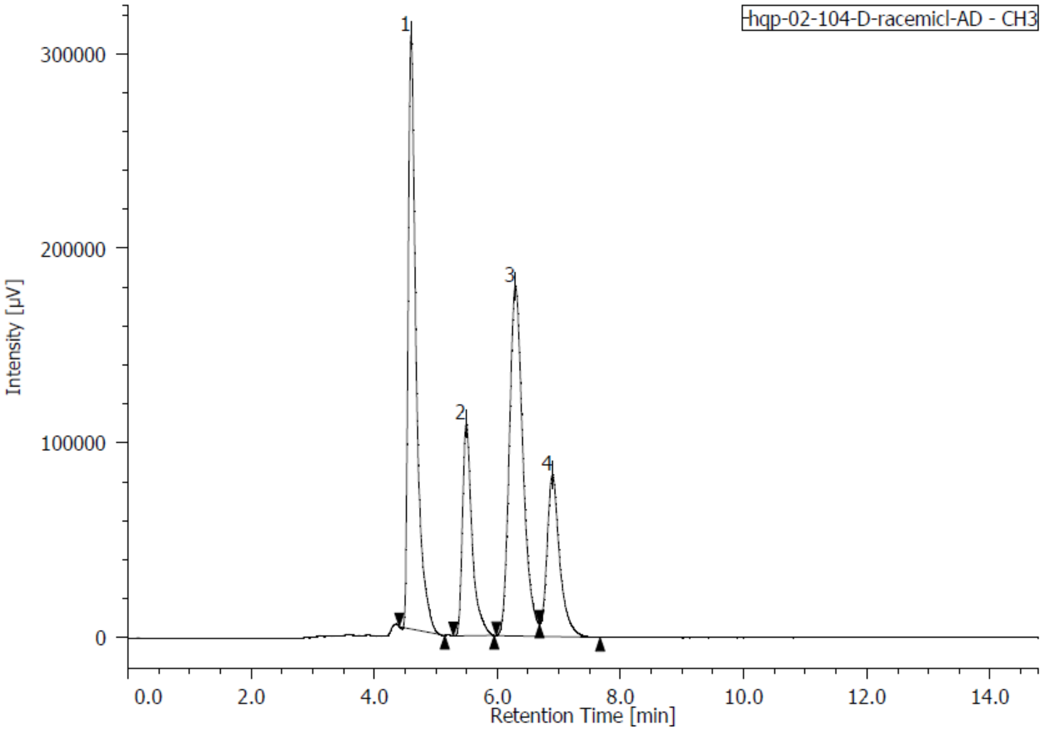

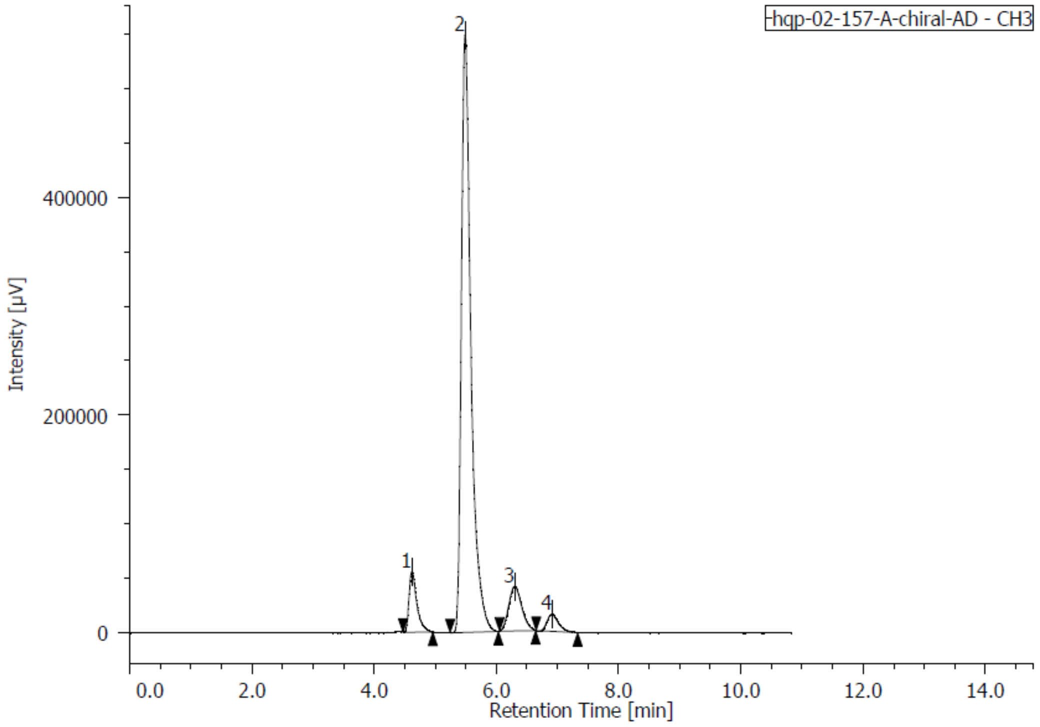

Peak	1	2	3	4
Retention Time (min)	4.62	5.49	6.30	6.91
Area (%)	6.8	82.1	8.3	2.8

Compound **4ia**

HPLC conditions: DAICEL Chiralpak AD-3 column, hexane/*i*-PrOH = 80/20, 254 nm, 0.8 mL/min, 40 °C, t_{major} = 8.88 min, t_{minor} = 12.52 min;

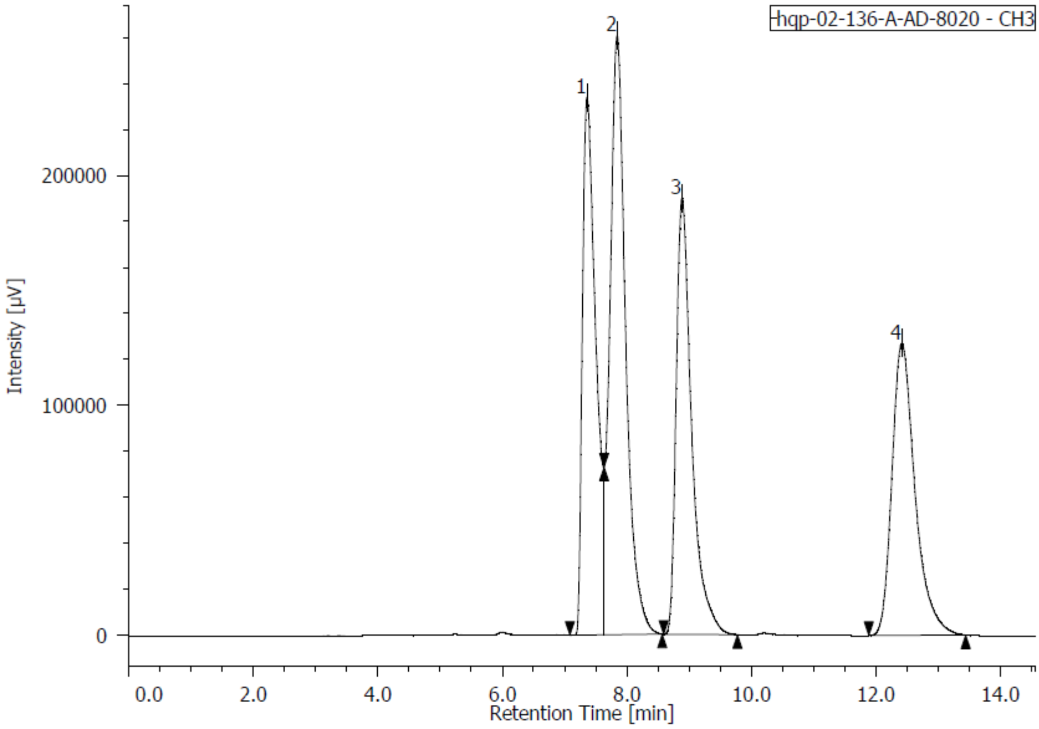

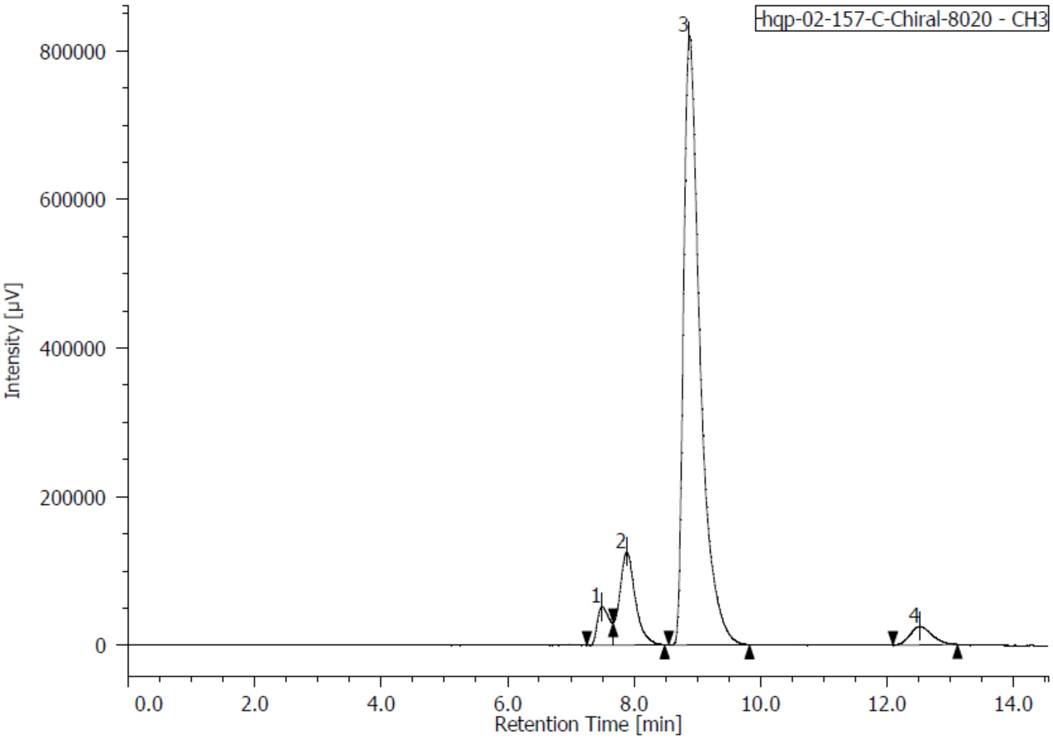

**Peak**	**1**	**2**	**3**	**4**
Retention Time (min)	7.49	7.88	8.88	12.52
Area (%)	3.8	11.8	81.1	3.3

Compound **4ja**

HPLC conditions: DAICEL Chiralpak OD-3 column, hexane/*i*-PrOH = 80/20, 254 nm, 1.0 mL/min, 40 °C, t_{major} = 5.55 min, t_{minor} = 6.79 min;

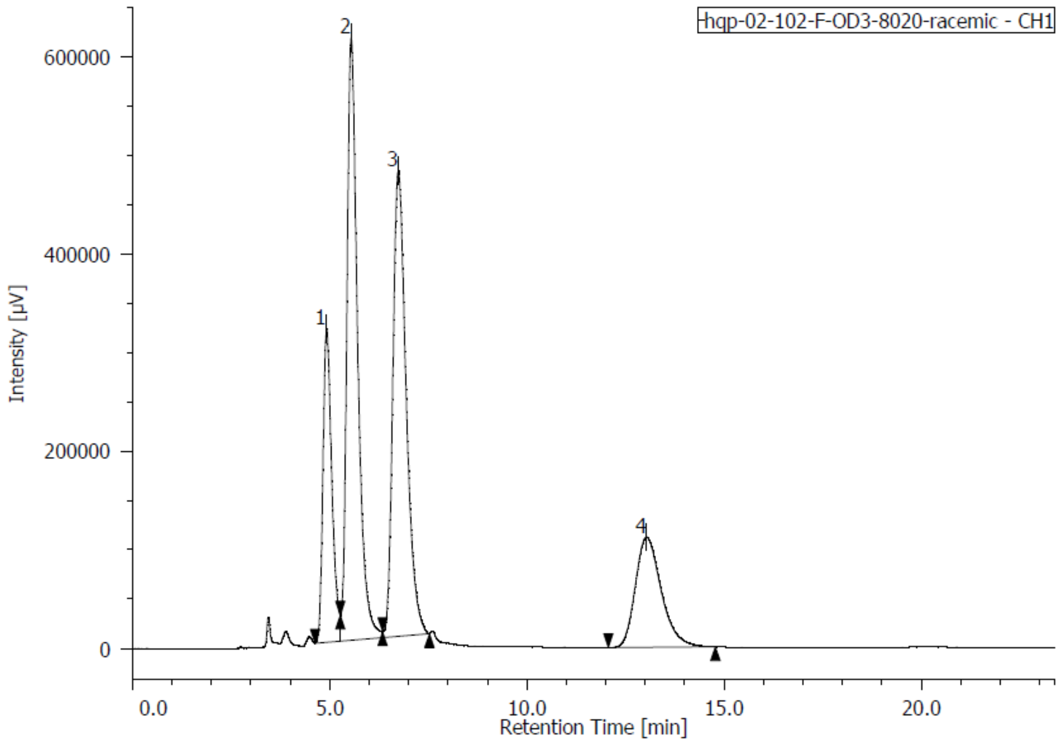

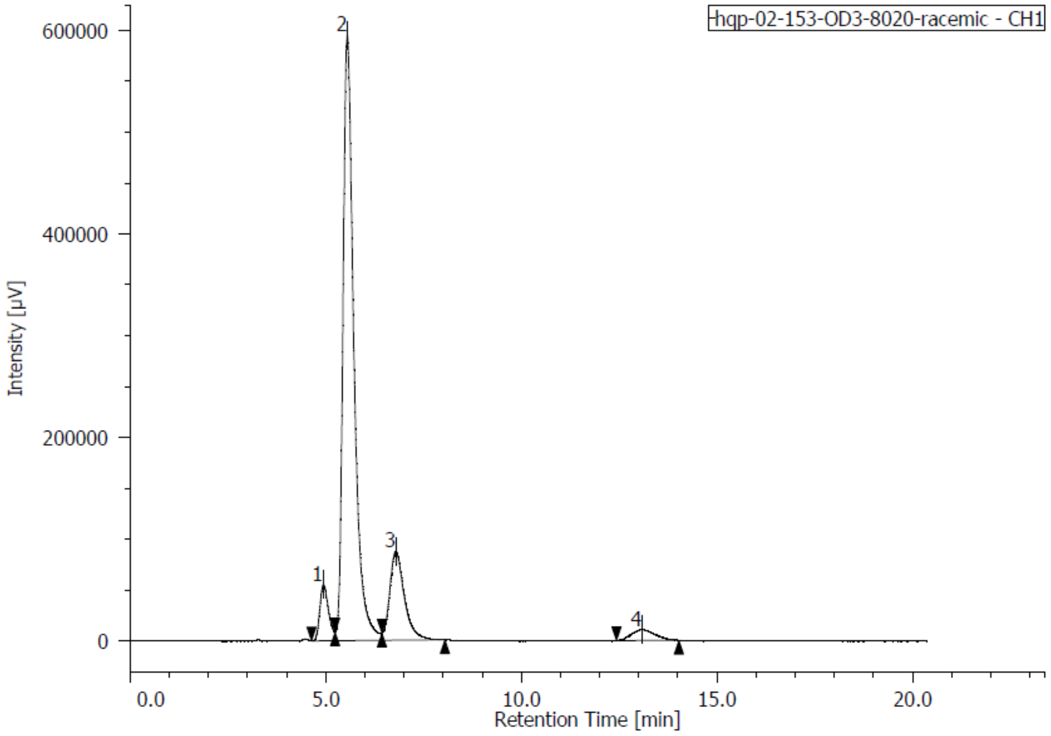

**Peak**	**1**	**2**	**3**	**4**
Retention Time (min)	4.94	5.55	6.79	13.08
Area (%)	5.7	76.2	15.1	3.0

Compound **4ka**

HPLC conditions: DAICEL Chiralpak AD-3 column, hexane/*i*-PrOH = 70/30, 254 nm, 1.0 mL/min, 40 °C, t_{major} = 9.06 min, t_{minor} = 14.25 min;

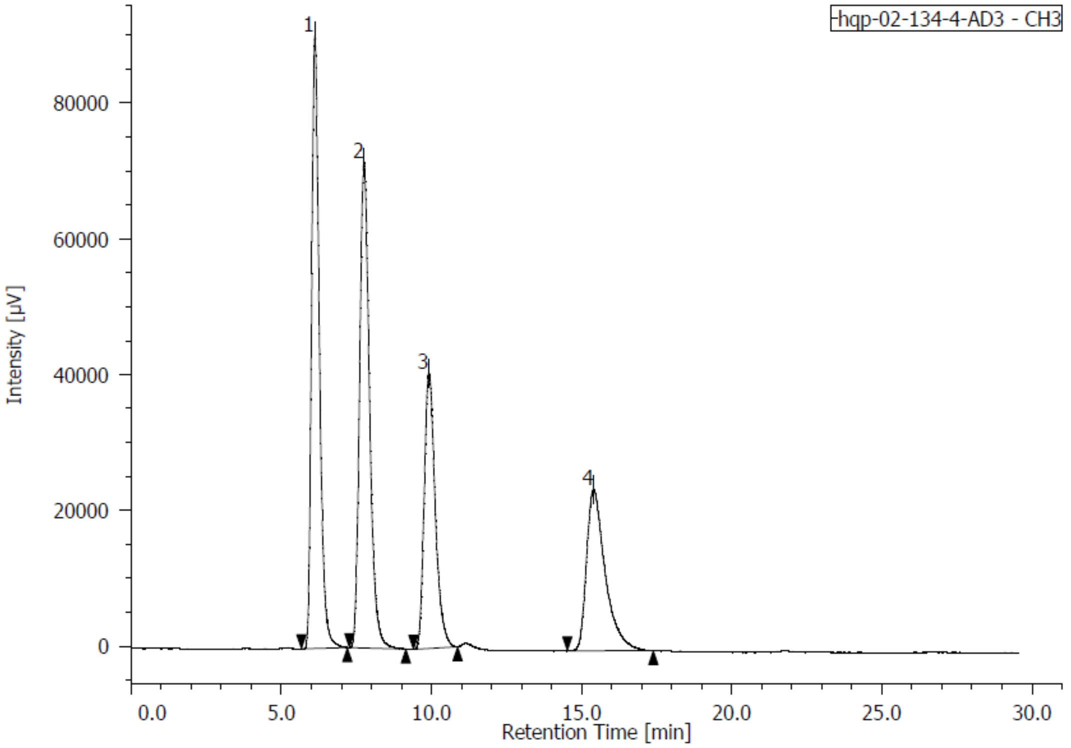

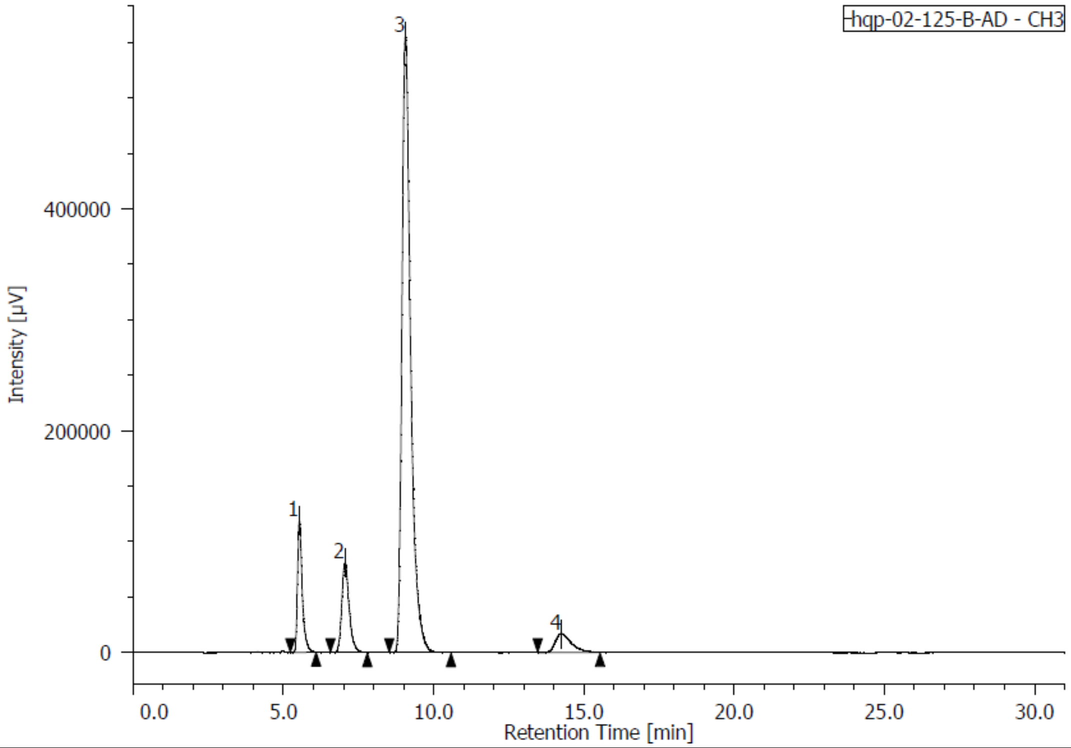

**Peak**	**1**	**2**	**3**	**4**
Retention Time (min)	5.53	7.05	9.06	14.3
Area (%)	9.0	9.5	77.1	4.4

Compound **4la**

HPLC conditions: DAICEL Chiralpak AD-3 column, hexane/*i*-PrOH = 70/30, 254 nm, 1.0 mL/min, 40 °C, t_{major} = 7.29 min, t_{minor} = 10.62 min;

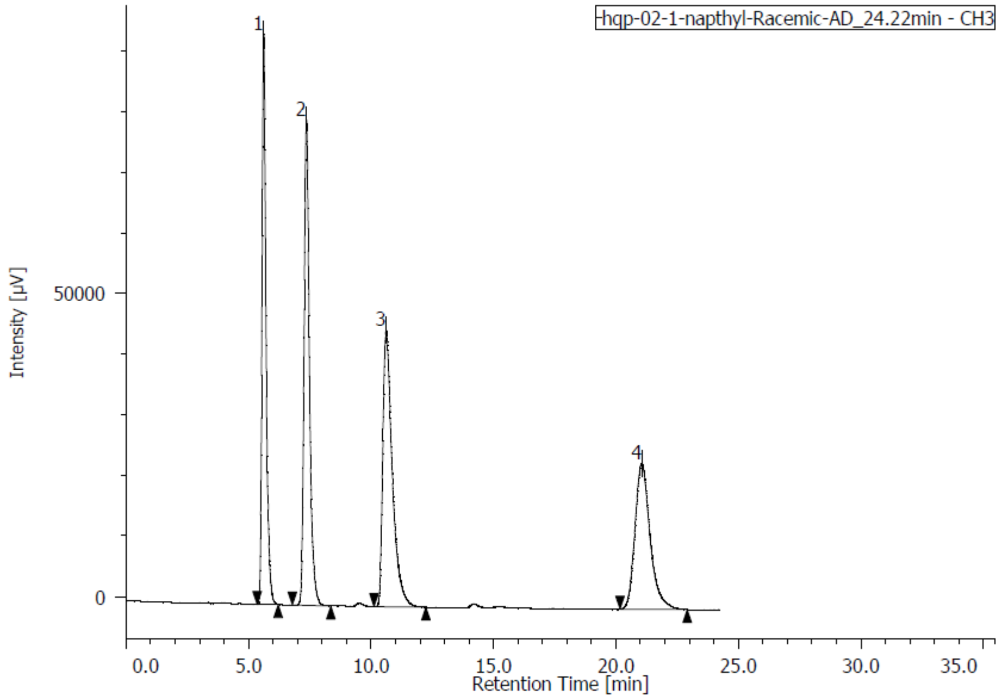

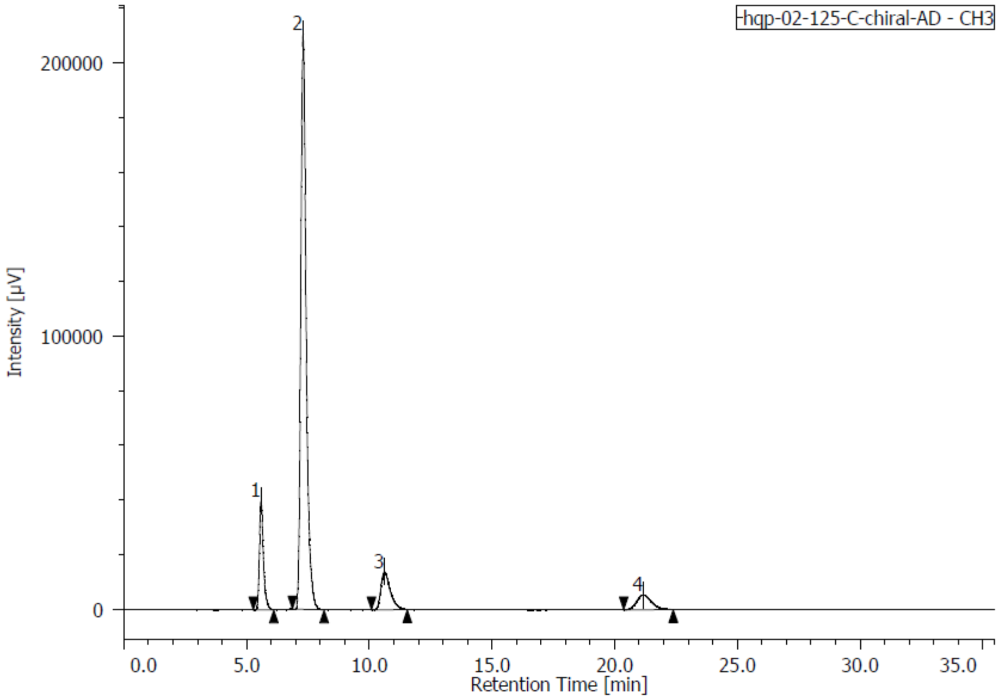

**Peak**	**1**	**2**	**3**	**4**
Retention Time (min)	5.59	7.29	10.62	21.16
Area (%)	10.5	75.5	8.6	6.4

Compound **4ma**

HPLC conditions: DAICEL Chiralpak IA-3 column, hexane/*i*-PrOH = 70/30, 254 nm, 0.5 mL/min, 30 °C, t_{major} = 18.38 min, t_{minor} = 25.98 min;

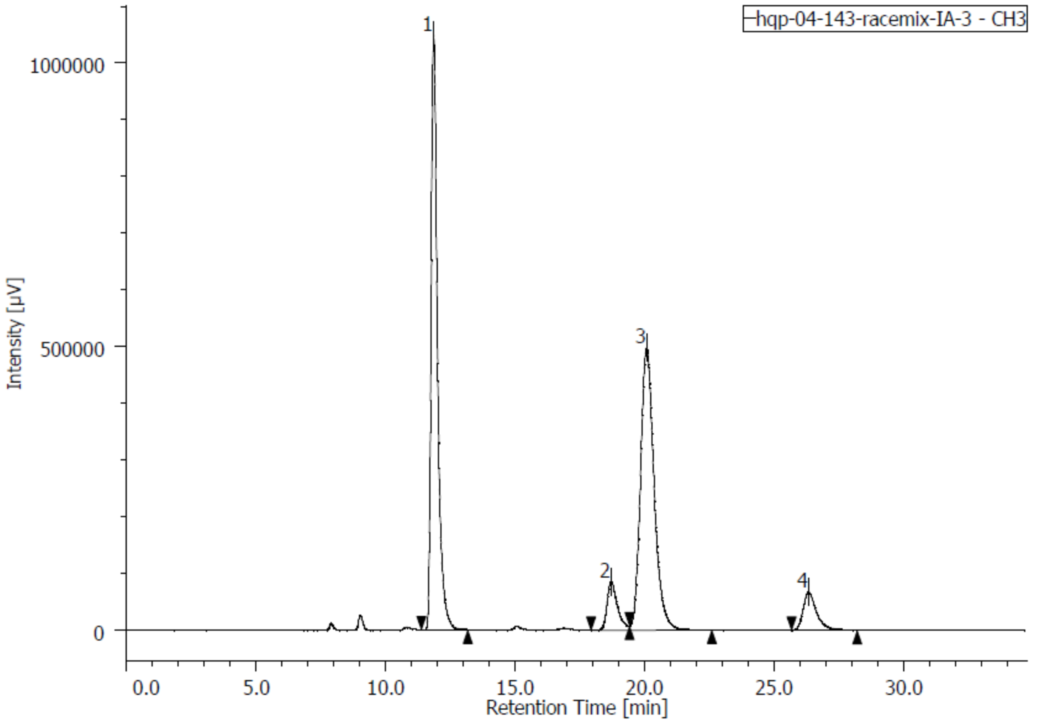

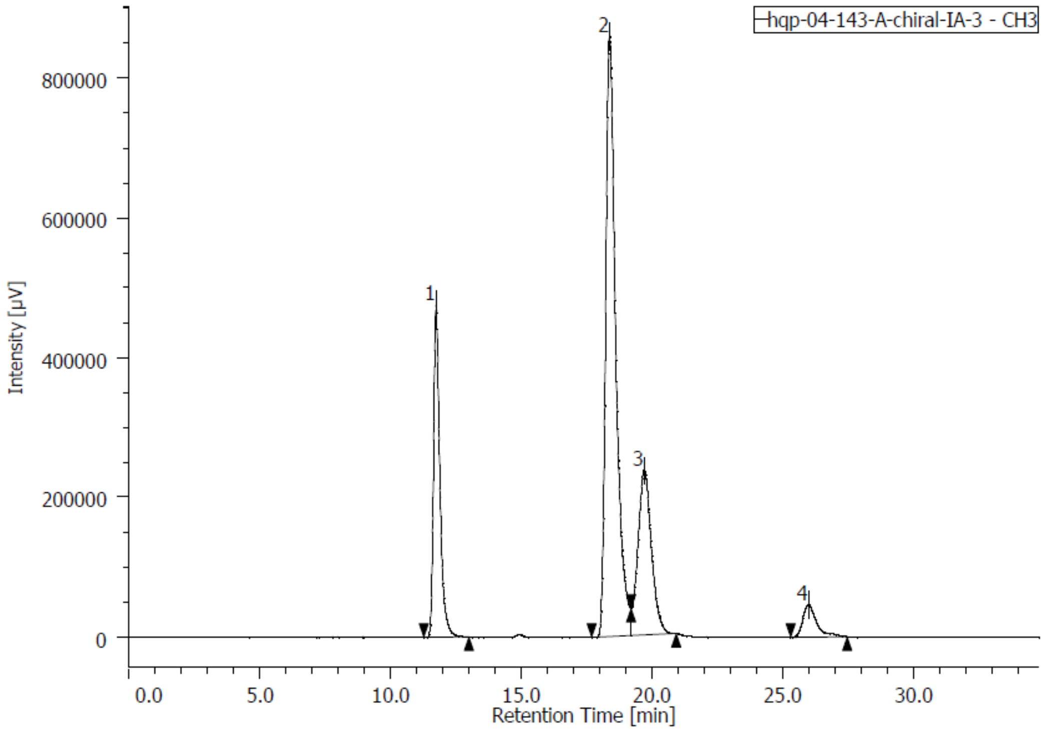

**Peak**	**1**	**2**	**3**	**4**
Retention Time (min)	11.74	18.38	19.70	25.98
Area (%)	19.4	55.9	20.6	4.2

Compound **4na**

HPLC conditions: DAICEL Chiralpak AD-3 column, hexane/*i*-PrOH = 70/30, 254 nm, 1.0 mL/min, 40 °C, t_{major} = 5.64 min, t_{minor} = 7.73 min;

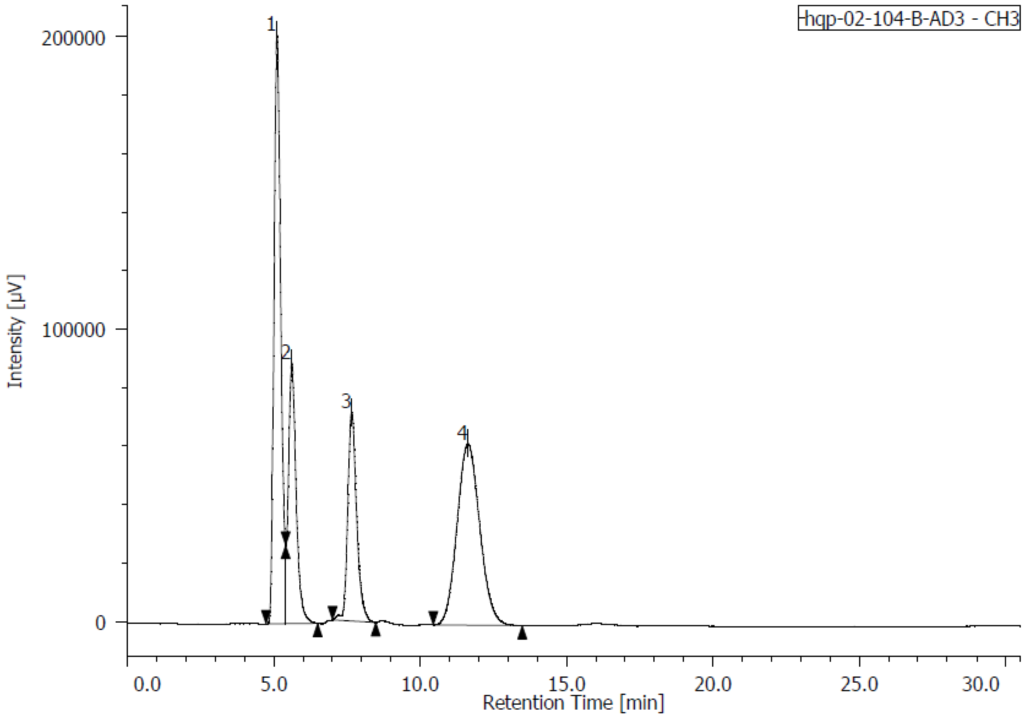

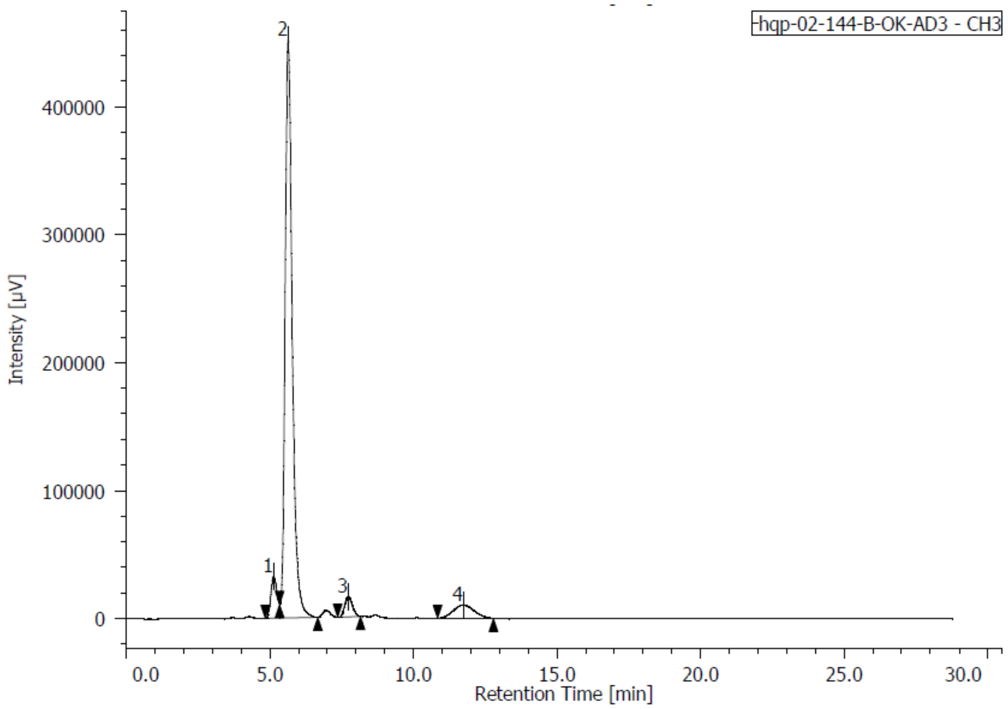

**Peak**	**1**	**2**	**3**	**4**
Retention Time (min)	5.13	5.64	7.73	11.74
Area (%)	5.4	85.5	3.2	5.9

Compound **4ab**

HPLC conditions: DAICEL Chiralpak AD-3 column, hexane/*i*-PrOH = 70/30, 254 nm, 1.0 mL/min, 40 °C, t_{major} = 6.23 min, t_{minor} = 8.78 min;

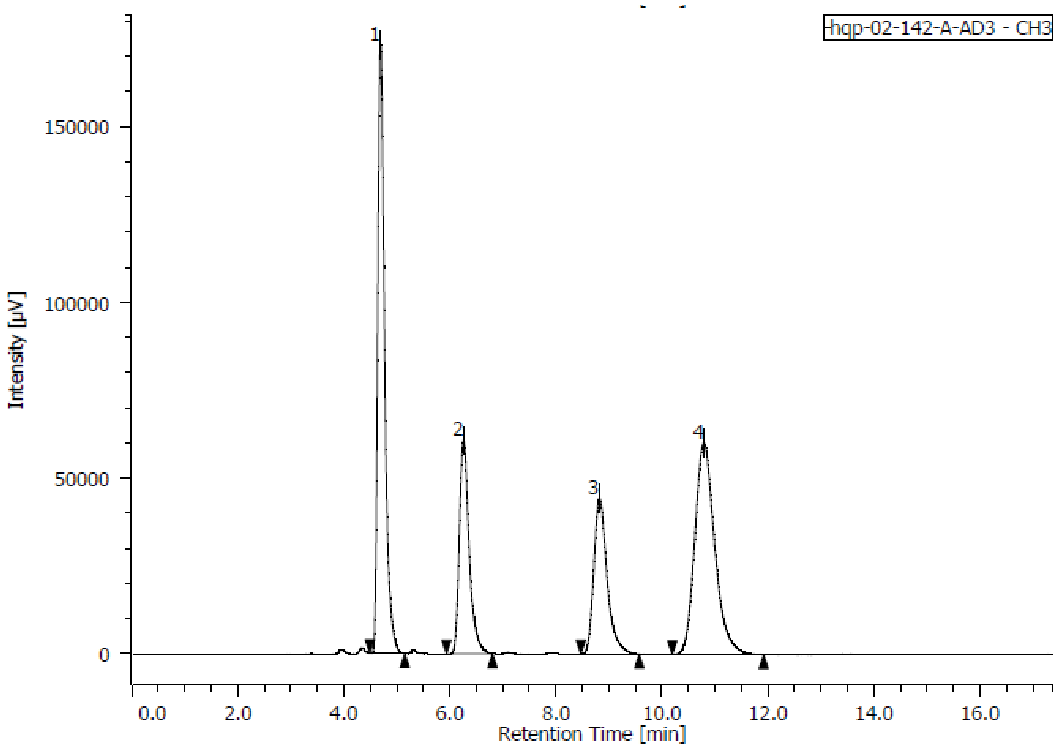

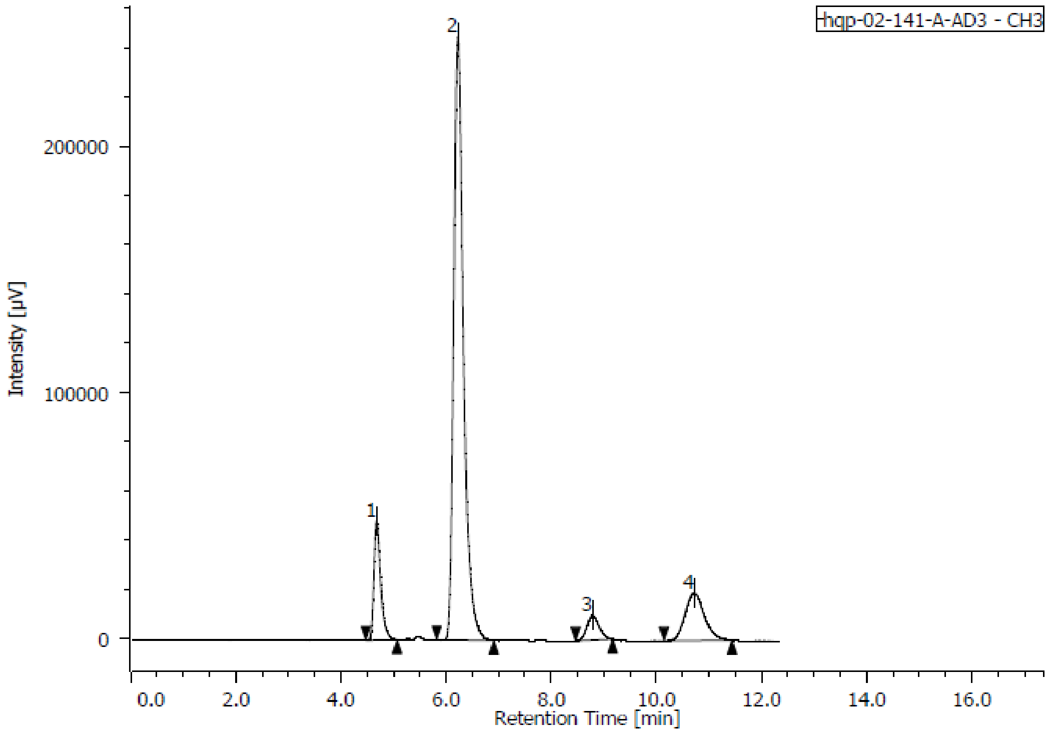

**Peak**	**1**	**2**	**3**	**4**
Retention Time (min)	4.68	6.23	8.78	10.72
Area (%)	10.3	74.3	3.8	11.6

Compound **4ac**

HPLC conditions: DAICEL Chiralpak AD-3 column, hexane/*i*-PrOH = 70/30, 254 nm, 1.0 mL/min, 40 °C, t_{major} = 5.13 min, t_{minor} = 6.78 min;

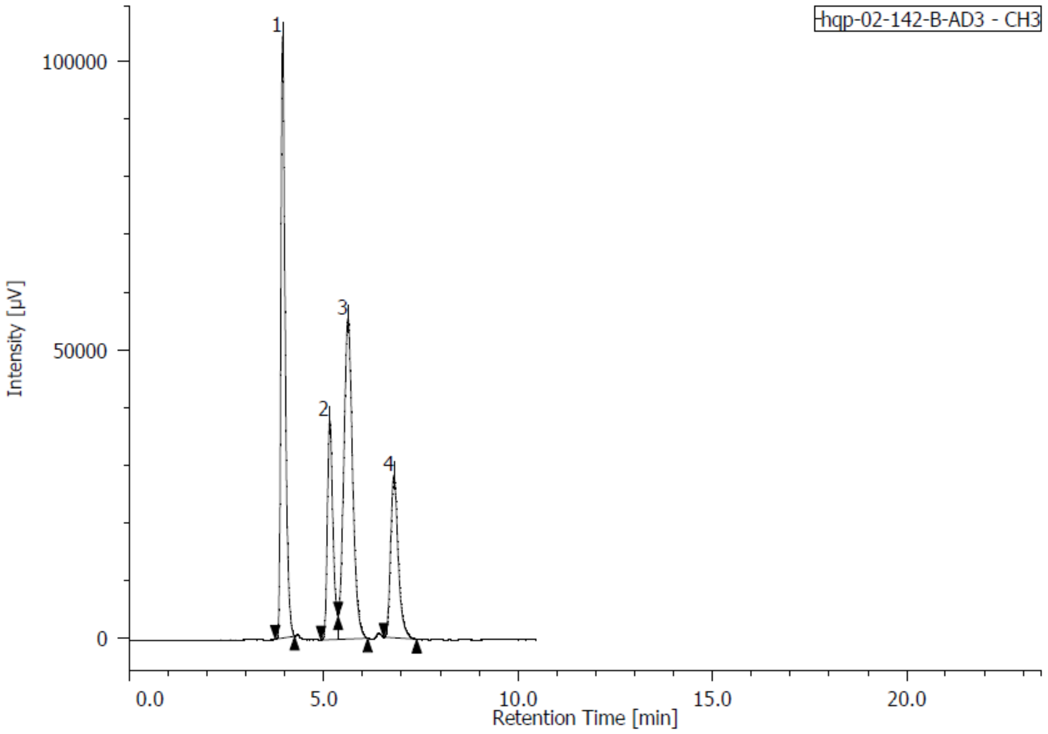

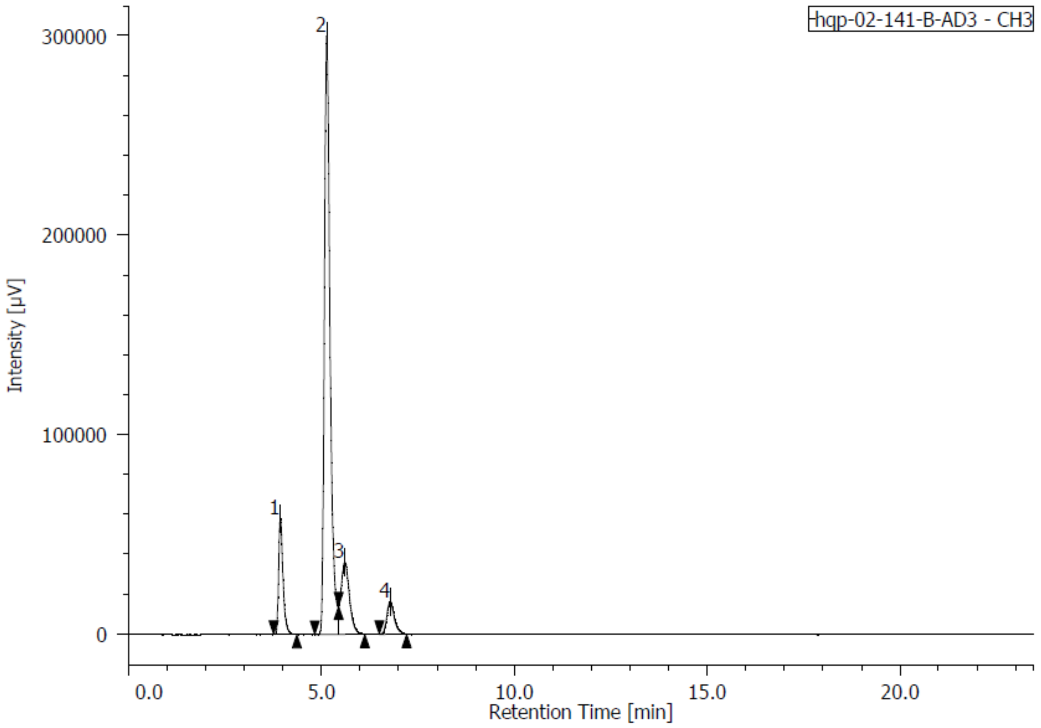

**Peak**	**1**	**2**	**3**	**4**
Retention Time (min)	3.93	5.13	5.61	6.78
Area (%)	10.6	71.5	12.8	5.1

Compound **4ad**

HPLC conditions: DAICEL Chiralpak AD-3 column, hexane/*i*-PrOH = 70/30, 254 nm, 1.0 mL/min, 40 °C, t_{major} = 5.83 min, t_{minor} = 11.57 min;

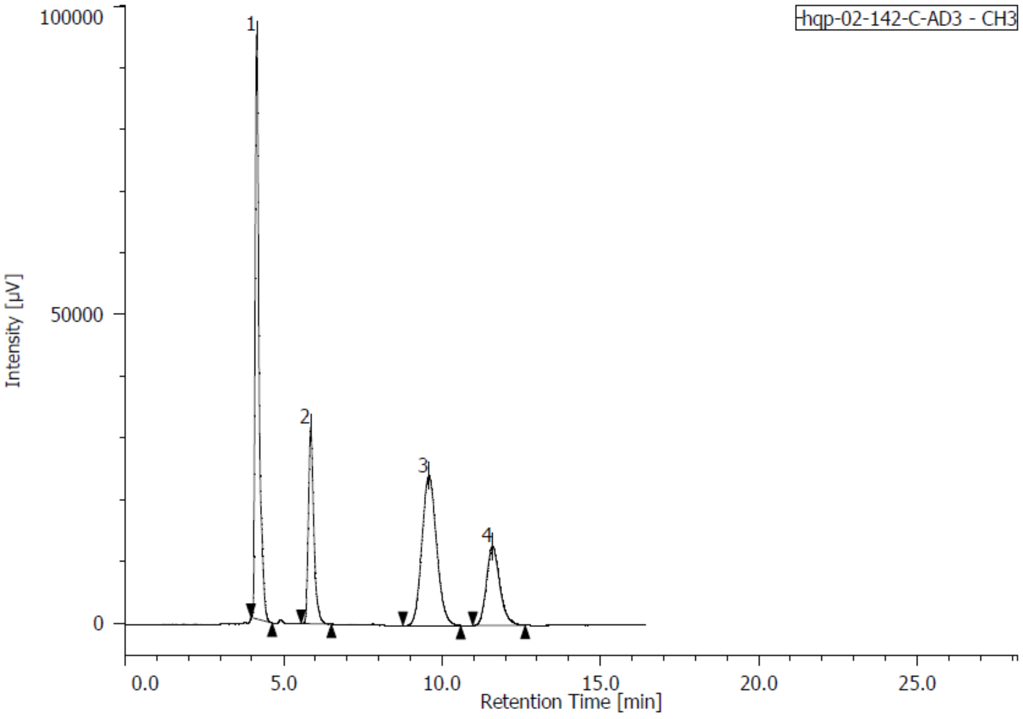

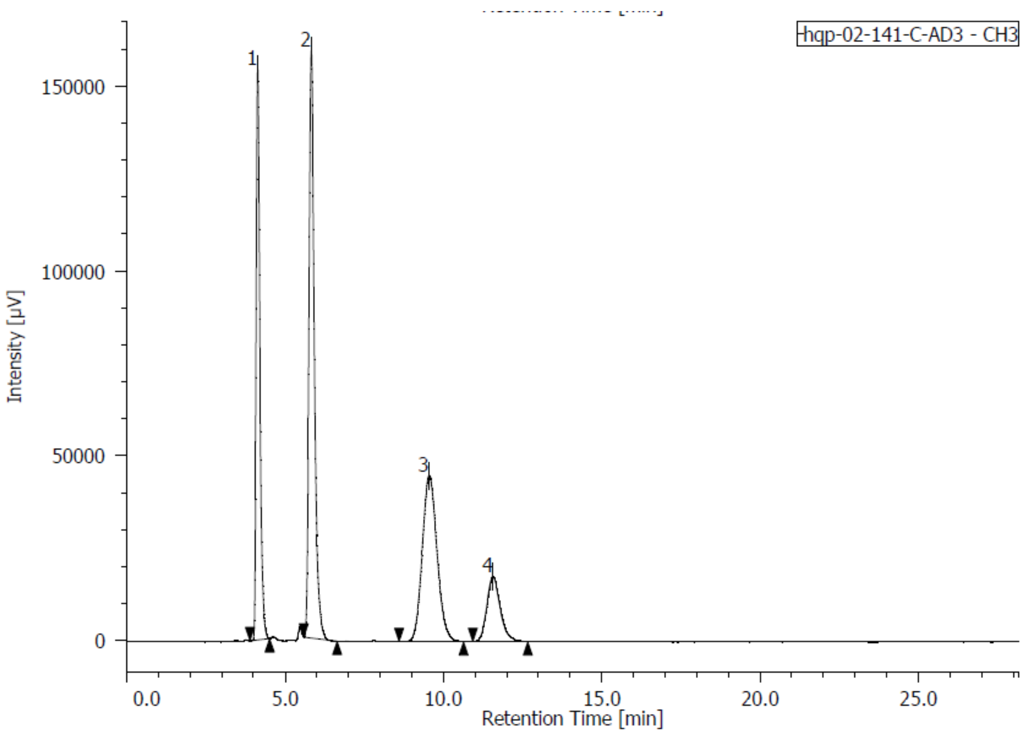

**Peak**	**1**	**2**	**3**	**4**
Retention Time (min)	4.14	5.83	9.55	11.57
Area (%)	24.7	36.6	28.6	10.1

Compound **4ae**

HPLC conditions: DAICEL Chiralpak AD-3 column, hexane/*i*-PrOH = 70/30, 254 nm, 1.0 mL/min, 40 °C, t_{major} = 7.50 min, t_{minor} = 13.00 min;

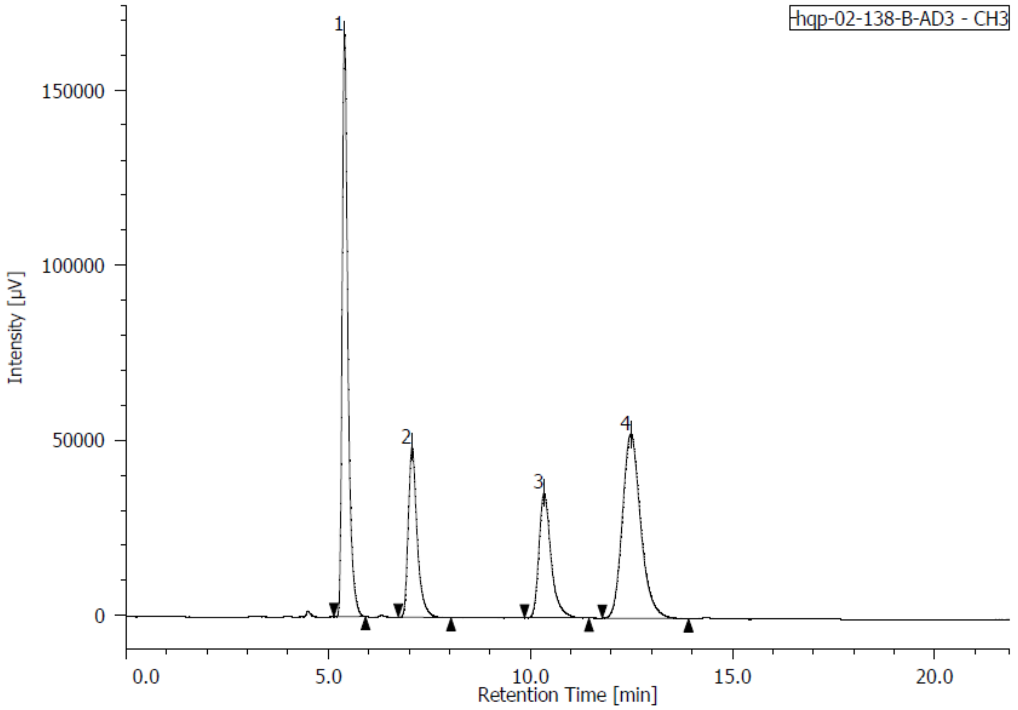

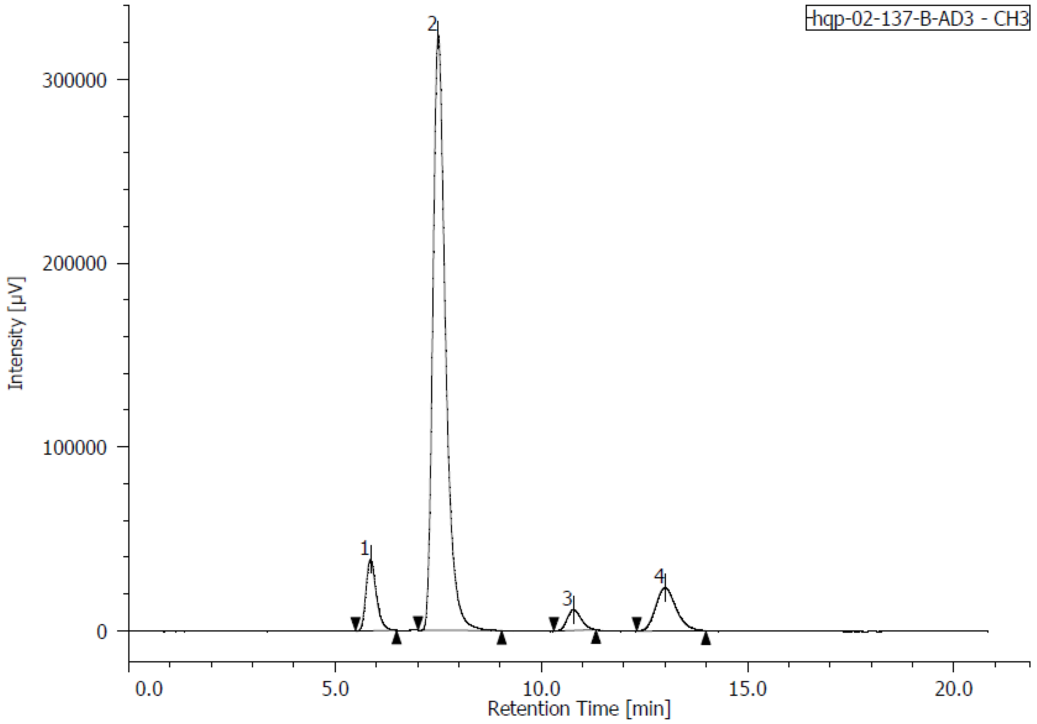

**Peak**	**1**	**2**	**3**	**4**
Retention Time (min)	5.86	7.50	10.78	13.00
Area (%)	7.8	79.7	3.1	9.4

Compound **4af**

HPLC conditions: DAICEL Chiralpak AD-3 column, hexane/*i*-PrOH = 70/30, 254 nm, 1.0 mL/min, 40 ℃, t_{major} = 6.59 min, t_{minor} = 11.52 min;

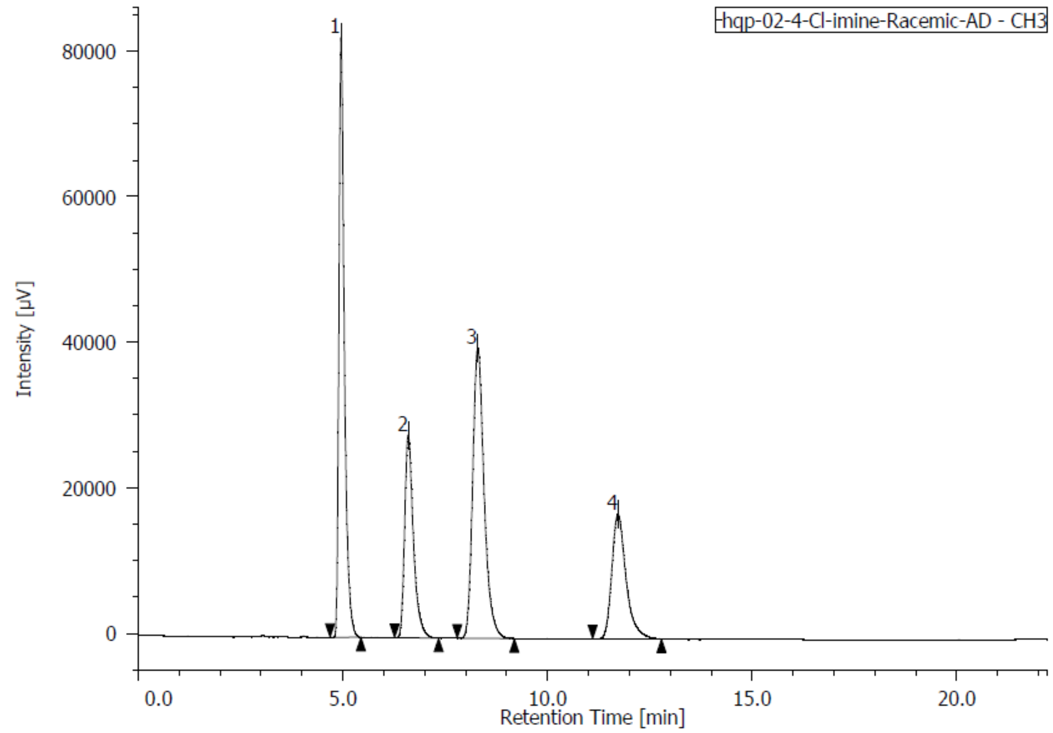

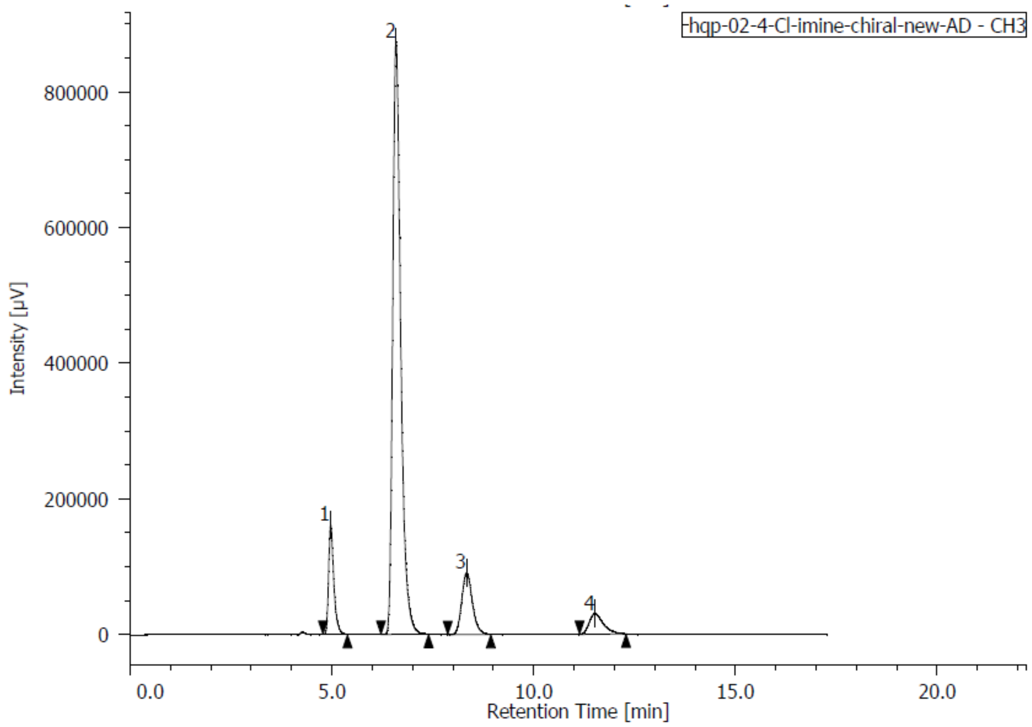

**Peak**	**1**	**2**	**3**	**4**
Retention Time (min)	4.97	6.59	8.33	11.52
Area (%)	9.1	75.7	10.5	4.7

Compound **4ag**

HPLC conditions: DAICEL Chiralpak AD-3 column, hexane/*i*-PrOH = 70/30, 254 nm, 1.0 mL/min, 40 °C, t_{major} = 7.25 min, t_{minor} = 10.42 min;

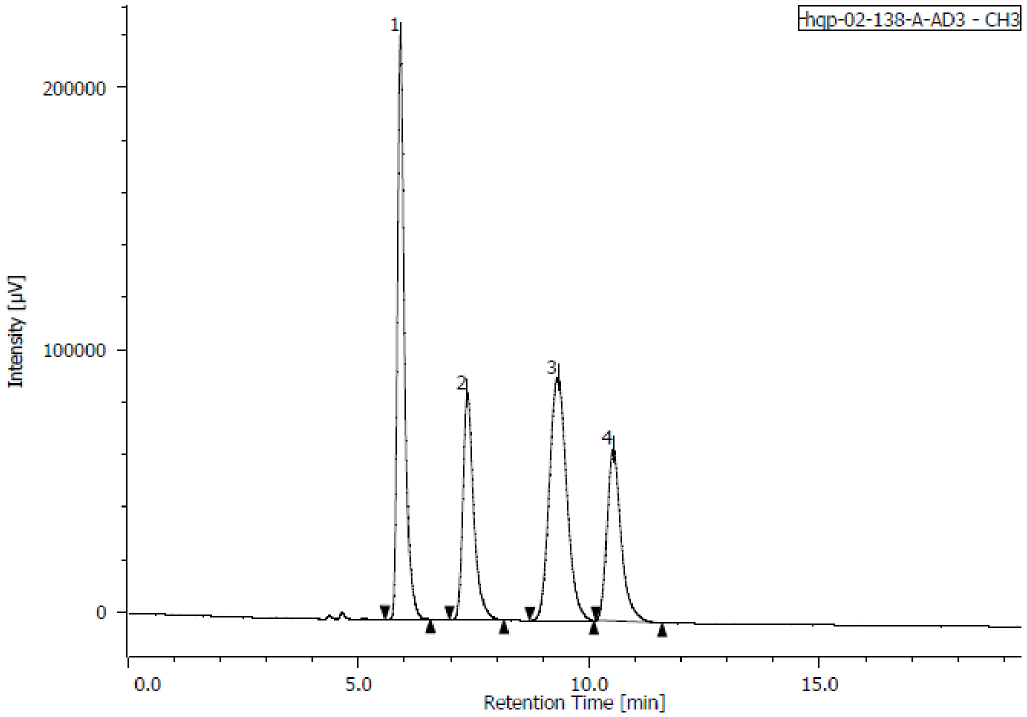

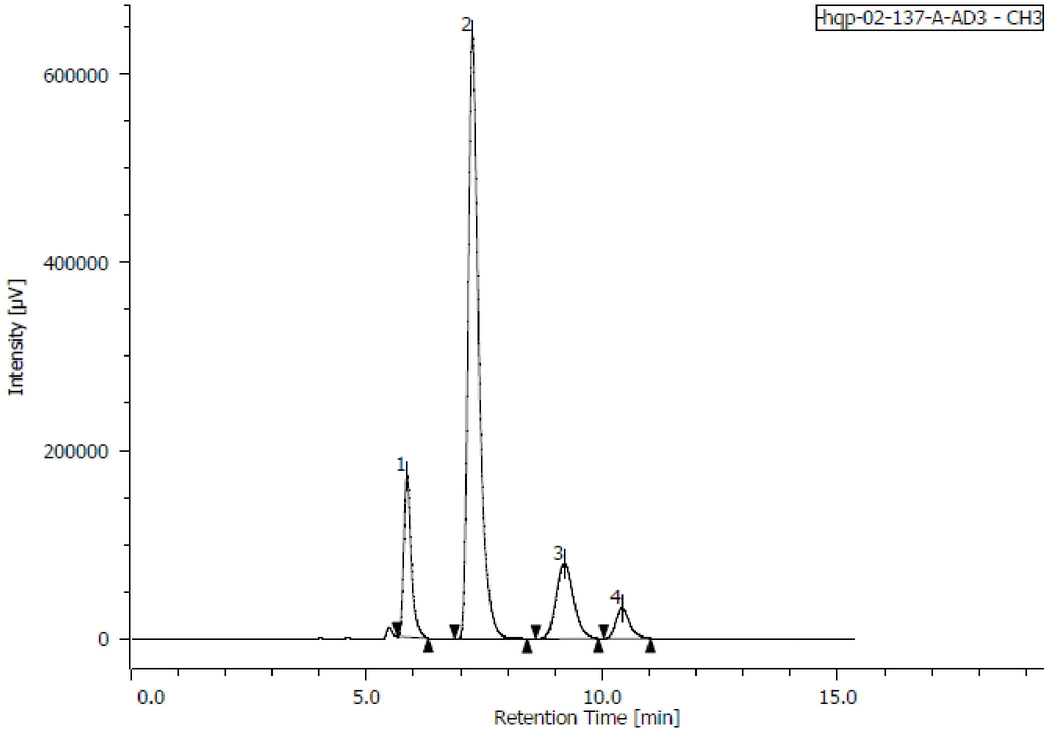

**Peak**	**1**	**2**	**3**	**4**
Retention Time (min)	5.86	7.25	9.20	10.42
Area (%)	12.7	69.2	13.7	4.4

Compound **4ah**

HPLC conditions: DAICEL Chiralpak OD-3 column, hexane/*i*-PrOH = 90/10, 254 nm, 1.0 mL/min, 30 °C, t_{major} = 11.59 min, t_{minor} = 14.65 min;

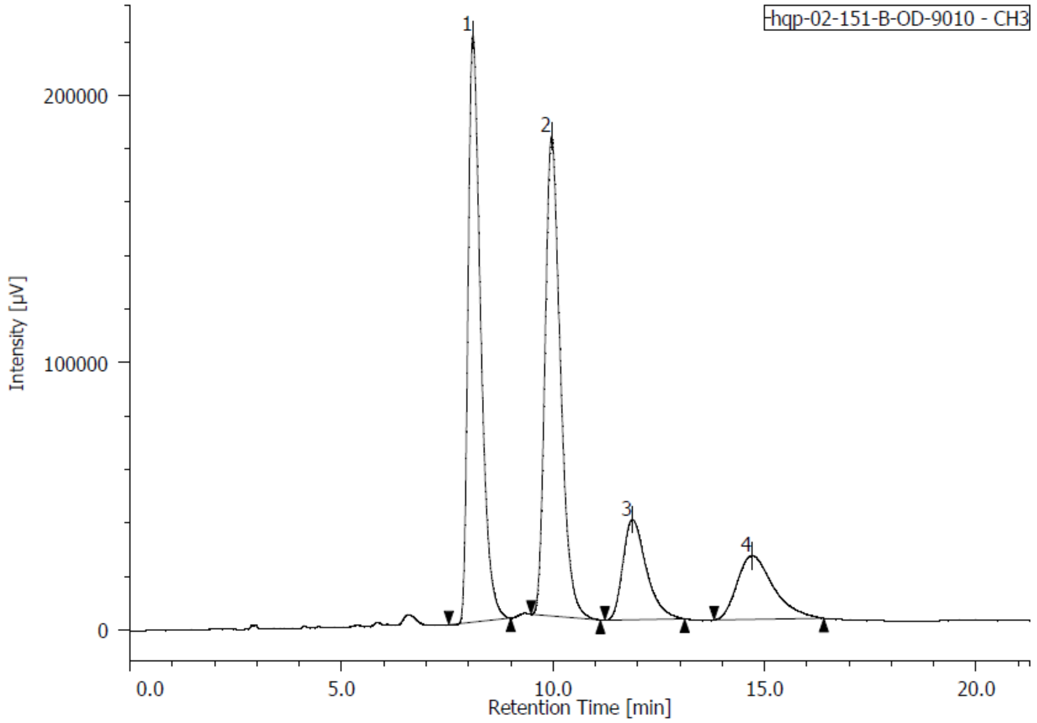

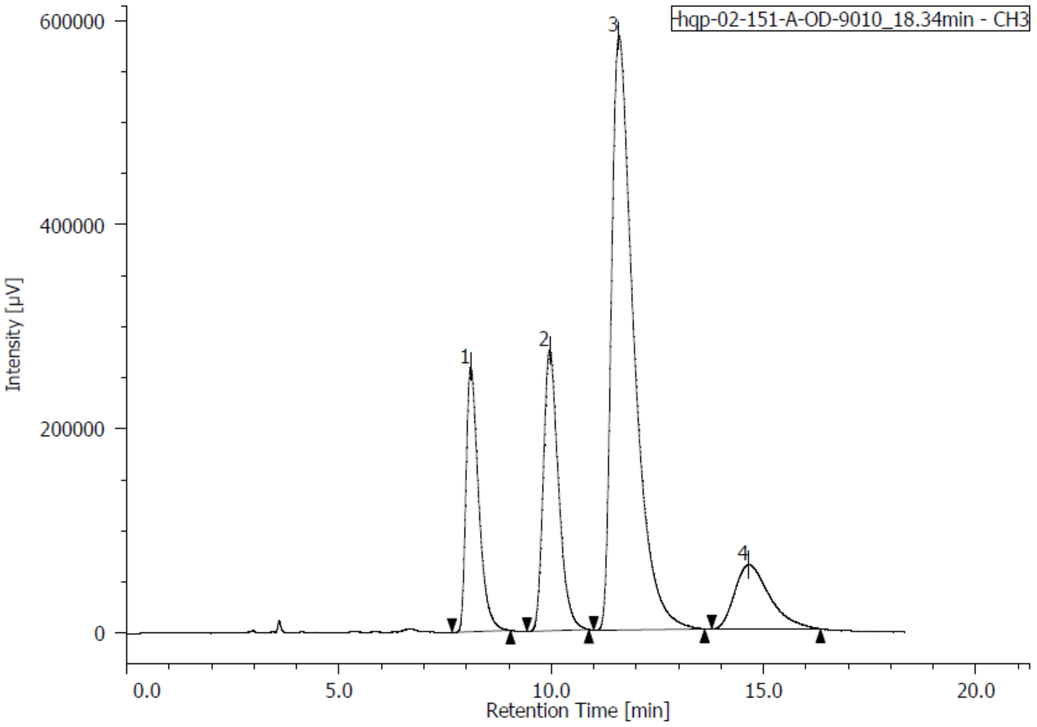

**Peak**	**1**	**2**	**3**	**4**
Retention Time (min)	8.10	9.97	11.59	14.65
Area (%)	14.4	18.3	57.5	9.7

Compound **4oa**

HPLC conditions: DAICEL Chiralpak IA-3 column, hexane/*i*-PrOH = 70/30, 254 nm, 1.0 mL/min, 40 °C, t_{major} = 6.29 min, t_{minor} = 7.48 min;

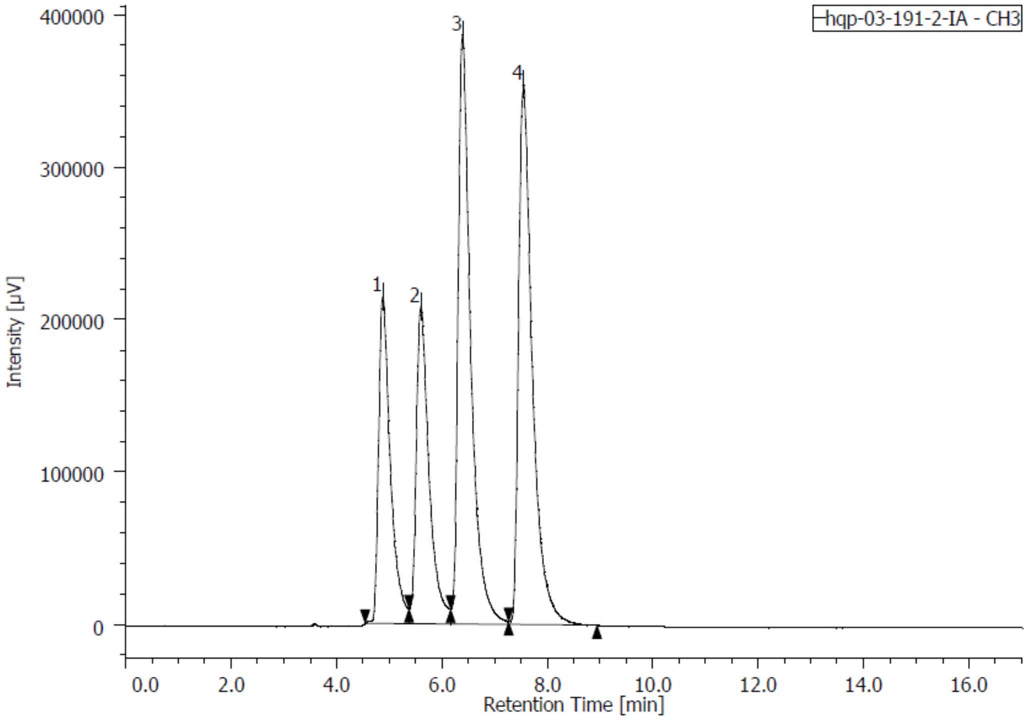

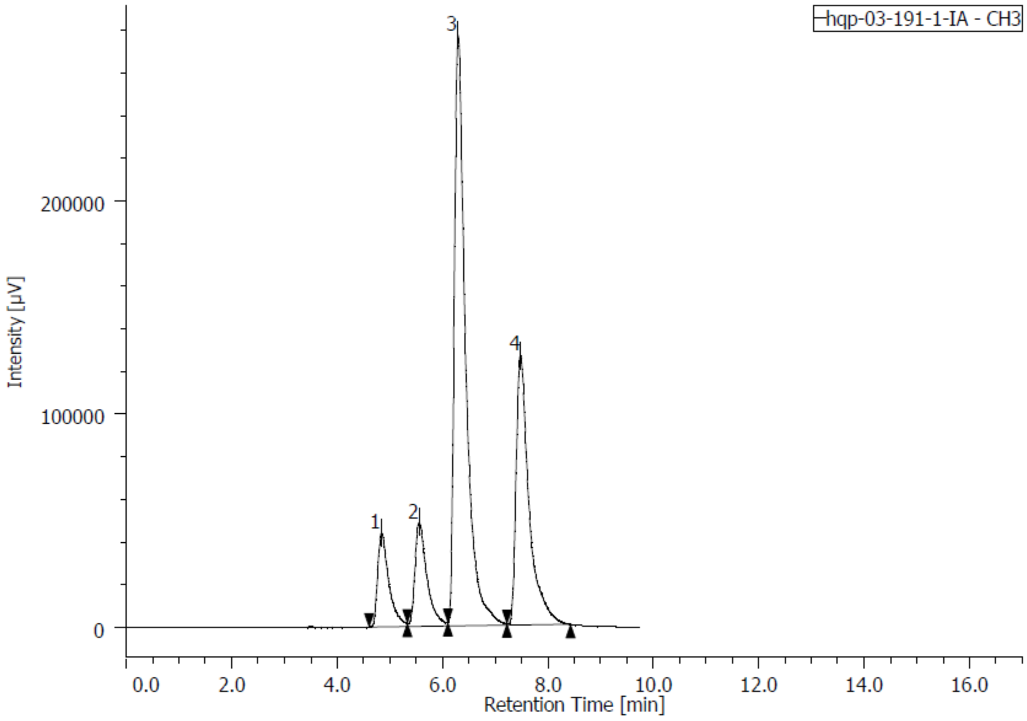

**Peak**	**1**	**2**	**3**	**4**
Retention Time (min)	4.84	5.55	6.29	7.48
Area (%)	8.5	9.9	53.8	27.8

Compound **5**

HPLC conditions: DAICEL Chiralpak AD-3 column, hexane/*i*-PrOH = 70/30, 254 nm, 1.0 mL/min, 40 °C, t_{major} = 7.46 min, t_{minor} = 7.06 min;

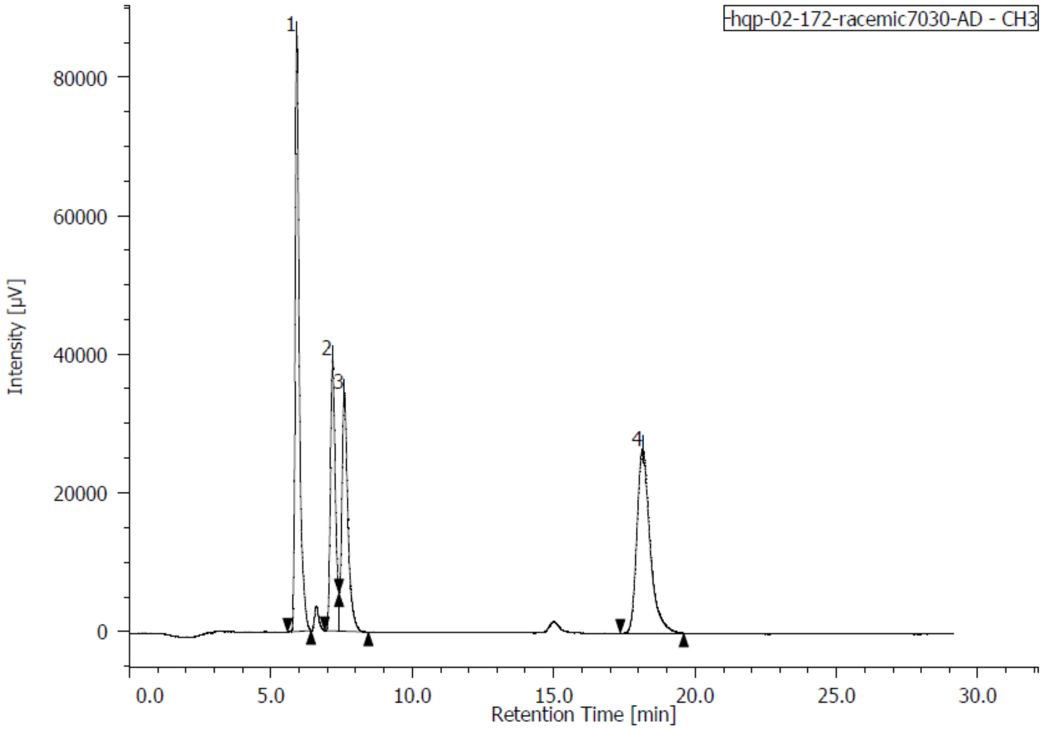

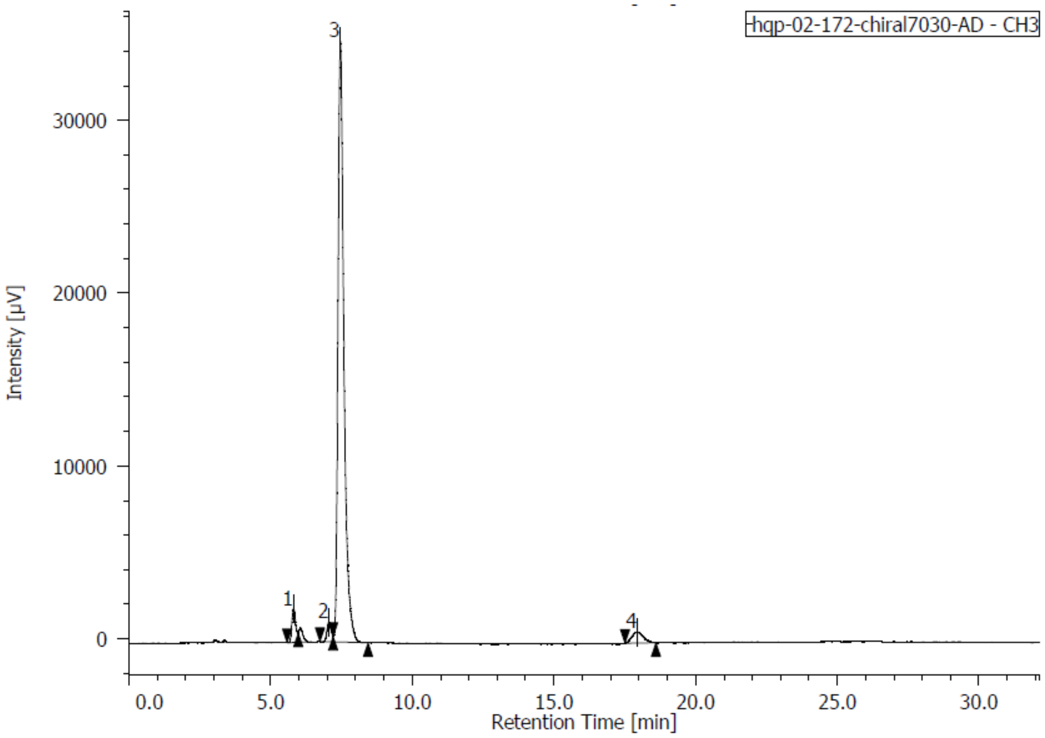

**Peak**	**1**	**2**	**3**	**4**
Retention Time (min)	5.81	7.06	7.46	17.9
Area (%)	3.3	2.2	91.3	3.2

8. NMR Spectra

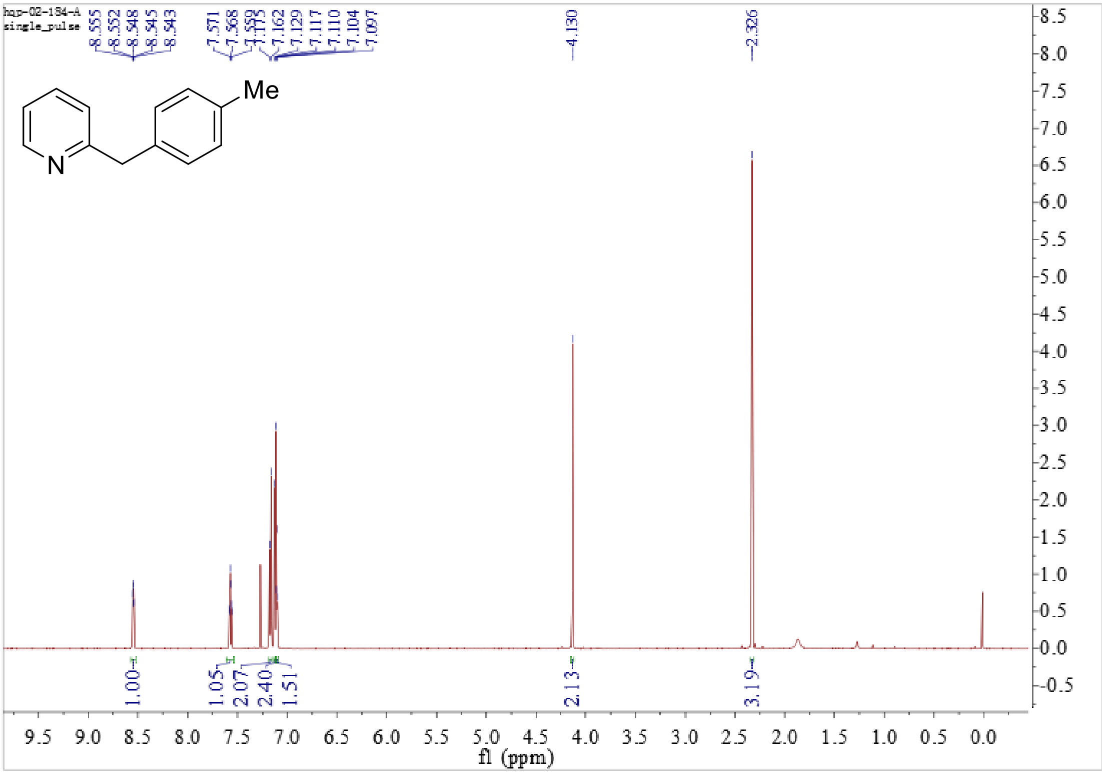

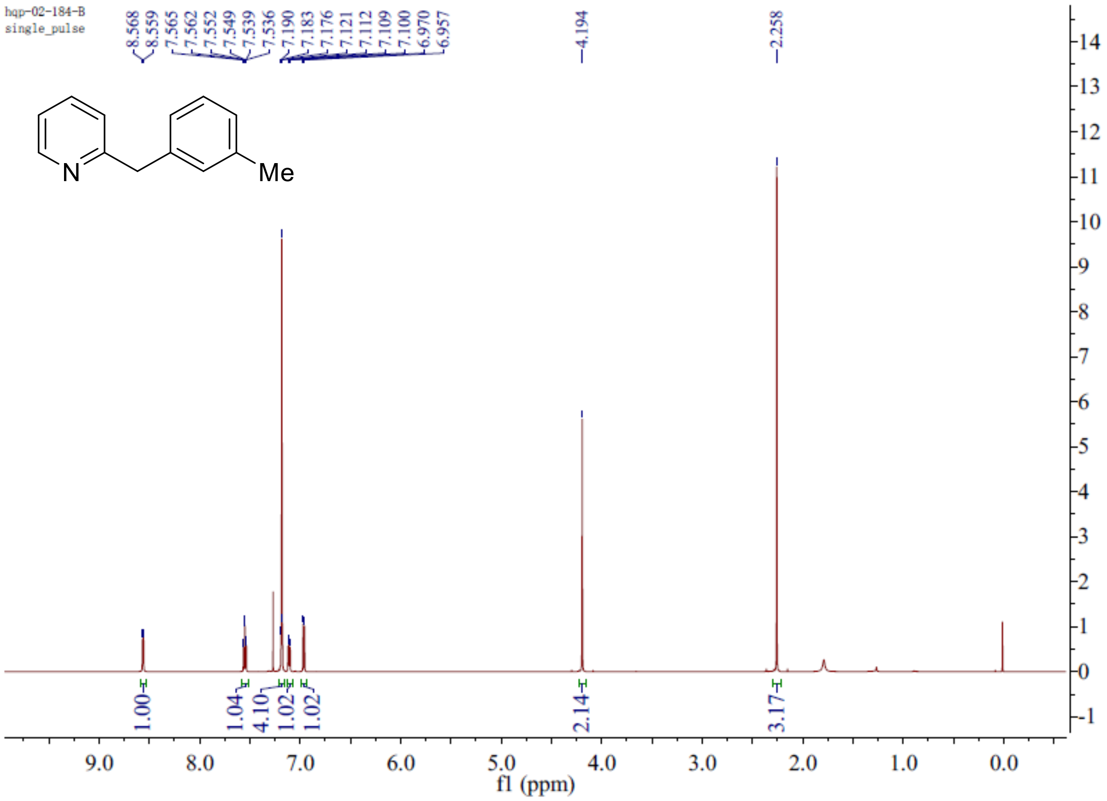

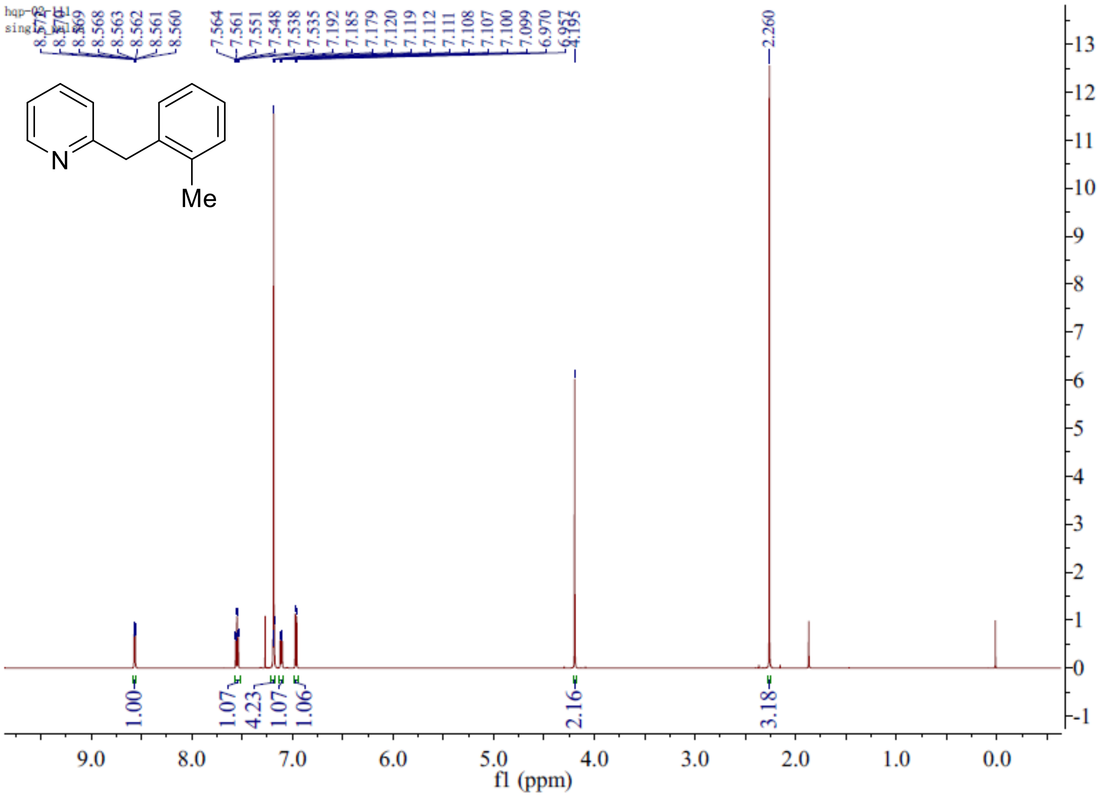

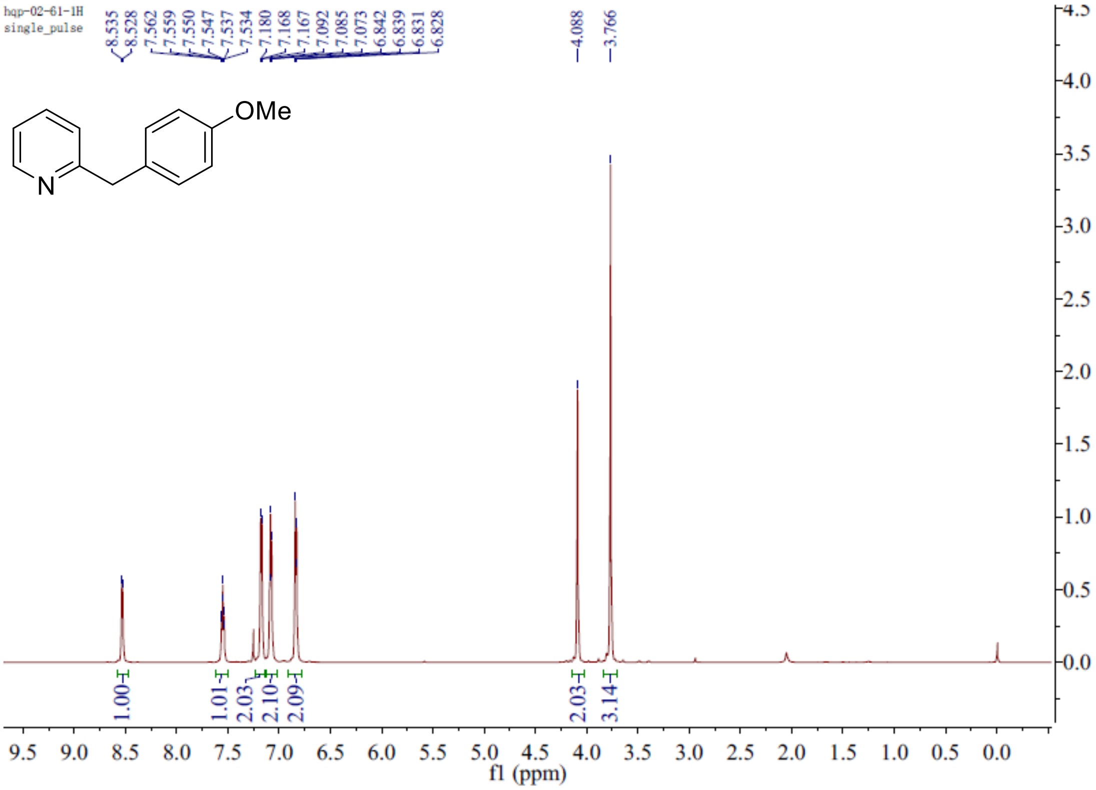

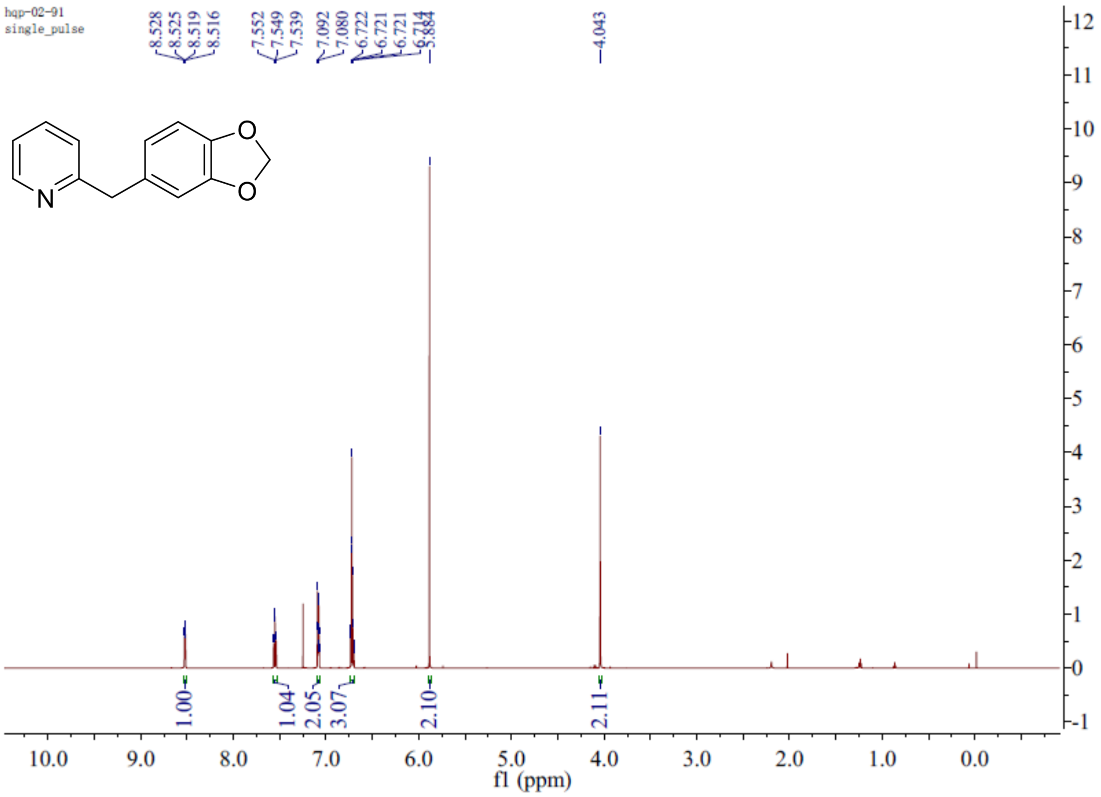
hqp-02-91
single_pulse
8.528
8.525
8.519
8.516
7.552
7.549
7.539
7.092
7.080
6.722
6.721
6.721
6.714
5.884
4.043
N
O
O
1.00
1.04
2.05
3.07
2.10
2.11
f1 (ppm)

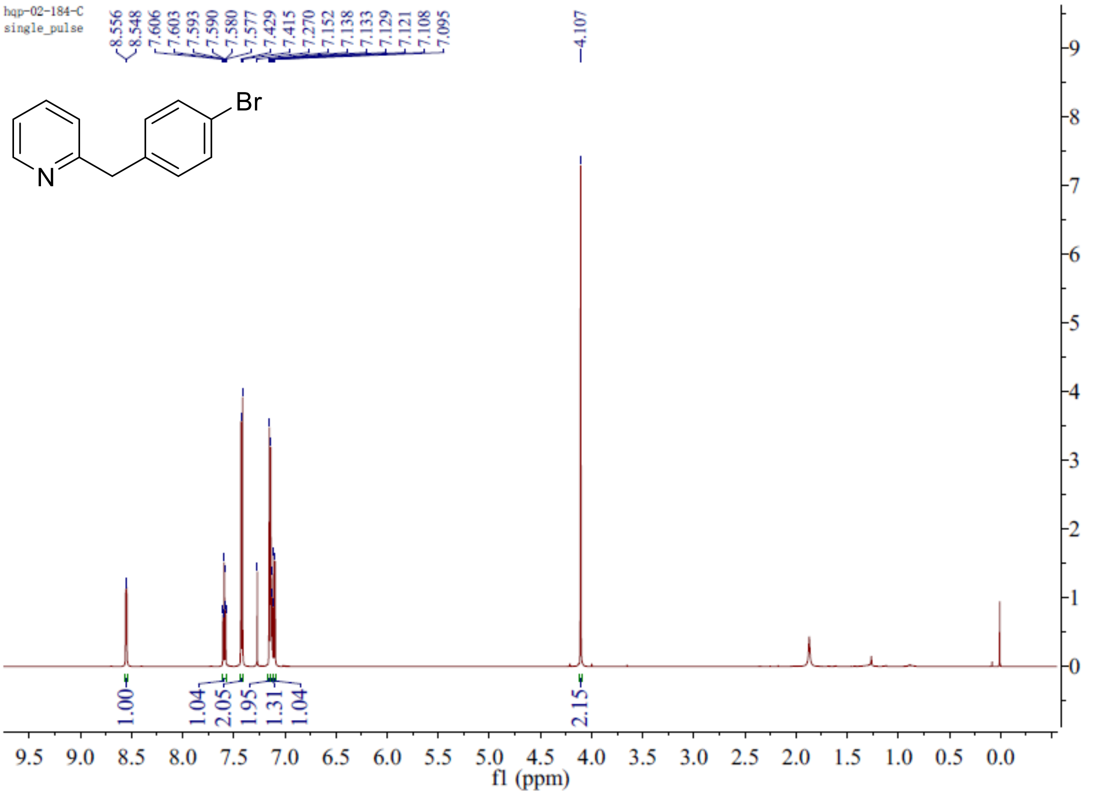
hqp-02-184-C
single_pulse
8.556
8.548
7.606
7.603
7.593
7.590
7.580
7.577
7.429
7.415
7.270
7.152
7.138
7.133
7.129
7.121
7.108
7.095
4.107
Br
N
1.00
1.04
2.05
1.95
1.31
1.04
2.15
f1 (ppm)

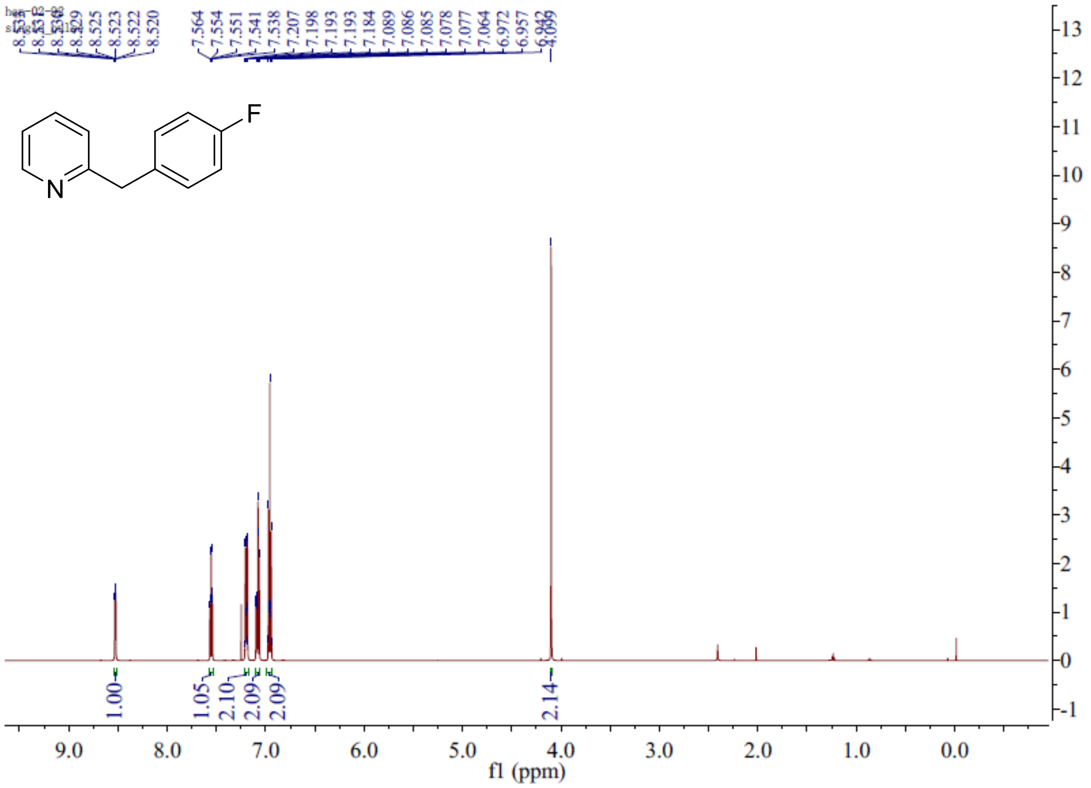

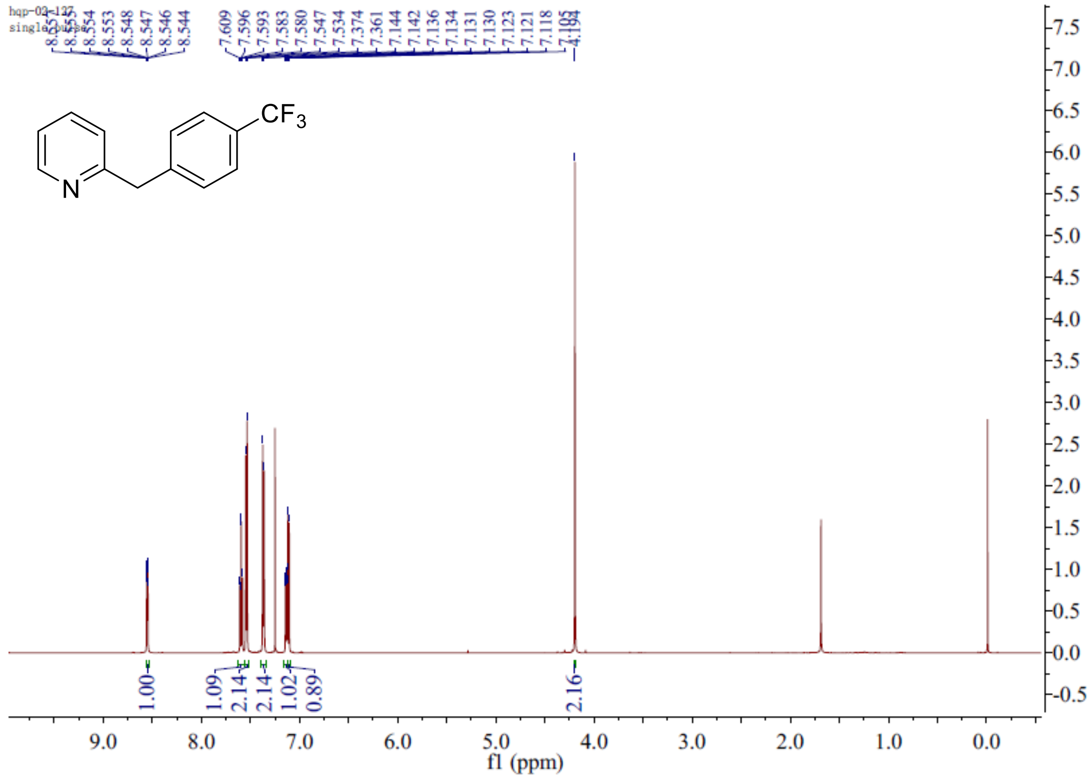

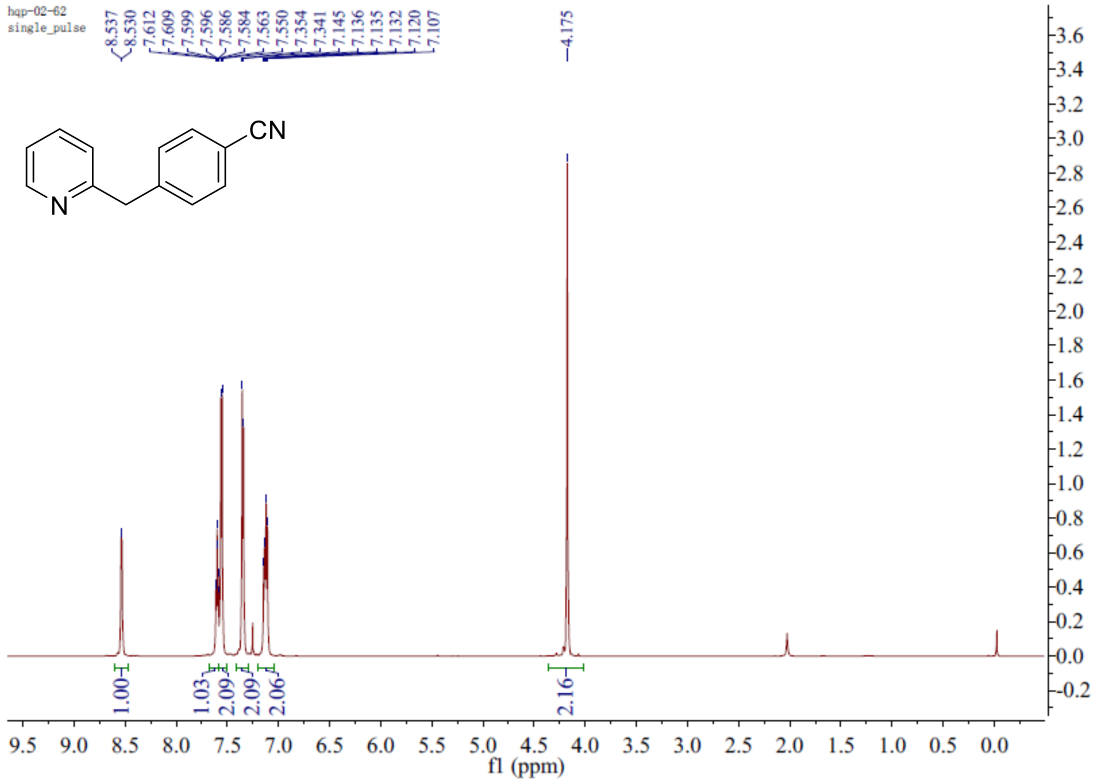
hqp-02-62
single_pulse
8.537
8.530
7.612
7.609
7.599
7.596
7.586
7.584
7.563
7.550
7.354
7.341
7.145
7.136
7.135
7.132
7.120
7.107
4.175
CN
N
1.00
1.03
2.09
2.09
2.06
2.16
f1 (ppm)

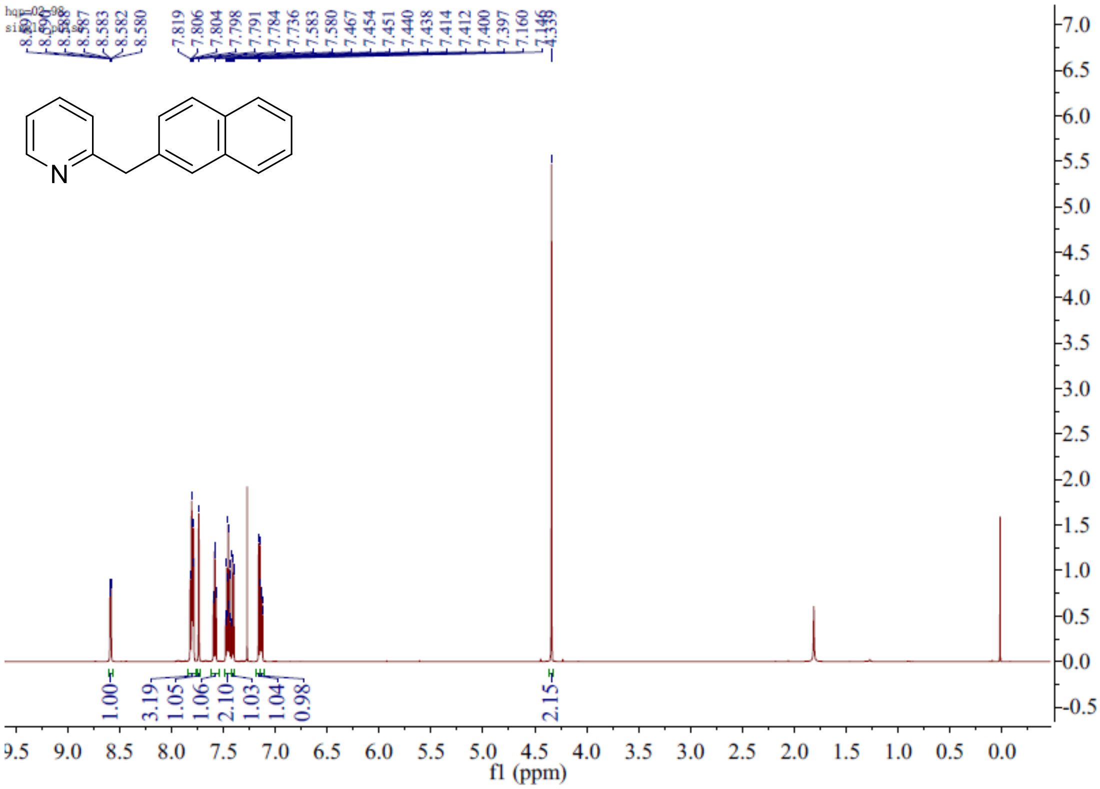
8.587
8.583
8.582
8.580
7.819
7.806
7.804
7.798
7.791
7.784
7.736
7.583
7.580
7.467
7.454
7.451
7.440
7.438
7.414
7.412
7.400
7.397
7.160
N
1.00
3.19
1.05
1.06
2.10
1.03
1.04
0.98
2.15
f1 (ppm)

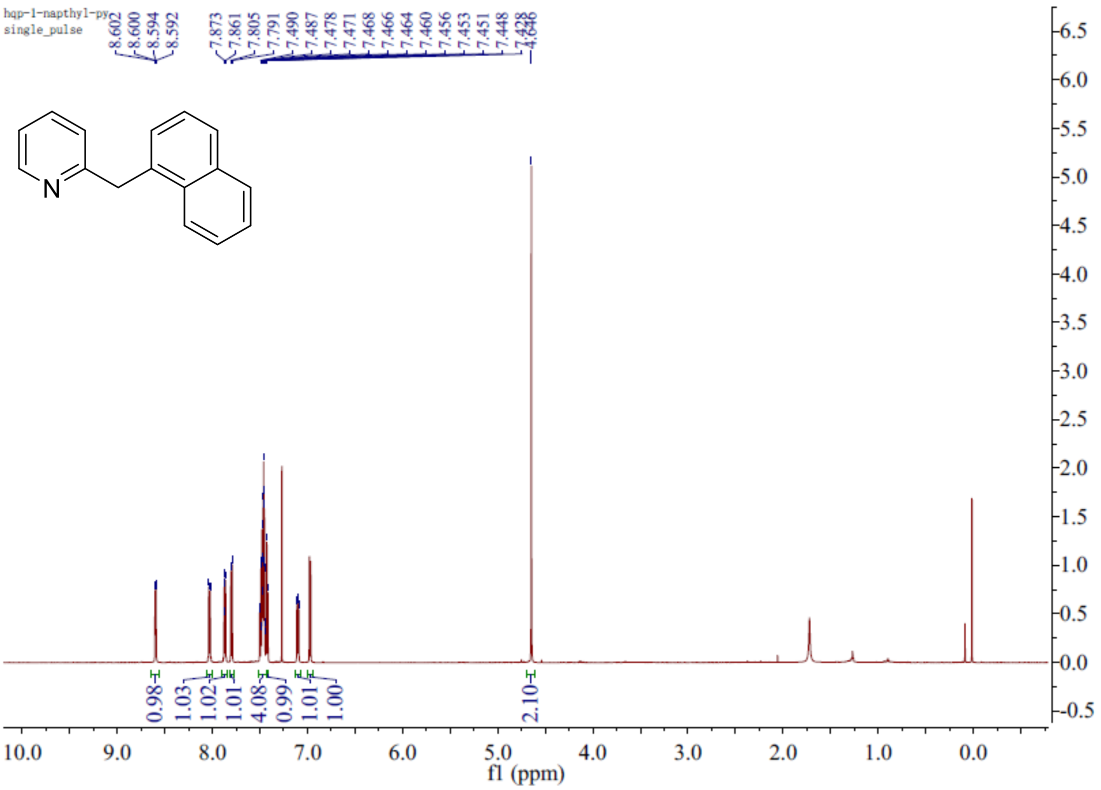

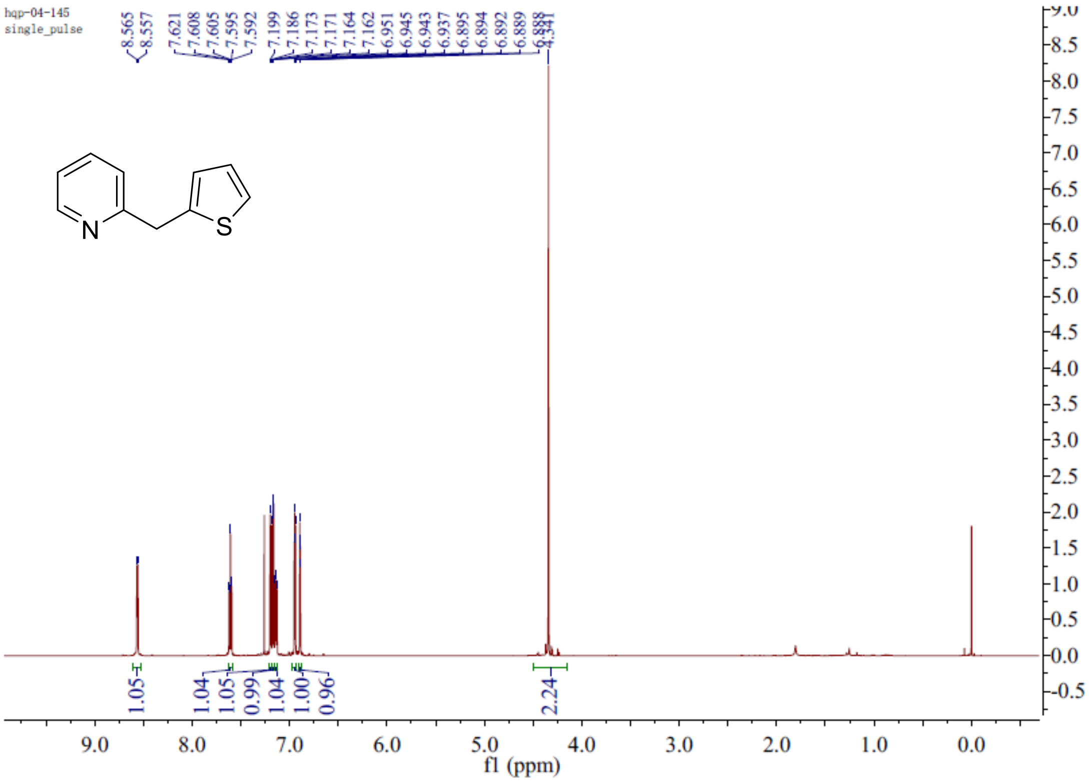

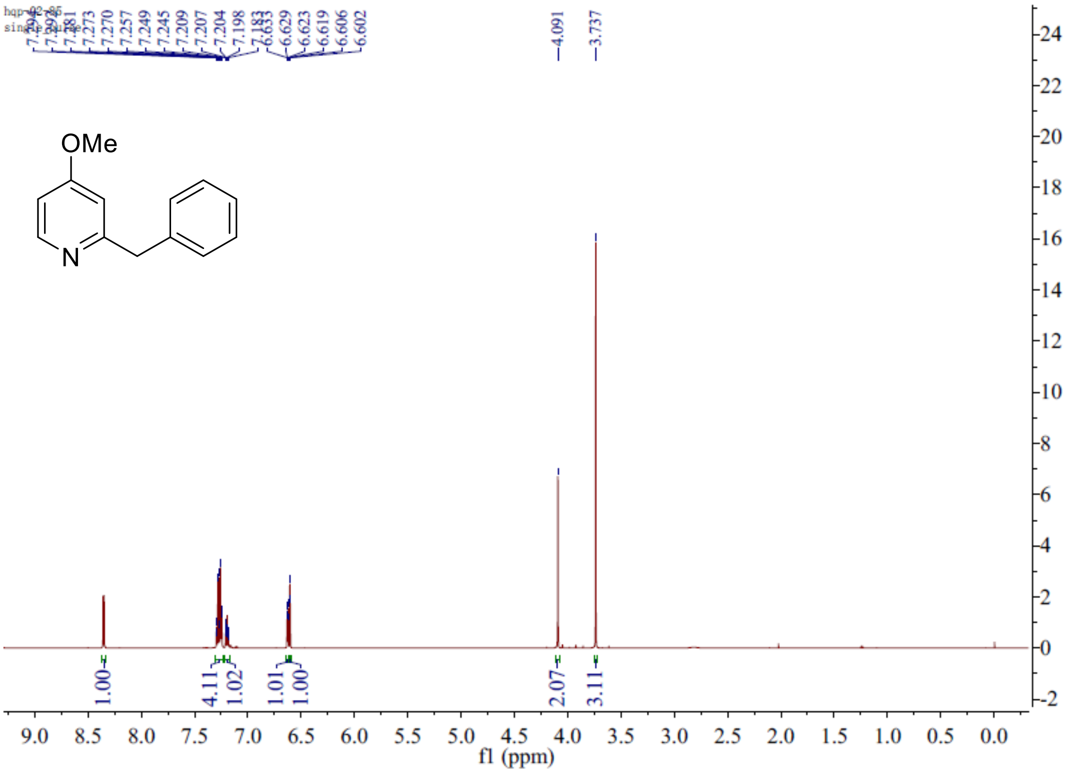

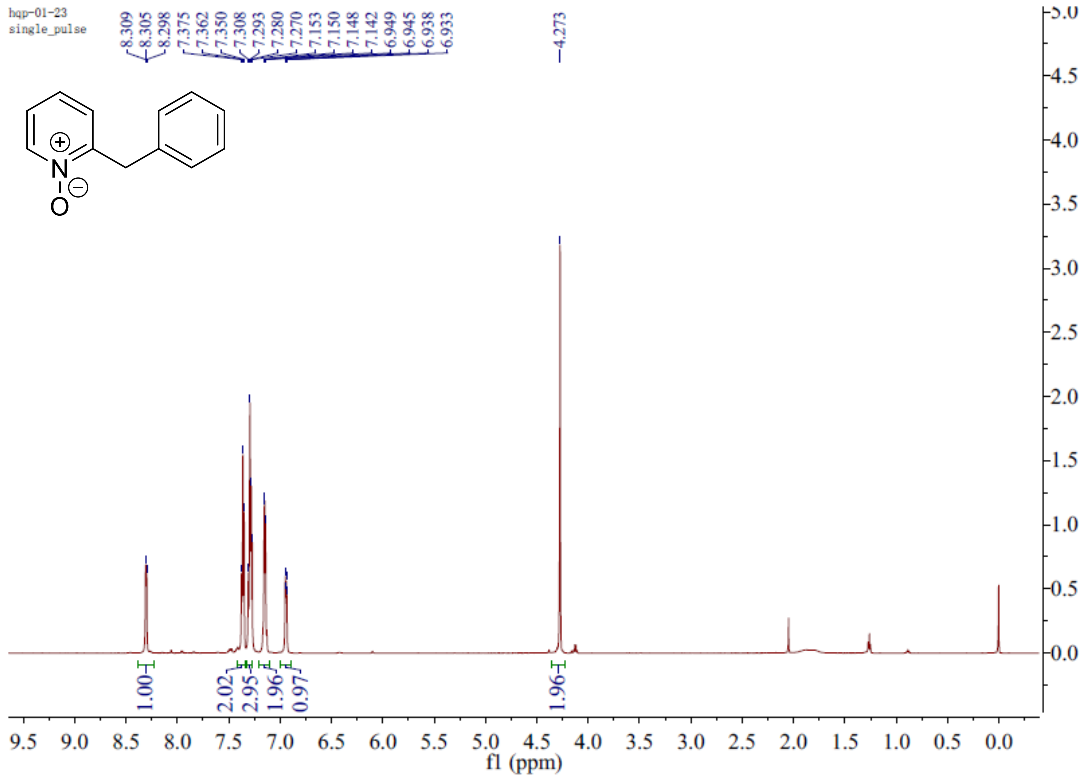
hqp-01-23
single_pulse
8.309
8.305
8.298
7.375
7.362
7.350
7.308
7.293
7.280
7.270
7.153
7.150
7.148
7.142
6.949
6.945
6.938
6.933
4.273
1.00
2.02
2.95
1.96
0.97
1.96
f1 (ppm)

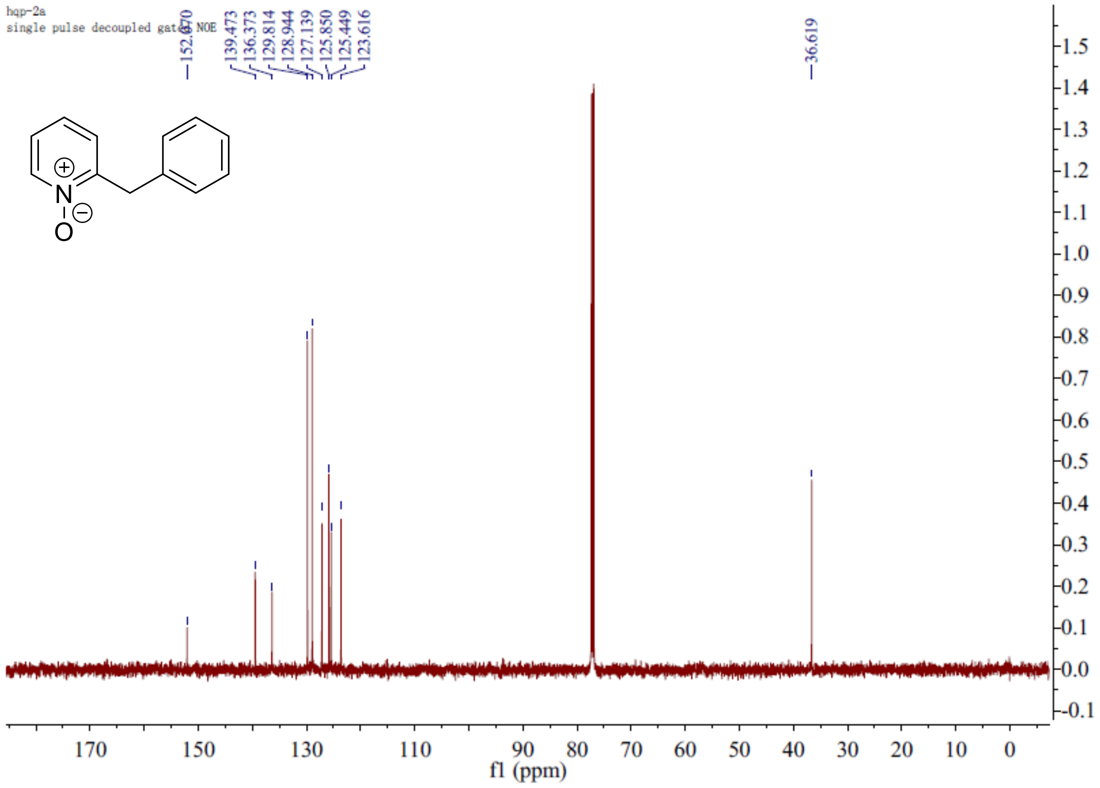
hqp-2a
single pulse decoupled gated NOE
152.070
139.473
136.373
129.814
128.944
127.139
125.850
125.449
123.616
36.619
f1 (ppm)

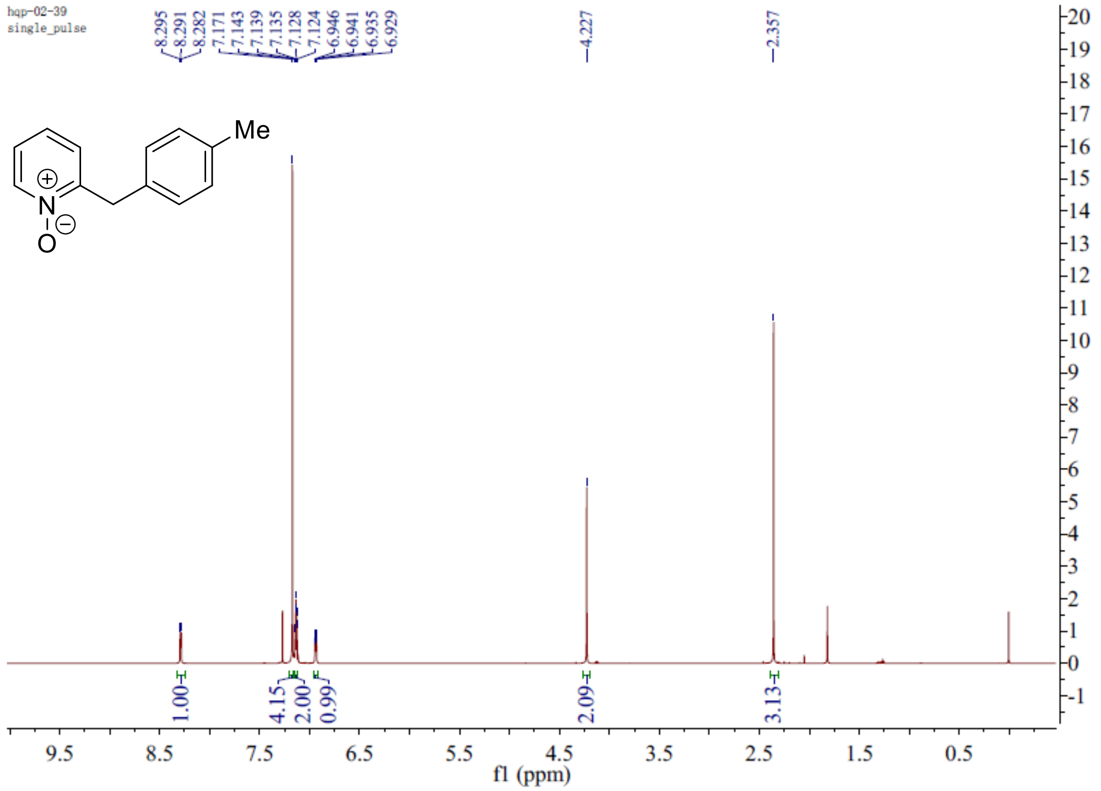
hqp-02-39
single_pulse
8.295
8.291
8.282
7.171
7.143
7.139
7.135
7.128
7.124
6.946
6.941
6.935
6.929
4.227
2.357
Me
N
O
1.00
4.15
2.00
0.99
2.09
3.13
f1 (ppm)

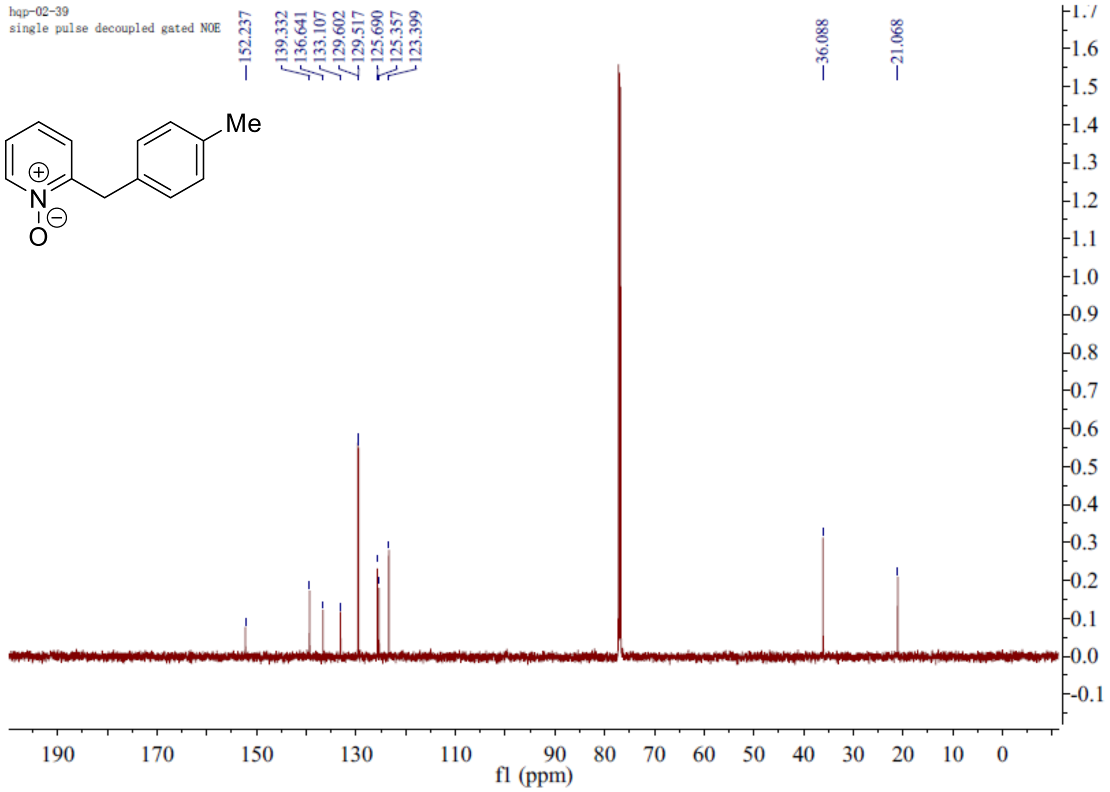
hqp-02-39
single pulse decoupled gated NOE
152.237
139.332
136.641
133.107
129.602
129.517
125.690
125.357
123.399
36.088
21.068
Me
N
O
f1 (ppm)

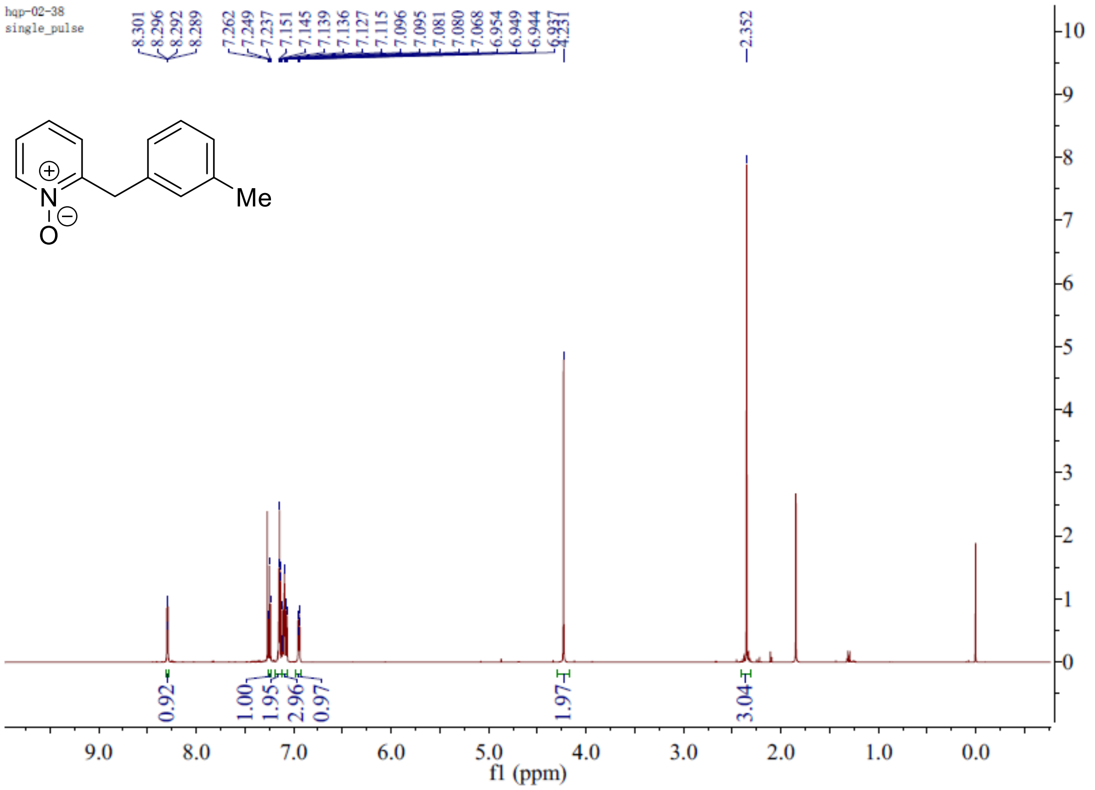

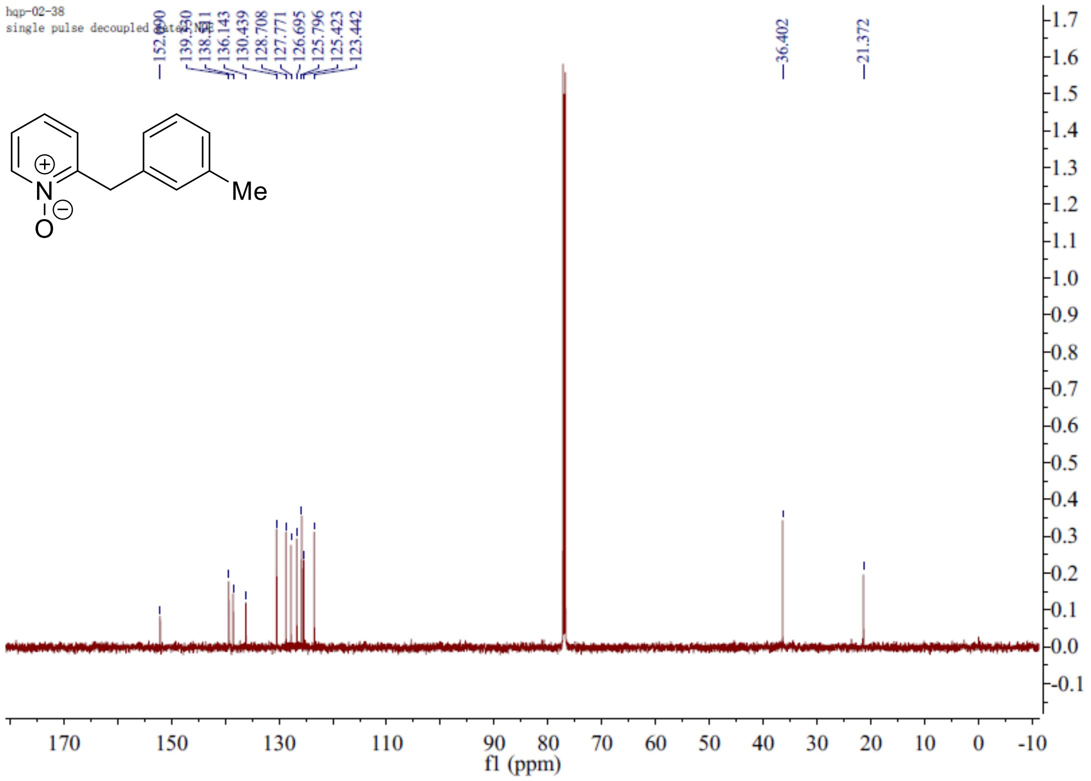

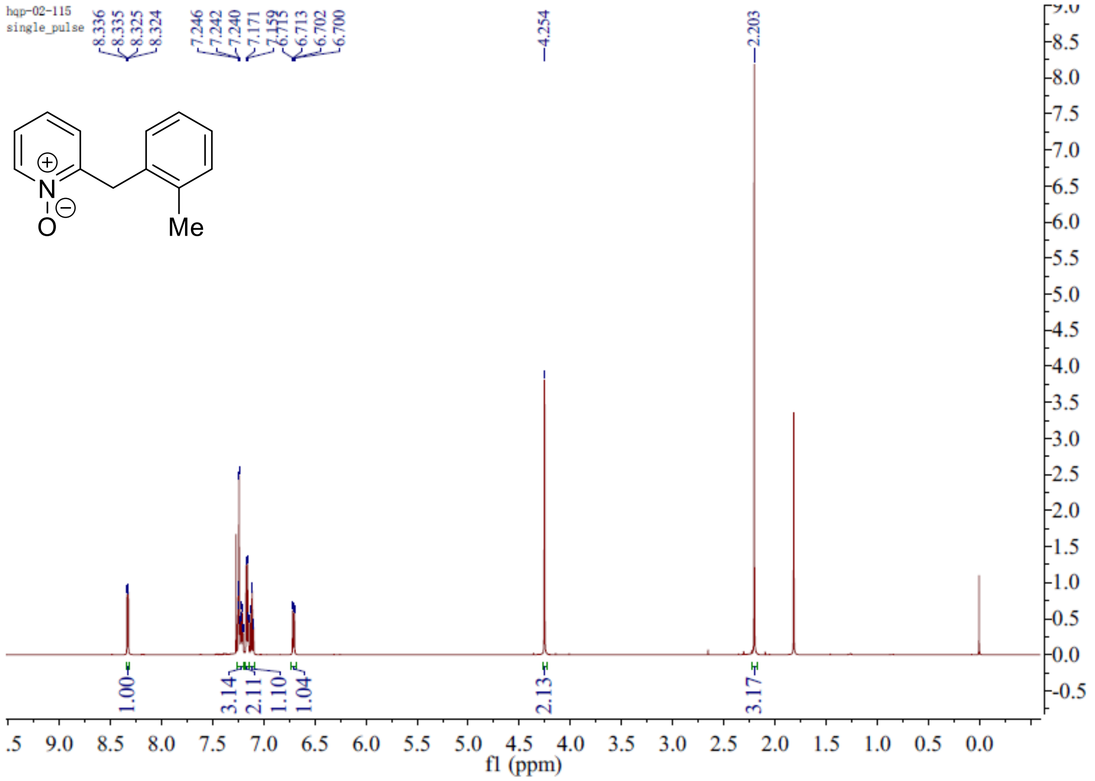
hqp-02-115
single_pulse
8.336
8.335
8.325
8.324
7.246
7.242
7.240
7.171
7.159
6.715
6.713
6.702
6.700
4.254
2.203
Me
1.00
3.14
2.11
1.10
1.04
2.13
3.17
f1 (ppm)

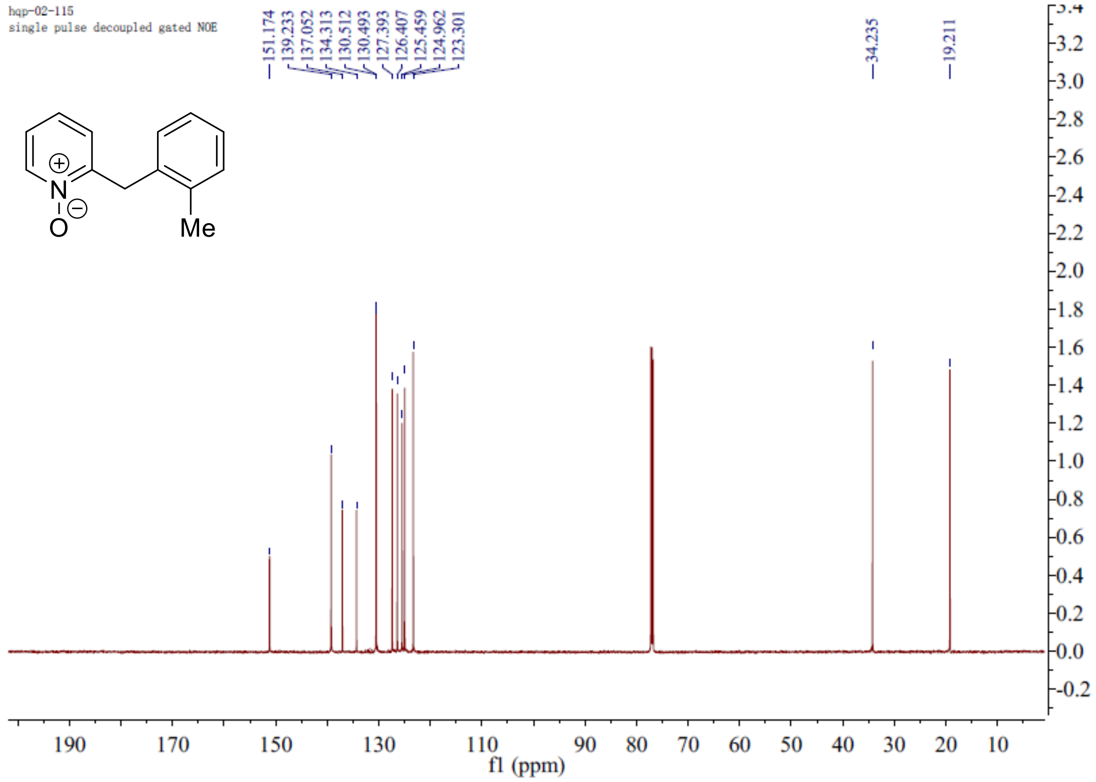
hqp-02-115
single pulse decoupled gated NOE
151.174
139.233
137.052
134.313
130.512
130.493
127.393
126.407
125.459
124.962
123.301
34.235
19.211
Me
f1 (ppm)

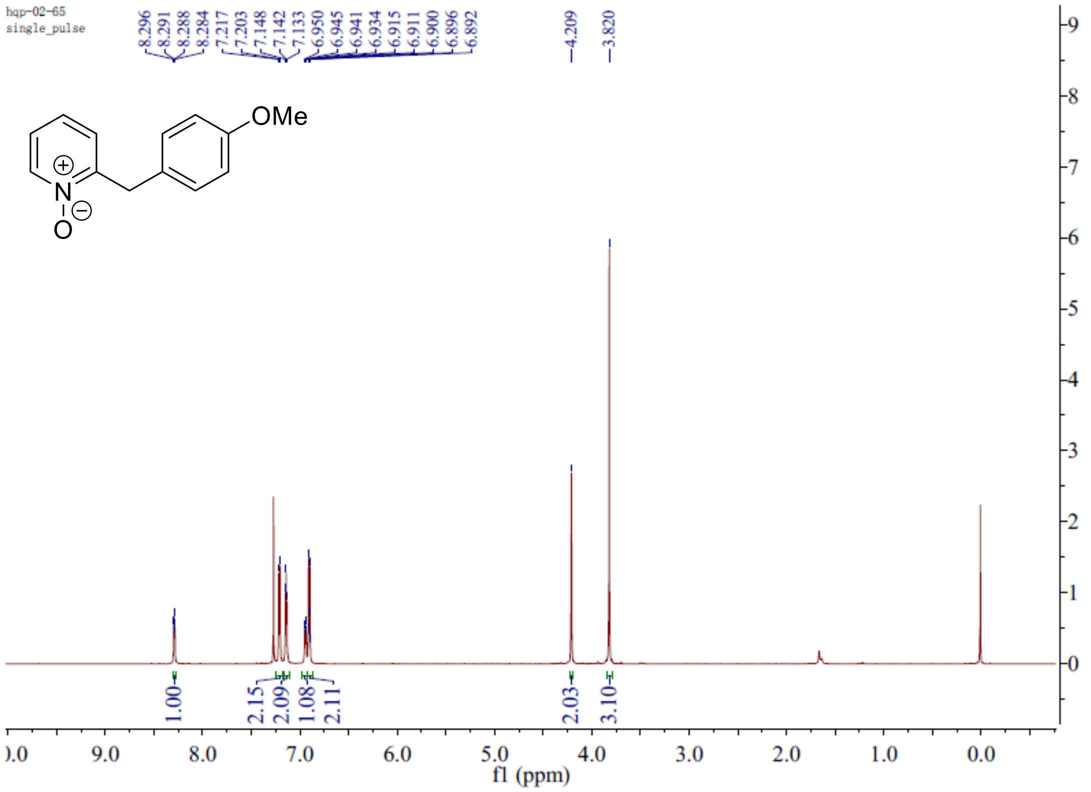
hqp-02-65
single_pulse
8.296
8.291
8.288
8.284
7.217
7.203
7.148
7.142
7.133
6.950
6.945
6.941
6.934
6.915
6.911
6.900
6.896
6.892
4.209
3.820
OMe
N
O
1.00
2.15
2.09
1.08
2.11
2.03
3.10
fl (ppm)

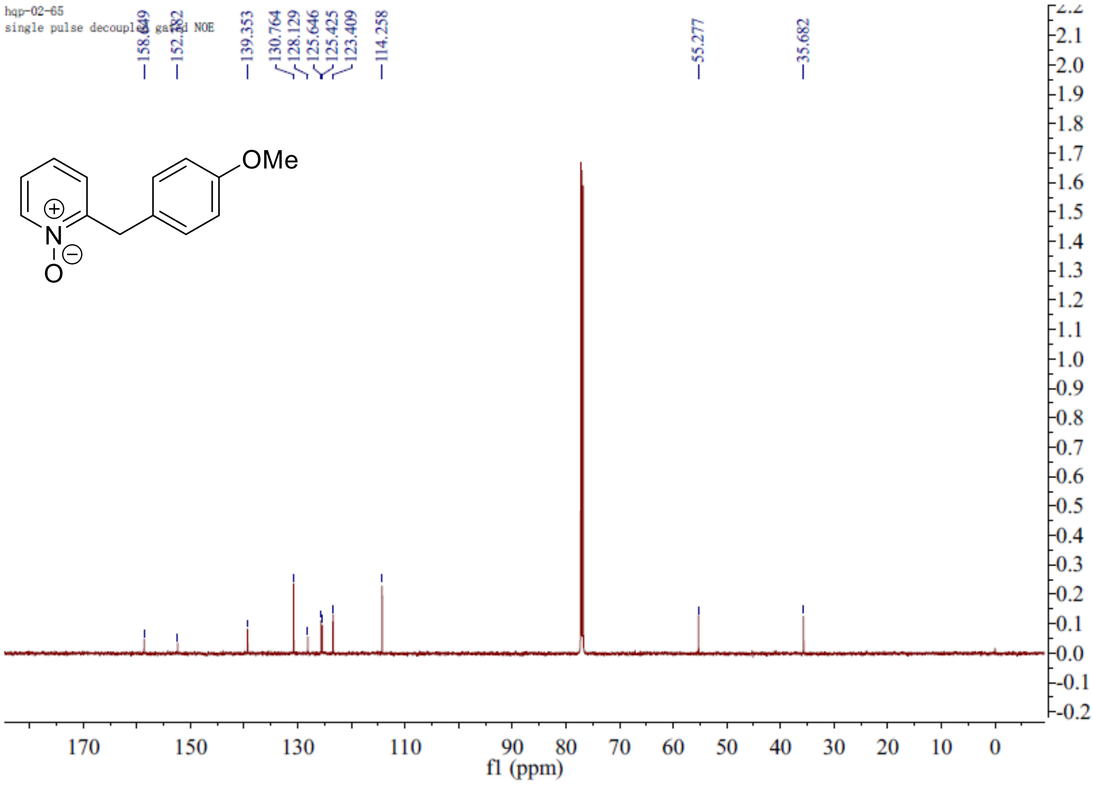
hqp-02-65
single pulse decoupled gated NOE
158.649
152.782
139.353
130.764
128.129
125.646
125.425
123.409
114.258
55.277
35.682
OMe
N
O
fl (ppm)

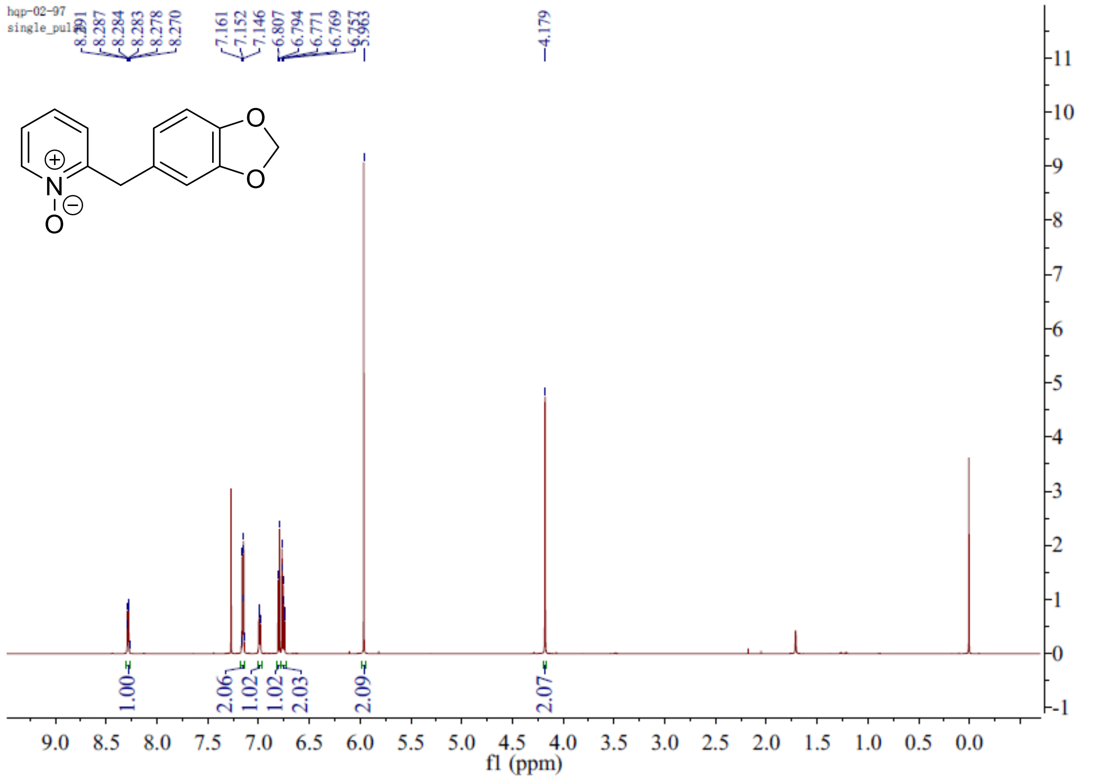
hqp-02-97
single_pulse
f1 (ppm)

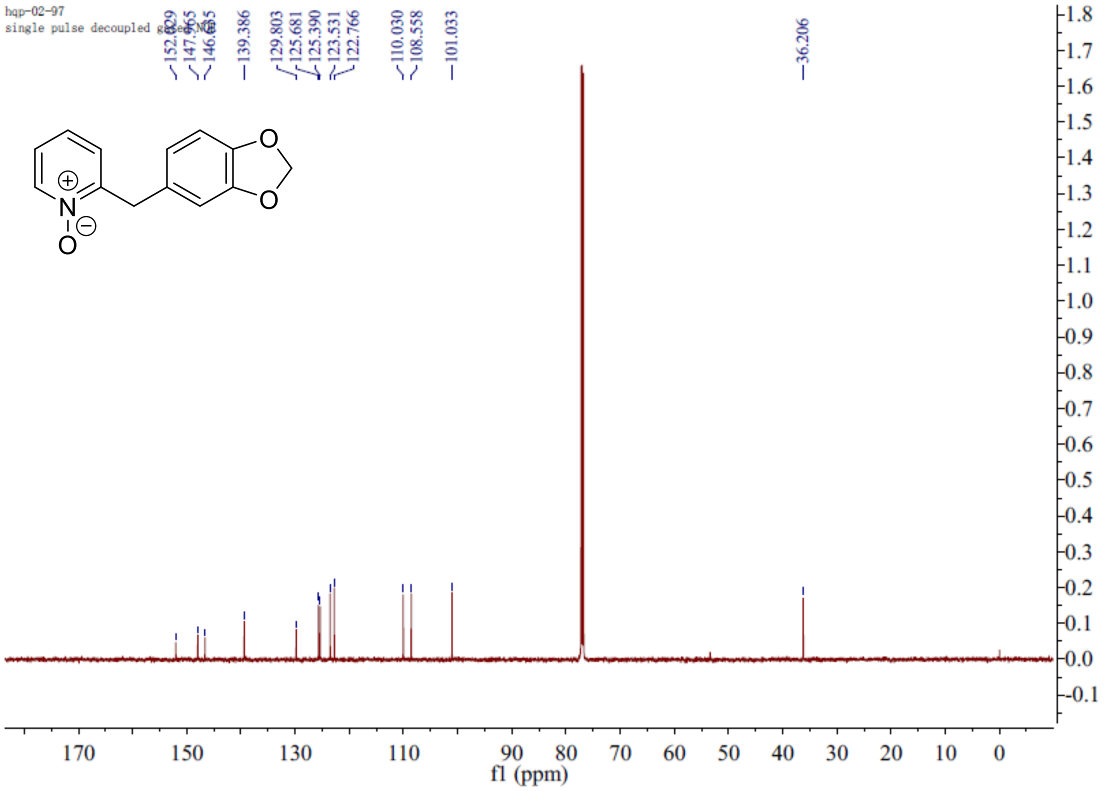
hqp-02-97
single pulse decoupled gated NOE
152.929
147.965
146.665
139.386
129.803
125.681
125.390
123.531
122.766
110.030
108.558
101.033
36.206
f1 (ppm)

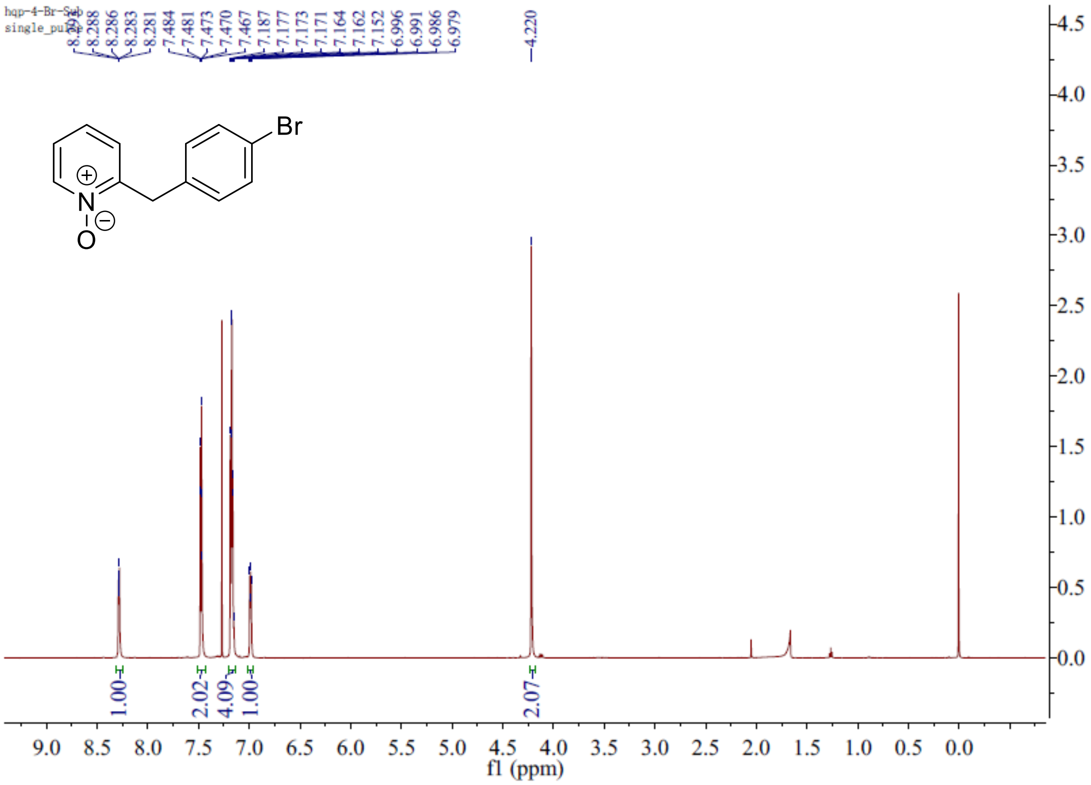
hqp-4-Br-Sub
single_pulse
8.288
8.286
8.283
8.281
7.484
7.481
7.473
7.470
7.467
7.187
7.177
7.173
7.171
7.164
7.162
7.152
6.996
6.991
6.986
6.979
4.220
Br
N
O
1.00
2.02
4.09
1.00
2.07
f1 (ppm)

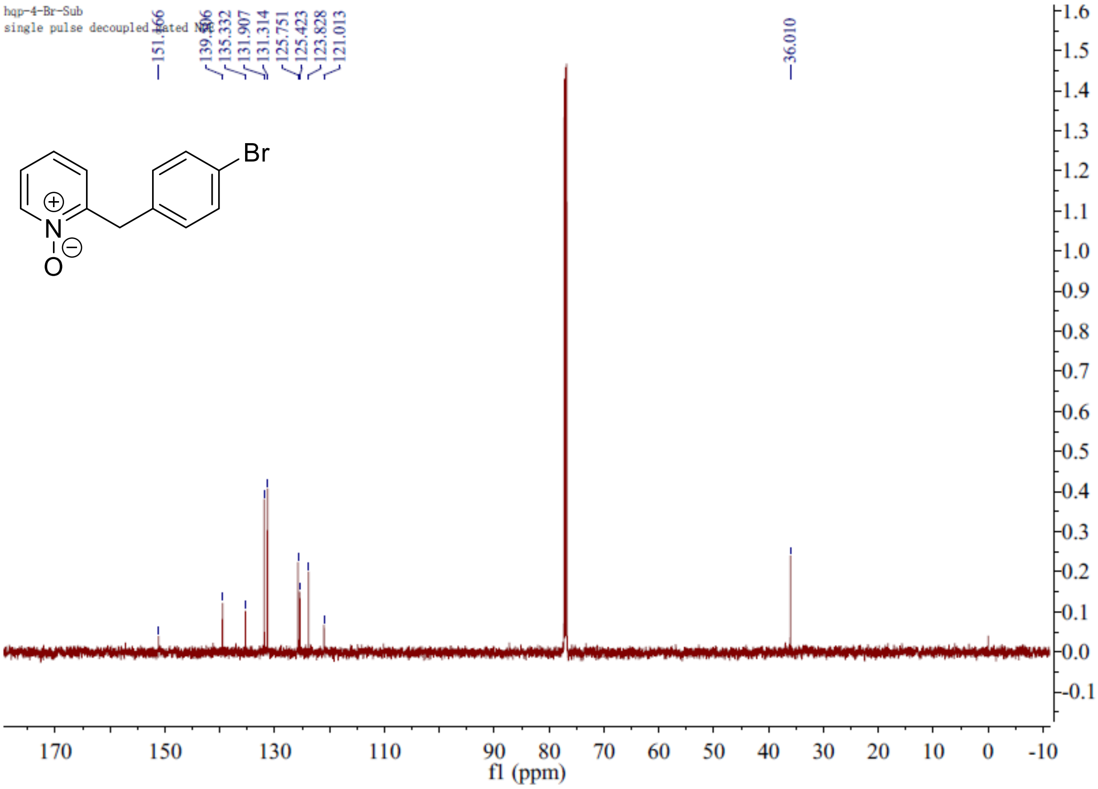
hqp-4-Br-Sub
single pulse decoupled
135.332
131.907
131.314
125.751
125.423
123.828
121.013
36.010
Br
N
O
f1 (ppm)

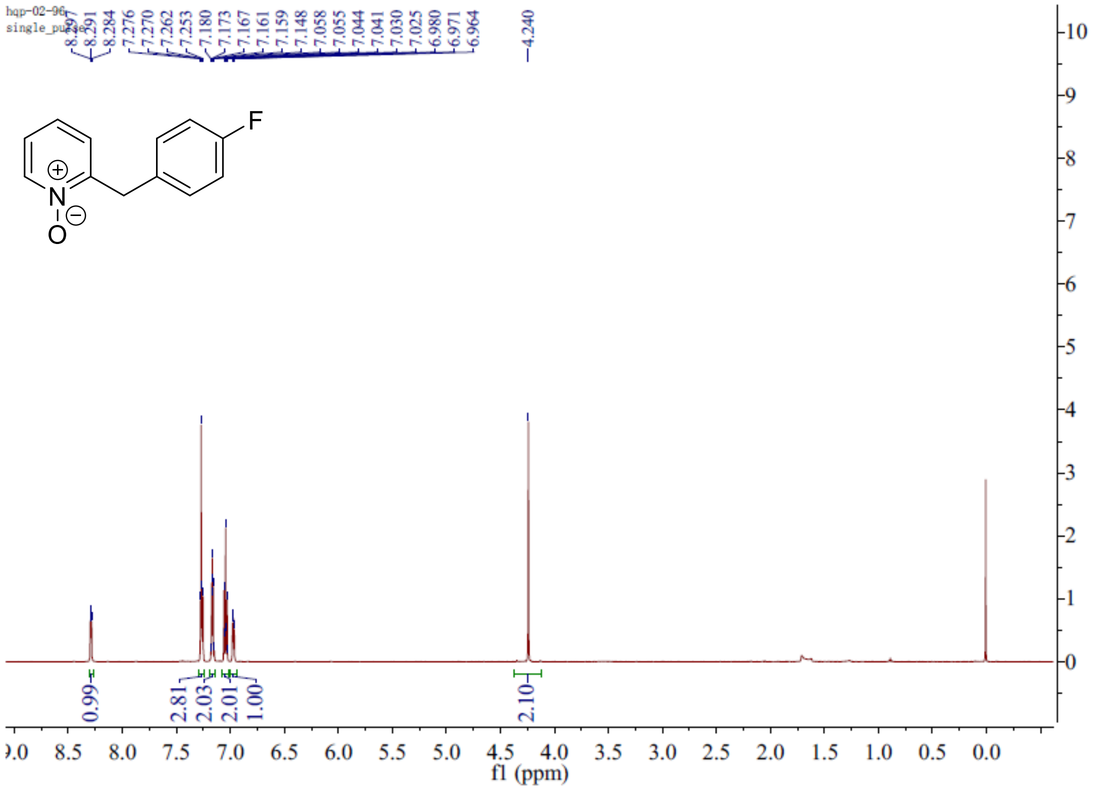

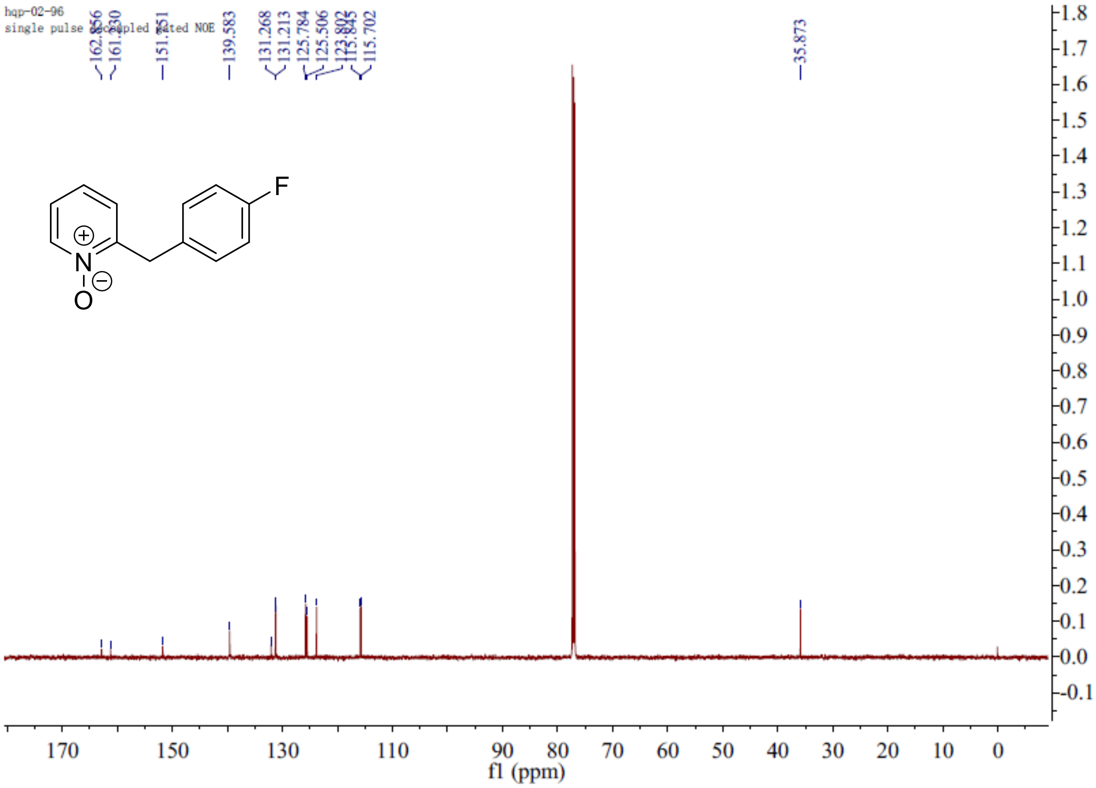

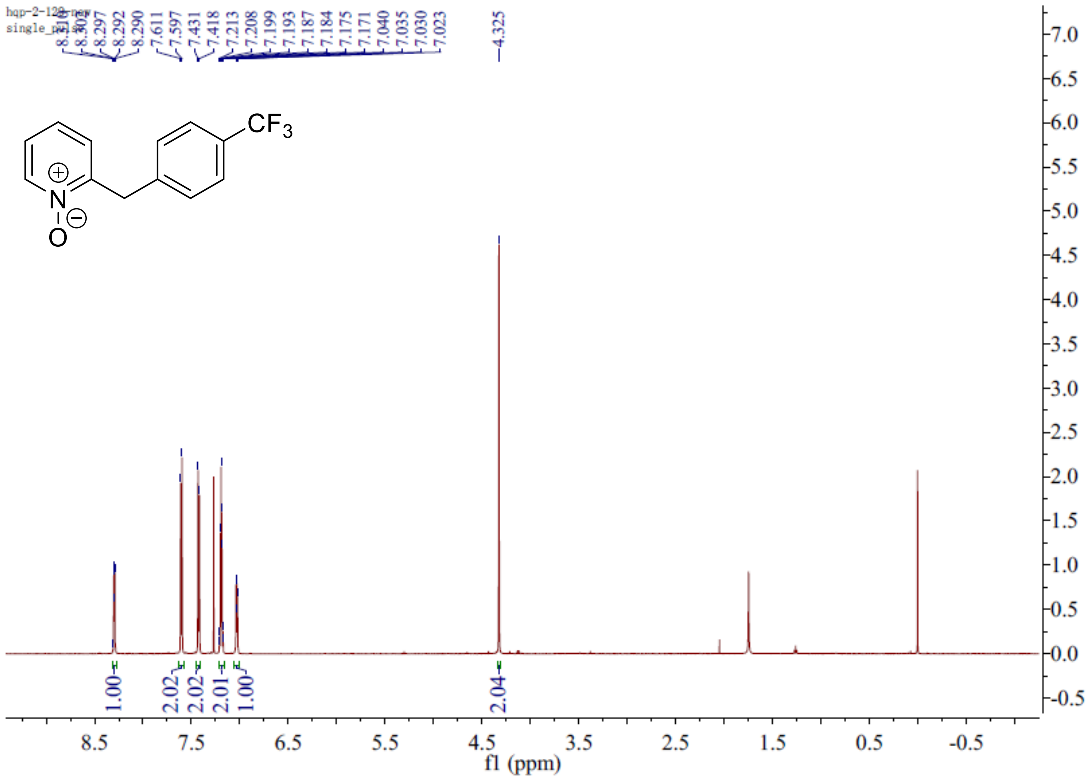
hqp-2-129-new
single_pulse
8.316
8.302
8.297
8.292
8.290
7.611
7.597
7.431
7.418
7.213
7.208
7.199
7.193
7.187
7.184
7.175
7.171
7.040
7.035
7.030
7.023
4.325
CF3
N
O
1.00
2.02
2.02
2.01
1.00
2.04
f1 (ppm)

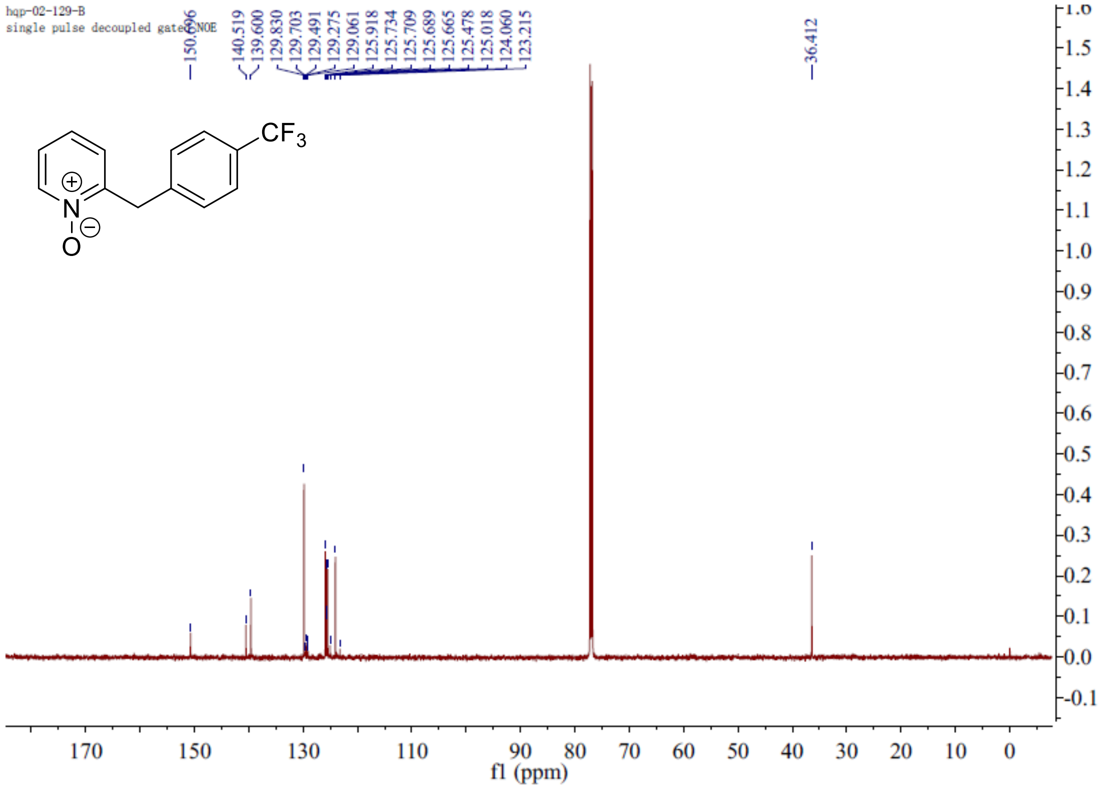
hqp-02-129-B
single pulse decoupled gated NOE
150.096
140.519
139.600
129.830
129.703
129.491
129.275
129.061
125.918
125.734
125.709
125.689
125.665
125.478
125.018
124.060
123.215
36.412
CF3
N
O
f1 (ppm)

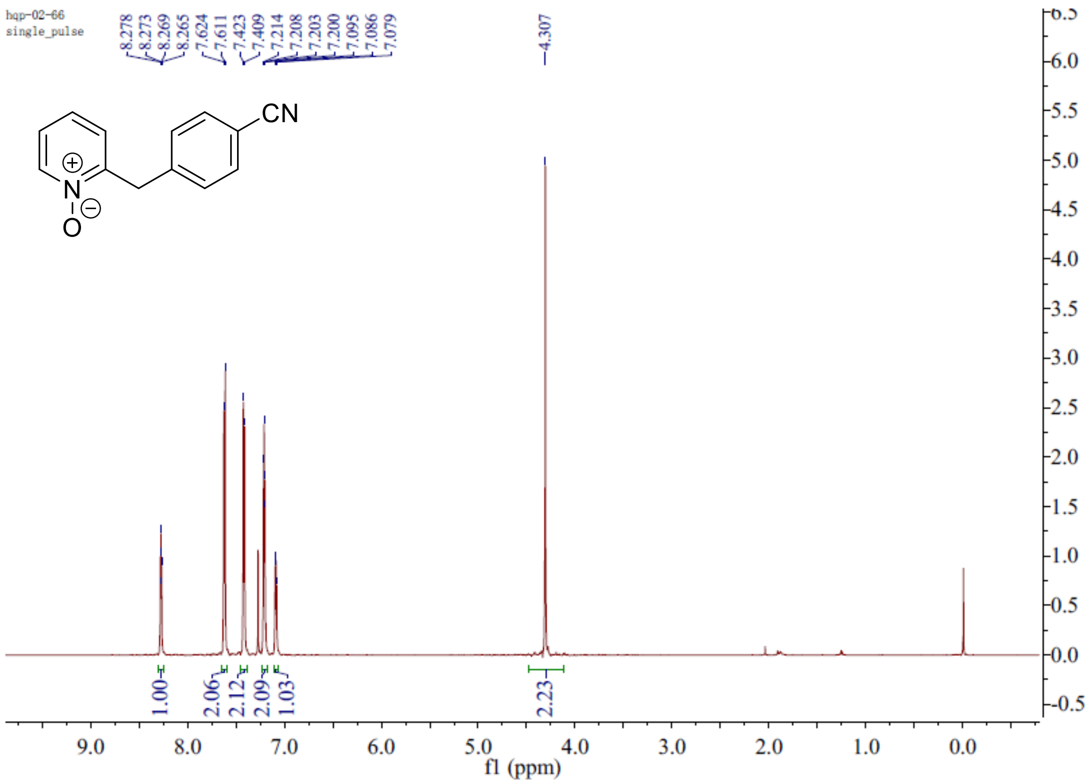
hqp-02-66
single_pulse
8.278
8.273
8.269
8.265
7.624
7.611
7.423
7.409
7.214
7.208
7.203
7.200
7.095
7.086
7.079
4.307
CN
N
O
1.00
2.06
2.12
2.09
1.03
2.23
f1 (ppm)

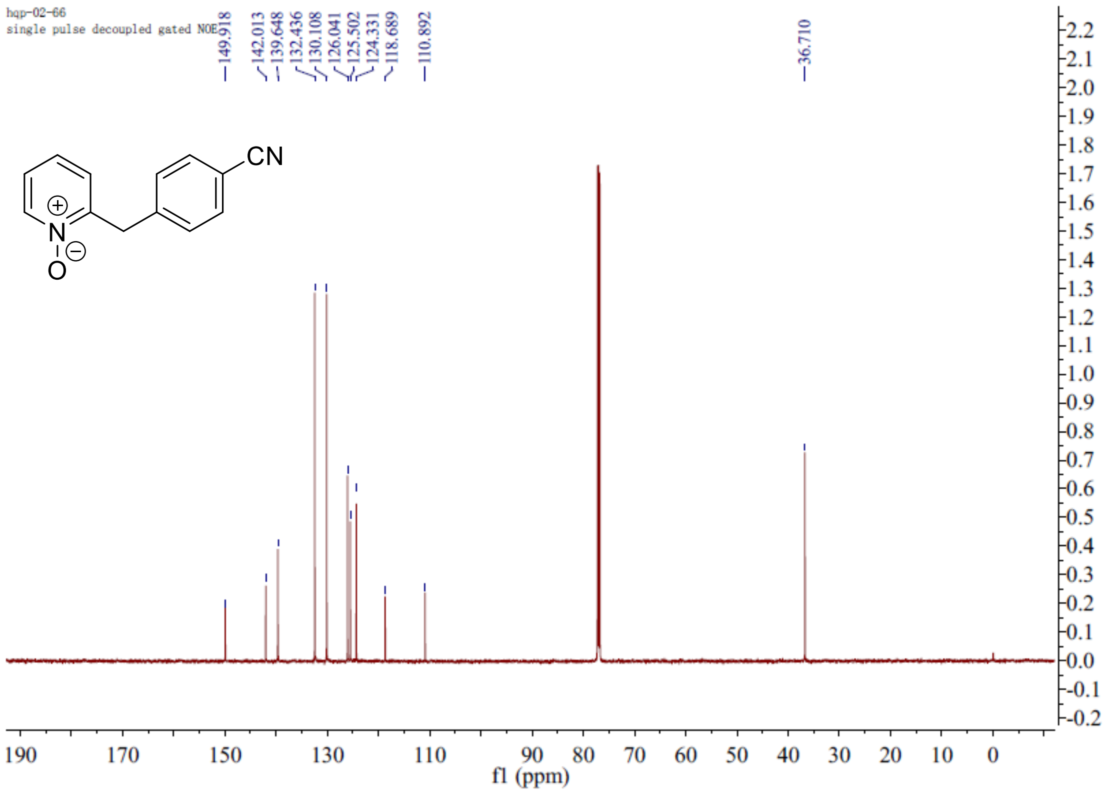
hqp-02-66
single pulse decoupled gated NOE
149.918
142.013
139.648
132.436
130.108
126.041
125.502
124.331
118.689
110.892
36.710
CN
N
O
f1 (ppm)

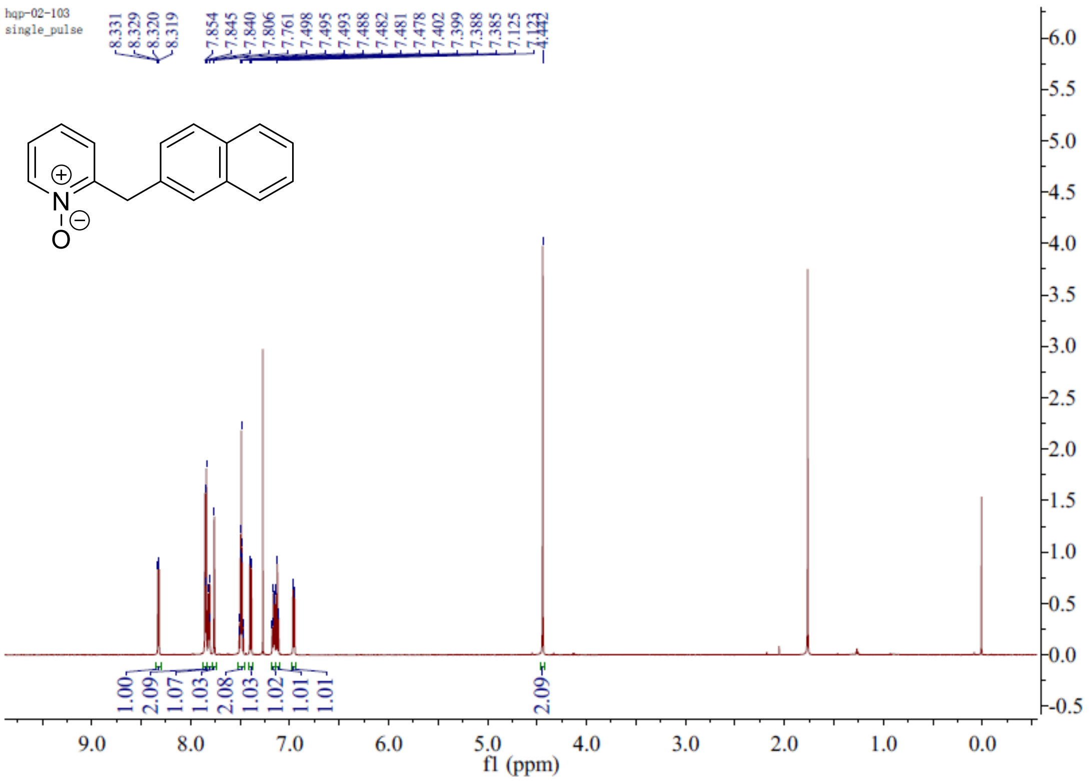
hqp-02-103
single_pulse
8.331
8.329
8.320
8.319
7.854
7.845
7.840
7.806
7.761
7.498
7.495
7.493
7.488
7.482
7.481
7.478
7.402
7.399
7.388
7.385
7.125
4.442
1.00
2.09
1.07
1.03
2.08
1.03
1.02
1.01
1.01
2.09
fl (ppm)

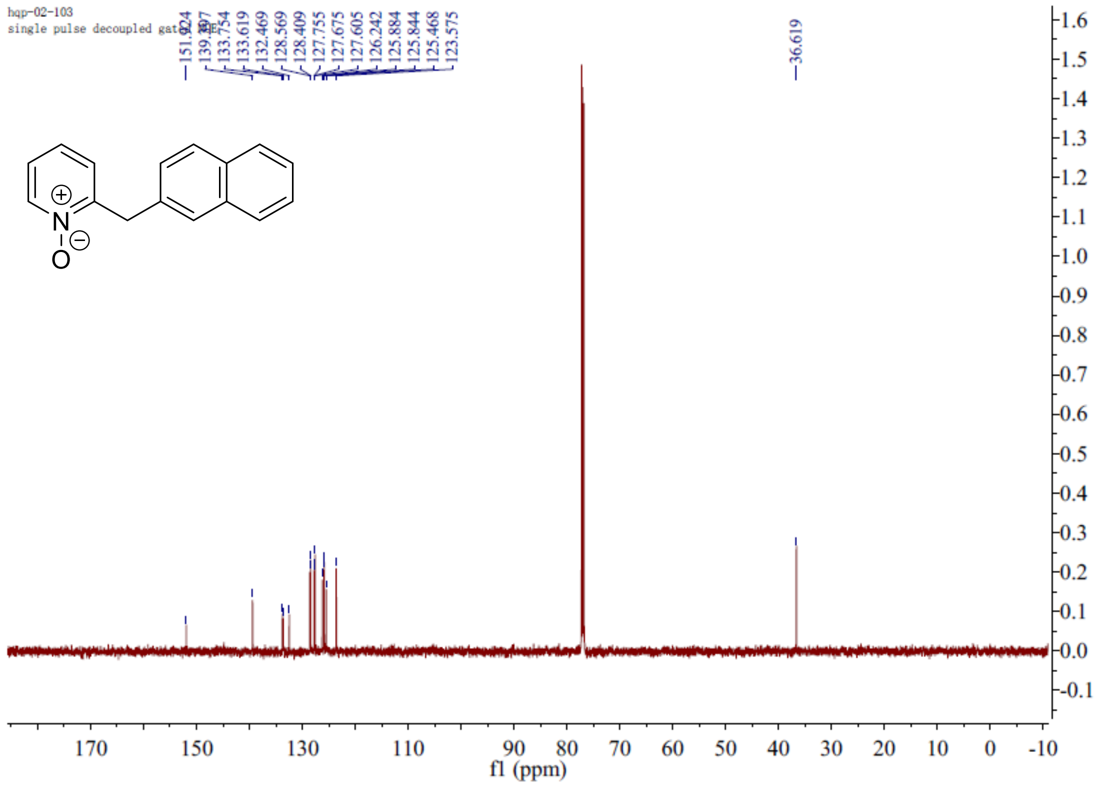
hqp-02-103
single pulse decoupled
151.924
133.754
133.619
132.469
128.569
128.409
127.755
127.675
127.605
126.242
125.884
125.844
125.468
123.575
36.619
fl (ppm)

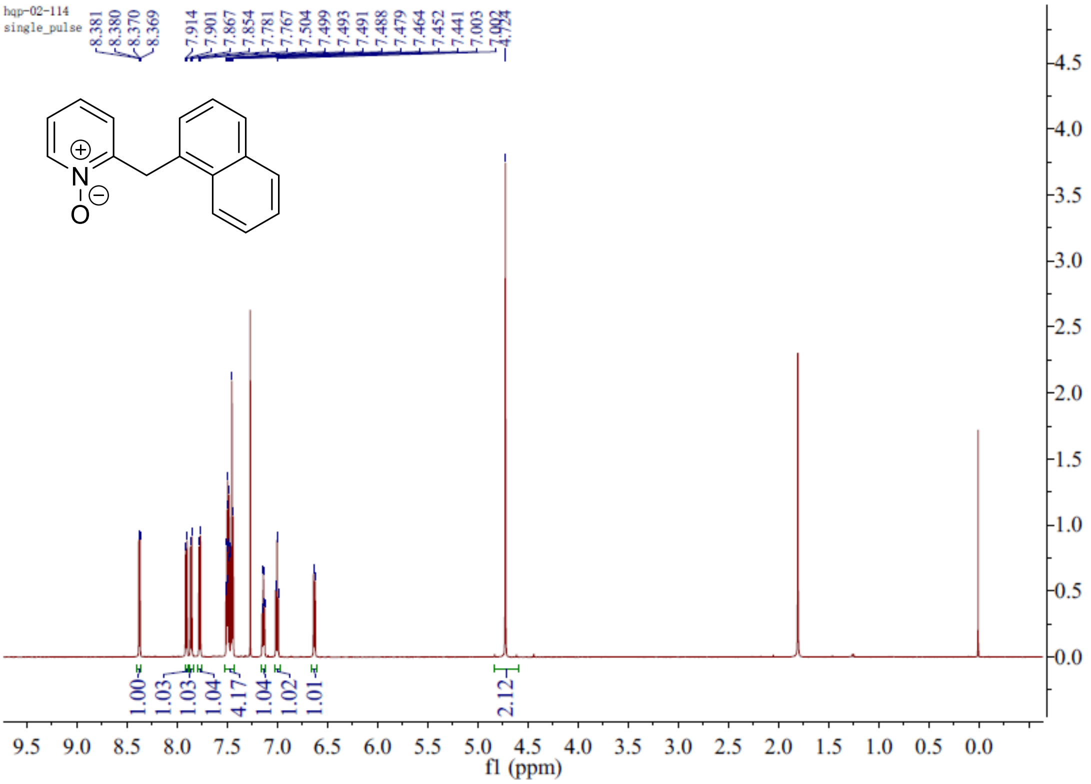
hqp-02-114
single_pulse
8.381
8.380
8.370
8.369
7.914
7.901
7.867
7.854
7.781
7.767
7.504
7.499
7.493
7.491
7.488
7.479
7.464
7.452
7.441
7.003
7.002
4.724
1.00
1.03
1.03
1.04
4.17
1.04
1.02
1.01
2.12
f1 (ppm)

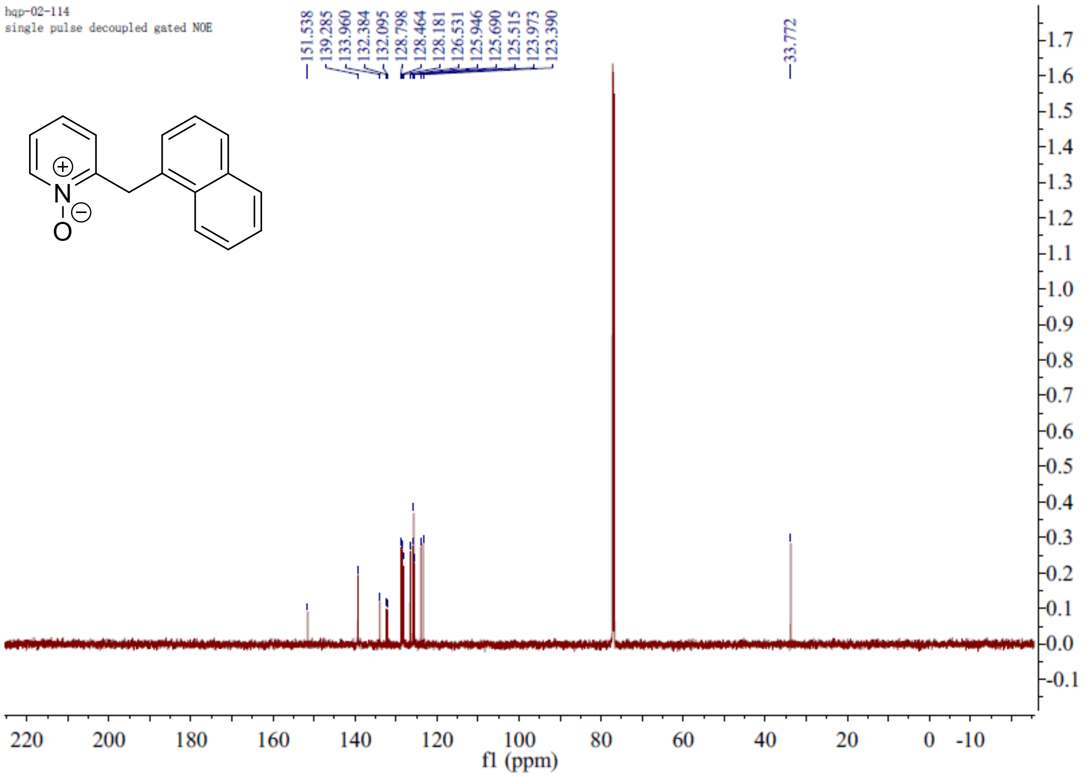
hqp-02-114
single pulse decoupled gated NOE
151.538
139.285
133.960
132.384
132.095
128.798
128.464
128.181
126.531
125.946
125.690
125.515
123.973
123.390
33.772
f1 (ppm)

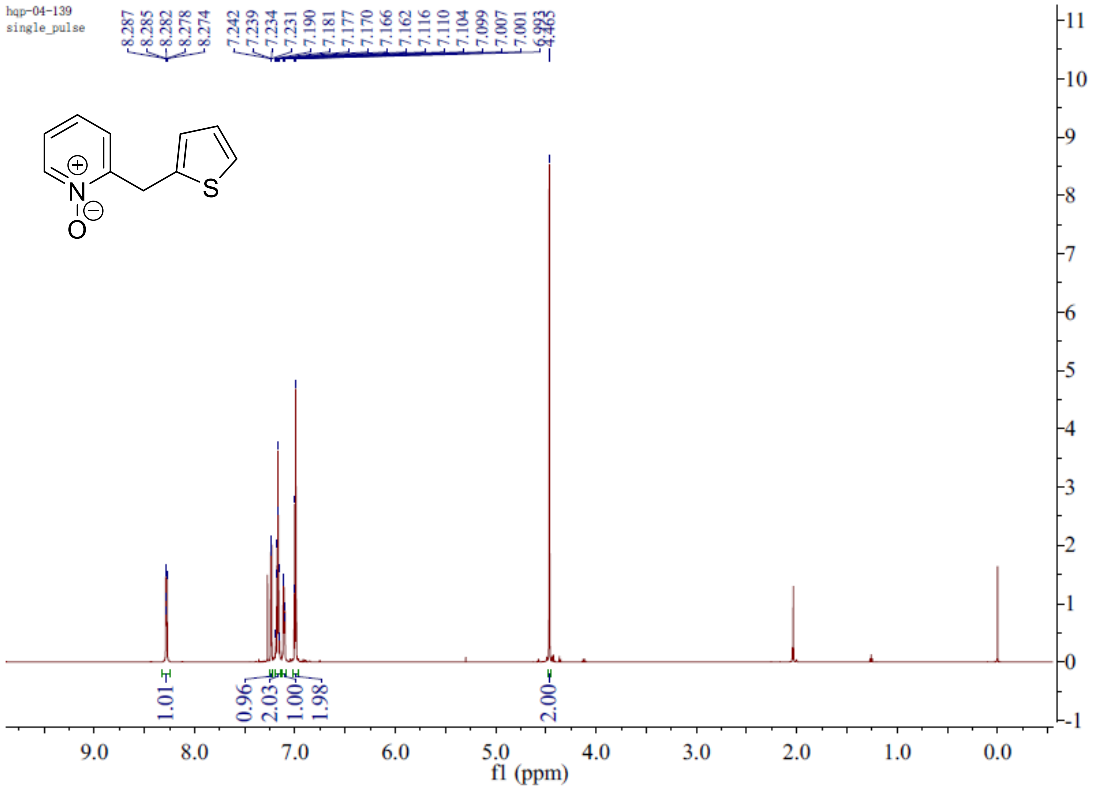

hqp-04-139
single_pulse
8.287
8.285
8.282
8.278
8.274
7.242
7.239
7.234
7.231
7.190
7.181
7.177
7.170
7.166
7.162
7.116
7.110
7.104
7.099
7.007
7.001
6.993
4.465
N
O
S
1.01
0.96
2.03
1.00
1.98
2.00
f1 (ppm)

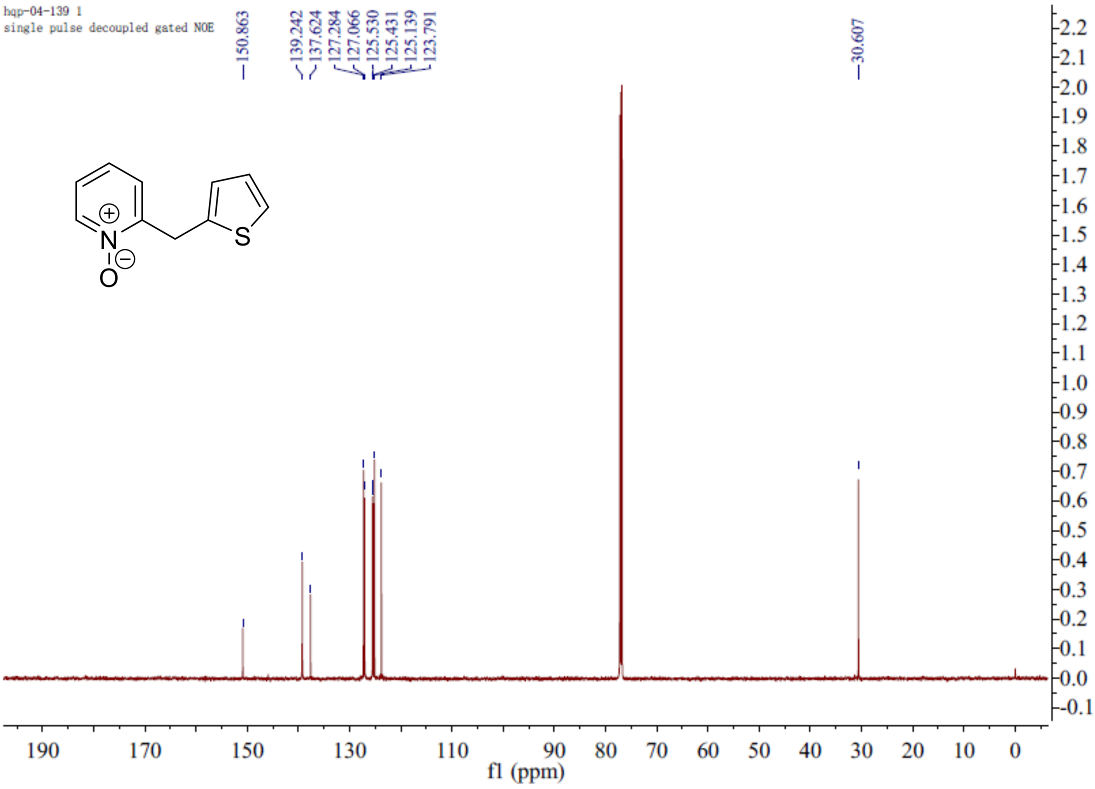

hqp-04-139 1
single pulse decoupled gated NOE
150.863
139.242
137.624
127.284
127.066
125.530
125.431
125.139
123.791
30.607
N
O
S
f1 (ppm)

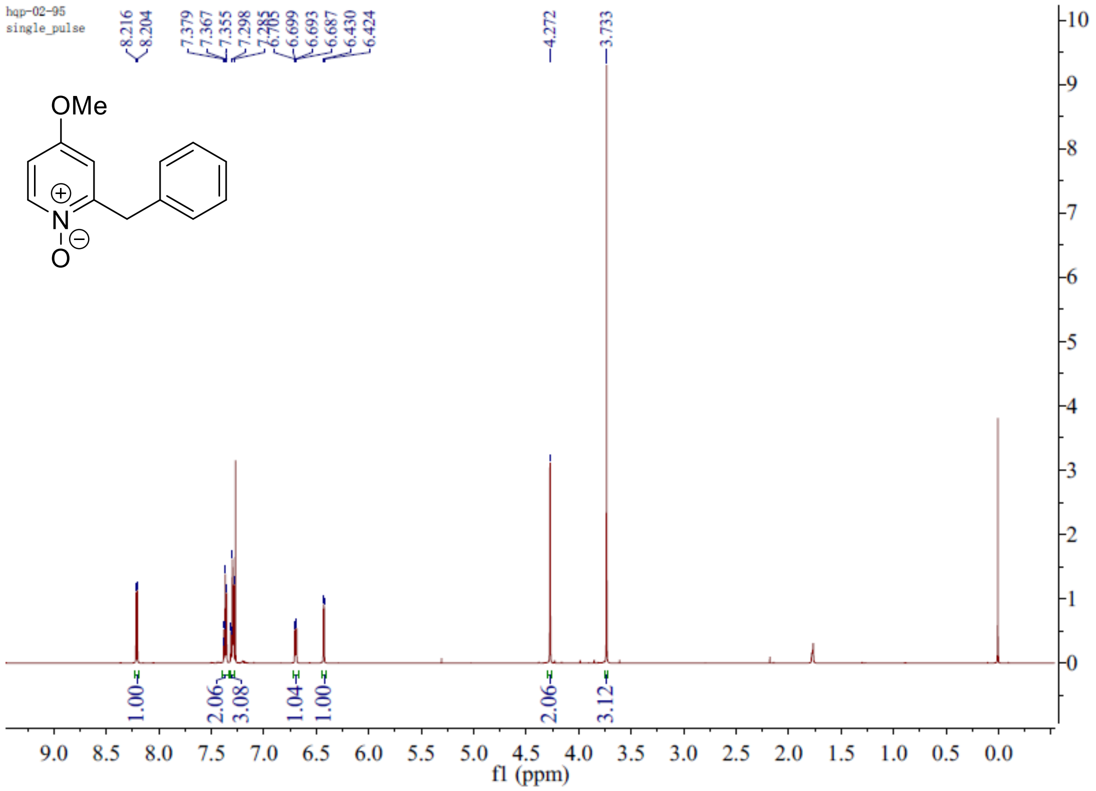
hqp-02-95
single_pulse
8.216
8.204
7.379
7.367
7.355
7.298
7.285
6.705
6.699
6.693
6.687
6.430
6.424
4.272
3.733
OMe
N
O
1.00
2.06
3.08
1.04
1.00
2.06
3.12
f1 (ppm)

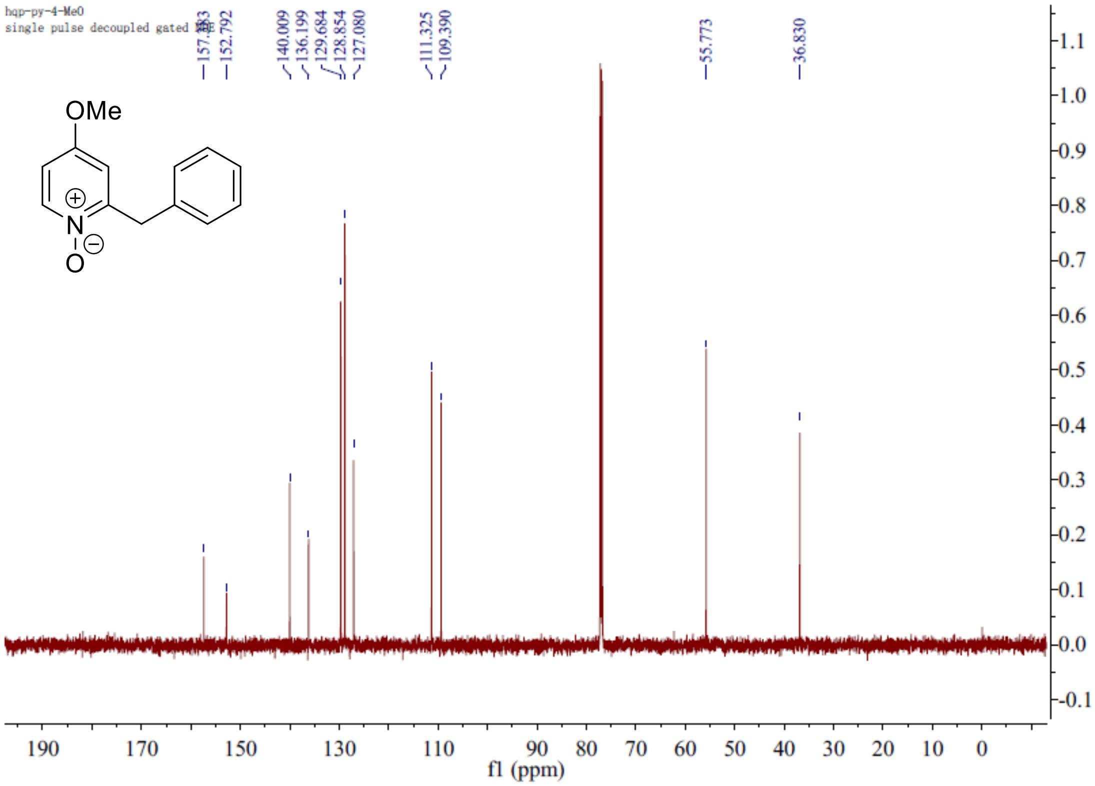
hqp-py-4-MeO
single pulse decoupled gated
152.792
140.009
136.199
129.684
128.854
127.080
111.325
109.390
55.773
36.830
OMe
N
O
f1 (ppm)

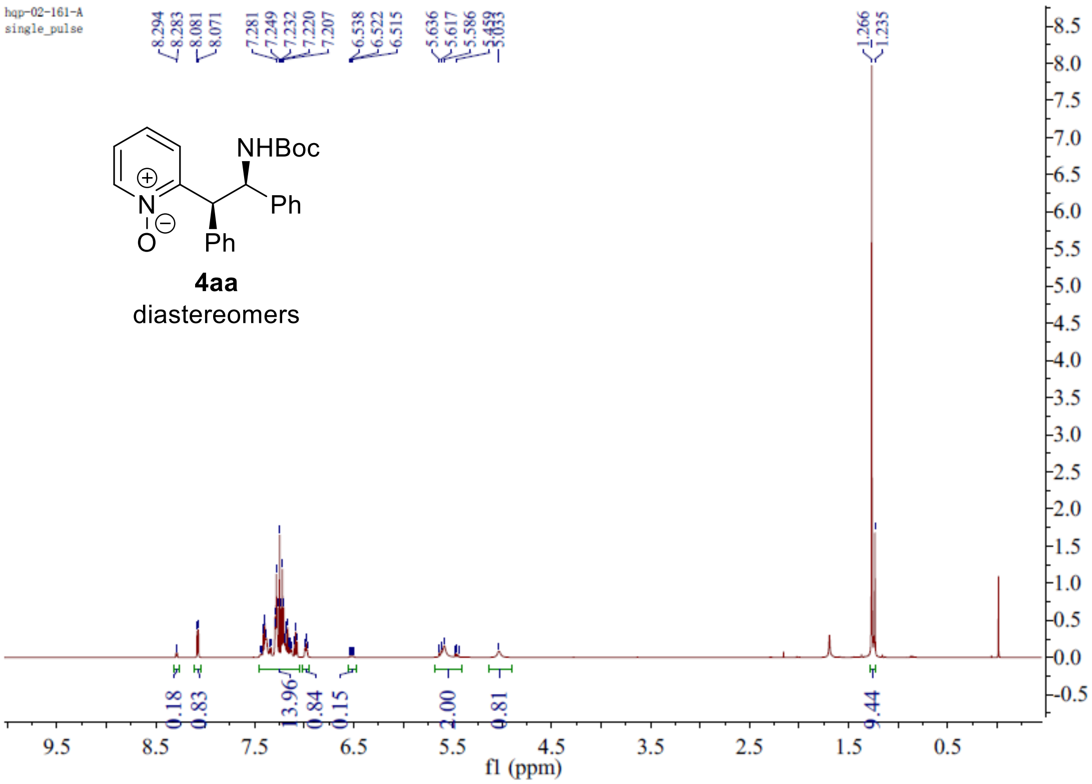
hqp-02-161-A
single_pulse
8.294
8.283
8.081
8.071
7.281
7.249
7.232
7.220
7.207
6.538
6.522
6.515
5.636
5.617
5.586
5.459
5.033
1.266
1.235
NHBoc
Ph
N
O
Ph
4aa
diastereomers
0.18
0.83
13.96
0.84
0.15
2.00
0.81
9.44
f1 (ppm)

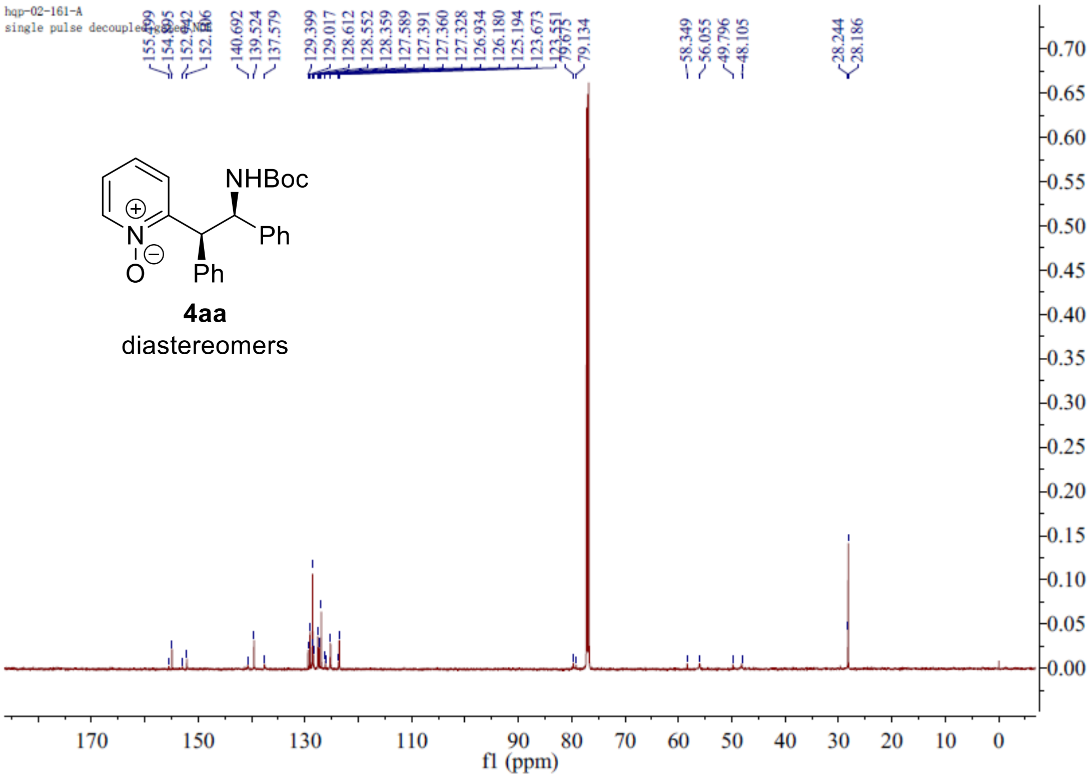
hqp-02-161-A
single pulse decoupled gated NOE
155.499
154.595
152.942
152.306
140.692
139.524
137.579
129.399
129.017
128.612
128.552
128.359
127.589
127.391
127.360
127.328
126.934
126.180
125.194
123.673
123.551
79.675
79.134
58.349
56.055
49.796
48.105
28.244
28.186
NHBoc
Ph
N
O
Ph
4aa
diastereomers
f1 (ppm)

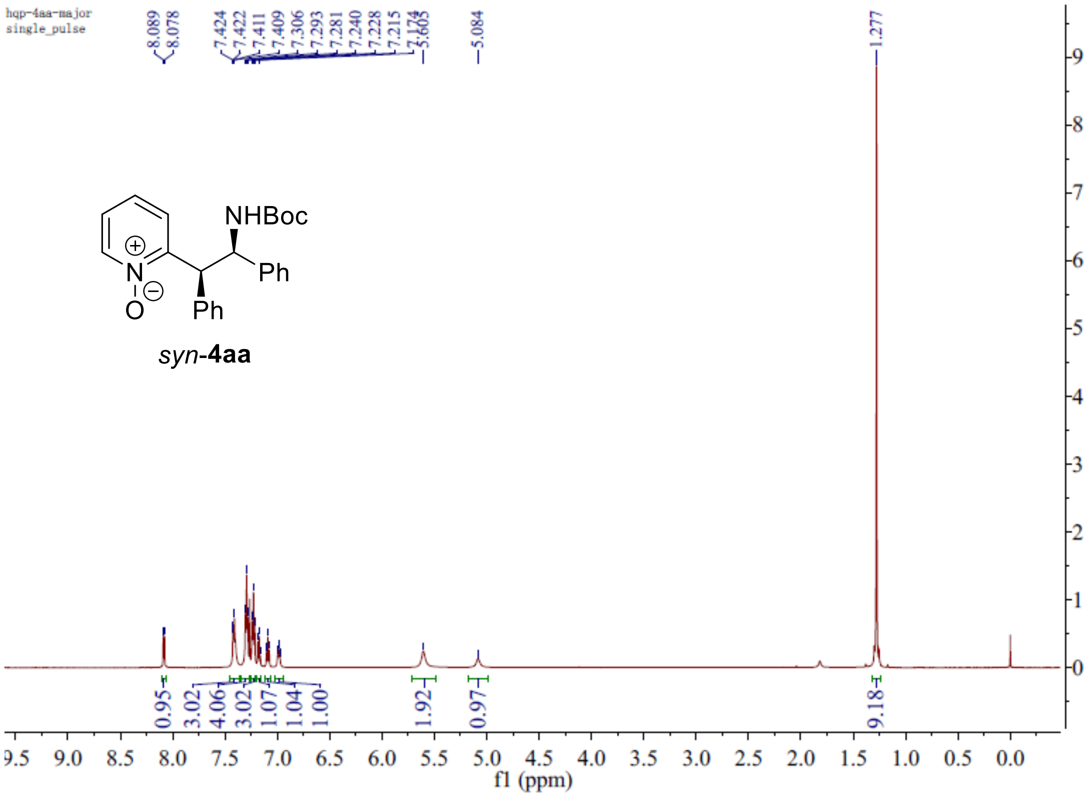
hqp-4aa-major
single_pulse
8.089
8.078
7.424
7.422
7.411
7.409
7.306
7.293
7.281
7.240
7.228
7.215
7.174
5.603
5.084
1.277
NHBoc
Ph
Ph
N
O
syn-**4aa**
0.95
3.02
4.06
3.02
1.07
1.04
1.00
1.92
0.97
9.18
f1 (ppm)

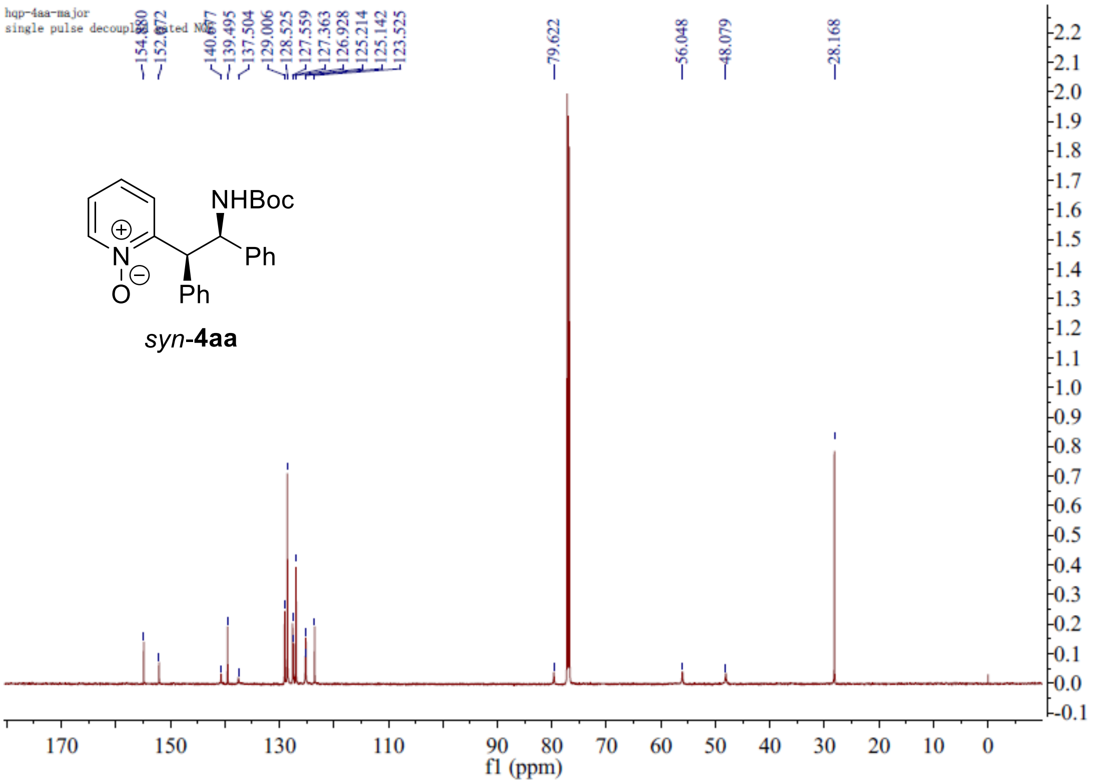
hqp-4aa-major
154.880
152.672
140.677
139.495
137.504
129.006
128.525
127.559
127.363
126.928
125.214
125.142
123.525
79.622
56.048
48.079
28.168
NHBoc
Ph
Ph
N
O
syn-**4aa**
f1 (ppm)

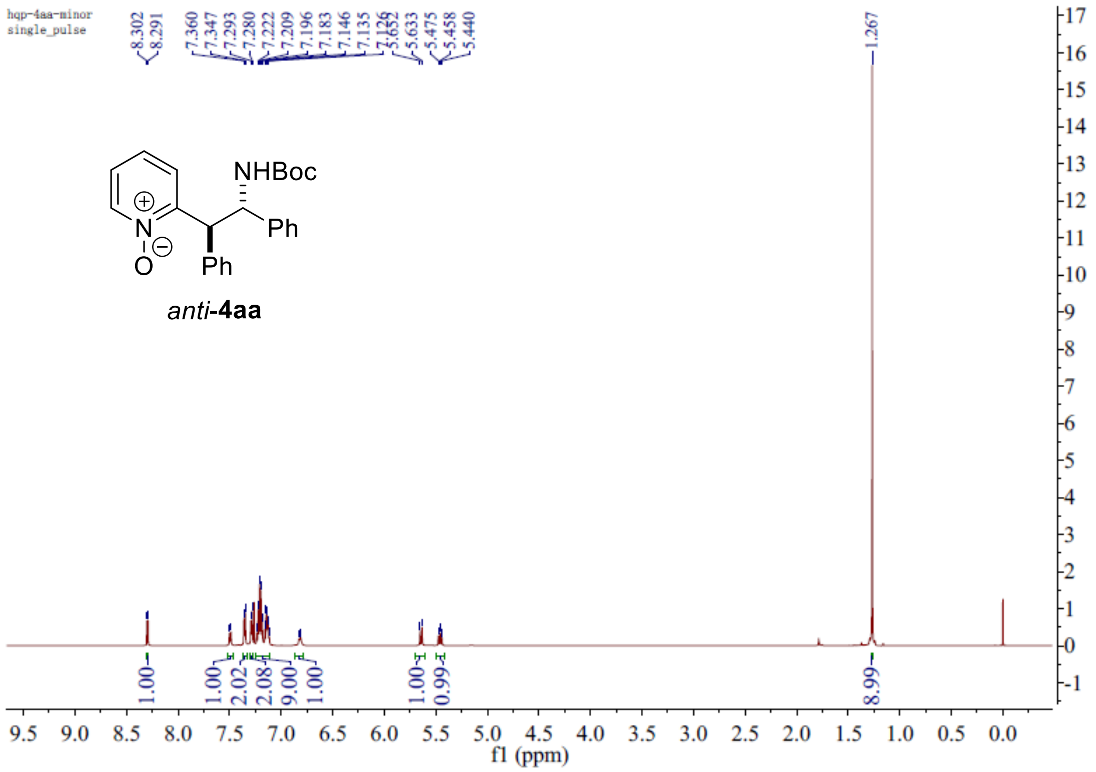
hqp-4aa-minor
single_pulse
8.302
8.291
7.360
7.347
7.293
7.280
7.222
7.209
7.196
7.183
7.146
7.135
5.633
5.475
5.458
5.440
1.267
NHBoc
Ph
Ph
anti-4aa
1.00
1.00
2.02
2.08
9.00
1.00
1.00
0.99
8.99
f1 (ppm)

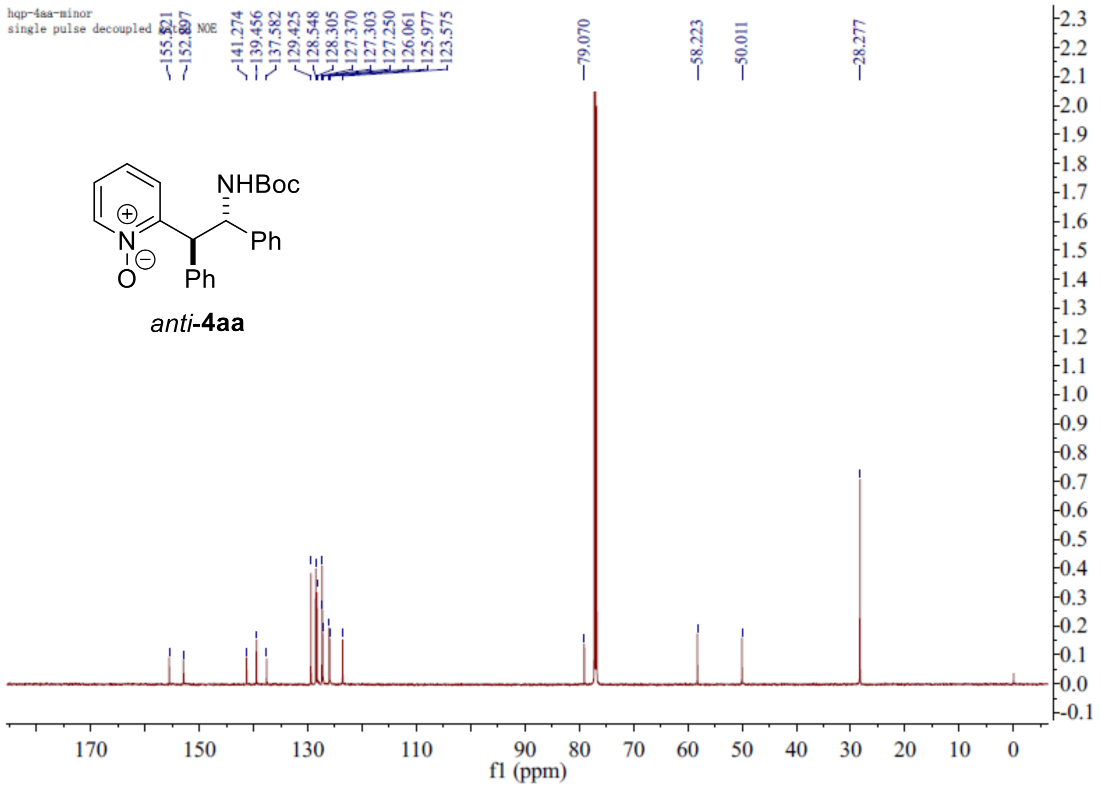
hqp-4aa-minor
141.274
139.456
137.582
129.425
128.548
128.305
127.370
127.303
127.250
126.061
125.977
123.575
79.070
58.223
50.011
28.277
NHBoc
Ph
Ph
anti-4aa
f1 (ppm)

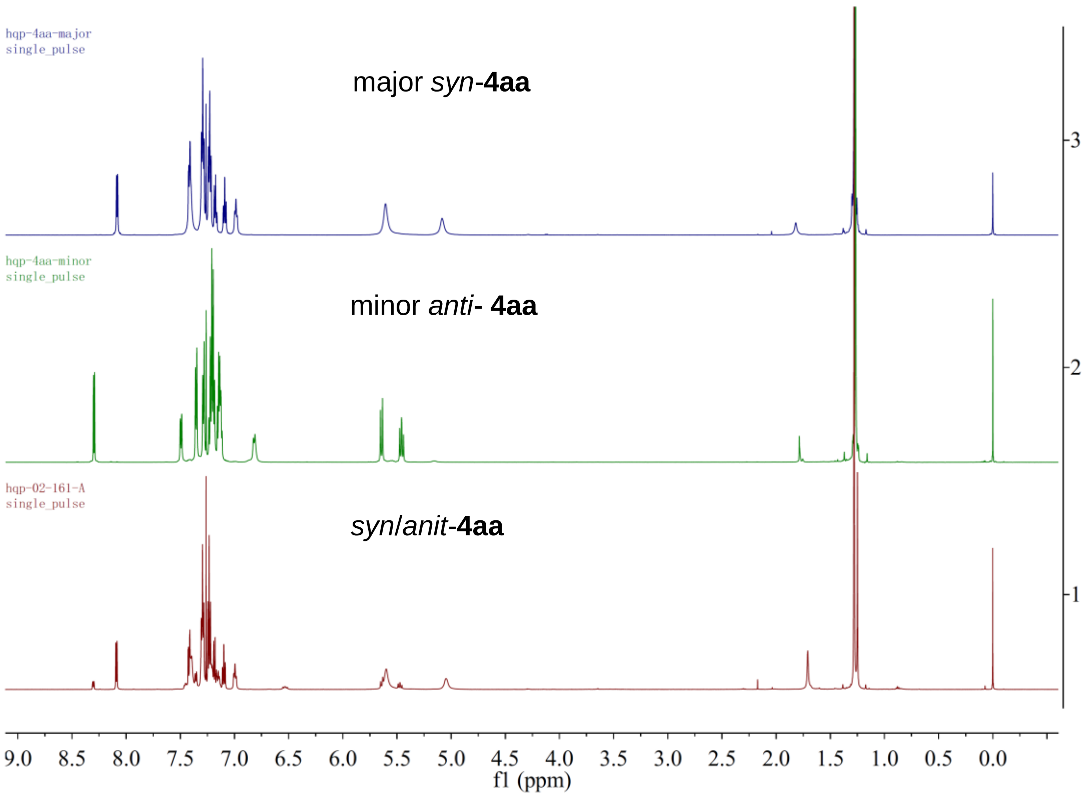
hqp-4aa-major
single_pulse
major *syn*-**4aa**
hqp-4aa-minor
single_pulse
minor *anti*- **4aa**
hqp-02-161-A
single_pulse
syn/*anit*-**4aa**
3
2
1
9.0 8.5 8.0 7.5 7.0 6.5 6.0 5.5 5.0 4.5 4.0 3.5 3.0 2.5 2.0 1.5 1.0 0.5 0.0
f1 (ppm)

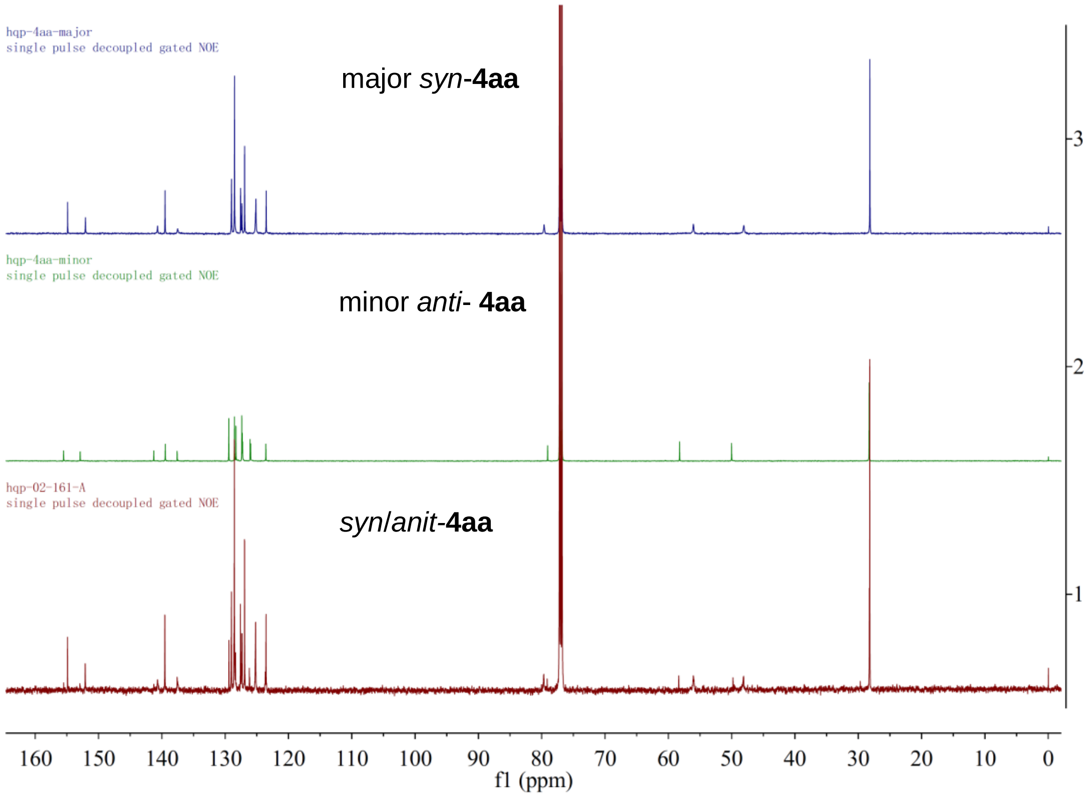
hqp-4aa-major
single pulse decoupled gated NOE
major *syn*-**4aa**
hqp-4aa-minor
single pulse decoupled gated NOE
minor *anti*- **4aa**
hqp-02-161-A
single pulse decoupled gated NOE
syn/*anit*-**4aa**
3
2
1
160 150 140 130 120 110 100 90 80 70 60 50 40 30 20 10 0
f1 (ppm)

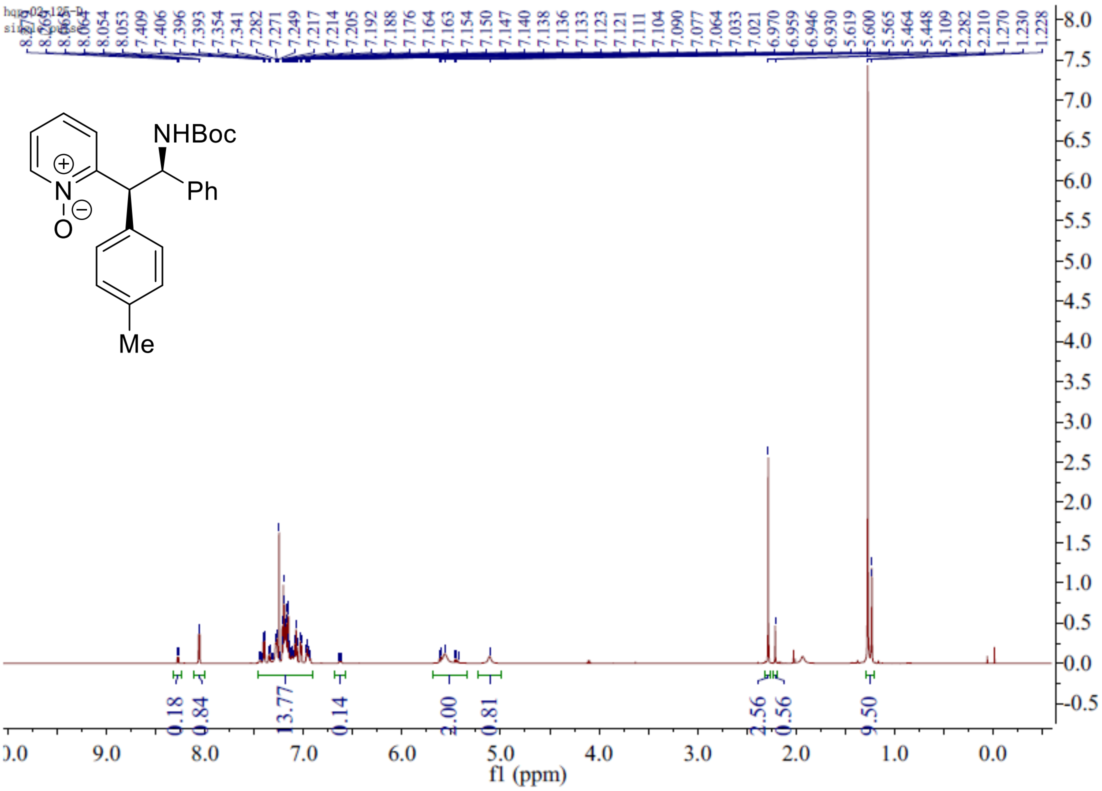
hqp-02-125-D
NHBoc
Ph
N
O
Me
f1 (ppm)

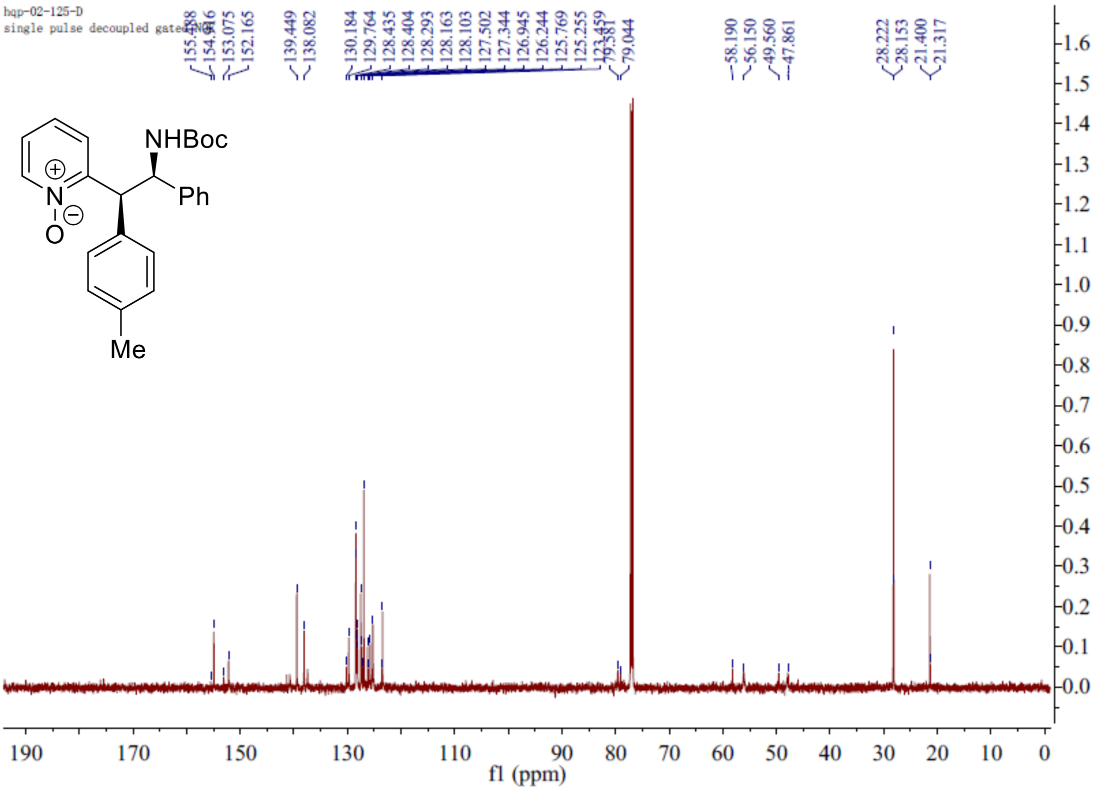
hqp-02-125-D
single pulse decoupled gated NOE
155.388
153.075
152.165
139.449
138.082
130.184
129.764
128.435
128.404
128.293
128.163
128.103
127.502
127.344
126.945
126.244
125.769
125.255
79.044
58.190
56.150
49.560
47.861
28.222
28.153
21.400
21.317
NHBoc
Ph
N
O
Me
f1 (ppm)

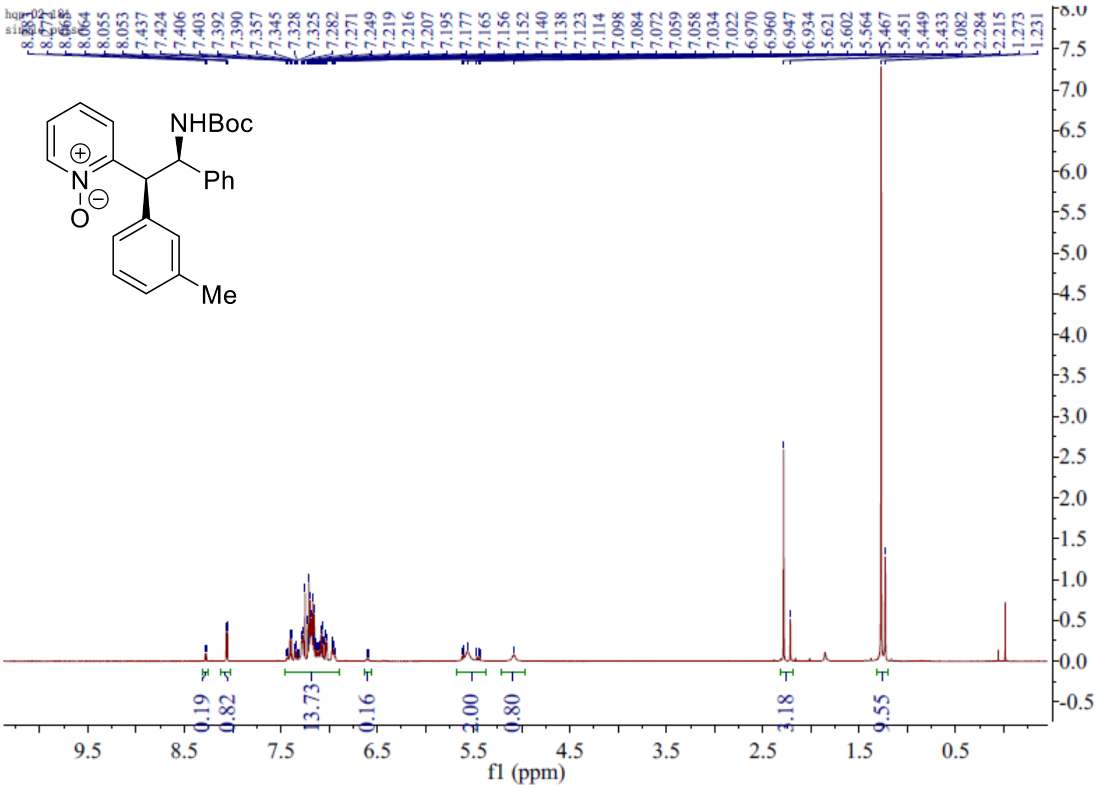

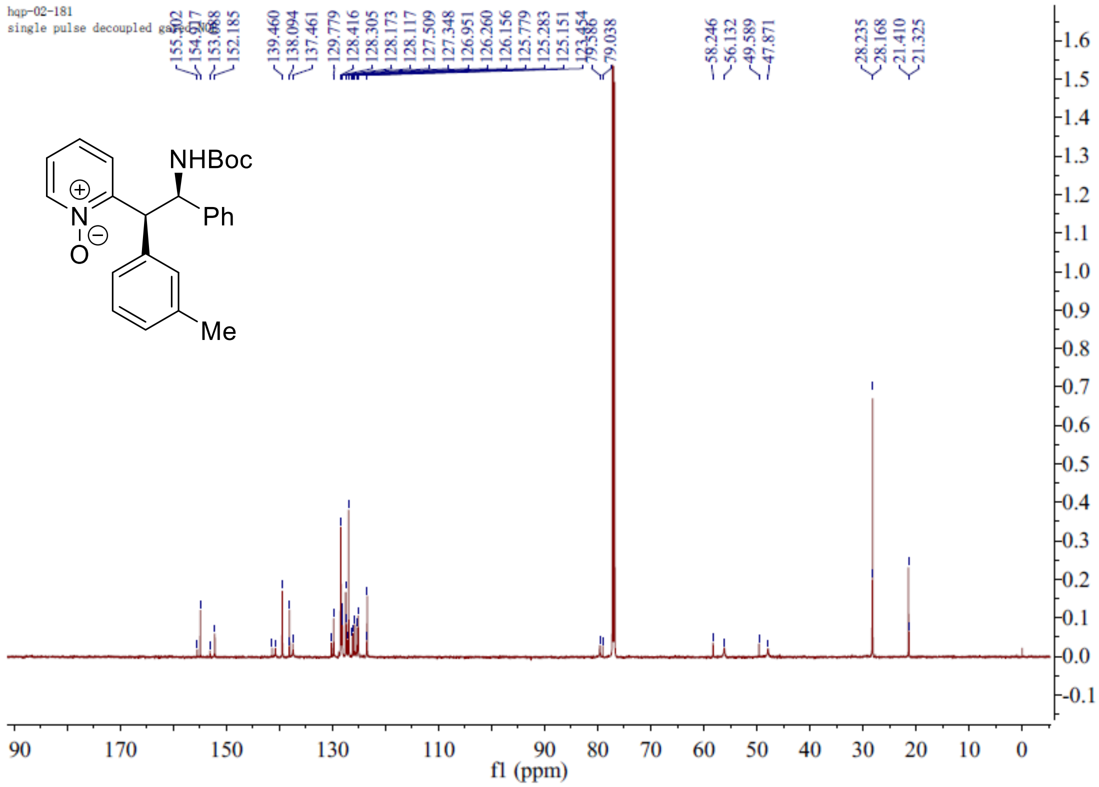

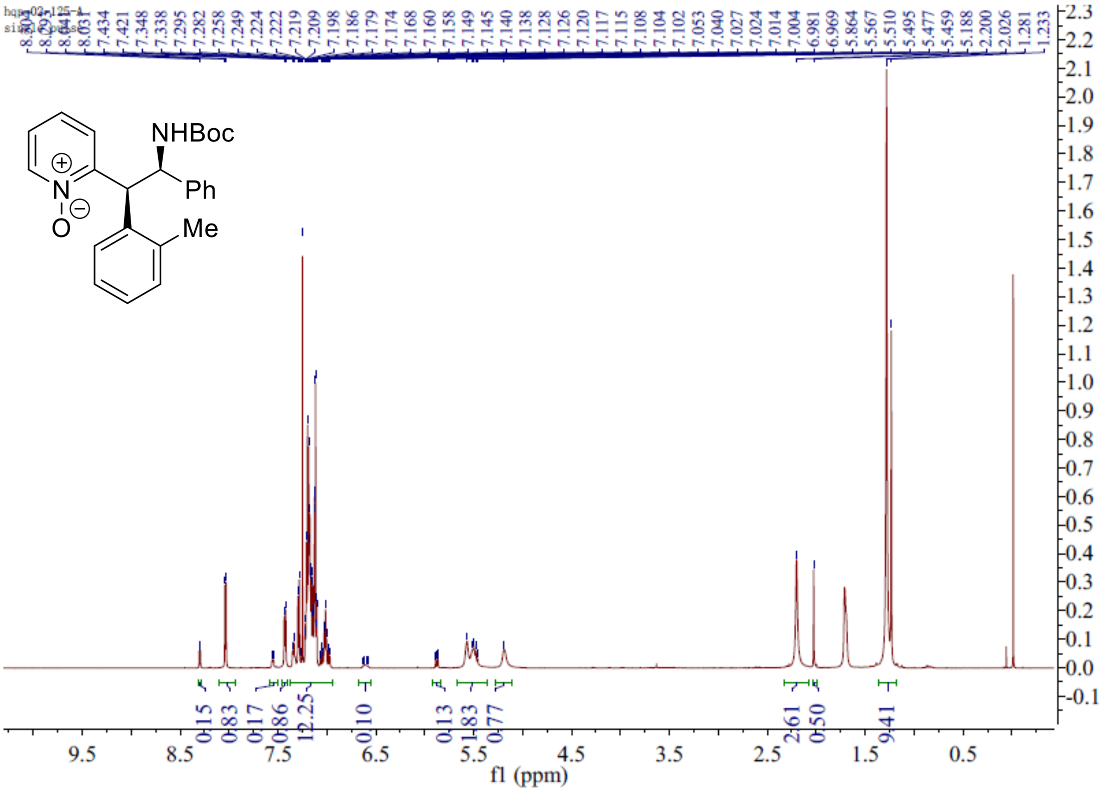
hqp-02-125-A
NHBoc
Ph
Me
N
O
f1 (ppm)

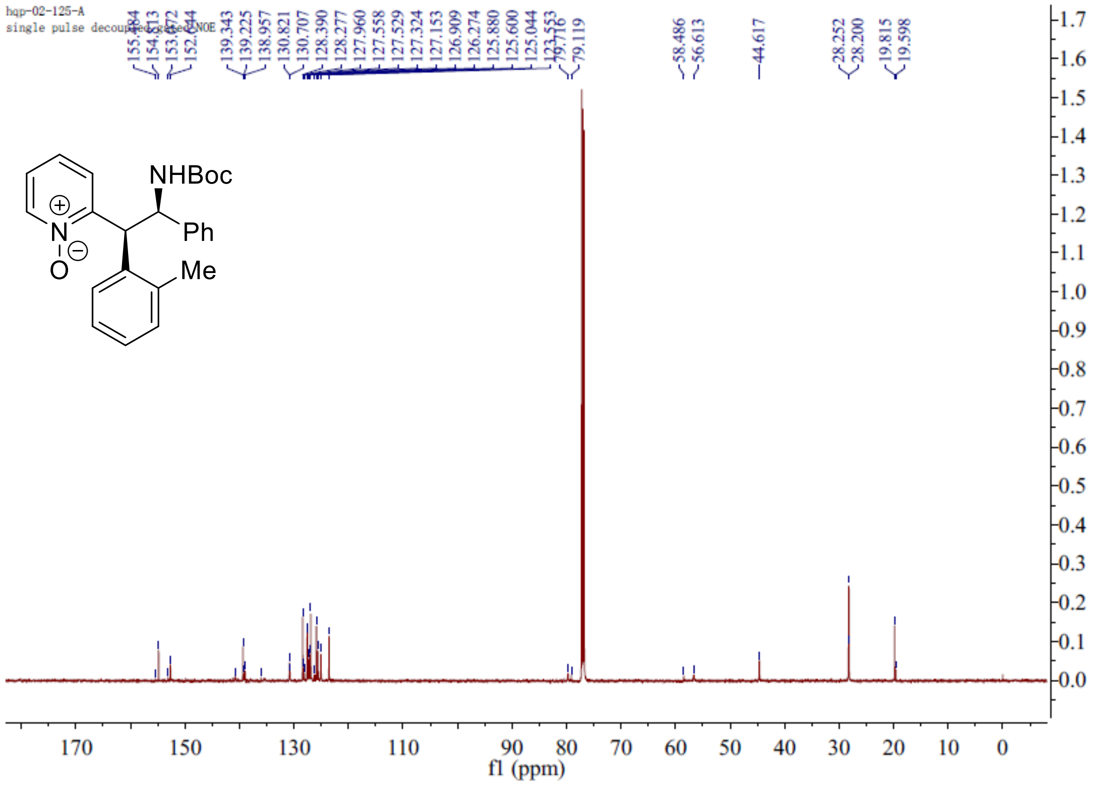
hqp-02-125-A
NHBoc
Ph
Me
N
O
f1 (ppm)

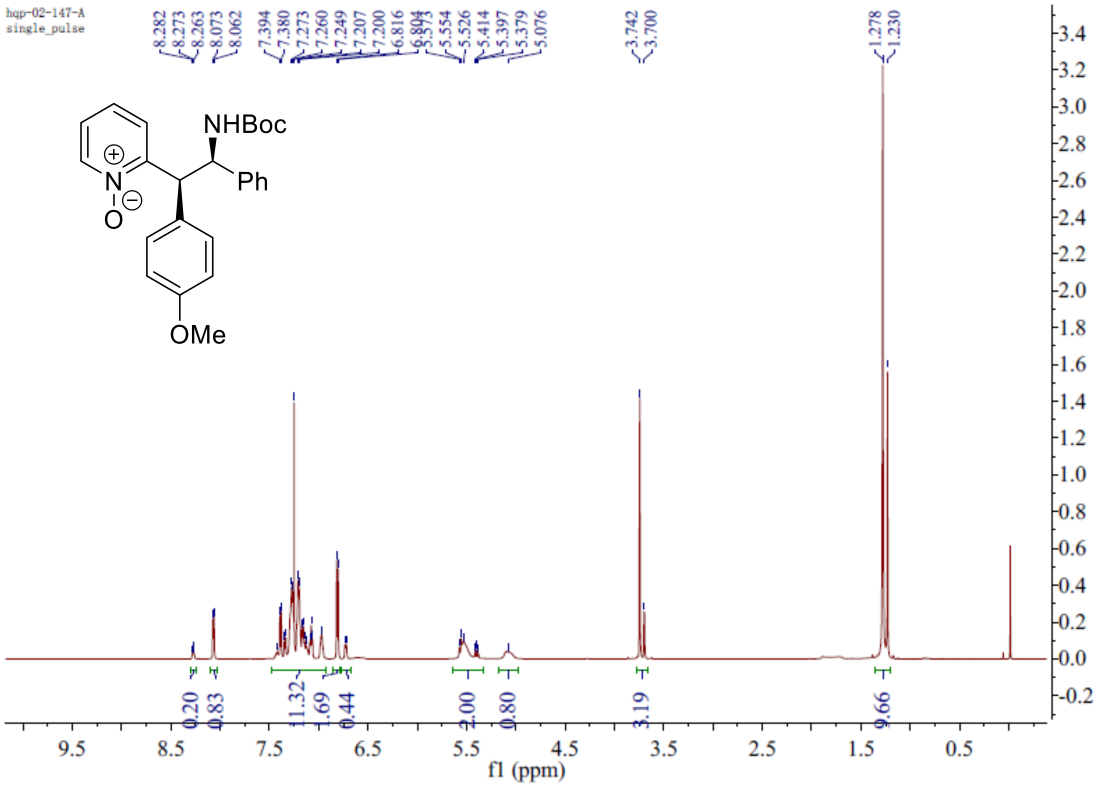
hqp-02-147-A
single_pulse
8.282
8.273
8.263
8.073
8.062
7.394
7.380
7.273
7.260
7.249
7.207
7.200
6.816
5.554
5.526
5.414
5.397
5.379
5.076
3.742
3.700
1.278
1.230
NHBoc
Ph
O
OMe
0.20
0.83
11.32
1.69
0.44
2.00
0.80
3.19
9.66
f1 (ppm)

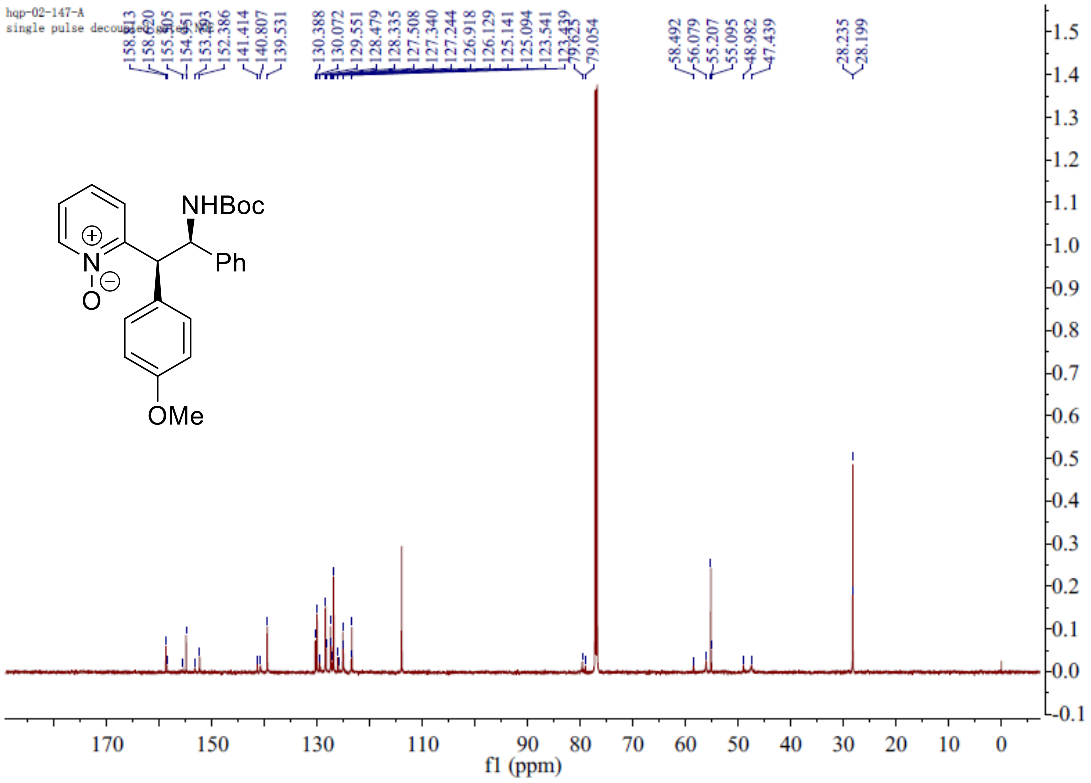
hqp-02-147-A
158.620
155.305
152.386
141.414
140.807
139.531
130.388
130.072
129.551
128.479
128.335
127.508
127.340
127.244
126.918
126.129
125.141
125.094
123.541
79.054
58.492
56.079
55.207
55.095
48.982
47.439
28.235
28.199
NHBoc
Ph
O
OMe
f1 (ppm)

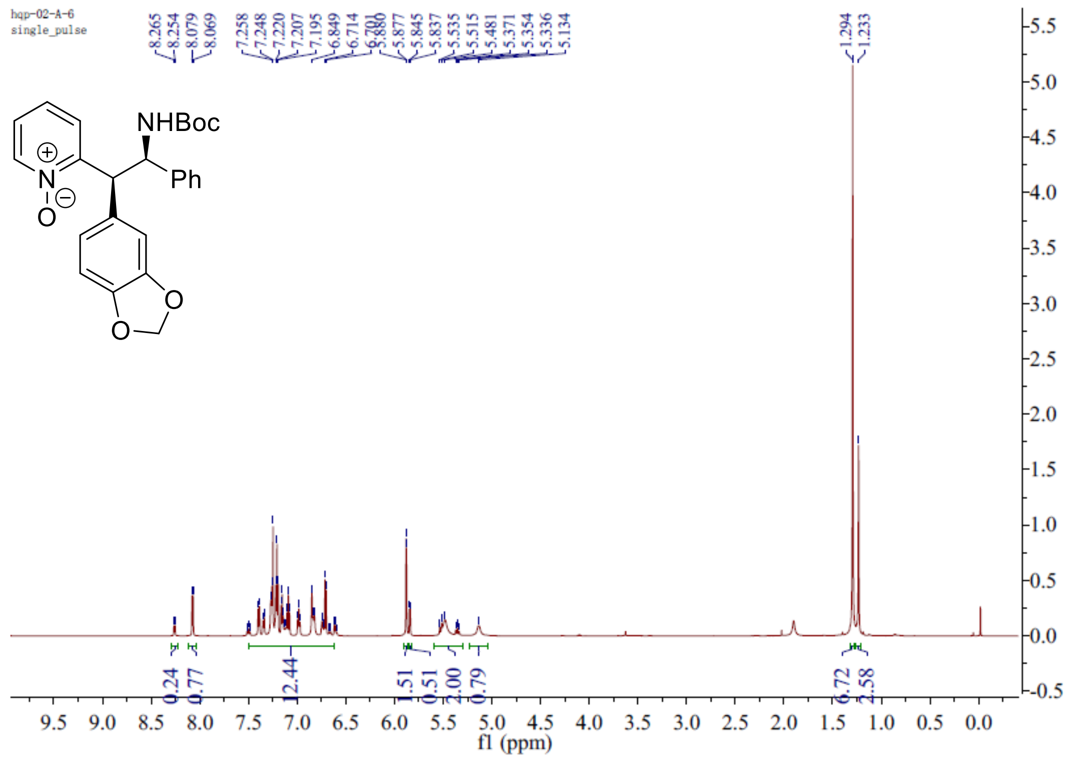

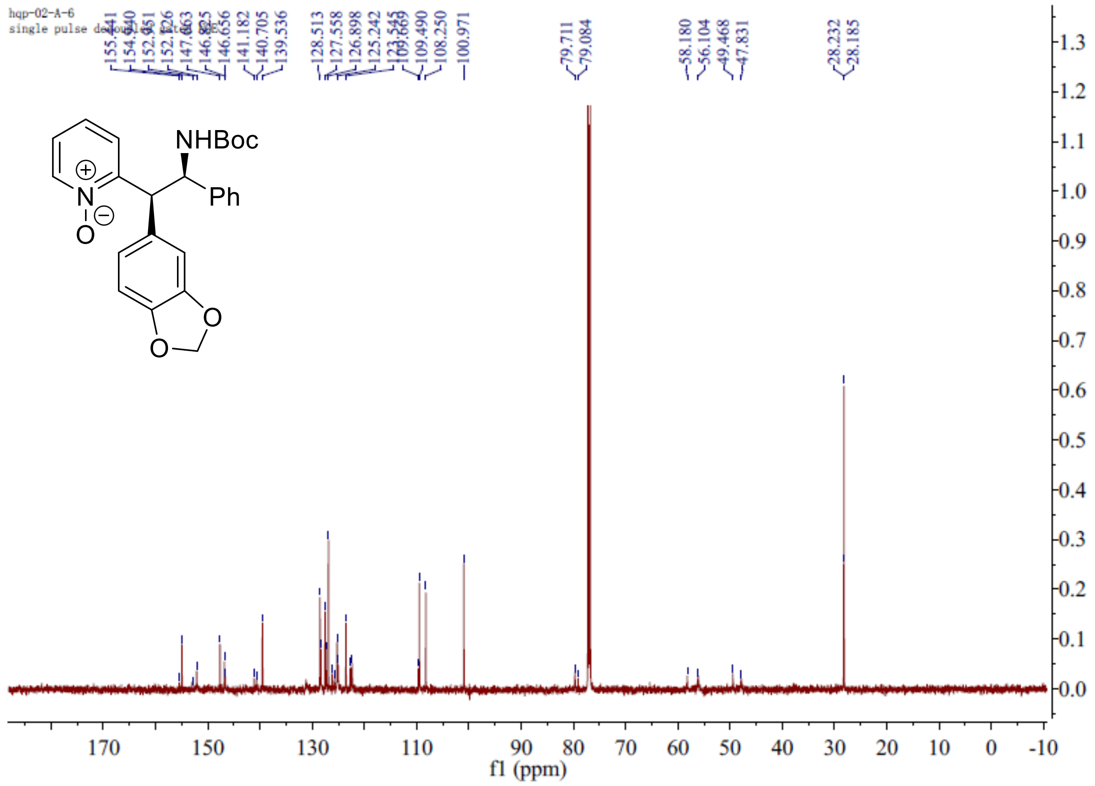

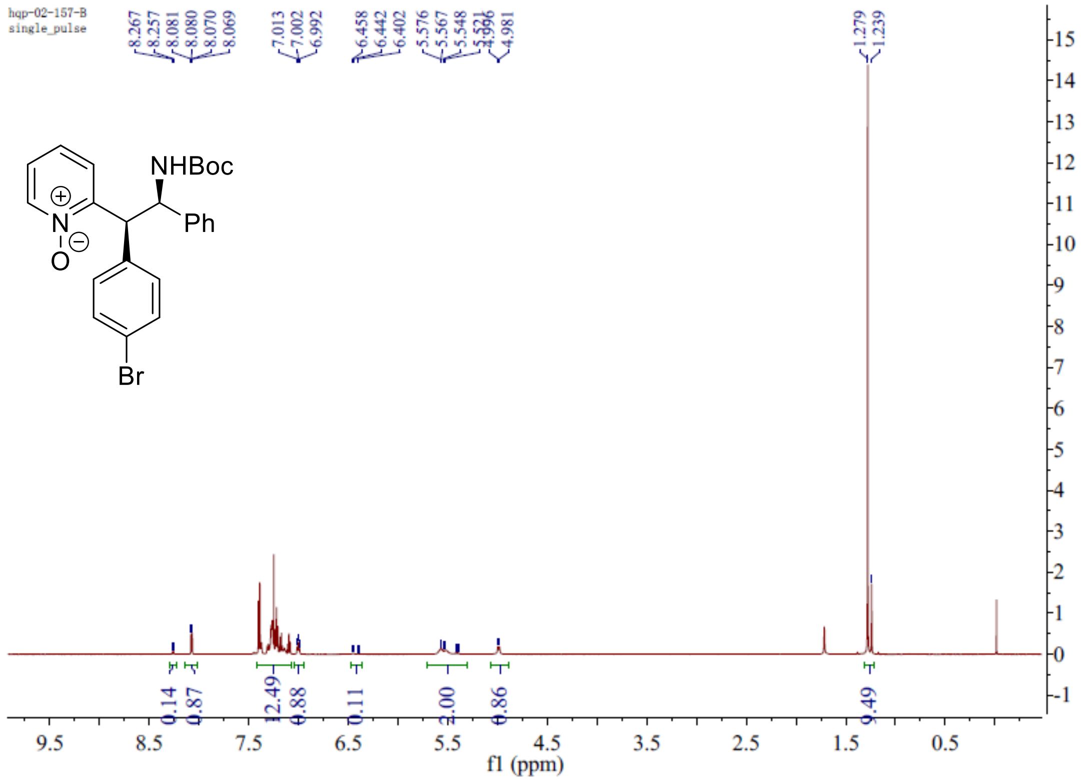
hqp-02-157-B
single_pulse
8.267
8.257
8.081
8.080
8.070
8.069
7.013
7.002
6.992
6.458
6.442
6.402
5.576
5.567
5.548
4.981
1.279
1.239
NHBoc
Ph
N
O
Br
0.14
0.87
12.49
0.88
0.11
2.00
0.86
9.49
f1 (ppm)

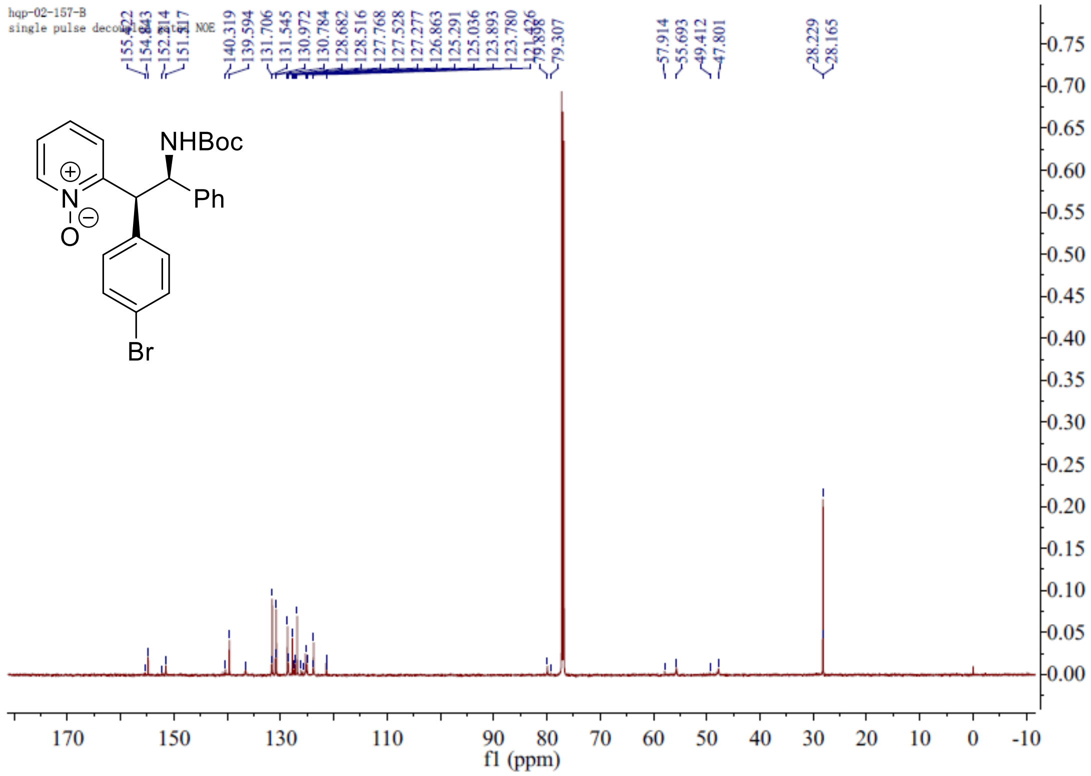
hqp-02-157-B
154.843
140.319
139.594
131.706
131.545
130.972
130.784
128.682
128.516
127.768
127.528
127.277
126.863
125.291
125.036
123.893
123.780
79.307
57.914
55.693
49.412
47.801
28.229
28.165
NHBoc
Ph
N
O
Br
f1 (ppm)

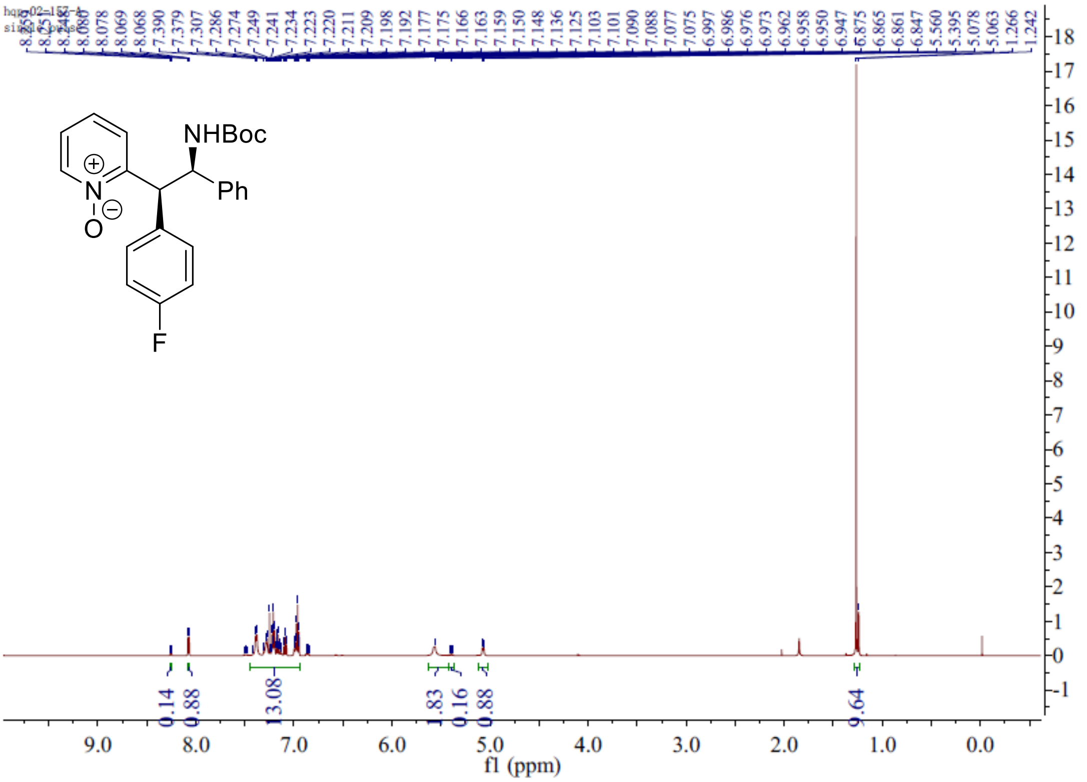
NHBoc
Ph
N
O
F
9.0
8.0
7.0
6.0
5.0
4.0
3.0
2.0
1.0
0.0
f1 (ppm)

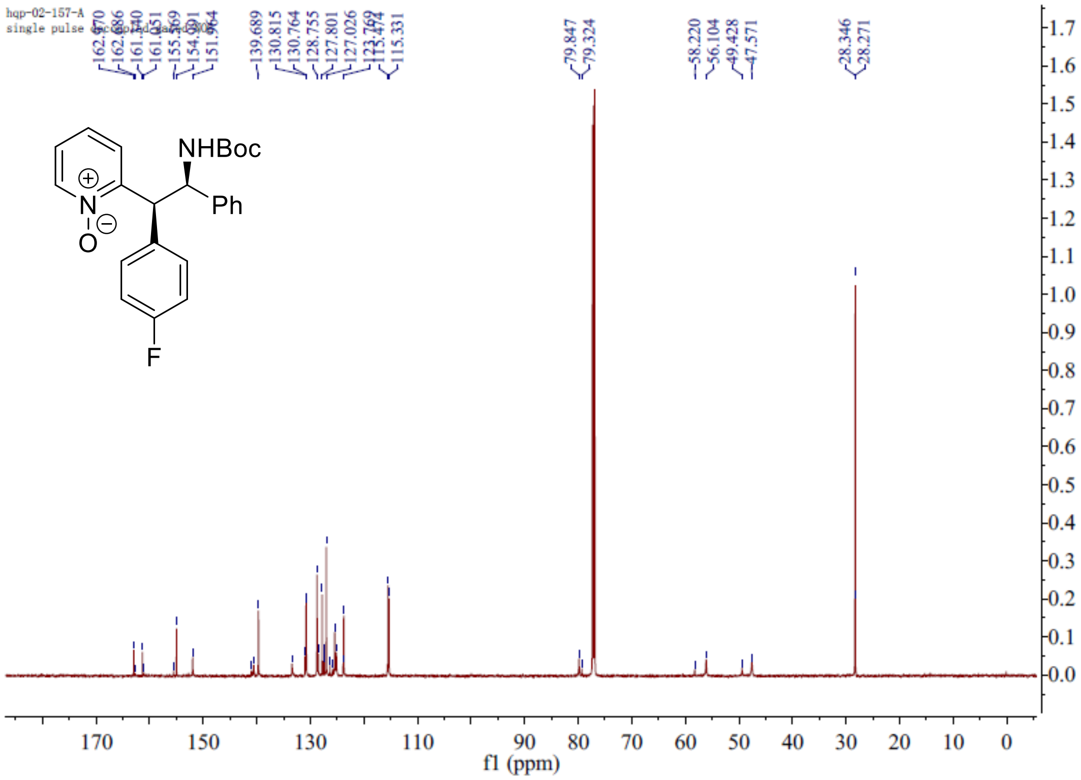
hqp-02-157-A
NHBoc
Ph
N
O
F
139.689
130.815
130.764
128.755
127.801
127.026
115.331
79.847
79.324
58.220
56.104
49.428
47.571
28.346
28.271
170
150
130
110
90
80
70
60
50
40
30
20
10
0
f1 (ppm)

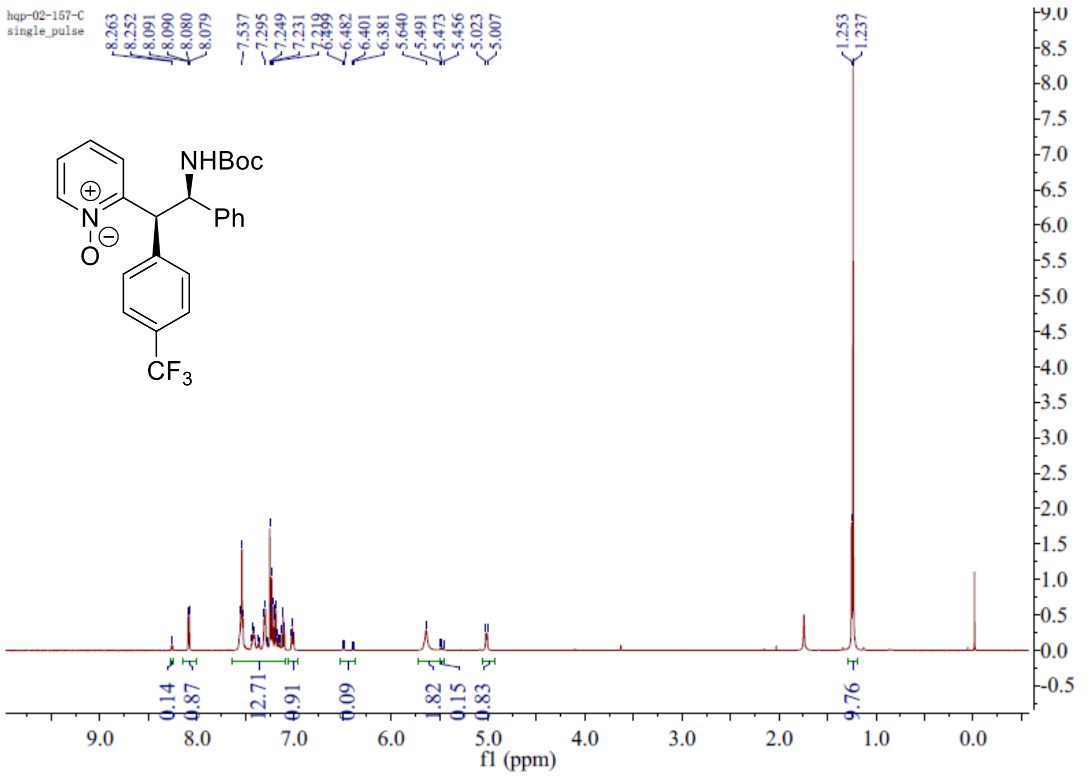
hqp-02-157-C
single_pulse
8.263
8.252
8.091
8.090
8.080
8.079
7.537
7.295
7.249
7.231
6.482
6.401
6.381
5.640
5.491
5.473
5.456
5.023
5.007
1.253
1.237
NHBoc
Ph
N
O
CF3
0.14
0.87
12.71
0.91
0.09
1.82
0.15
0.83
9.76
f1 (ppm)

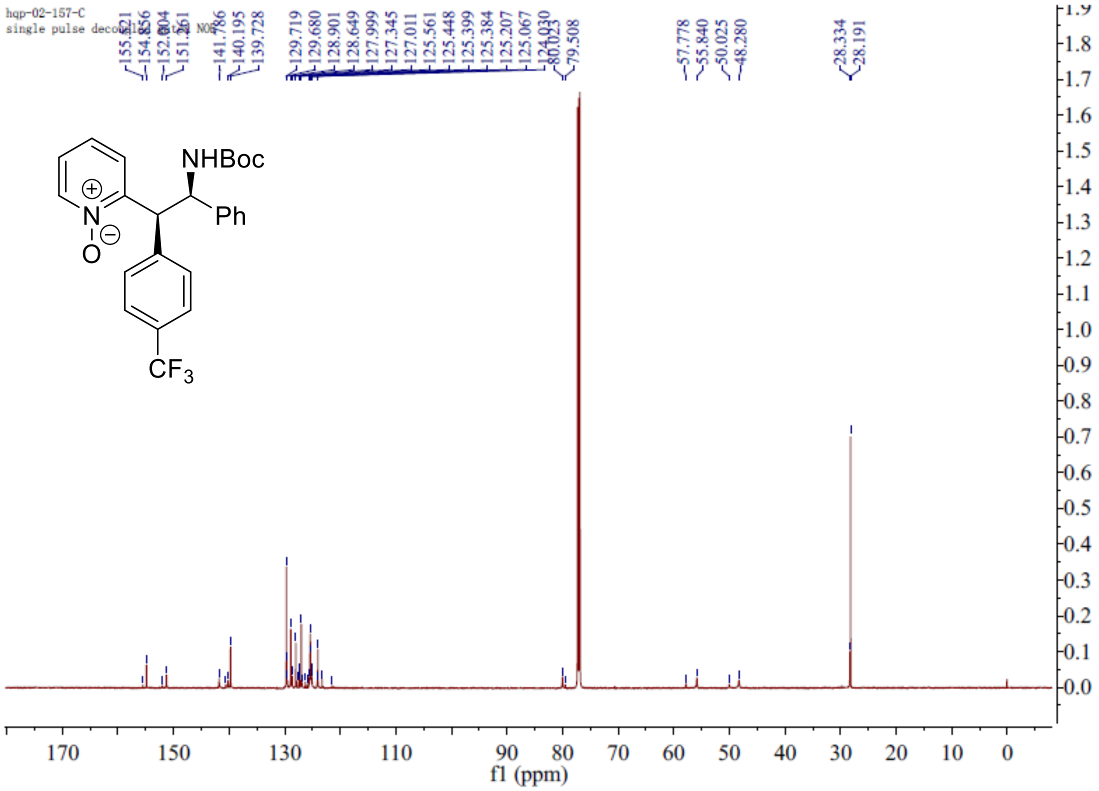
hqp-02-157-C
155.021
141.786
140.195
139.728
129.719
129.680
128.901
128.649
127.999
127.345
127.011
125.561
125.448
125.399
125.384
125.207
125.067
80.023
79.508
57.778
55.840
50.025
48.280
28.334
28.191
NHBoc
Ph
N
O
CF3
f1 (ppm)

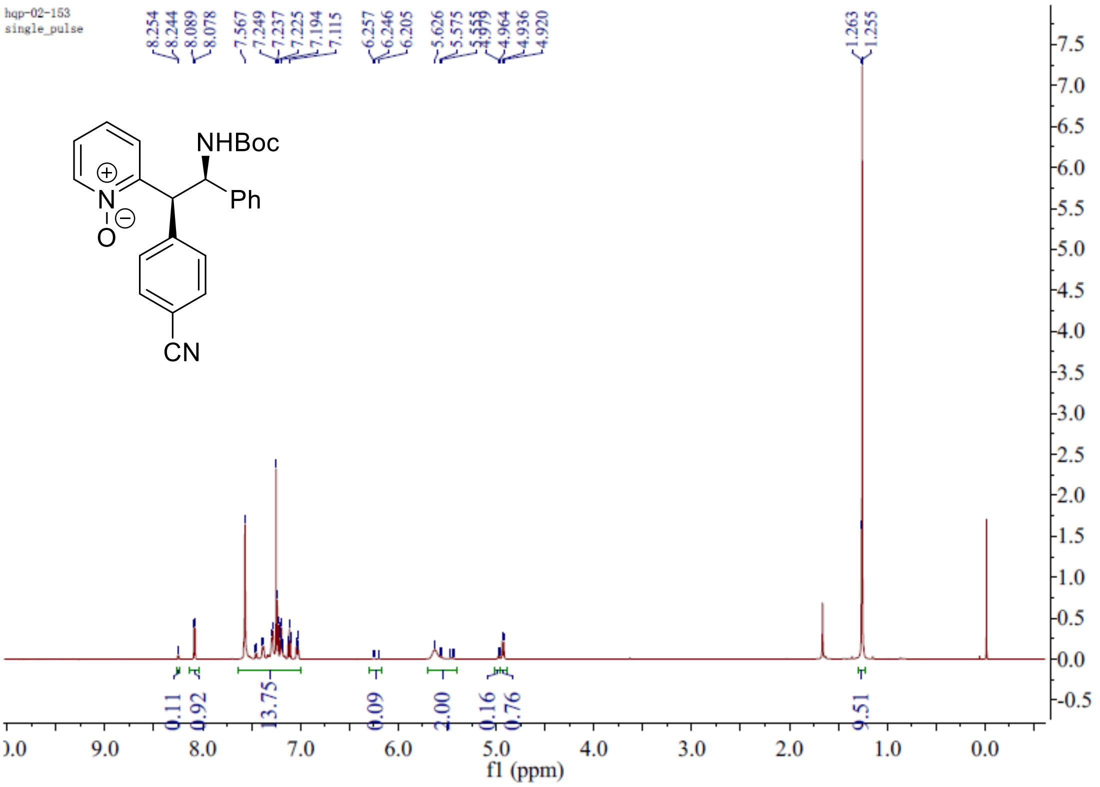
hqp-02-153
single_pulse
8.254
8.244
8.089
8.078
7.567
7.249
7.237
7.225
7.194
7.115
6.257
6.246
6.205
5.626
5.575
4.964
4.936
4.920
1.263
1.255
NHBoc
Ph
N
O
CN
0.11
0.92
13.75
0.09
2.00
0.16
0.76
9.51
f1 (ppm)

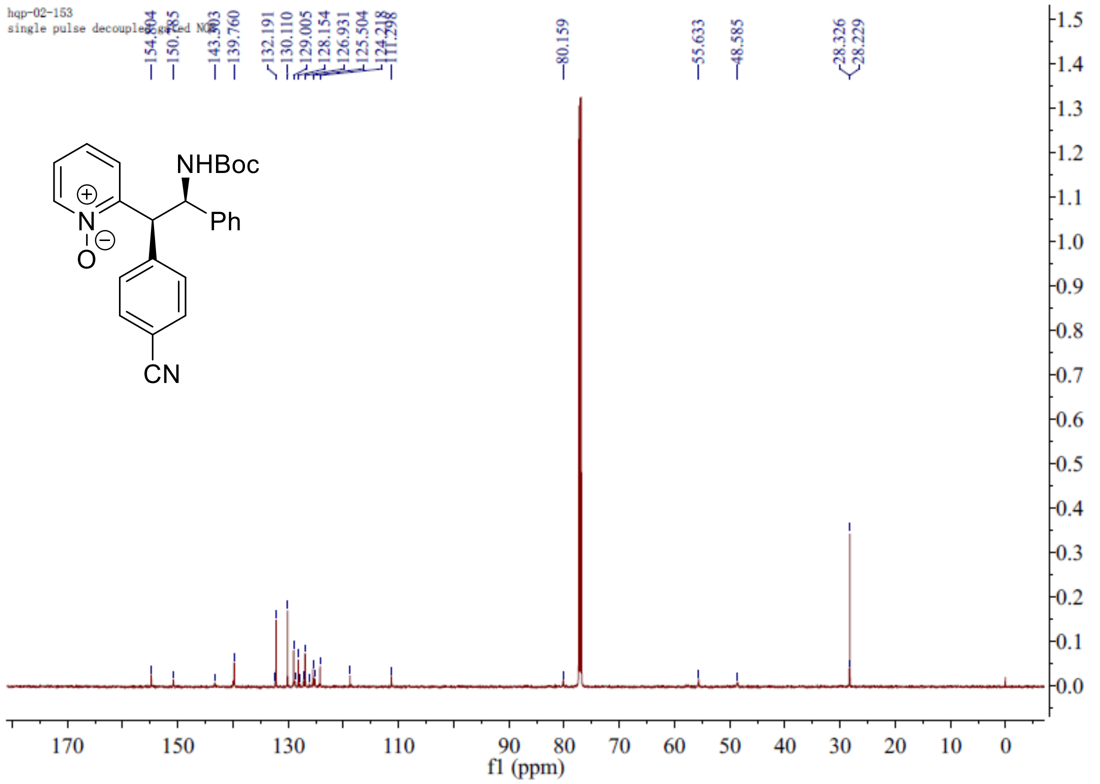
hqp-02-153
single pulse decoupled gated NOE
139.760
132.191
130.110
129.005
128.154
126.931
125.504
80.159
55.633
48.585
28.326
28.229
NHBoc
Ph
N
O
CN
f1 (ppm)

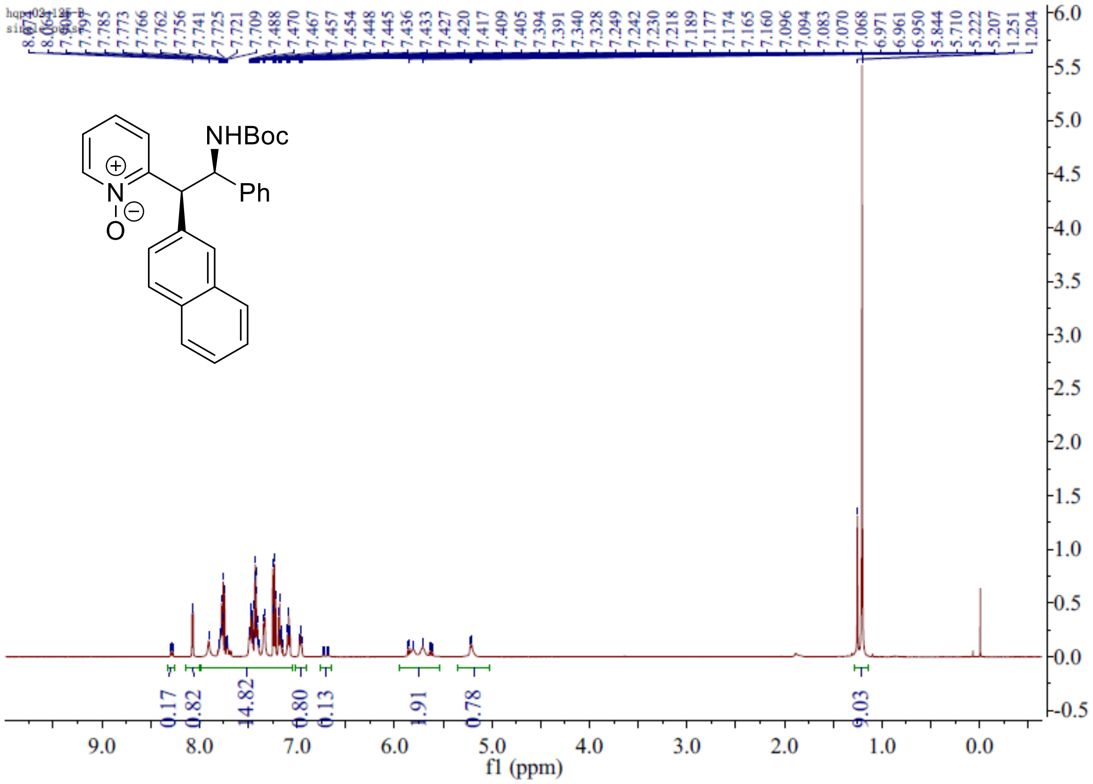

7.785
7.773
7.766
7.762
7.756
7.741
7.725
7.721
7.709
7.488
7.470
7.467
7.457
7.454
7.448
7.445
7.436
7.433
7.427
7.420
7.417
7.409
7.405
7.394
7.391
7.340
7.328
7.249
7.242
7.230
7.218
7.189
7.177
7.174
7.165
7.160
7.096
7.094
7.083
7.070
7.068
6.971
6.961
6.950
5.844
5.710
5.222
5.207
1.251
1.204
NHBoc
Ph
N
O
0.17
0.82
14.82
0.80
0.13
1.91
0.78
9.03
f1 (ppm)

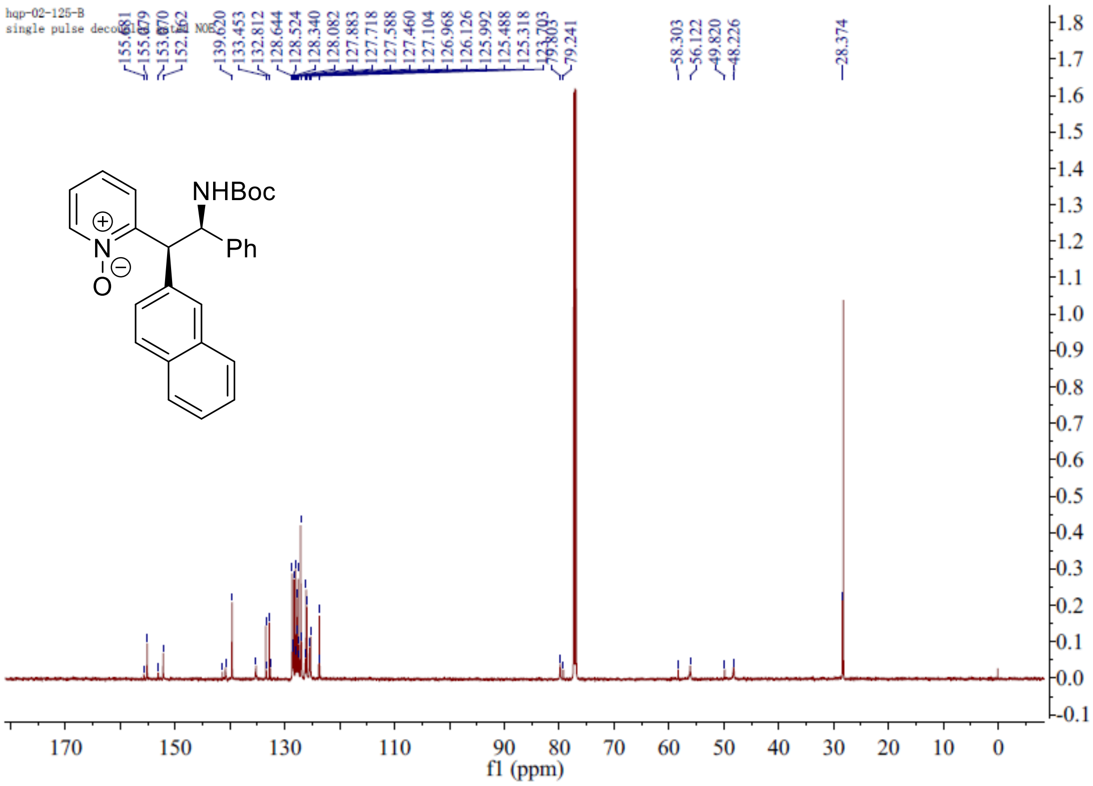

hqp-02-125-B
139.620
133.453
132.812
128.644
128.524
128.340
128.082
127.883
127.718
127.588
127.460
127.104
126.968
126.126
125.992
125.488
125.318
79.241
58.303
56.122
49.820
48.226
28.374
NHBoc
Ph
N
O
f1 (ppm)

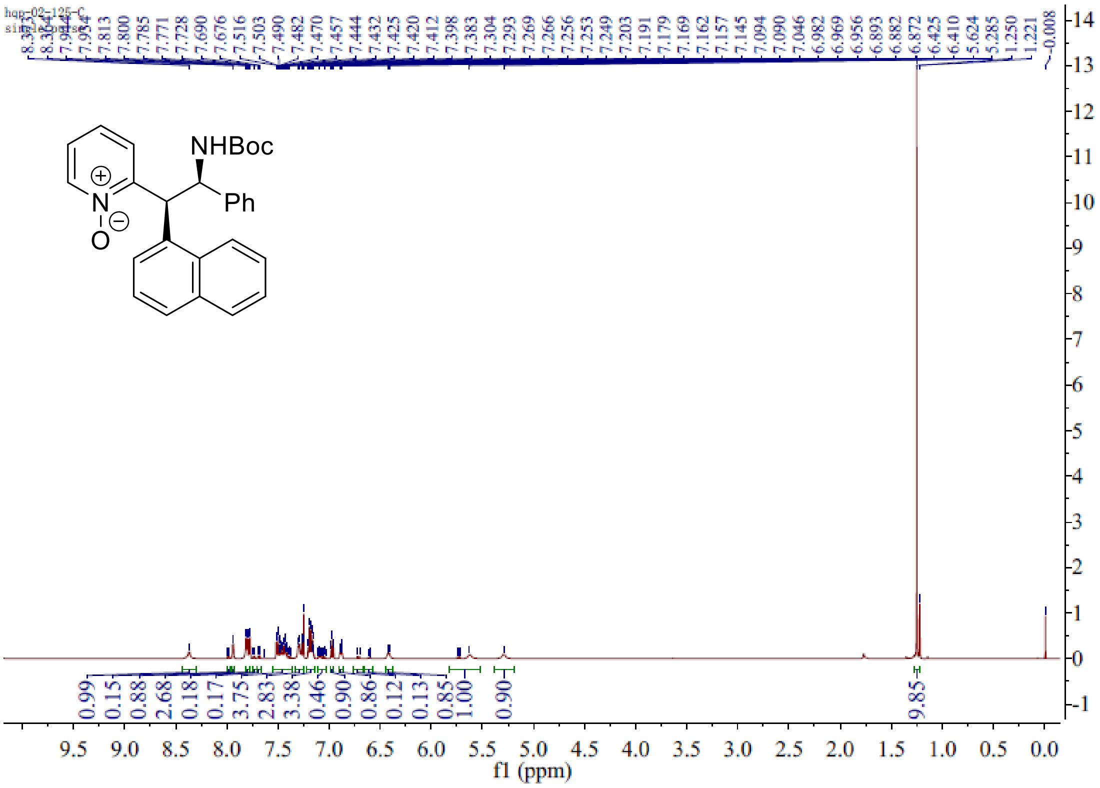

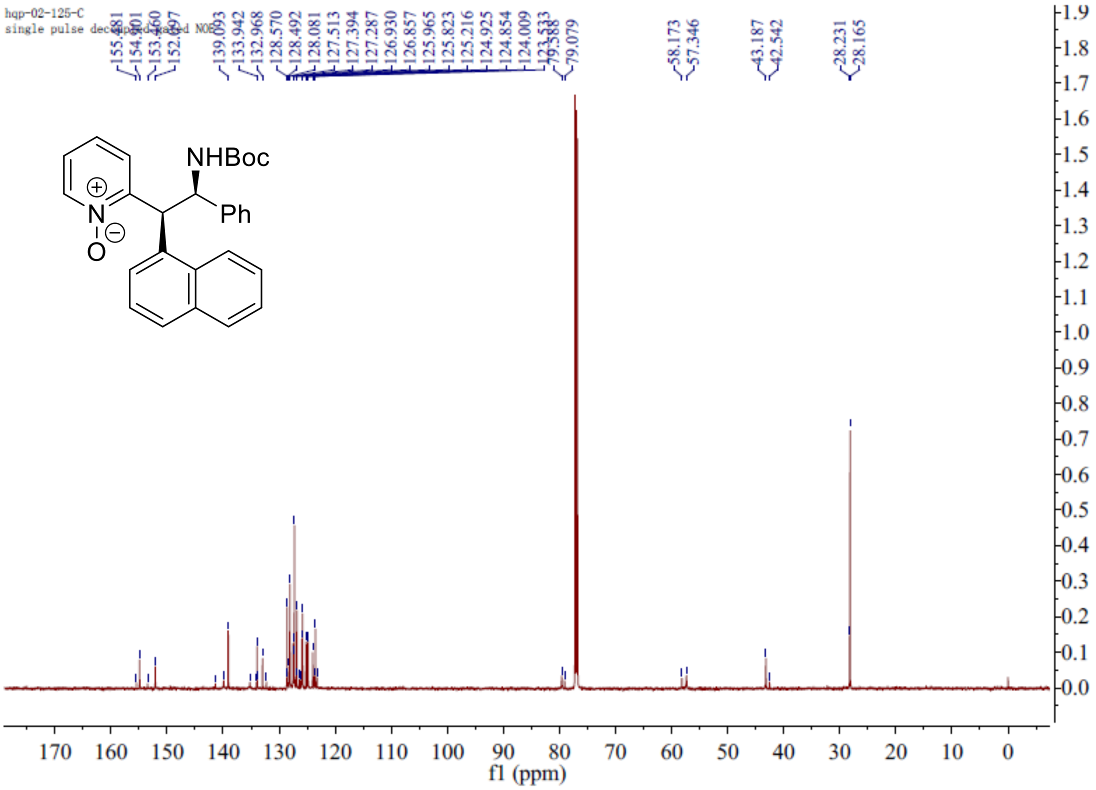

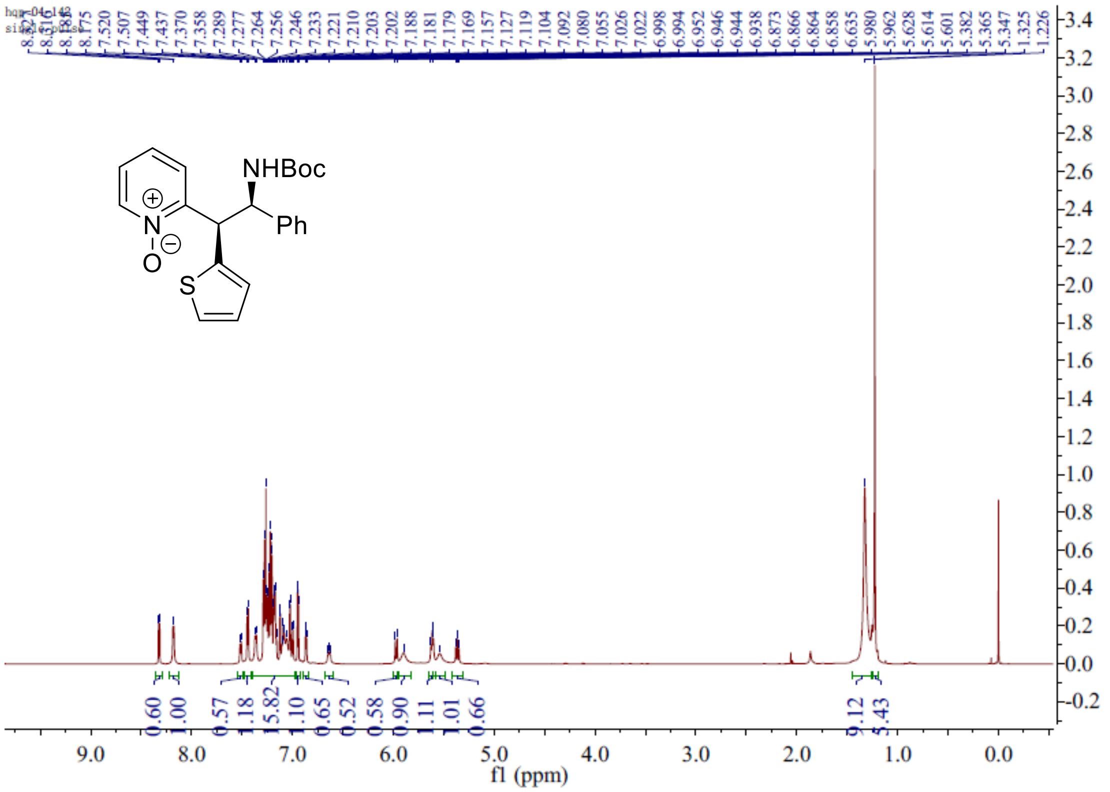
NHBoc
Ph
N
O
S
f1 (ppm)

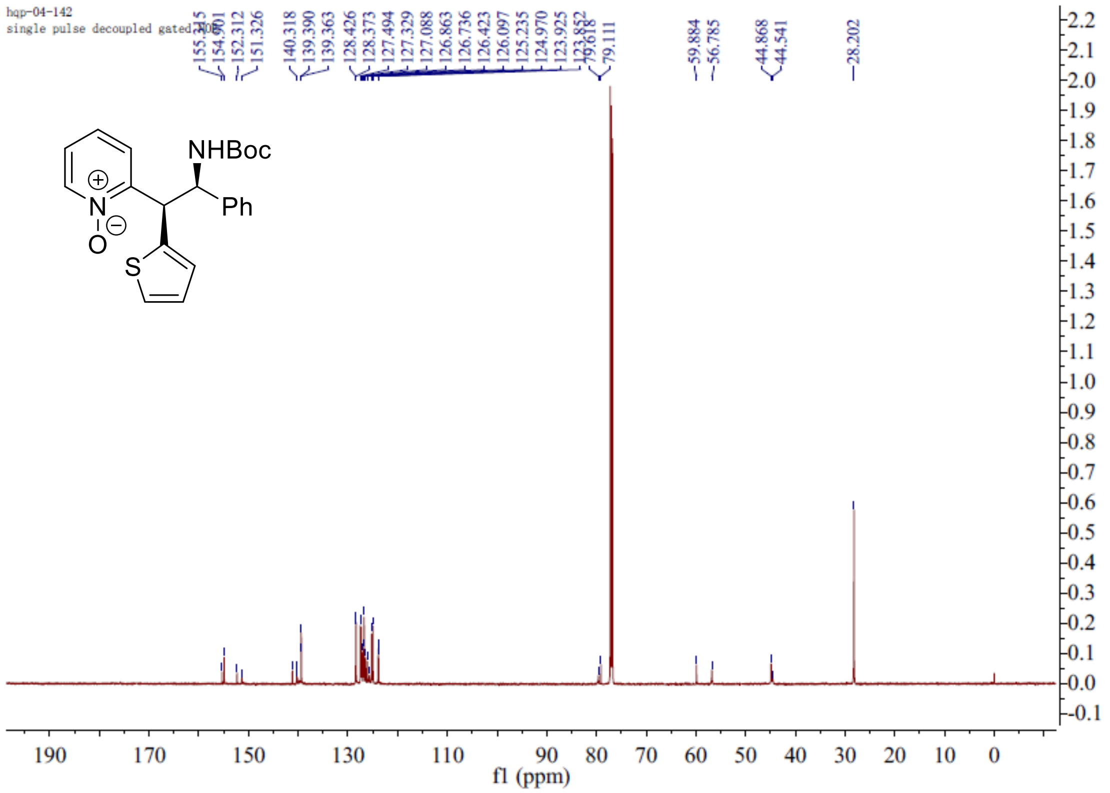
hqp-04-142
single pulse decoupled gated NOE
155.345
154.901
152.312
151.326
140.318
139.390
139.363
128.426
128.373
127.494
127.329
127.088
126.863
126.736
126.423
126.097
125.235
124.970
123.925
123.852
79.618
79.111
59.884
56.785
44.868
44.541
28.202
NHBoc
Ph
N
O
S
f1 (ppm)

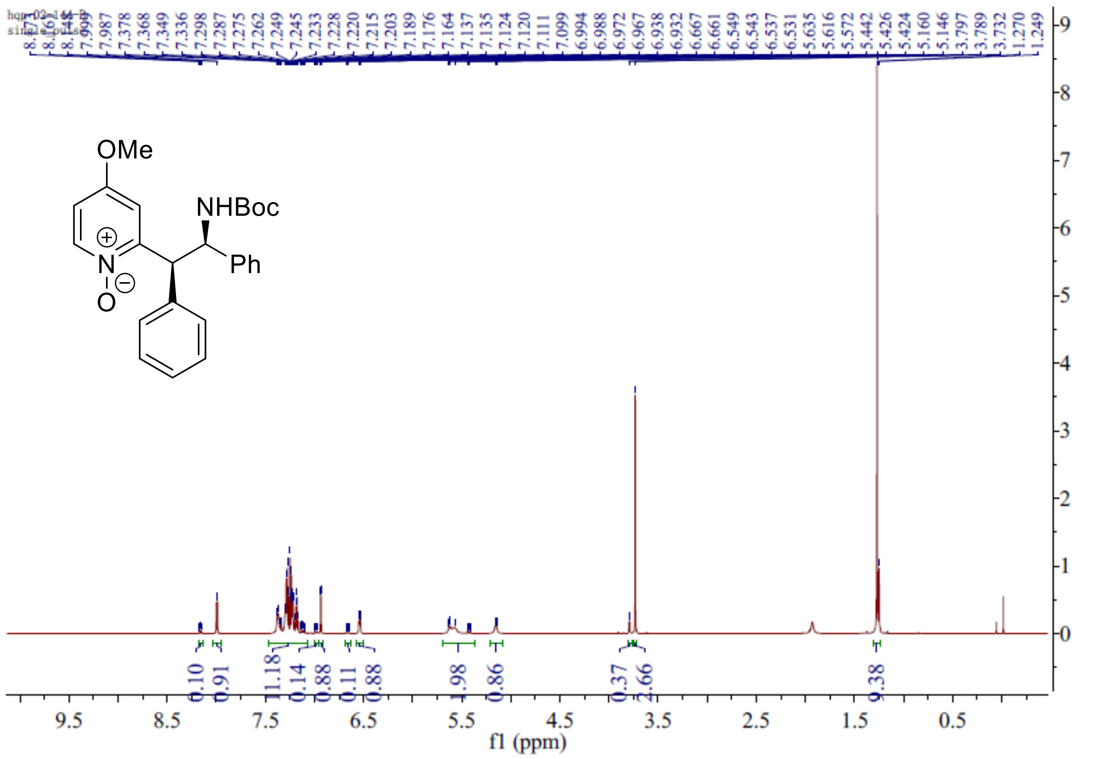

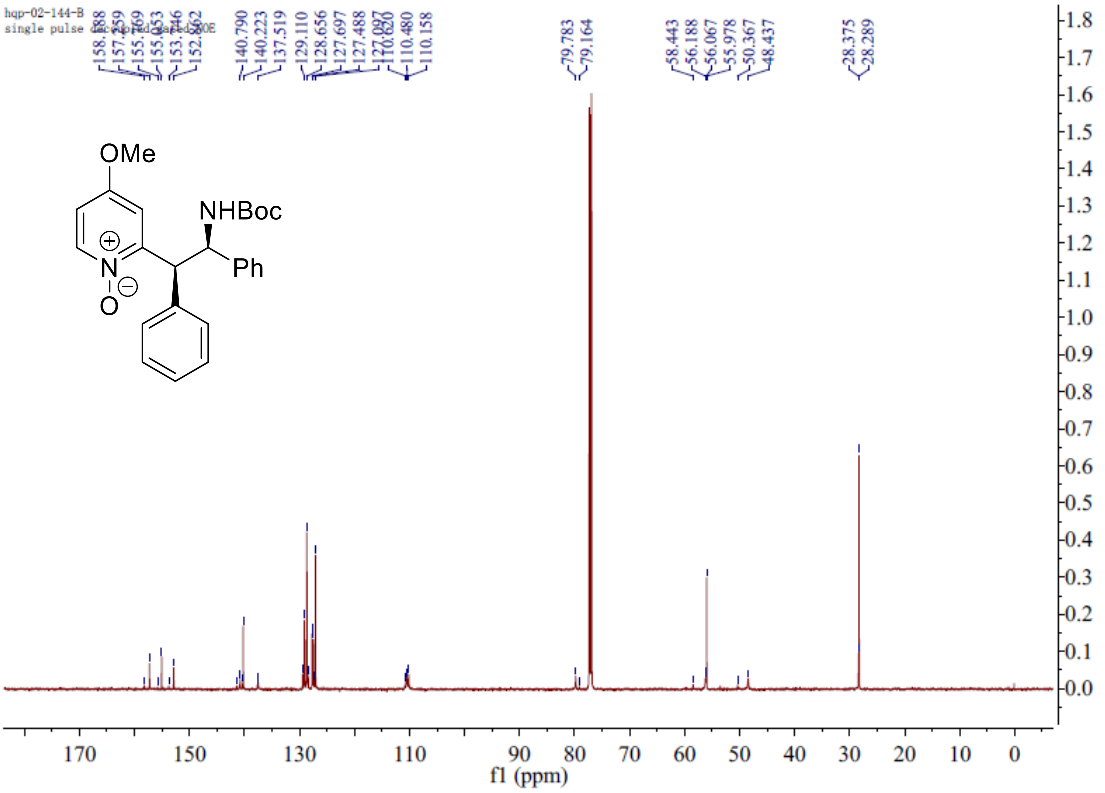

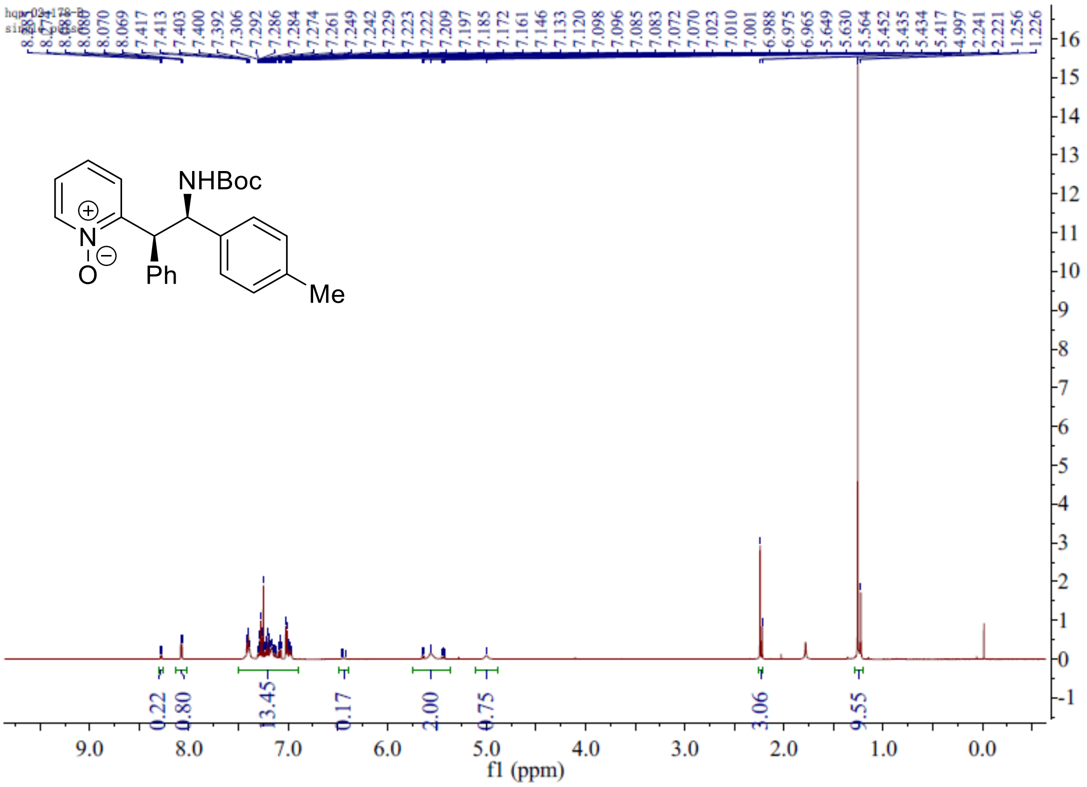

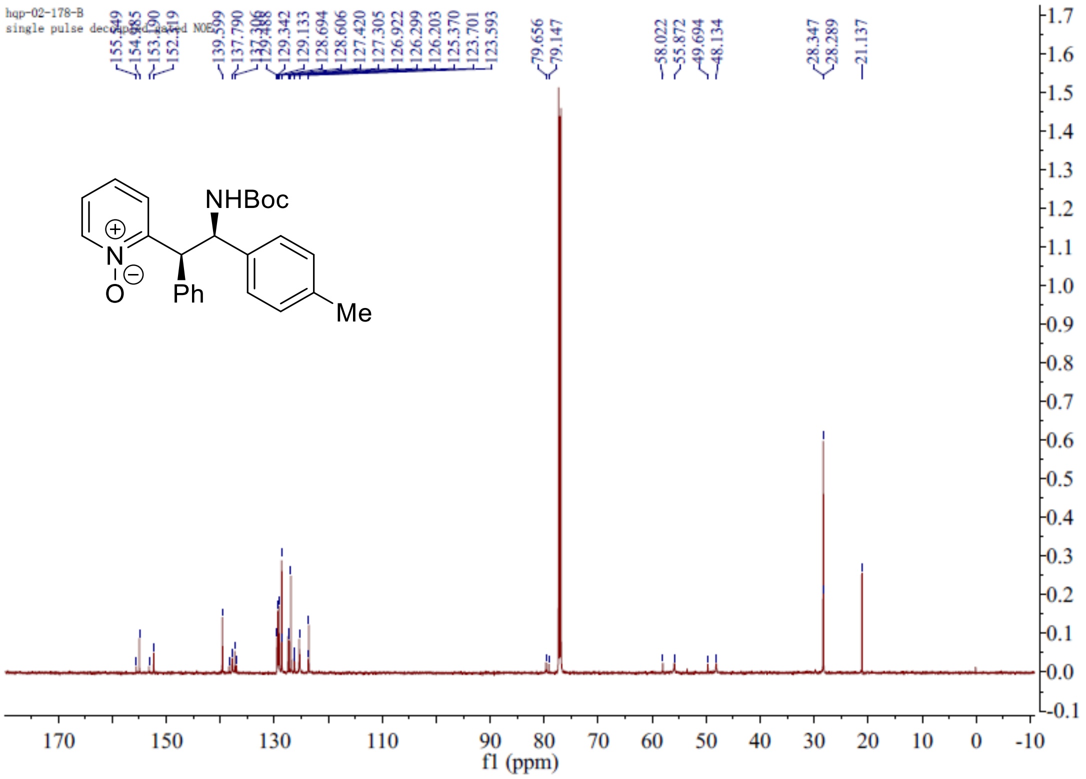

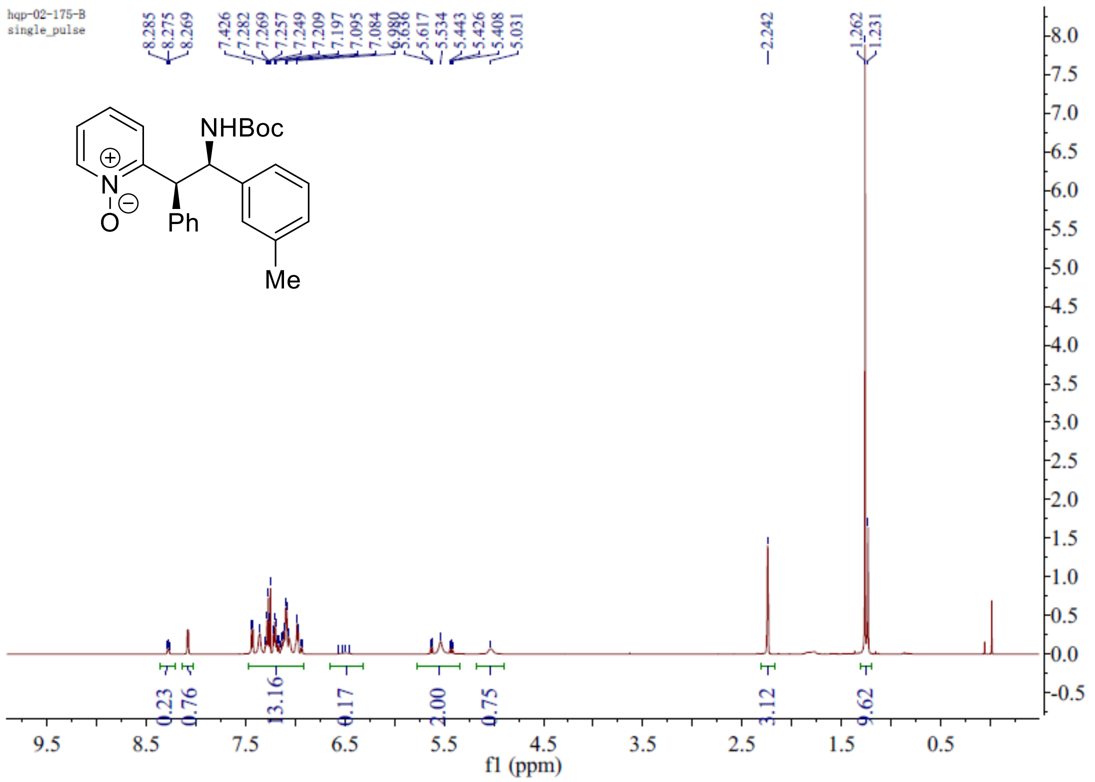
hqp-02-175-B
single_pulse
8.285
8.275
8.269
7.426
7.282
7.269
7.257
7.249
7.209
7.197
7.095
7.084
5.617
5.534
5.443
5.426
5.408
5.031
2.242
1.262
1.231
NHBoc
N
O
Ph
Me
0.23
0.76
13.16
2.00
0.75
3.12
9.62
f1 (ppm)

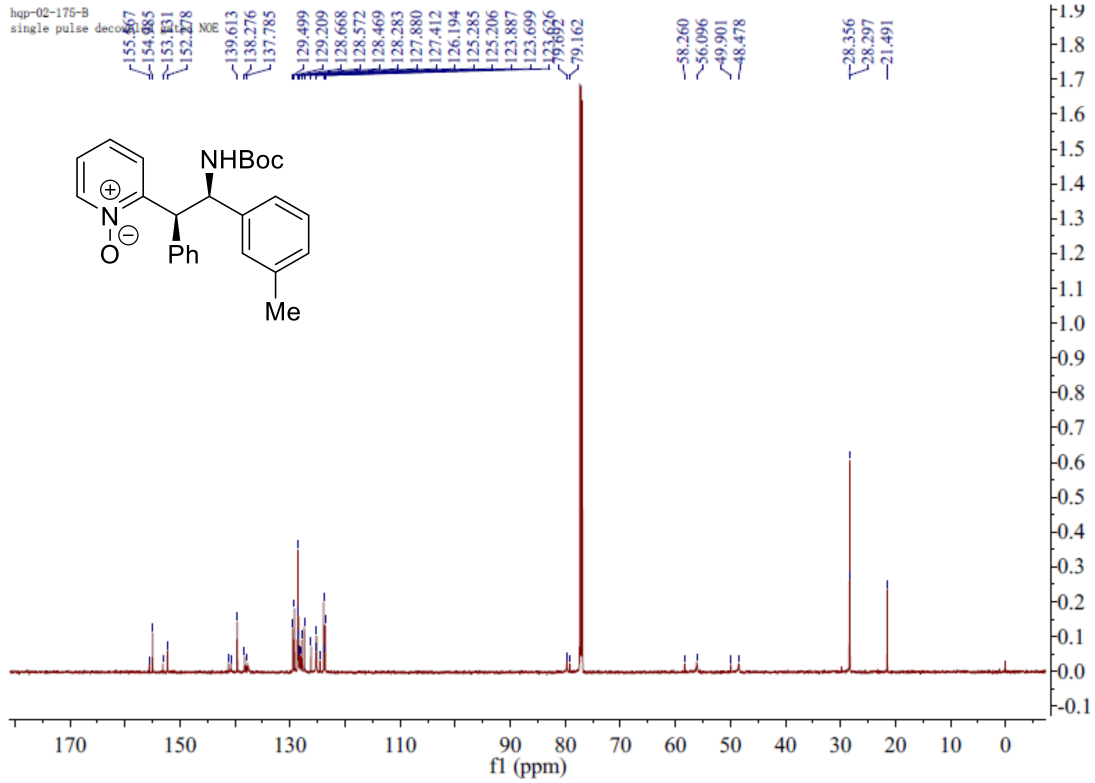
hqp-02-175-B
139.613
138.276
137.785
129.499
129.209
128.668
128.572
128.469
128.283
127.880
127.412
126.194
125.285
125.206
123.887
123.699
79.162
58.260
56.096
49.901
48.478
28.356
28.297
21.491
NHBoc
N
O
Ph
Me
f1 (ppm)

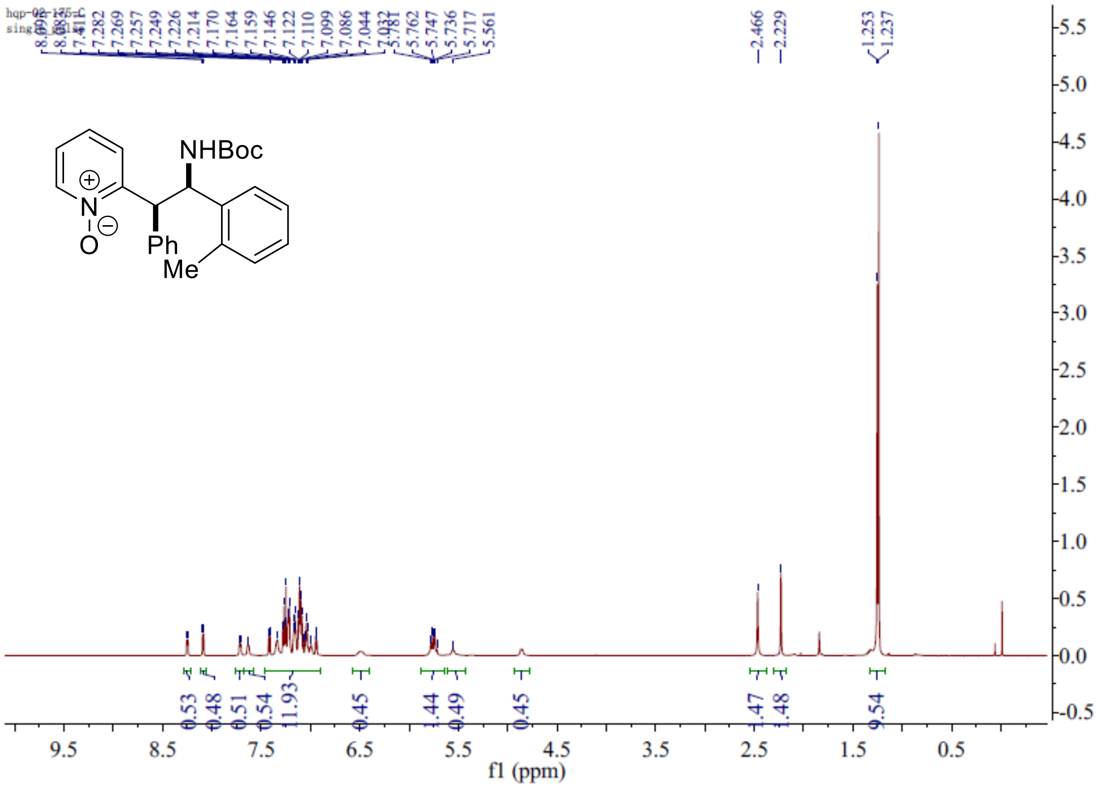

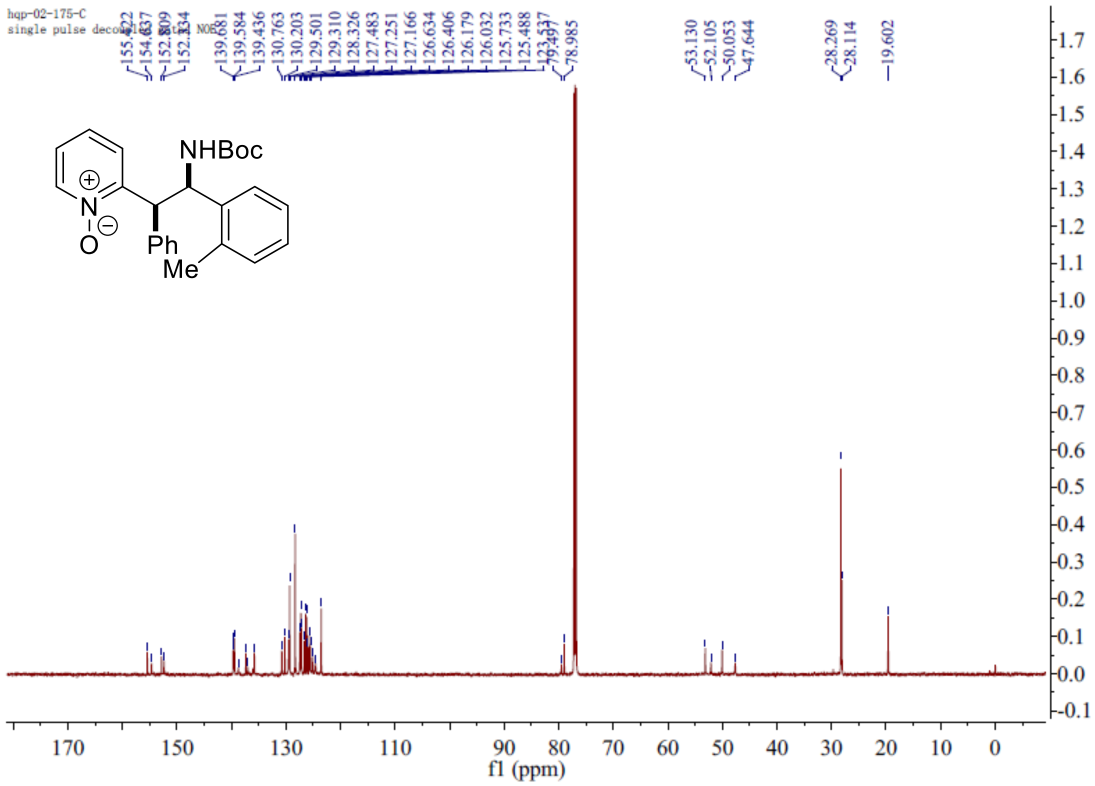

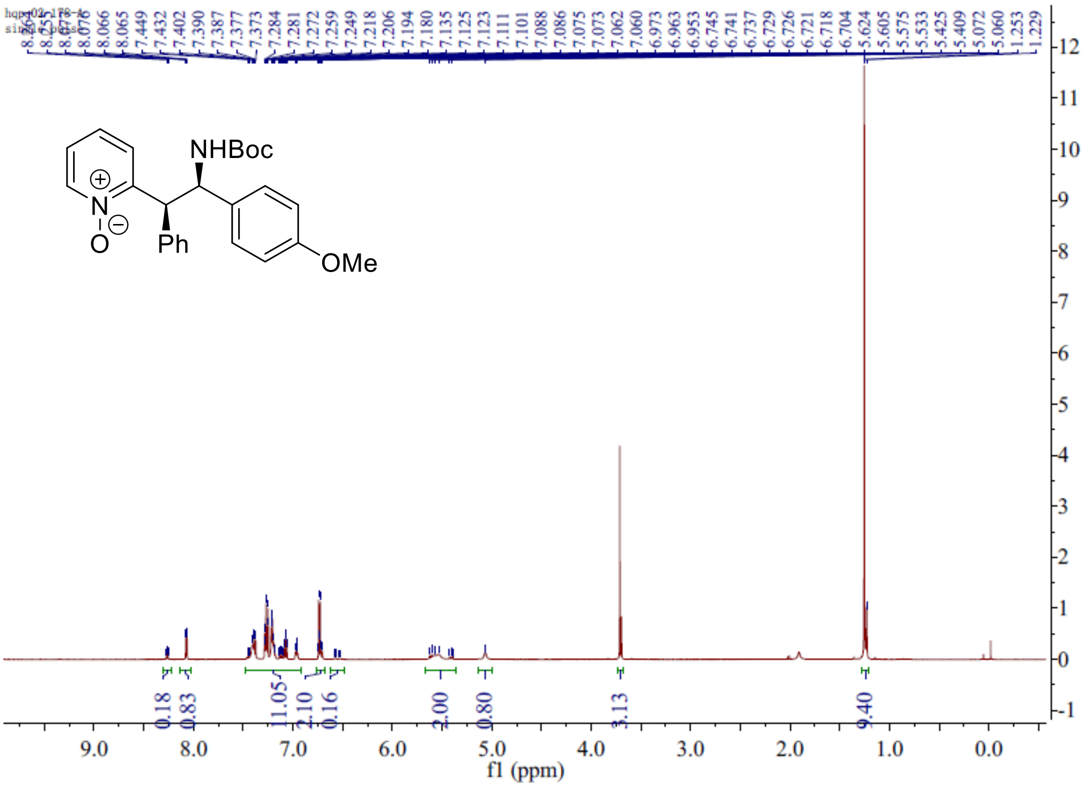
hqp-02-178-A
single pulse
NHBoc
N
O
Ph
OMe
0.18
0.83
11.05
2.10
0.16
2.00
0.80
3.13
9.40
f1 (ppm)

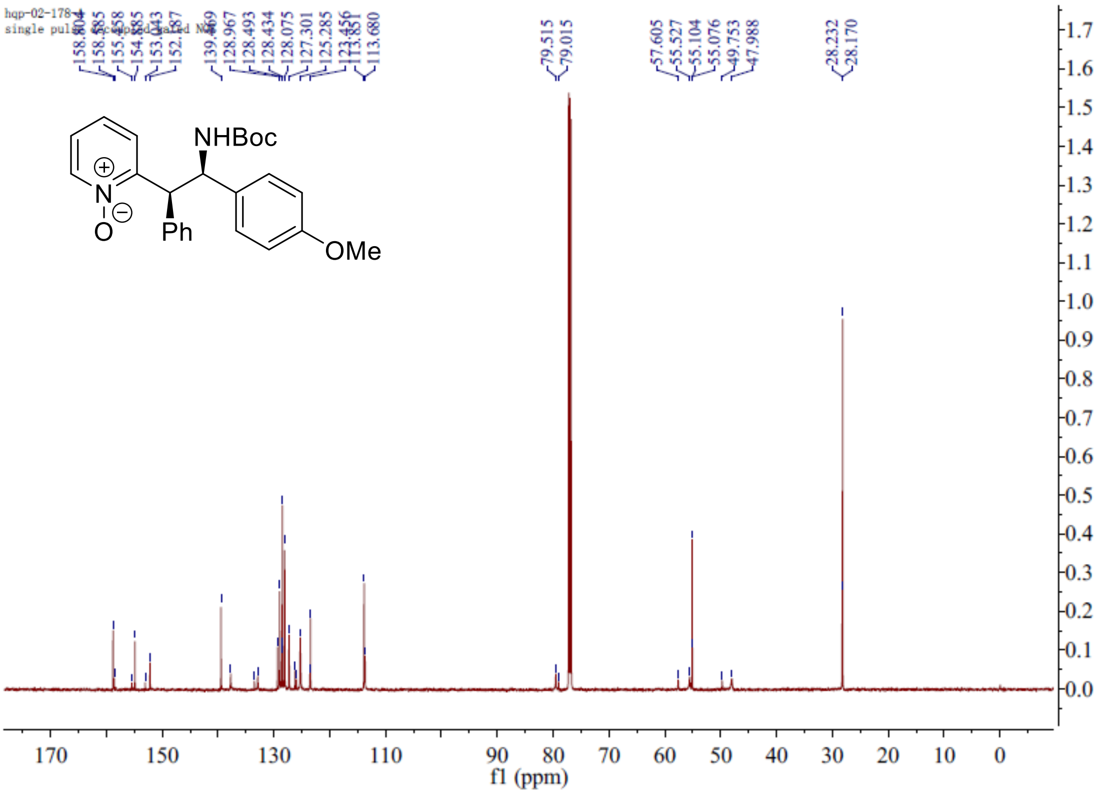
hqp-02-178-A
NHBoc
N
O
Ph
OMe
79.515
79.015
57.605
55.527
55.104
55.076
49.753
47.988
28.232
28.170
f1 (ppm)

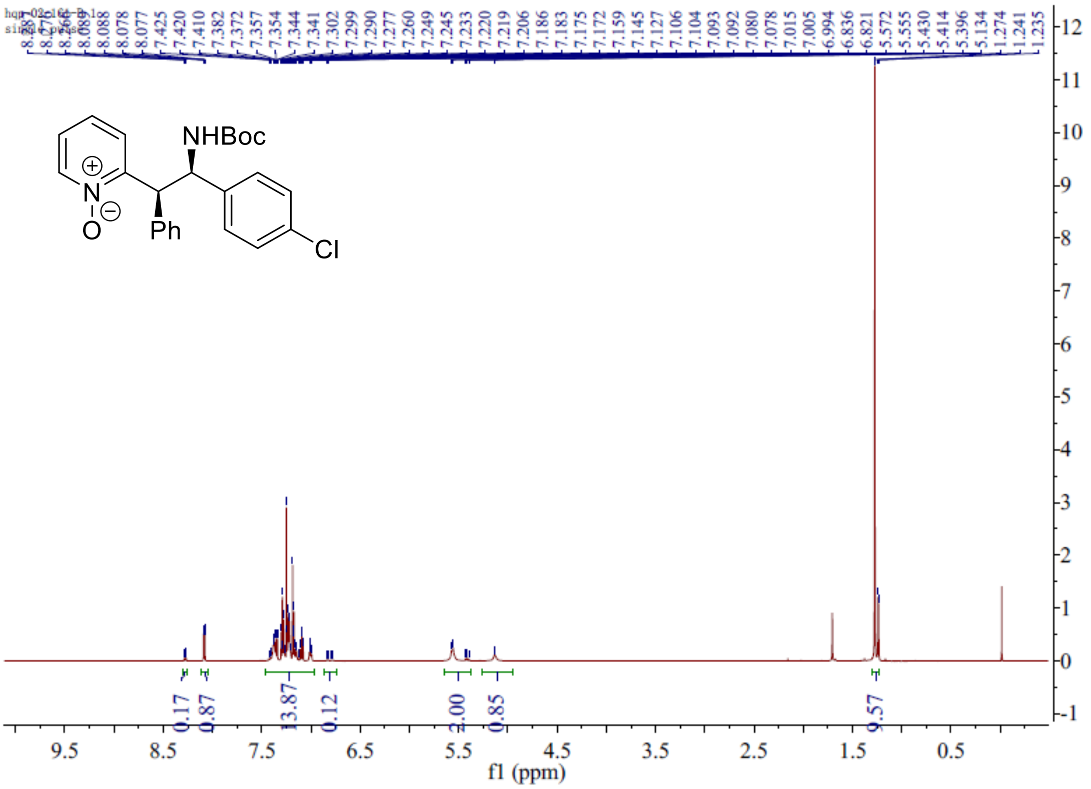
NHBoc
N
O
Ph
Cl
8.088
8.078
8.077
7.425
7.420
7.410
7.382
7.372
7.357
7.354
7.344
7.341
7.302
7.299
7.290
7.277
7.260
7.249
7.245
7.233
7.220
7.219
7.206
7.186
7.183
7.175
7.172
7.159
7.145
7.127
7.106
7.104
7.093
7.092
7.080
7.078
7.015
7.005
6.994
6.836
6.821
5.572
5.555
5.430
5.414
5.396
5.134
1.274
1.241
1.235
0.17
0.87
13.87
0.12
2.00
0.85
9.57
f1 (ppm)

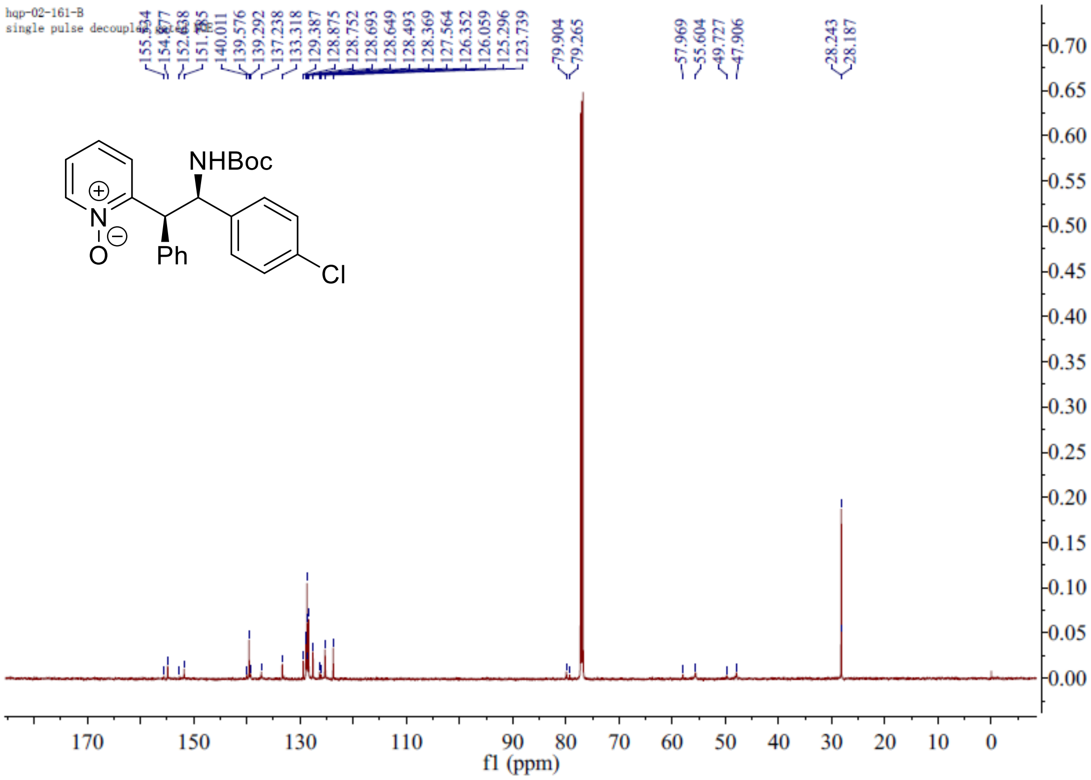
hqp-02-161-B
NHBoc
N
O
Ph
Cl
155.334
154.877
152.638
140.011
139.576
139.292
137.238
133.318
129.387
128.875
128.752
128.693
128.649
128.493
128.369
127.564
126.352
126.059
125.296
123.739
79.904
79.265
57.969
55.604
49.727
47.906
28.243
28.187
f1 (ppm)

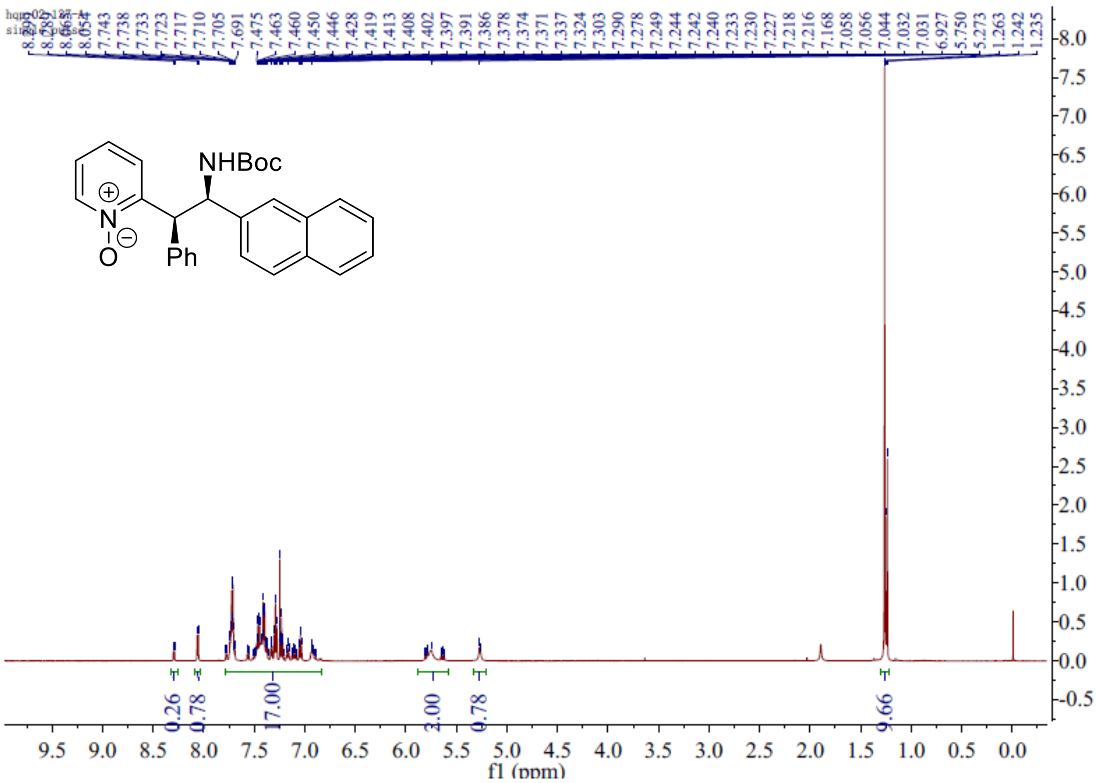
hqp-02-137-A
NHBoc
N
O
Ph
f1 (ppm)

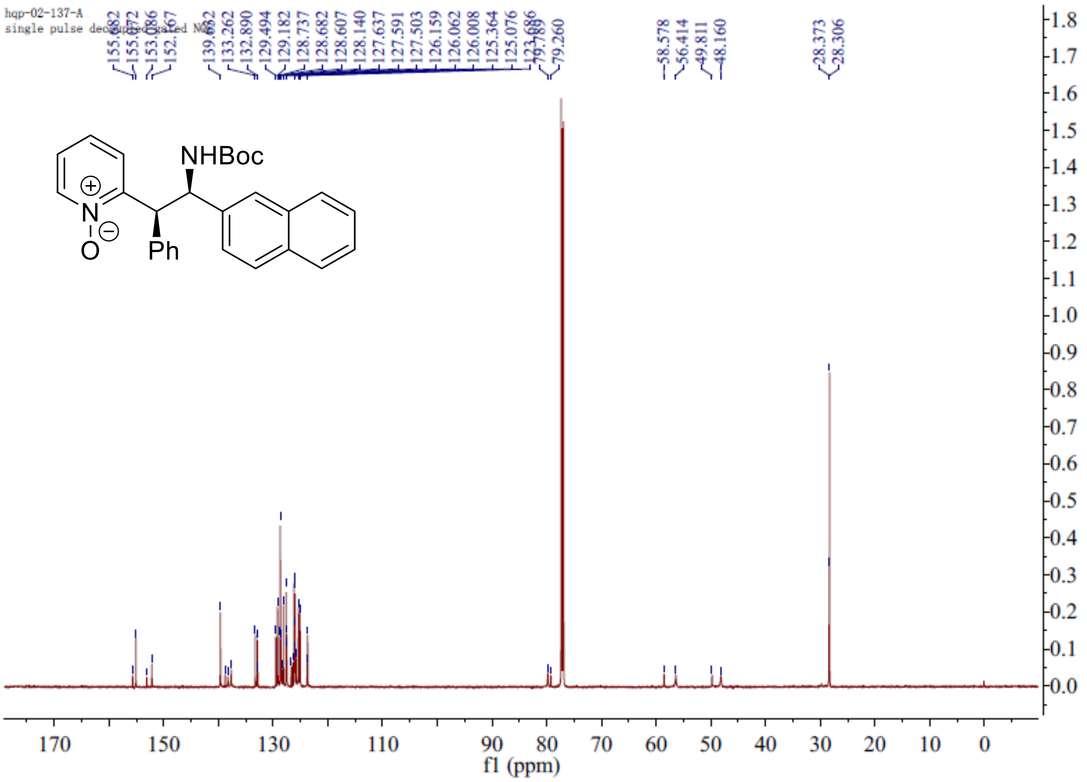
hqp-02-137-A
NHBoc
N
O
Ph
f1 (ppm)

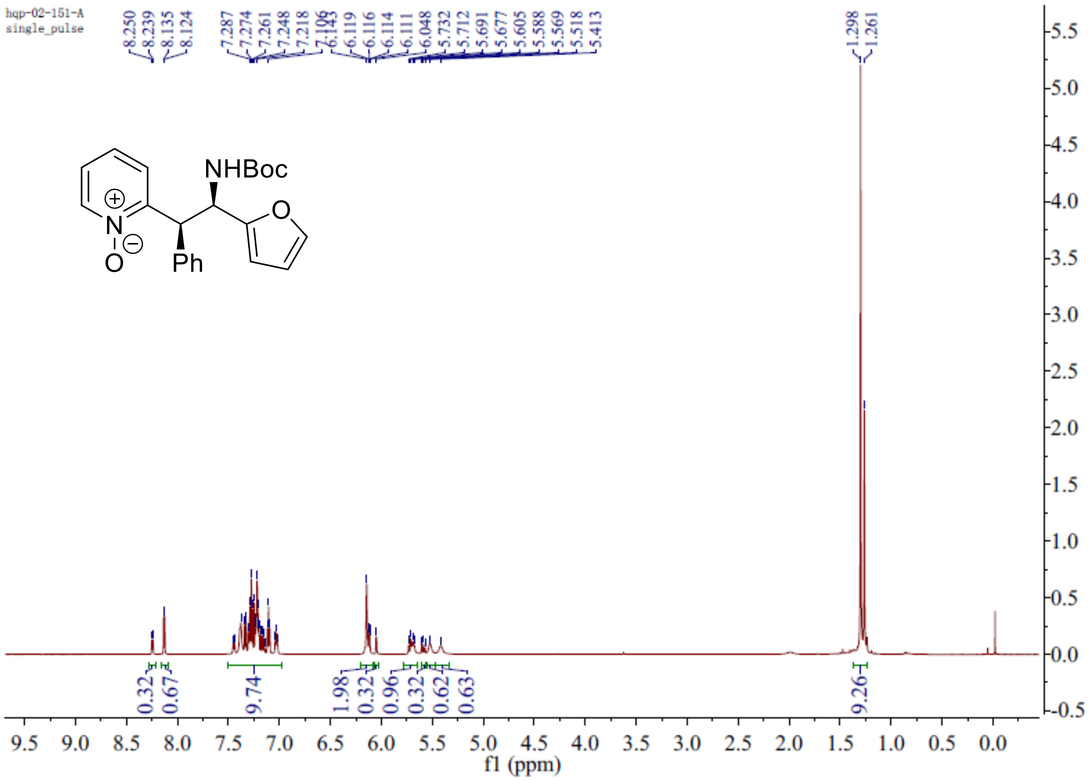

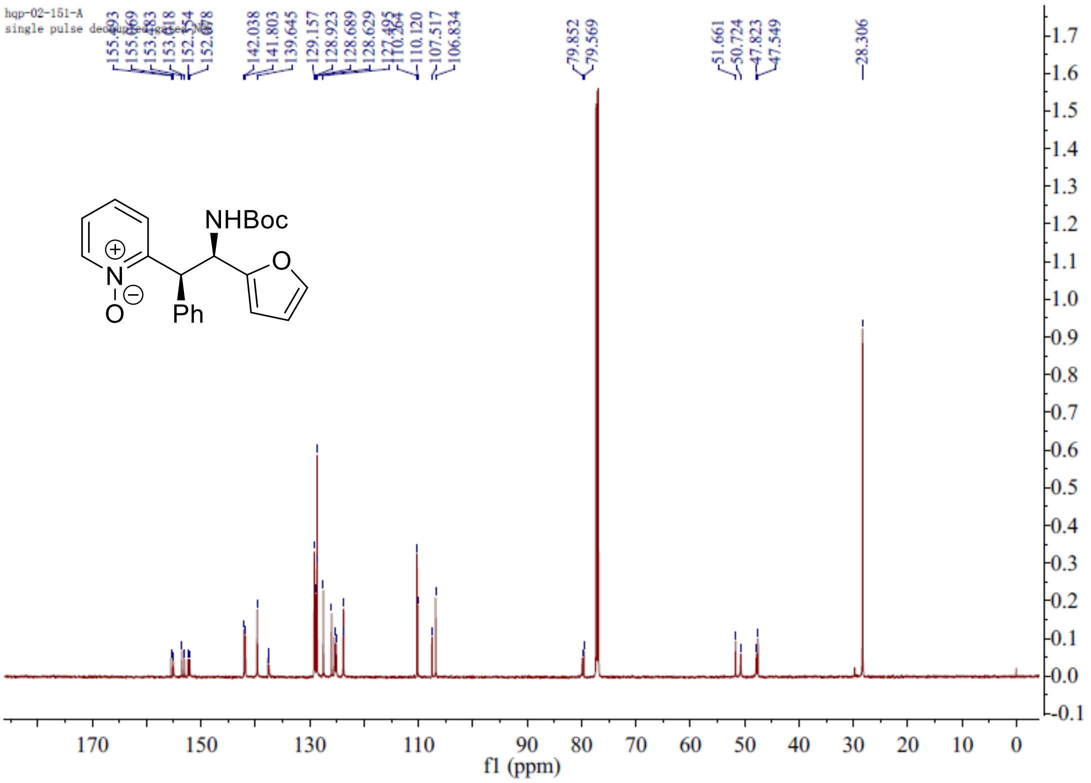

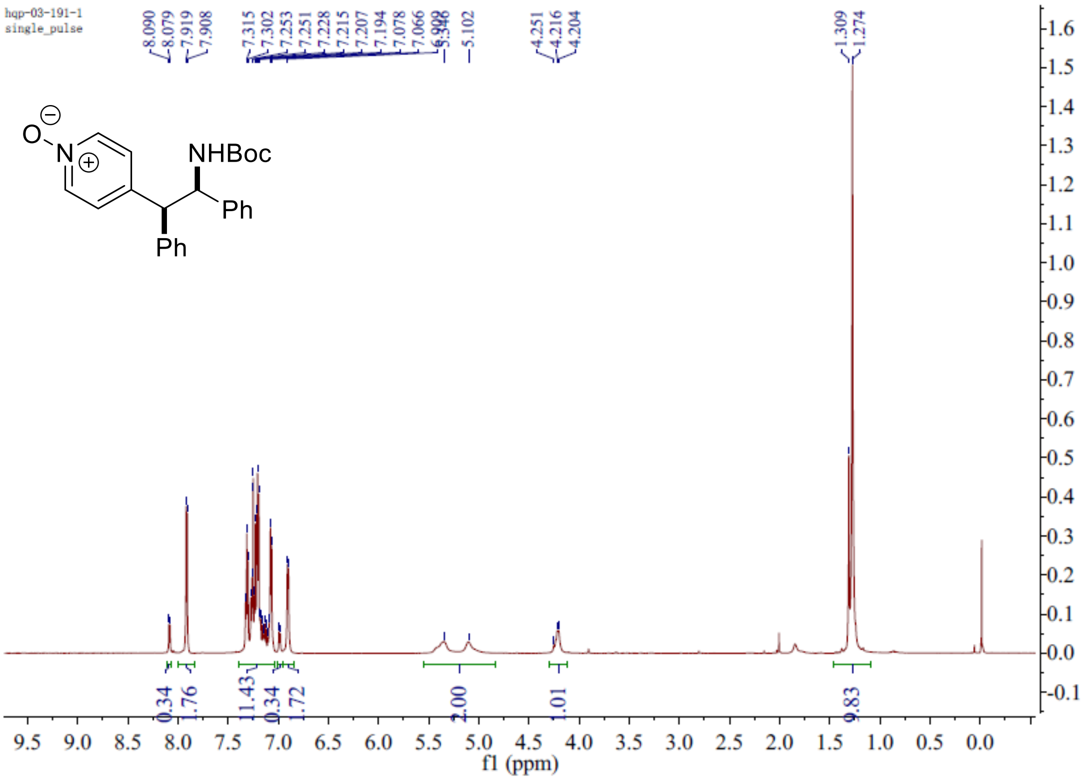
hqp-03-191-1
single_pulse
8.090
8.079
7.919
7.908
7.315
7.302
7.253
7.251
7.228
7.215
7.207
7.194
7.078
7.066
5.102
4.251
4.216
4.204
1.309
1.274
NHBoc
Ph
Ph
0.34
1.76
11.43
0.34
1.72
2.00
1.01
9.83
f1 (ppm)

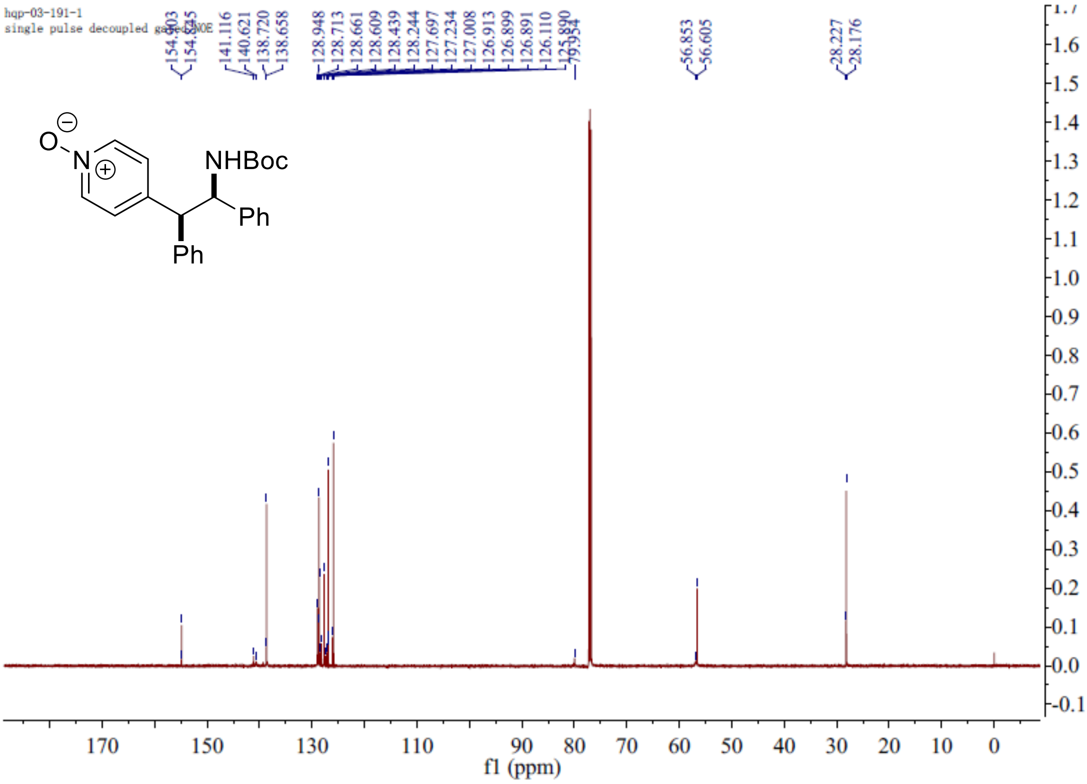
hqp-03-191-1
single pulse decoupled gated NOE
141.116
140.621
138.720
138.658
128.948
128.713
128.661
128.609
128.439
128.244
127.697
127.234
127.008
126.913
126.899
126.891
126.110
79.954
56.853
56.605
28.227
28.176
NHBoc
Ph
Ph
f1 (ppm)

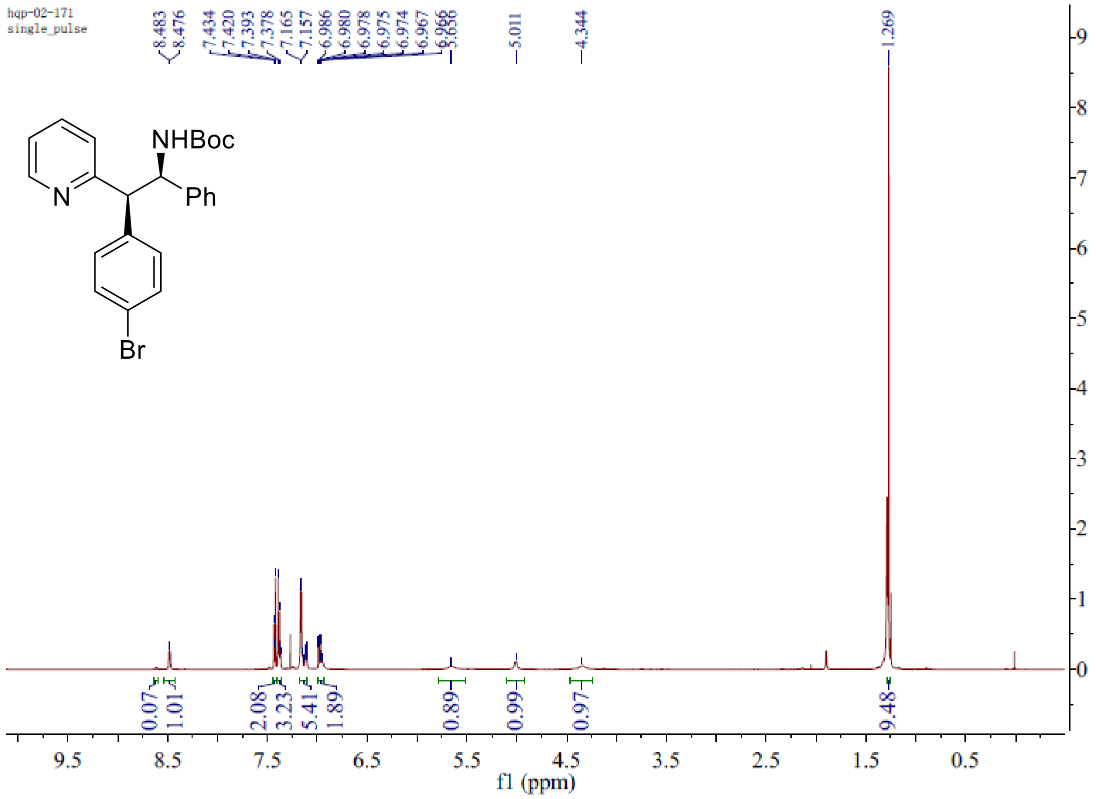

hqp-02-171
single_pulse
NHBoc
Ph
N
Br
8.483
8.476
7.434
7.420
7.393
7.378
7.165
7.157
6.986
6.980
6.978
6.975
6.974
6.967
5.011
4.344
1.269
0.07
1.01
2.08
3.23
5.41
1.89
0.89
0.99
0.97
9.48
f1 (ppm)

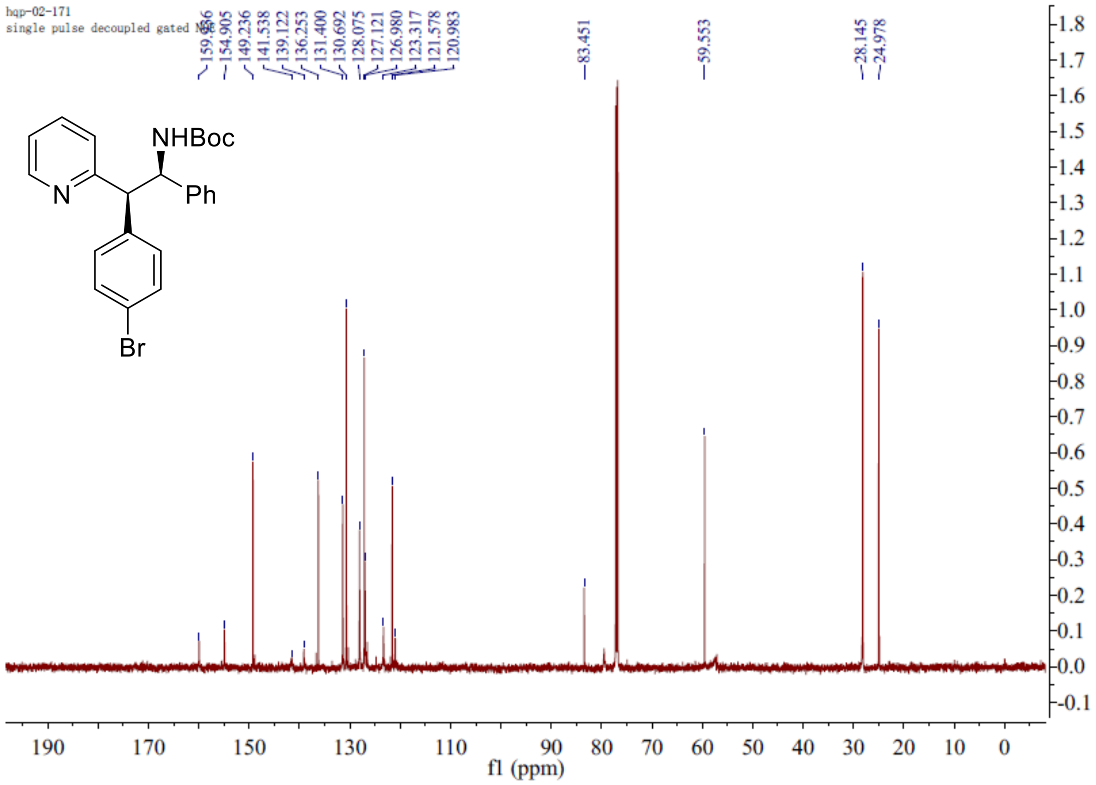

hqp-02-171
single pulse decoupled gated
NHBoc
Ph
N
Br
149.236
141.538
139.122
136.253
131.400
130.692
128.075
127.121
126.980
123.317
121.578
120.983
83.451
59.553
28.145
24.978
f1 (ppm)

9. Referances

[1] T. Niwa, H. Yorimitsu and K. Oshima, *Angew. Chem. Int. Ed.*, 2007, **46**, 2643-2645.

[2] W. M. Dean, M. Šiaučiulis, T. E. Storr, W. Lewis and R. A. Stockman, *Angew. Chem. Int. Ed.*, 2016, **55**, 10013-10016.

[3] H. Ohmiya, H. Yorimitsu and K. Oshima, *Chem. Lett.*, 2004, **33**, 1240-1241.

[4] R. Shang, Z. Yang, Y. Wang, S. Zhang and L. Liu, *J. Am. Chem. Soc.*, 2010, **132**, 14391-14393.

[5] W. Jin, P. Zheng, W.-T. Wong, G.-L. Law, *Adv. Synth. Catal.*, 2017, **359**, 1588-1593.

[6] L. V. Desai, K. J. Stowers and M. S. Sanford, *J. Am. Chem. Soc.*, 2008, **130**, 13285-13293.

[7] T. Suehiro, T. Niwa, H. Yorimitsu and K Oshima, *Chem. Asian J.*, 2009, **4**, 1217-1220.

[8] E. Kianmehr, N. Faghih and K. M. Khan, *Org. Lett.*, 2015, **17**, 414-417.

[9] D. J. Schipper, L. Campeau and K. Fagnou, *Tetrahedron*, 2009, **65**, 3155-3164.
